# Evaluation of Novel Isatin–Schiff Base Derivatives: Monoamine Oxidase Inhibition and Antimicrobial Assessment

**DOI:** 10.1002/cmdc.70437

**Published:** 2026-08-15

**Authors:** Marthinus J. Jooste, Stephanus J. Cloete, Anél Petzer, Jacobus P. Petzer, Theunis T. Cloete

**Affiliations:** ^1^ Centre of Excellence for Pharmaceutical Sciences North‐West University Potchefstroom South Africa; ^2^ Pharmaceutical Chemistry School of Pharmacy North‐West University Potchefstroom South Africa

**Keywords:** depression, isatin, minimum inhibitory concentration, monoamine oxidase, Parkinson's disease, Schiff base, urease

## Abstract

Five novel series of isatin–Schiff base derivatives (**1a–5e**) were synthesized, and their neuroprotective and anti‐infective potential was evaluated. Their half‐maximal inhibitory concentration (IC_50_) was determined against both monoamine oxidase A (MAO‐A) and MAO‐B targets for the treatment of depression and Parkinson's disease, respectively. The anti‐infective potential of the synthesized derivatives was evaluated by measuring their IC_50_ values against urease, an enzyme utilized by specific pathogenic bacteria as a virulence factor. The minimum inhibitory concentration for each compound was assessed against a panel of human pathogens. Most derivatives had potent in vitro activity against MAO‐A and MAO‐B, even greater than the positive control, isatin. The 4‐chloroisatin substituted series (**2a**–**d**) showed exceptional MAO‐A inhibition (**2d**: 0.040 ± 0.002 µM), while the unsubstituted, 5‐, 6‐, and 7‐chloroisatin analogues (**1a**–**e**, **3a**–**c**, **4a**–**b**, and **5a**–**e**) demonstrated high potency and selectivity toward MAO‐B, the most active being **5d** (0.018 ± 0.003 µM; selectivity index = 15 389). Kinetic analysis confirmed a competitive and reversible mode of inhibition. None of the derivatives showed urease inhibition or significant antibacterial activity. These findings highlight the isatin–Schiff base derivatives as promising agents for potent and selective MAO inhibitors with therapeutic potential for neurodegenerative disorders.

## Graphical abstract

Five novel series of isatin–Schiff base derivatives are synthesized and evaluated for neuroprotective and anti‐infective potential. The compounds demonstrate exceptional, reversible, and competitive monoamine oxidase inhibition, with chloro‐substitutions dictating isoform selectivity. Although antibacterial activity remains poor due to limited aqueous solubility, these findings validate the scaffold as promising early‐stage neuroprotective leads.

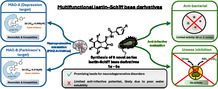

## 1 Introduction

**FIGURE 1:**
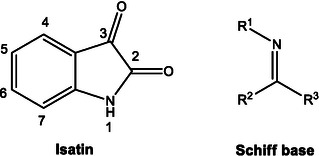
Important structures discussed in the text.

Isatin (1*H*‐indole‐2,3‐dione), first synthesized more than 170 years ago by Erdmann and Laurent [1], has since been isolated from various natural sources, including plants, animals, and humans [2, 3]. Isatin is an indole‐based compound and offers multiple sites for derivatization at N‐1, C‐2, C‐3, and the aromatic ring system (Figure 1) [4], enabling various modifications to modulate reactivity and biological affinity. Isatin and its derivatives have an extensive array of pharmacological properties, including antibacterial, anti‐HIV [5], anticancer, anticonvulsant [6], antifungal [7], anti‐inflammatory [8], antimalarial [5, 9], antioxidant [10], antitubercular [11], and antiviral activities [12]. Isatin can also inhibit monoamine oxidase A (MAO‐A) and MAO‐B targets for depression and Parkinson's disease (PD), respectively [13, 14]. Substitutions at the C‐5 and C‐6 positions of isatin significantly influence the binding affinity for MAO and can even induce selective inhibition of MAO‐B [13, 15, 16]. In addition, isatin also inhibits the urease enzyme, a known bacterial virulence factor [17].

Synthetically, Schiff bases (Figure 1) are produced through the condensation of an amine with an active carbonyl compound, resulting in the formation of a signature (C=N) double bond [18]. These compounds have a wide range of biological activities, including antibacterial [19], anticancer [20], trypanocidal, anti‐HIV, antimalarial [21], and anti‐inflammatory effects [22]. Schiff bases have also been reported to inhibit several enzymes of therapeutic relevance, such as acetylcholinesterase, tyrosinase, and urease [21, 23, 24].

MAO‐A and MAO‐B are flavoenzymes that oxidatively deaminate neurotransmitters and xenobiotic amines. These two isoforms differ in their susceptibility to inhibitors and also in their selectivity toward the catabolism of different neurotransmitters [25]. MAO‐A is responsible for metabolizing serotonin and noradrenaline, while MAO‐B preferentially metabolizes the trace amines phenethylamine and methylhistamine. The metabolic degradation of dopamine is facilitated by both MAO‐A and MAO‐B isoforms [25]. Furthermore, the upregulation of these enzymes is associated with various neurological and psychiatric conditions, including depression, Alzheimer's disease, and PD [26, 27]. Isatin reversibly inhibits both MAO‐A and MAO‐B, and modifications at the C‐5 and C‐6 positions have yielded compounds with improved isoform selectivity and potency [13].

A safety concern with MAO inhibition is a hypertensive crisis called the cheese reaction, which results from ingesting food rich in tyramine while simultaneously taking an MAO‐A inhibitor [28]. If MAO‐A, which usually metabolizes tyramine, is inhibited with an irreversible MAO‐A inhibitor, tyramine can enter the systemic circulation, resulting in the release of dopamine and noradrenaline triggering a hypertensive reaction [29]. Selective MAO‐B inhibitors have a decreased chance of causing a cheese reaction and are considered safer [30].

MAO‐B is a target enzyme for the treatment of PD, a neurodegenerative disease which results from the loss of dopaminergic neurons specifically within the substantia nigra [31]. PD is a multisystem heterogeneous disease with a complex symptom profile; however, most notably it is characterized by diminished postural quality, gait impairment, muscular rigidity, resting tremors, and bradykinesia [32, 33]. PD is unfortunately progressive, and although treatment can alleviate the symptoms, the disease will eventually have a fatal clinical outcome [32]. In the early stage of the disease, selective MAO‐B inhibitors, i.e., rasagiline and selegiline, are used as monotherapy to increase the dopamine concentration [31]. As the disease progresses, MAO‐B inhibitors are used in combination with levodopa to alleviate the motor complications [31]. In the advanced and end‐of‐life stages, other complications include aspiration pneumonia, pain, and infection [32]. These complications underscore the potential of multitarget drugs (MTDs) to address both the primary pathology as well complications associated with PD.

The development, progression, and therapeutic management of various neuropsychiatric disorders, most notably depression, are heavily reliant upon the activity of MAO‐A [34]. Depression is a psychiatric condition characterized by disrupted neurovegetative functions (e.g., appetite and sleep cycles) alongside profound mood disturbances, typically manifesting as excessive guilt and a significant loss of self‐esteem [35]. When MAO‐A is overexpressed in the brain, serotonin and noradrenaline are metabolized to a greater extent, resulting in depression. Consequently, MAO‐A inhibitors, e.g., moclobemide, are commonly used to treat depression [34].

Antimicrobial resistance (AMR) results when a bacterium that was once susceptible to an antibiotic develops resistance mechanisms, rendering it less susceptible [36]. AMR is a natural occurrence resulting from selective pressure when exposing a pathogen to an antibiotic [37]. AMR is unfortunately accelerated by the over‐ and misuse of antibiotics [37]. The World Health Organization (WHO) has designated 12 bacterial families as priority pathogens, highlighting an urgent need for the development of new antibiotics [38]. Among these priority pathogens, the ESKAPE pathogens are known to rapidly develop resistance against antibiotics and cause nosocomial infections [39]. The ESKAPE pathogens refers to: *Enterococcus faecium*, *Staphylococcus aureus*, *Klebsiella pneumoniae*, *Acinetobacter baumannii*, *Pseudomonas aeruginosa*, and *Enterobacter* species [39].

The enzymatic hydrolysis of urea into carbamate and ammonia is catalyzed by the nickel‐containing enzyme urease (EC 3.5.1.5), which represents a critical component in the virulence mechanisms of diverse pathogens [40]. Urease neutralizes gastric acid, creating a microenvironment that enables colonization of the stomach lining that is beneficial to acid‐sensitive pathogens like *Helicobacter pylori* [41]. In the urinary tract, organisms, such as *Staphylococcus*, *Klebsiella*, *Proteus*, and *Pseudomonas* species, produce urease to hydrolyze urea, elevating urinary pH and forming struvite and calcium phosphate stones that protect bacteria and promote infection [42]. Even *Mycobacterium tuberculosis* uses urease as a source of nitrogen, allowing the pathogen to survive in nutrient deficient environments [43].

As mentioned above, both isatin and Schiff base derivatives have antibacterial activity [5, 44], highlighting their potential as MTDs which has the potential to treat not only depression and PD, but also the bacterial infections associated with late‐stage PD [32]. Because of the complex pathology of neurodegenerative disorders, some researchers suggest that MTDs might be superior to the standard “one‐drug, one‐target” approach [45].

The aim of this study was to develop five novel series of isatin–Schiff base derivatives and characterize their in vitro profiles as MAO inhibitors, antibacterial agents, and urease inhibitors. Inhibitory activities against MAO‐A and MAO‐B were determined to evaluate their neuroprotective potential. To determine the molecular basis of the in vitro activities, the most potent derivatives were subjected to molecular docking simulations within the active sites of their respective isoforms. Because bacterial infection is a complication of late‐stage PD, their anti‐infective capacity was assessed against *S. aureus*, *K. pneumoniae*, *P. aeruginosa*, and *Klebsiella aerogenes*, along with urease inhibition studies to investigate their ability to inhibit this critical bacterial virulence factor. This study aims to validate the potential of isatin–Schiff base derivatives as early‐stage leads for the treatment of neurodegenerative disorders and bacterial infections.

## 2 Results and Discussion

### 2.1 Synthesis of the Isatin–Schiff Base Derivatives

**SCHEME 1:**
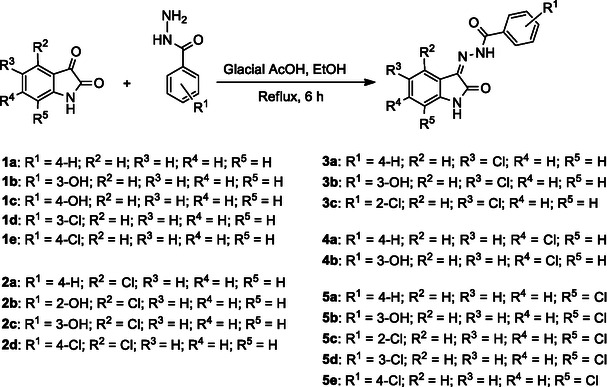
Synthetic route for the synthesis of the target derivatives, **1a**–**e**, **2a**–**d**, **3a**–**c**, **4a**–**b**, and **5a**–**e**. Reagents were glacial acetic acid (AcOH) and ethanol (EtOH).

All derivatives were successfully synthesized (Scheme 1), as determined by nuclear magnetic resonance (NMR) and high‐resolution mass spectrometry (HRMS) and the purity was confirmed by high‐performance liquid chromatography (HPLC). The purity of the derivatives ranged between 90% and 100%. Considering the carbon (^13^C) NMR spectrum, it was anticipated that the signals for the carbonyl carbons (C‐8 and C‐13) and the imine carbon (C‐7) would be found at ≈δ 163 ppm and δ 138 ppm, respectively. As these are nonprotonated carbons, they are known to exhibit weak signals due to long spin‐lattice relaxation times (*T*_1_) and a reduced nuclear Overhauser effect [46]. Consequently, only one of the two expected carbonyl signals was resolved. Analysis of the heteronuclear multiple bond correlation spectrum for derivative **1a** (Supporting Data) confirmed that the observed signal corresponds to C‐13, while the signal for C‐8 was not detected. The imine carbon (C‐7) also presented as a characteristically weak signal that was not consistently observed in the ^13^C spectrum of the compounds. The fully resolved proton (^1^H), ^13^C, and heteronuclear single‐quantum correlation (HSQC) NMR spectra for all synthesized derivatives, alongside the corresponding HPLC chromatograms and HRMS spectra, are provided in the Supporting Information.

Several derivatives exhibited poor water solubility during synthesis, purification, and in vitro assays, an observation that was confirmed by in silico *LogS* calculations (Table 1). Based on the *LogS* scale, 11 derivatives were predicted to have poor solubility and 8 to have moderate solubility. The poor solubility of the derivatives proved problematic during the broth microdilution assay when the high concentrations were evaluated in the antibacterial test, i.e., 128 µg/mL.

**TABLE 1:** Table of the aqueous solubility (*LogS*) of the isatin–Schiff base derivatives (**1a**–**5e**).

Derivative	***LogS***	Predicted solubility class
**1a**	−5.44	Moderately soluble
**1b**	−4.86	Moderately soluble
**1c**	−4.86	Moderately soluble
**1d**	−6.04	Poorly soluble
**1e**	−6.04	Poorly soluble
**2a**	−6.04	Poorly soluble
**2b**	−5.46	Moderately soluble
**2c**	−5.46	Moderately soluble
**2d**	−6.64	Poorly soluble
**3a**	−6.04	Poorly soluble
**3b**	−5.46	Moderately soluble
**3c**	−6.64	Poorly soluble
**4a**	−6.04	Poorly soluble
**4b**	−5.46	Moderately soluble
**5a**	−6.04	Poorly soluble
**5b**	−5.46	Moderately soluble
**5c**	−6.64	Poorly soluble
**5d**	−6.64	Poorly soluble
**5e**	−6.64	Poorly soluble

### 2.2 In Vitro Monoamine Oxidase (MAO) Inhibition

**FIGURE 2:**
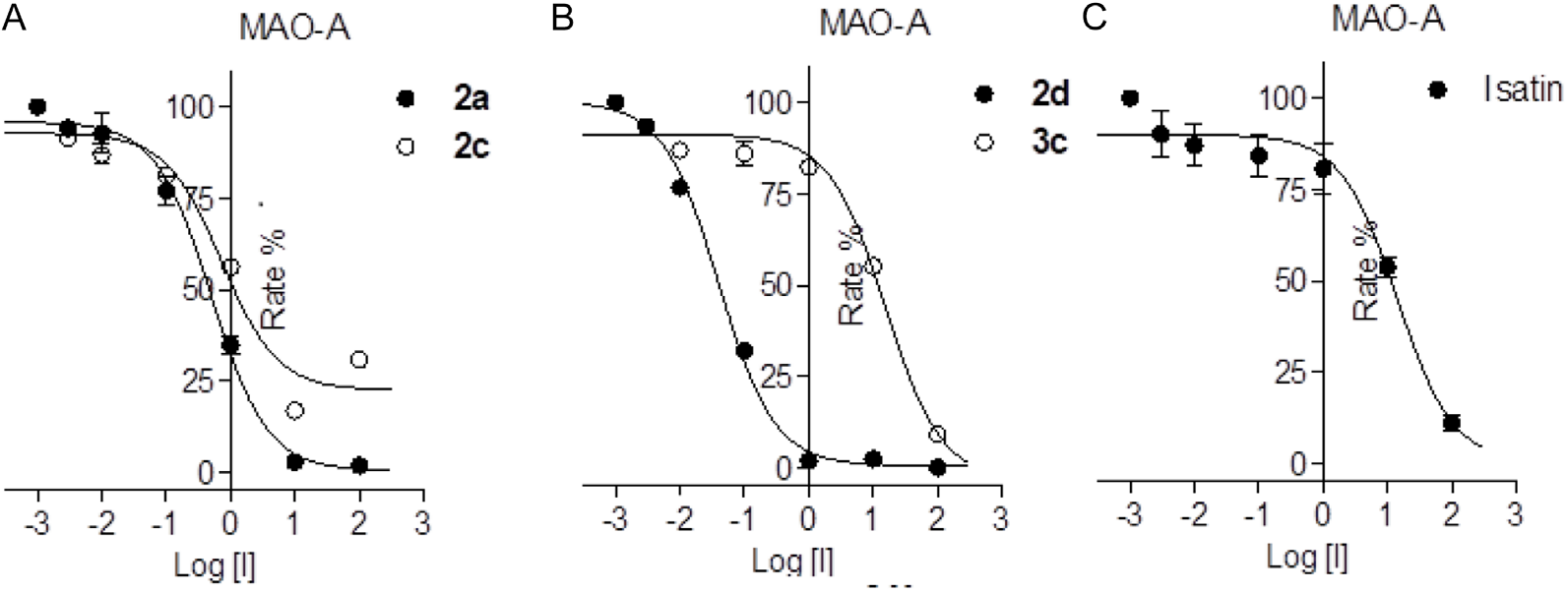
Examples of sigmoidal plots of the inhibition of MAO‐A by selected derivatives, i.e., **2a**, **2c**, **2d, 3c**, and isatin.

**FIGURE 3:**
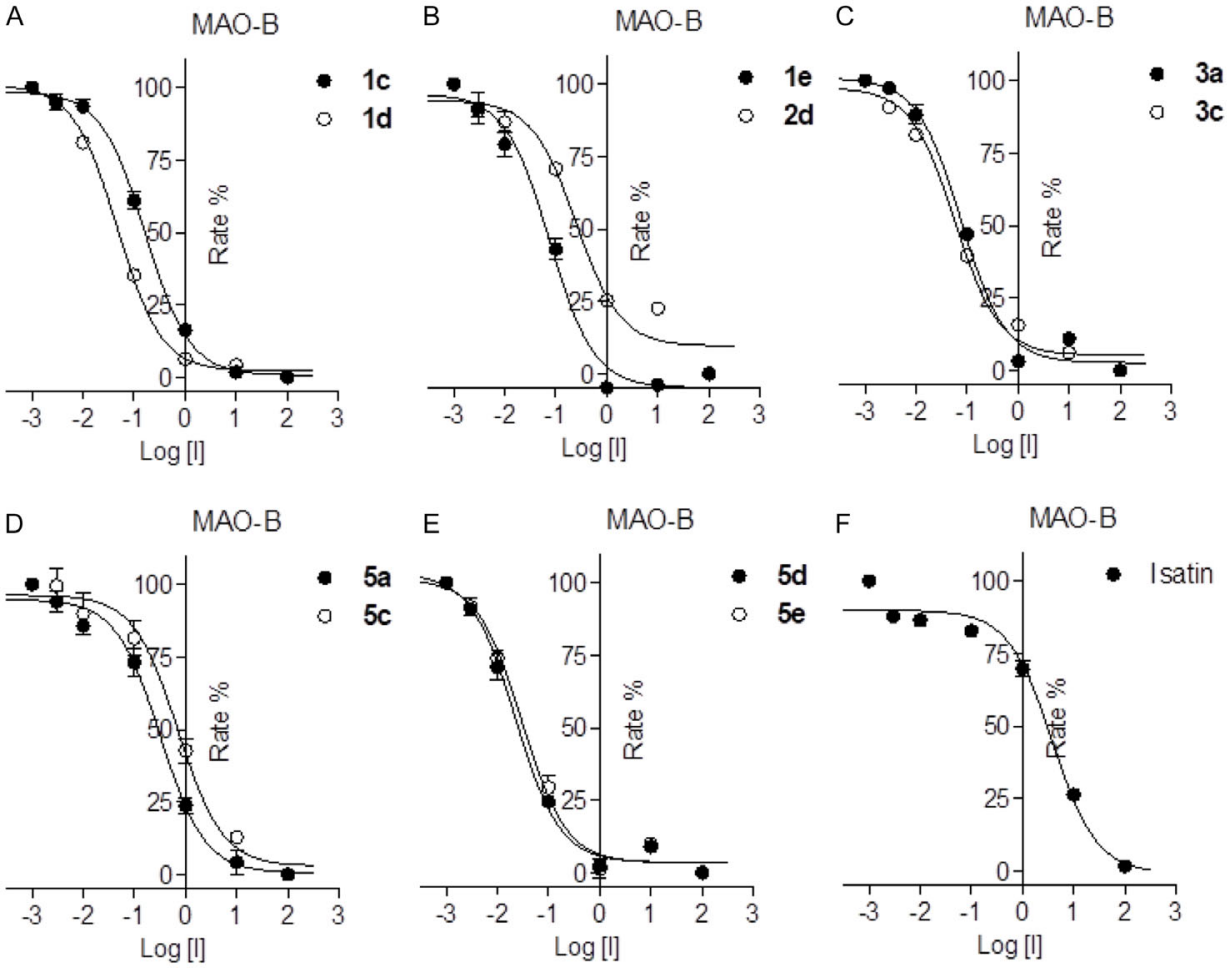
Examples of sigmoidal plots of the inhibition of MAO‐B by selected derivatives, i.e., **1c**, **1d**, **1e**, **2d**, **3a**, **3c**, **5a**, **5c**, **5d**, **5e**, and isatin.

**FIGURE 4:**
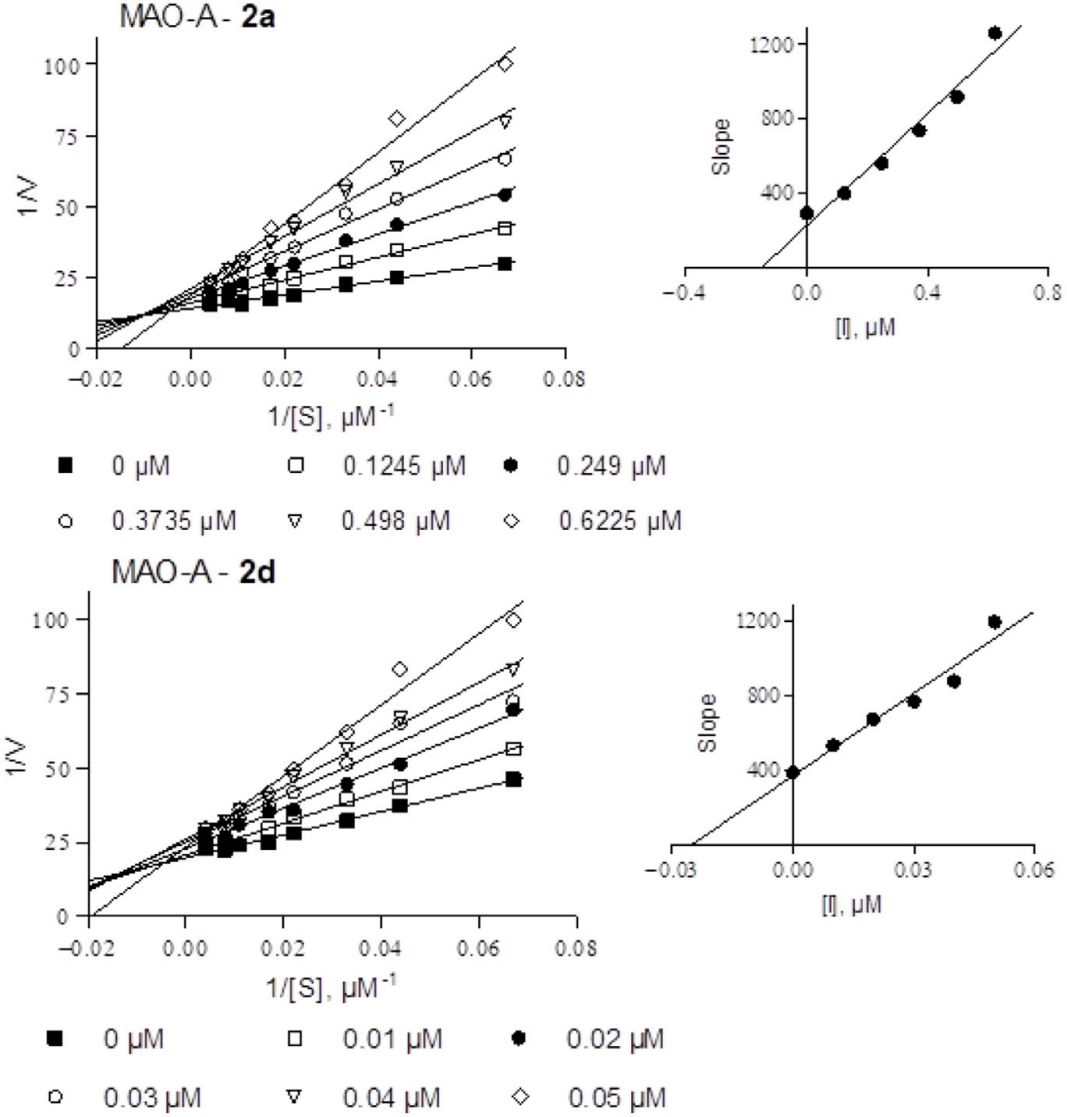
Lineweaver–Burk plots for the inhibition of human MAO‐A. Top: **2a** (*K* *_i_* = 0.15 µM); bottom: **2d** (*K* *_i_* = 0.025 µM).

**FIGURE 5:**
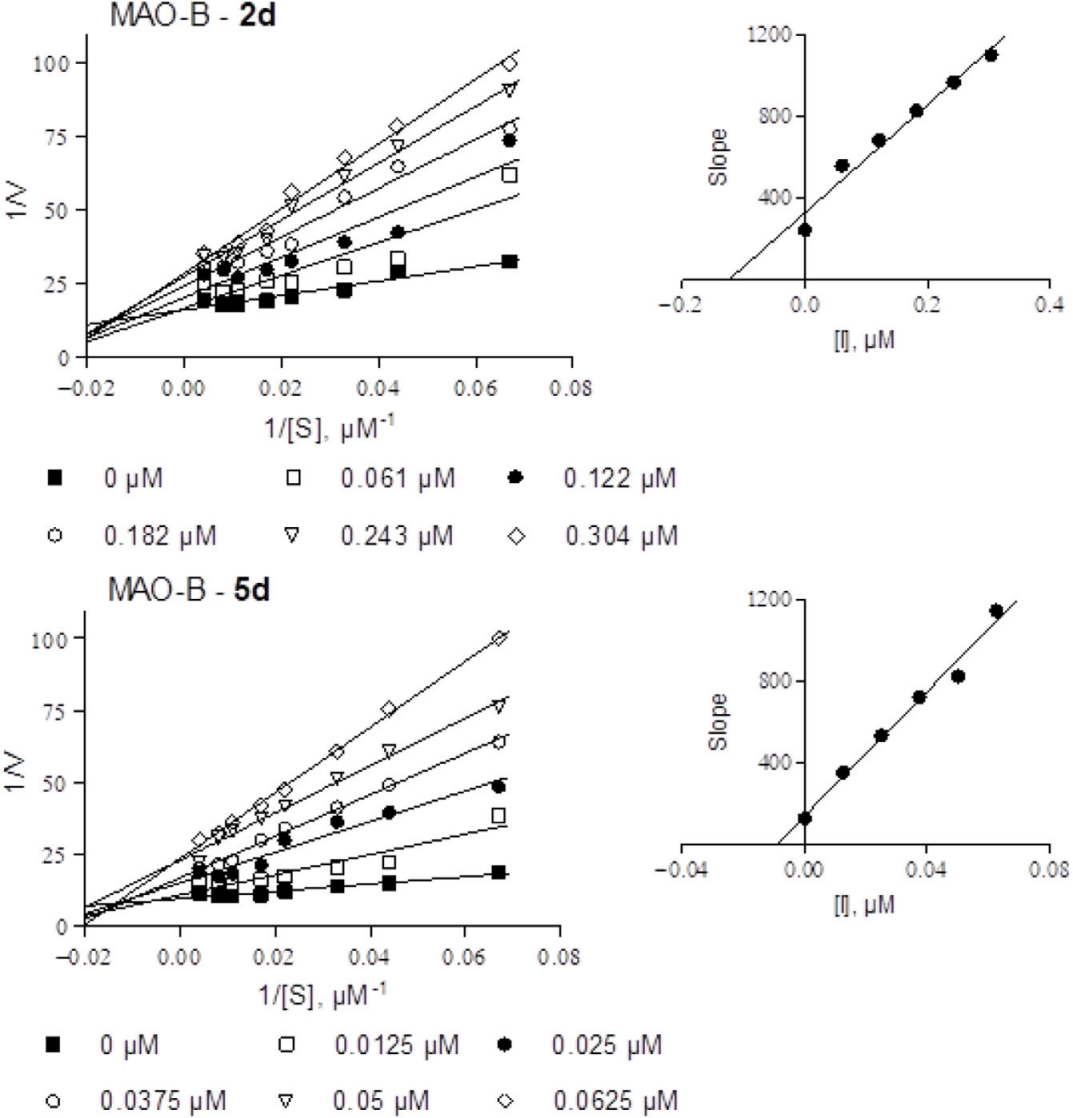
Lineweaver–Burk plots for the inhibition of human MAO‐B. Top: **2d** (*K* *_i_* = 0.12 µM); bottom: **5d** (*K* *_i_* = 0.009 µM).

The in vitro half‐maximal inhibitory concentration (IC_50_) values of the derivatives against both MAO isoforms are shown in Table 2 and the sigmoidal plots that were used to determine the IC_50_ values of some of the derivatives are shown in Figure 2 (MAO‐A) and Figure 3 (MAO‐B). In addition to the IC_50_ values, Lineweaver–Burk plots were constructed to determine the mode of inhibition for **2a** and **2d** against MAO‐A (Figure 4) and **2d** and **5d** against MAO‐B (Figure 5).

**TABLE 2:** IC_50_ values of target derivatives against MAO‐A and MAO‐B.

Derivative	MAO‐A, µM ± SD	MAO‐B, µM ± SD	**SI** a
**1a**	13.7 ± 1.48	1.43 ± 0.127	9.6
**1b**	27.0 ± 3.75	2.68 ± 1.20	10.1
**1c**	7.19 ±71.78	0.137 ± 0.020	52.5
**1d**	26.3 ± 0.707	0.037 ± 0.001	710
**1e**	15.8 ± 1.15	0.065 ± 0.009	243
**2a**	0.498 ± 0.054	1.41 ± 0.072	0.35
**2b**	1.14 ± 0.020	15.5 ± 0.304	0.07
**2c**	1.49 ± 0.100	7.45 ± 0.499	0.20
**2d**	0.040 ± 0.002	0.243 ± 0.019	0.16
**3a**	62.4 ± 11.1	0.067 ± 0.001	931
**3b**	6.86 ± 2.18	0.056 ± 0.006	122
**3c**	14.2 ± 0.692	0.044 ± 0.001	322
**4a**	50.5 ± 0.580	30.7 ± 1.38	1.6
**4b**	59.1 ± 13.6	0.180 ± 0.004	328
**5a**	33.4 ± 13.0	0.349 ± 0.057	95.7
**5b**	36.1 ± 2.45	1.16 ± 0.126	31.1
**5c**	1.73 ± 0.424	0.473 ± 0.069	3.7
**5d**	277 ± 65.5	0.018 ± 0.003	15,389
**5e**	39.5 ± 5.13	0.026 ± 0.006	1519
Isatin^b^	14.3 ± 3.37	3.70 ± 0.229	3.9
Safinamide^b^	—	0.176 ± 0.036	—
Methylene blue^b^	0.030 ± 0.0045	—	—

Abbreviation: SD, standard deviation.

a SI: selectivity index (IC_50_ MAO‐A/IC_50_ MAO‐B).

b Positive control.

While all synthesized derivatives were active against MAO‐A, some displayed inhibitory potency that markedly exceeded that of the positive control, isatin (14.3 ± 3.37 µM). However, none were more active than the reference inhibitor methylene blue (0.030 ± 0.0045 µM). Series 2 (**2a**–**d**), which features a 4‐chloro substituent on the isatin scaffold was the most active, i.e., **2a** (0.498 ± 0.054 µM), **2b** (1.14 ± 0.020 µM), **2c** (1.49 ± 0.1 µM), and **2d** (0.040 ± 0.002 µM). In general, series 1 (**1a**–**e**) (unsubstituted) had similar activity compared to series 3 (**3a**–**c**) (5‐chloro), whereas series 4 (**4a**–**b**) (6‐chloro) and series 5 (**5a**–**e**) (7‐chloro) were less active. Comparing the activity of the derivatives within each series, no clear structure–activity relationship could be established regarding the hydroxy‐ and chloro‐substitution on the benzyl ring.

All derivatives also displayed inhibitory activity against MAO‐B, with the majority being more potent than isatin (3.70 ± 0.229 µM) and 8 being more potent than safinamide (0.176 ± 0.036 µM). Series 3 was the most active: **3a** (0.067 ± 0.001 µM), **3b** (0.056 ± 0.006 µM), and **3c** (0.044 ± 0.001 µM); however, series 1 and 5 also had good activity with derivative **5d** being the most potent, i.e., 0.018 ± 0.003 µM. Series 2 and 4 presented with a mixed activity profile, containing some potent inhibitors but also derivatives that were considerably less active than isatin. Kumar and coworkers (2024) synthesized similar isatin derivatives and found that substituting a hydrogen atom on the *meta*‐position of the isatin core (series 1) with a chlorine atom (series 3) increased MAO‐B activity [16]. Similarly, in the current study, derivatives **1a** and **1b** were less active compared to their corresponding chlorine analogues, i.e., **3a** and **3b**. Comparing the activity of the derivatives within each series, the chloro‐substituted derivatives were in general more potent than the hydroxy‐substituted derivatives.

The selectivity index (SI) reveals that most derivatives possess significantly greater inhibitory potency against the MAO‐B isoform compared to MAO‐A. The only exception is observed in series 2, which exhibits a preference for MAO‐A inhibition.

Others have also found that the 4‐chloro‐substitution on isatin results in specific inhibition of MAO‐A, while 5‐chloro‐substitution markedly increases in vitro affinity against MAO‐B compared with 6‐chloro‐substitution [47]. The results discussed in this study as well as others established that the halogenation on the isatin ring can influence inhibitory potency, while the specific substitution positions dictate isoform selectivity [13, 47].

With regard to the substitution on the phenyl ring, Maliyakkal and co‐workers (2024) synthesized similar isatin derivatives with different halogen substituents on the *para*‐position and found that MAO‐B inhibition decreased in the following order; —Cl > —Br > —F > —H > —OH [14]. This confirms the findings in the present study where chloro‐substituted derivatives were more active than their corresponding hydroxy‐substituted counterparts. Because chloro substituents are considerably more lipophilic than hydroxyl substituents [48], this trend highlights the importance of lipophilic substituents on the phenyl ring for increased MAO‐B activity.

To ascertain the binding mechanism, Lineweaver–Burk plots were constructed for selected derivatives, i.e., **2a** and **2d** (MAO‐A) (Figure 4) and **2d** and **5d** (MAO‐B) (Figure 5). It was found that both isoforms of MAO were inhibited competitively by the selected derivatives since the lines of the Lineweaver–Burk plots were linear and intersected near the *y*‐axis. Replots of the slopes of the Lineweaver–Burk plots versus inhibitor concentration yielded linear lines with enzyme–inhibitor inhibition constants (*K* *_i_*) of 0.15 µM (**2a**) and 0.025 µM (**2d**) for MAO‐A and 0.12 µM (**2d**) and 0.009 µM (**5d**) for MAO‐B.

To determine whether the test derivatives inhibit the target enzymes reversibly or irreversibly, the time dependency of inhibition was investigated for derivatives **2a, 2d**, and **5d**. A distinct time‐dependent reduction of MAO activity is anticipated for irreversible inhibitors, such as pargyline (MAO‐A) and (R)‐deprenyl (MAO‐B). In contrast, the degree of enzyme inhibition produced by a reversible inhibitor remains essentially unchanged, regardless of the preincubation time with the enzyme [49, 50].

**FIGURE 6:**
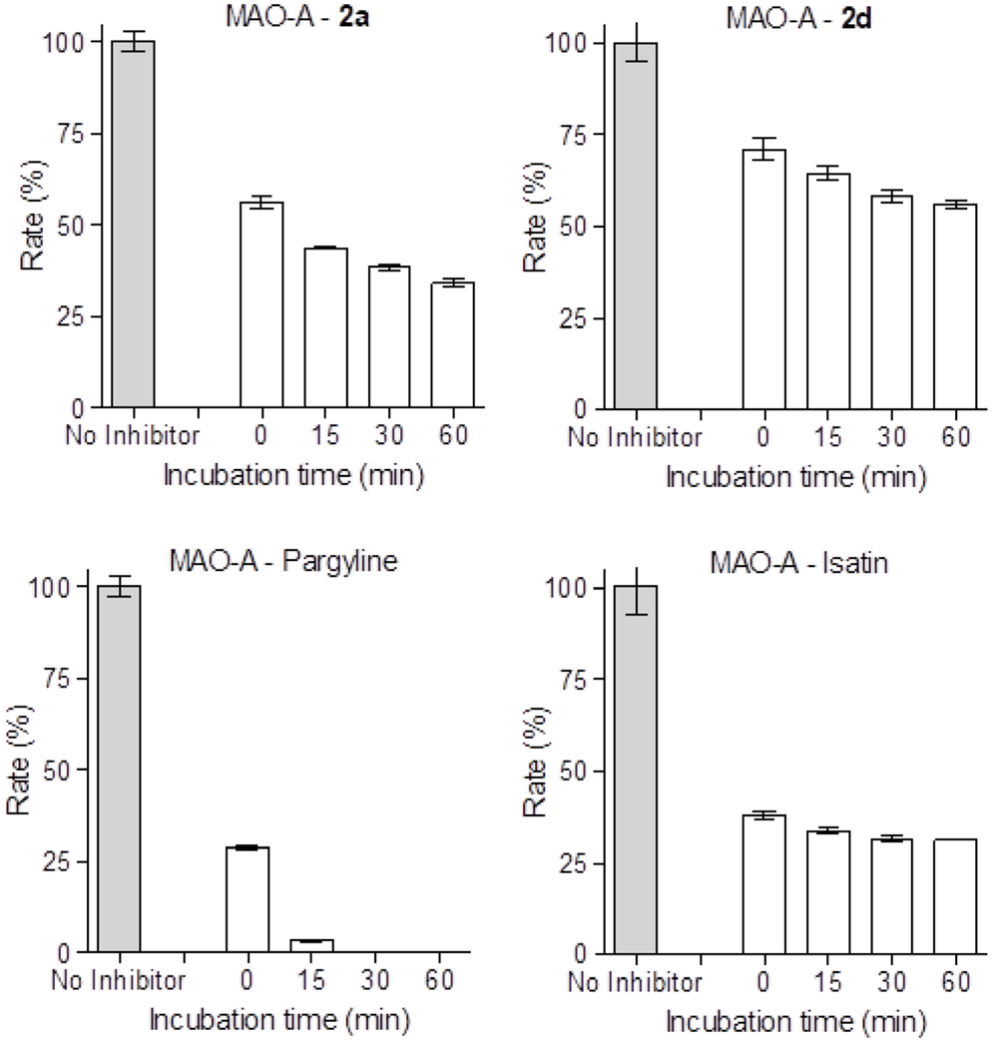
The time dependency of MAO‐A inhibition by **2a**, **2d**, isatin, and pargyline.

**FIGURE 7:**
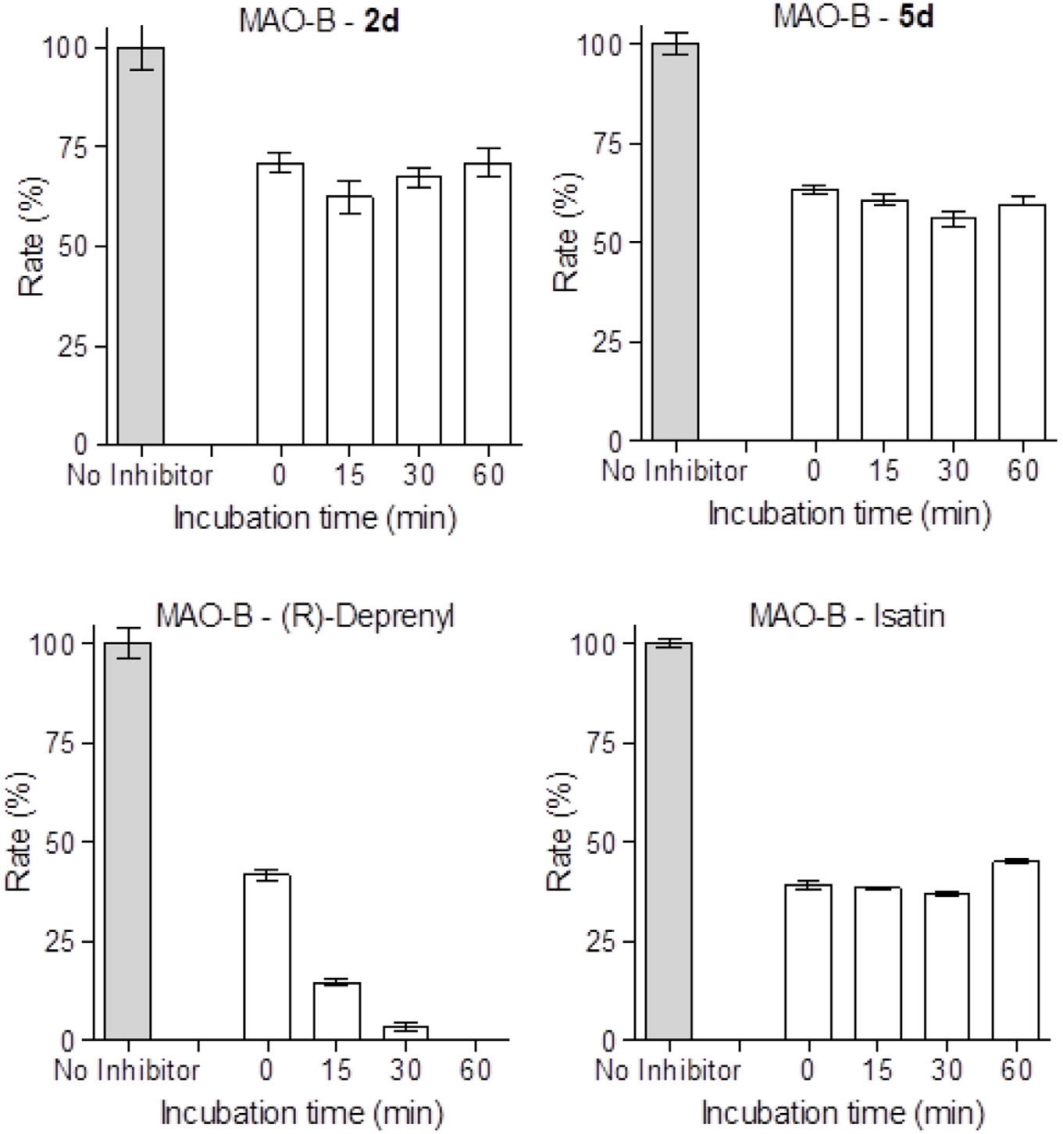
Time‐dependent inhibition of MAO‐B by **2d**, **5d**, isatin, and (R)‐deprenyl.

The time‐dependent inhibition studies showed that preincubation of **2a** and **2d** with MAO‐A (Figure 6) and **2d** and **5d** with MAO‐B (Figure 7) did not produce a progressive reduction in enzyme activity over 0–60 min. The residual activities remained close to the expected 50% for the test inhibitor (at a concentration that is equal to IC_50_), with only minor variations that did not increase with longer preincubation. This behavior was consistent with reversible inhibition. In contrast, the irreversible inhibitors, i.e., pargyline (MAO‐A) (Figure 6) and (R)‐deprenyl (MAO‐B) (Figure 7), displayed the characteristic potent, time‐dependent decline in activity, with residual rates falling below 50%. Together, these findings confirm that compounds **2a**, **2d**, and **5d** act as reversible inhibitors of MAO‐A or MAO‐B, respectively.

This finding correlates with previous research showing that both unsubstituted and halogen‐substituted isatins act as competitive and reversible inhibitors, and that their competitive inhibition is retained when conjugated with a Schiff base, such as benzoylhydrazine [16, 47]. Reversible inhibition is preferred because it decreases the chance of invoking a potentially fatal hypertensive crisis when consumed together with food rich in tyramine [51, 52].

### 2.3 In Vitro Antibacterial Minimum Inhibitory Concentration (MIC)

The results of the broth microdilution assay are shown in Table 3. When diluting the concentrated dimethyl sulfoxide (DMSO) stock solutions to working concentrations, most derivatives precipitated. None of the derivatives exhibited activity against the Gram‐negative bacteria, i.e., *K. aerogenes*, *K. pneumoniae*, and *P. aeruginosa*. Only one derivative, **5d**, had antibacterial activity with a minimum inhibitory concentration (MIC) of 16 µg/mL against *S. aureus*, the only Gram‐positive bacterium. The MIC, as measured with the colorimetric indicator (resazurin) of 8 µg/mL, corresponded with the turbidity reading. Aromatic substituents are known to increase lipophilicity, potentially lowering aqueous solubility [53]. The poor water solubility of the derivatives, largely due to the presence of aromatic rings, likely reduced their ability to exert in vitro antibacterial activity. Other research groups have synthesized isatin–Schiff base derivatives and found that they had antimicrobial activity [54, 56]. The poor aqueous solubility of the derivatives evaluated in this study might explain the discrepancy between our findings and the literature.

**TABLE 3:** MIC values of the target derivatives as determined by turbidity (OD_600_) and colorimetric (resazurin) measurements.

Derivatives	*S. aureus*		*K. aerogenes*		*K. pneumoniae*		*P. aeruginosa*	
	MIC, µg/mL OD_600_	MIC, µg/mL Resa.	MIC, µg/mL OD_600_	MIC, µg/mL Resa.	MIC, µg/mL OD_600_	MIC, µg/mL Resa.	MIC, µg/mL OD_600_	MIC, µg/mL Resa.
**1a**	>128	>128	>128	>128	>128	>128	>128	>128
**1b**	>128	>128	>128	>128	>128	>128	>128	>128
**1c**	>128	>128	>128	>128	>128	>128	>128	>128
**1d**	>128	>128	>128	>128	>128	>128	>128	>128
**1e**	>128	>128	>128	>128	>128	>128	>128	>128
**2a**	>128	>128	>128	>128	>128	>128	>128	>128
**2b**	>128	>128	>128	>128	>128	>128	>128	>128
**2c**	>128	>128	>128	>128	>128	>128	>128	>128
**2d**	>128	>128	>128	>128	>128	>128	>128	>128
**3a**	>128	>128	>128	>128	>128	>128	>128	>128
**3b**	>128	>128	>128	>128	>128	>128	>128	>128
**3c**	>128	>128	>128	>128	>128	>128	>128	>128
**4a**	>128	>128	>128	>128	>128	>128	>128	>128
**4b**	>128	>128	>128	>128	>128	>128	>128	>128
**5a**	>128	>128	>128	>128	>128	>128	>128	>128
**5b**	>128	>128	>128	>128	>128	>128	>128	>128
**5c**	>128	>128	>128	>128	>128	>128	>128	>128
**5d**	16	8	>128	>128	>128	>128	>128	>128
**5e**	>128	>128	>128	>128	>128	>128	>128	>128
**Tetracycline** a	0.125–0.25	0.25	2–1	4	2–1	4–2	8	16

*Note*: Resazurin (Reza.).

a Positive control.

### 2.4 In Vitro Urease Inhibition

None of the synthesized derivatives exhibited any inhibitory activity against Jack bean urease at the tested concentrations. This lack of activity might also be a result of low aqueous solubility of the derivatives and bulky aromatic substituents.

This is consistent with previous research showing that bulky substituents reduce urease inhibitory activity, whereas the absence of such groups facilitates easier access to the substrate‐binding pocket and minimizes unfavorable steric interactions [57].

### 2.5 Molecular Modeling

**FIGURE 8:**
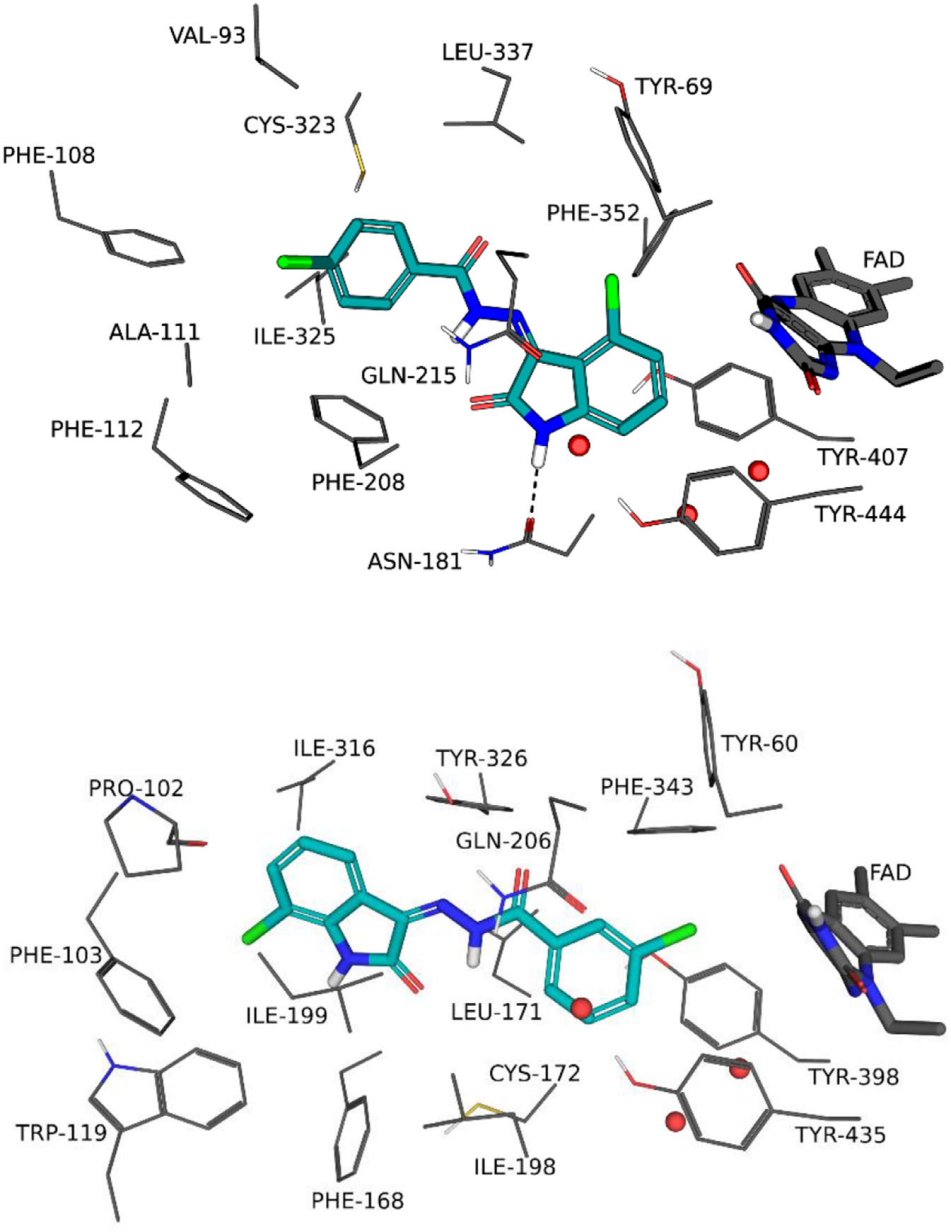
The docked orientations of **2d** (top) and **5d** (bottom) within the active sites of MAO‐A and MAO‐B, respectively. Hydrogen bonding interactions are represented by dashed lines.

Derivatives **2d** and **5d** were found to be the most potent inhibitors of MAO‐A and MAO‐B, respectively, and the molecular docking study thus focused on these two compounds. Their respective docking scores are shown in Table 4. Derivative **2d** was predicted to bind in the MAO‐A active site with the isatin moiety placed directly in front of the flavin while the chlorophenyl extended toward the entrance of the active site (Figure 8). A hydrogen bond formed with the side chain carbonyl of Asn‐181, while the isatin phenyl ring formed pi–pi interactions with Tyr‐407 and Tyr‐444. Within MAO‐B, a reversed binding mode was observed with the placement of the isatin moiety in the entrance cavity, the space beyond the gating residue, Ile‐199. Although no hydrogen binding interactions were observed, the chlorophenyl formed pi–pi interactions with Tyr‐398 and Tyr‐435 in front of the flavin. It should be noted that the inhibitors were relatively small compared to the active site cavities of the MAOs and thus did not completely fill the cavities. As a result, several orientations of the inhibitors were possible and only the highest ranked poses generated by CDOCKER were presented here.

**TABLE 4:** Docking scores of **2d** and **5d** docked into the active site of MAO‐A and MAO‐B, respectively.

Derivative	CDOCKER_ENERGY	CDOCKER_INTERACTION_ENERGY
**2d**	0.702	41.6
**5d**	21.2	46.9

## 3 Conclusion

In this study, five series of isatin–Schiff base derivatives were successfully synthesized, each with a hydroxy‐ and chloro‐substitution in different positions. The derivatives were first evaluated as potential inhibitors of MAO‐A and MAO‐B which are targets for the treatment of depression and PD, respectively. Thereafter, their anti‐infective activity was determined against a panel of bacteria as well as against the urease enzyme, a known virulence factor.

All derivatives had in vitro activity against both MAO‐A and MAO‐B, some with activity greater than the positive control. All series were more active against MAO‐B, except the 4‐chloro‐substituted isatin derivatives (series 2) which favored MAO‐A inhibition. This highlights the importance of the chloro‐substitution on isatin for selectively targeting the different MAO isoforms. With regard to MAO‐A inhibition, the 4‐chloro‐substituted isatin derivatives (series 2) were the most potent. The unsubstituted (series 1) and the 5‐chloro‐substituted derivatives (series 3) were moderately active, while the 6‐chloro‐substituted (series 4) and 7‐chloro‐substituted isatin (series 5) derivatives were less active. With regard to MAO‐B inhibition, series 3 was the most potent; however, series 1 and 5 also presented with good activity. Series 2 and 4 had potent inhibitors as well as some that were less active. Kinetic studies found that the mode of MAO inhibition was competitive and reversible.

Computational docking highlighted important binding interactions for each isoform. For MAO‐A, inhibitory potency was increased by a hydrogen bond with Asn‐181 and dual pi–pi stacking interactions with Tyr‐407 and Tyr‐444. In the case of MAO‐B, derivative **5d** bound in a reverse inhibitor orientation; while hydrogen bonding was absent, stability was maintained through pi–pi interactions involving Tyr‐398 and Tyr‐435.

With the exception of a single derivative that showed poor activity against *S. aureus*, none of the derivatives had any antibacterial activity against the four tested pathogens and likewise showed no inhibition of the urease enzyme. This general lack of activity may partially be attributed to poor aqueous solubility.

This study validates the potential of isatin–Schiff base derivatives as early‐stage leads for the treatment of neurodegenerative disorders like depression and PD. Compound **5d** was identified as a lead compound for further biological investigation. Future studies should synthesize derivatives with improved aqueous solubility while maintaining their neuroprotective potency.

## 4 Experimental Section

### 4.1 Synthesis of the Isatin–Schiff Base Derivatives

The derivatives were synthesized according to a synthetic route detailed in previous work [55]. To a solution of the appropriate isatin (Sigma‐Aldrich, South Africa) and hydrazine (AK Scientific, USA) in 30 mL of absolute ethanol, a catalytic amount of glacial acetic acid was added. The mixture was then heated under reflux (90 °C) for 6 h. Reaction progress was monitored by thin‐layer chromatography using silica gel 60 F_254_ plates (Merck, South Africa). Upon the complete consumption of the isatin starting material, the resulting precipitate was isolated via vacuum filtration and dried to constant weight in a desiccator. Most derivatives were purified by recrystallization using ethanol or a mixture of ethanol and acetonitrile as the solvent. Two derivatives (**1c** and **2d**) were purified by silica gel column chromatography, employing a mixture of methanol/dichloromethane (1:14, v/v) as eluent.

The aqueous solubility (*LogS*) of the synthesized derivatives was predicted using the SILICOS‐IT model implemented in the SwissADME web tool [58]. This model estimates the decimal logarithm of the molar solubility of compounds in water based on fragmental contributions. The SILICOS‐IT model also provides a solubility classification, where compounds are categorized as very soluble (*LogS* > −2), soluble (−2 ≥ *LogS* > −4), moderately soluble (−4 ≥ *LogS* > −6), poorly soluble (−6 ≥ *LogS* > −10), and insoluble (*LogS* ≤ −10).

Structural characterization of the synthesized derivatives was carried out by ^1^H NMR (600 MHz), ^13^C NMR (150 MHz), and HSQC spectroscopy. All spectra were recorded with a Bruker 600 MHz Avance NEO spectrometer (USA). The derivatives were dissolved in deuterated dimethyl sulfoxide (DMSO‐*d6*) (Merck, South Africa) for the NMR analysis. NMR data were processed using MestReNova software (version 14.1.2, Mestrelab Research, Spain) and were reported as chemical shifts (δ) in parts per million (ppm) and coupling constants (*J*) in hertz (Hz), alongside signal integration and multiplicity. The multiplicity was reported as singlet (s), doublet (d), doublet of doublets (dd), triplet (t) or multiplet (m). The melting point of each derivative was measured in triplicate with a Büchi B‐545 (Büchi, Switzerland) using the capillary method. HRMS was used to confirm the mass of each derivative and was performed using atmospheric pressure chemical ionization (APCI) on a Bruker microTOF‐Q II system (Bruker, USA).

The chemical purity of all target compounds was established through HPLC using a Shimadzu Nexera‐i LC‐2040C 3D Plus instrument with diode array detection. A Venusil XBP C18 column (2.1 × 150 mm, 5 µm, 100 Å) served as the stationary phase. The eluent was composed of a mixture of Milli‐Q water and acetonitrile (Merck, South Africa), each supplemented with 0.1% formic acid. Sample volume of 1–5 µL was injected and monitored at 250–350 nm. Analysis was conducted at a flow rate of 0.35 mL/min using a gradient starting at 60% acetonitrile. The organic phase was increased linearly to 100% over 5 min, returned to 40% at 5.1 min, and the column was re‐equilibrated for a total of 8 min.

#### 4.1.1 N′‐[(3Z)‐2‐Oxo‐1,2‐Dihydro‐3H‐Indol‐3‐Ylidene]benzohydrazide (1a)

The title compound was prepared from isatin (1.4 mmol) and benzoylhydrazine (1.4 mmol). Yield: 100 mg (27%) obtained as yellow solid, mp: 285.6 °C.

^1^H NMR (600 MHz, DMSO‐*d6*) *δ* 13.94 (s, 1H, NH‐9), 11.36 (s, 1H, NH‐12), 7.91 (d, *J* = 7.5 Hz, 2H, H‐16, H‐17), 7.69 (t, *J* = 7.4 Hz, 1H, H‐20), 7.64–7.50 (m, 3H, H‐3, H‐18, H‐19), 7.39 (t, *J* = 7.7 Hz, 1H, H‐6), 7.12 (t, *J* = 7.6 Hz, 1H, H‐5), 6.96 (d, *J* = 7.8 Hz, 1H, H‐4).

^13^C NMR (151 MHz, DMSO‐*d6*) *δ* 163.53 (C‐13), 142.95 (C‐2), 138.61 (C‐7), 133.30 (C‐20), 132.57 (C‐15), 132.26 (C‐6), 129.62 (C‐3), 127.89 (C‐16,17), 123.22 (C‐5), 121.43 (C‐18, 19), 120.30 (C‐1), 111.71 (C‐4). *Note:* The signal for C‐8 (a carbonyl carbon) was not observed.

APCI‐HRMS *m*/*z*: [M+H]^+^ calculated for C_15_H_12_N_3_O_2_ (MH^+^), 266.0924, found 266.0925. Purity (HPLC‐250 nm): 99.5%.

#### 4.1.2 3‐Hydroxy‐N′‐[(3Z)‐2‐Oxo‐1,2‐Dihydro‐3H‐Indol‐3‐Ylidene]benzohydrazide (1b)

The title compound was prepared from isatin (1.4 mmol) and 3‐hydroxybenzhydrazide (2.1 mmol). Yield: 106 mg (27.7%) obtained as orange solid, mp: 273 °C.

^1^H NMR (600 MHz, DMSO‐*d6*) *δ* 13.85 (s, 1H, NH‐9), 11.21 (s, 1H, NH‐12), 9.98 (s, 1H, OH‐21), 7.61 (d, *J* = 7.5 Hz, 1H, H‐17), 7.43–7.36 (m, 2H, H‐6, H‐19), 7.34–7.28 (m, 2H, H‐16, H‐20), 7.12 (t, *J* = 7.6 Hz, 1H, H‐5), 7.09–7.04 (m, 1H, H‐3), 6.97 (d, *J* = 7.8 Hz, 1H, H‐4).

^13^C NMR (151 MHz, DMSO‐*d6*) *δ* 163.53 (C‐13), 158.39 (C‐18), 142.94 (C‐2), 138.51 (C‐7), 133.89 (C‐15), 132.21 (C‐6), 130.74 (C‐19), 123.21 (C‐5), 121.39 (C‐17), 120.40 (C‐3), 120.37 (C‐1), 118.24 (C‐20), 114.65 (C‐16), 111.70 (C‐4). *Note:* The signal for C‐8 (a carbonyl carbon) was not observed.

APCI‐HRMS *m*/*z*: [M+H]^+^ calculated for C_15_H_12_N_3_O_3_ (MH^+^), 282.0873, found 282.0851. Purity (HPLC‐250 nm): 99.0%.

#### 4.1.3 4‐Hydroxy‐N′‐[(3Z)‐2‐Oxo‐1,2‐Dihydro‐3H‐Indol‐3‐Ylidene]benzohydrazide (1c)

The title compound was prepared from isatin (1.4 mmol) and 4‐hydroxybenzhydrazide (2.1 mmol). Yield: 76 mg (19.8%) obtained as yellow solid, mp: 288 °C.

^1^H NMR (600 MHz, DMSO‐*d6*) *δ* 13.85 (s, 1H, NH‐12), 10.85 (s, 2H, NH‐9, OH‐21), 7.81–7.75 (m, 2H, H‐16, H‐17), 7.60 (d, *J* = 7.2 Hz, 1H, H‐3), 7.38 (td, *J* = 7.7, 1.2 Hz, 1H, H‐6), 7.11 (td, *J* = 7.6, 0.9 Hz, 1H, H‐5), 7.00–6.94 (m, 3H, H‐4, H‐18, H‐19).

^13^C NMR (151 MHz, DMSO‐*d6*) *δ* 163.56 (C‐13), 162.44 (C‐20), 142.77 (C‐2), 137.66 (C‐7), 131.92 (C‐6), 130.11 (C‐16, C‐17), 123.13 (C‐15), 122.83 (C‐5), 121.20 (C‐3), 120.48 (C‐1), 116.32 (C‐18, C‐19), 111.68 (C‐4). *Note:* The signal for C‐8 (a carbonyl carbon) was not observed.

APCI‐HRMS *m*/*z*: [M+H]^+^ calculated for C_15_H_12_N_3_O_3_ (MH^+^), 282.0873, found 282.0885. Purity (HPLC‐348 nm): 100%.

#### 4.1.4 3‐Chloro‐N′‐[(3Z)‐2‐Oxo‐1,2‐Dihydro‐3H‐Indol‐3‐Ylidene]benzohydrazide (1d)

The title compound was prepared from isatin (1.4 mmol) and 3‐chlorobenzhydrazide (2.1 mmol). Yield: 244 mg (59.86%) obtained as orange solid, mp: 287 °C.

^1^H NMR (600 MHz, DMSO‐*d6*) *δ* 13.88 (s, 1H, NH‐12), 11.36 (s, 1H, NH‐9), 7.89 (t, *J* = 1.9 Hz, 1H, H‐16), 7.83 (dt, *J* = 7.7, 1.3 Hz, 1H, H‐17), 7.75 (dd, *J* = 7.9, 2.1 Hz, 1H, H‐3), 7.65 (t, *J* = 7.9 Hz, 1H, H‐19), 7.61–7.57 (m, 1H, H‐20), 7.40 (td, *J* = 7.7, 1.2 Hz, 1H, H‐6), 7.11 (t, *J* = 7.6 Hz, 1H, H‐5), 6.96 (d, *J* = 7.8 Hz, 1H, H‐4).

^13^C NMR (151 MHz, DMSO‐*d6*) *δ* 163.45 (C‐13), 143.07 (C‐2), 139.23 (C‐7), 134.68 (C‐15), 134.34 (C‐18), 133.04 (C‐3), 132.45 (C‐6), 131.59 (C‐19), 127.90 (C‐16), 126.42 (C‐17), 123.27 (C‐5), 121.51 (C‐20), 120.16 (C‐1), 111.76 (C‐4). *Note:* The signal for C‐8 (a carbonyl carbon) was not observed.

APCI‐HRMS *m*/*z*: [M+H]^+^ calculated for C_15_H_11_ClN_3_O_2_ (MH^+^), 300.0534, found 300.0514. Purity (HPLC‐331 nm): 94.8%.

#### 4.1.5 4‐Chloro‐N′‐[(3Z)‐2‐Oxo‐1,2‐Dihydro‐3H‐Indol‐3‐Ylidene]benzohydrazide (1e)

The title compound was prepared from isatin (1.4 mmol) and 4‐chlorobenzhydrazide (2.1 mmol). Yield: 149 mg (36.55%) obtained as yellow solid, mp: 297 °C.

^1^H NMR (600 MHz, DMSO‐*d6*) *δ* 13.88 (s, 1H, NH‐12), 11.36 (s, 1H, NH‐9), 7.95–7.88 (m, 2H, H‐16, H‐17), 7.72–7.66 (m, 2H, H‐18, H‐19), 7.61 (d, *J* = 7.5 Hz, 1H, H‐3), 7.41 (td, *J* = 7.7, 1.2 Hz, 1H, H‐6), 7.15–7.10 (m, 1H, H‐5), 6.97 (d, *J* = 7.8 Hz, 1H, H‐4).

^13^C NMR (151 MHz, DMSO‐*d6*) *δ* 163.49 (C‐13), 143.04 (C‐2), 138.14 (C‐20), 132.39 (C‐6), 131.39 (C‐15), 129.72 (C‐16, C‐17, C‐18, C‐19), 123.26 (C‐5), 121.50 (C‐3), 120.24 (C‐1), 111.75 (C‐4). *Note:* The signals for C‐7 and C‐8 (an imine and carbonyl carbon) were not observed.

APCI‐HRMS *m*/*z*: [M+H]^+^ calculated for C_15_H_11_ClN_3_O_2_ (MH^+^), 300.0534, found 300.0470. Purity (HPLC‐325 nm): 100%.

#### 4.1.6 N′‐[(3Z)‐4‐Chloro‐2‐Oxo‐1,2‐Dihydro‐3H‐Indol‐3‐Ylidene]benzohydrazide (2a)

The title compound was prepared from 4‐chloroisatin(1 mmol) and benzoylhydrazine (1.5 mmol). Yield: 84 mg (28%) obtained as deep yellow solid, mp: 243 °C.

^1^H NMR (600 MHz, DMSO‐*d6*) *δ* 14.03 (s, 1H, NH‐12), 11.54 (s, 1H, NH‐9), 7.93 (d, *J* = 7.4 Hz, 2H, H‐16, H‐17), 7.69 (t, *J* = 7.4 Hz, 1H, H‐20), 7.61 (t, *J* = 7.6 Hz, 2H, H‐18, H‐19), 7.37 (t, *J* = 8.0 Hz, 1H, H‐6), 7.13 (d, *J* = 8.2 Hz, 1H, H‐5), 6.93 (d, *J* = 7.8 Hz, 1H, H‐4).

^13^C NMR (151 MHz, DMSO‐*d6*) *δ* 163.01 (C‐13), 144.19 (C‐2), 133.25 (C‐20), 132.93 (C‐6), 132.51 (C‐3), 129.46 (C‐18, C‐19), 128.30 (C‐16, C‐17), 124.29 (C‐5), 117.27 (C‐1), 110.34 (C‐4). *Note:* The signals for C‐7 and C‐8 (an imine and carbonyl carbon) were not observed.

APCI‐HRMS *m*/*z*: [M+H]^+^ calculated for C_15_H_11_ClN_3_O_2_ (MH^+^), 300.0534, found 300.0485. Purity (HPLC‐250 nm): 98.2%.

#### 4.1.7 N′‐[(3Z)‐4‐Chloro‐2‐Oxo‐1,2‐Dihydro‐3H‐Indol‐3‐Ylidene]‐2‐Hydroxybenzohydrazide (2b)

The title compound was prepared from 4‐chloroisatin (1 mmol) and 2‐hydroxybenzhydrazide (1.5 mmol). Yield: 84 mg (26.6%) obtained as deep yellow solid, mp: 329 °C.

^1^H NMR (600 MHz, DMSO‐*d6*) *δ* 14.51 (s, 1H, NH‐12), 11.60 (s, 1H, NH‐9), 11.34 (s, 1H, OH‐22), 7.98 (d, *J* = 7.8 Hz, 1H, H‐17), 7.49–7.42 (m, 1H, H‐20), 7.35 (t, *J* = 8.0 Hz, 1H, H‐6), 7.11 (d, *J* = 8.2 Hz, 1H, H‐5), 7.07–6.95 (m, 2H, H‐18, H‐19), 6.90 (d, *J* = 7.8 Hz, 1H, H‐4).

^13^C NMR (151 MHz, DMSO‐*d6*) *δ* 161.58 (C‐13), 157.23 (C‐16), 144.16 (C‐2), 136.32 (C‐7), 134.73 (C‐20), 132.47 (C‐6), 131.81 (C‐17), 128.19 (C‐3), 124.07 (C‐5), 120.15 (C‐18), 118.00 (C‐1), 117.76 (C‐15), 117.33 (C‐19), 109.98 (C‐4). *Note:* The signal for C‐8 (a carbonyl carbon) was not observed.

APCI‐HRMS *m*/*z*: [M+H]^+^ calculated for C_15_H_11_ClN_3_O_3_ (MH^+^), 316.0483, found 316.0464. Purity (HPLC‐250 nm): 97.8%.

#### 4.1.8 N′‐[(3Z)‐4‐Chloro‐2‐Oxo‐1,2‐Dihydro‐3H‐Indol‐3‐Ylidene]‐3‐Hydroxybenzohydrazide (2c)

The title compound was prepared from 4‐chloroisatin (1 mmol) and 3‐hydroxybenzhydrazide (1.5 mmol). Yield: 95 mg (30%) obtained as deep yellow solid, mp: 332 °C.

^1^H NMR (600 MHz, DMSO‐*d6*) *δ* 14.02 (s, 1H, NH‐12), 11.52 (s, 1H, NH‐9), 9.95 (s, 1H, OH‐22), 7.43–7.31 (m, 4H, H‐6, 16, 17, 19), 7.13 (d, *J* = 8.2 Hz, 1H, H‐5), 7.09–7.04 (m, 1H, H‐20), 6.93 (d, *J* = 7.8 Hz, 1H, H‐4).

^13^C NMR (151 MHz, DMSO‐*d6*) δ 163.05 (C‐13), 158.33 (C‐18), 144.17(C‐2), 133.83 (C‐15), 132.89 (C‐6), 130.66 (C‐19), 128.32 (C‐3), 124.29 (C‐5), 120.40 (C‐20), 118.44 (C‐17), 117.31 (C‐1), 114.87 (C‐16), 110.33 (C‐4). *Note:* The signals for C‐7 and C‐8 (an imine and carbonyl carbon) were not observed.

APCI‐HRMS *m*/*z*: [M+H]^+^ calculated for C_15_H_11_ClN_3_O_3_ (MH^+^), 316.0483, found 316.0470. Purity (HPLC‐250 nm): 97.4%.

#### 4.1.9 4‐Chloro‐N′‐[(3Z)‐4‐Chloro‐2‐Oxo‐1,2‐Dihydro‐3H‐Indol‐3‐Ylidene]benzohydrazide (2d)

The title compound was prepared from 4‐chloroisatin (1 mmol) and 4‐chlorobenzhydrazide (1 mmol). Yield: 73 mg (21.8%) obtained as yellow solid, mp: 307 °C.

^1^H NMR (600 MHz, DMSO‐*d6*) *δ* 13.95 (s, 1H, NH‐12), 11.59 (s, 1H, NH‐9), 7.94 (d, *J* = 8.1 Hz, 2H, H‐16, H‐17), 7.68 (d, *J* = 8.2 Hz, 2H, H‐18, H‐19), 7.38 (t, *J* = 8.0 Hz, 1H, H‐6), 7.13 (d, *J* = 8.1 Hz, 1H, H‐5), 6.93 (d, *J* = 7.8 Hz, 1H, H‐4).

^13^C NMR (151 MHz, DMSO‐*d6*) *δ* 162.98 (C‐13), 144.32 (C‐2), 138.11 (C‐20), 133.04 (C‐6), 131.23 (C‐15), 129.52 (C‐16, 17, 18, 19), 128.32 (C‐3), 124.30 (C‐5), 117.19 (C‐1), 110.39 (C‐4). *Note:* The signals for C‐7 and C‐8 (an imine and carbonyl carbon) were not observed.

APCI‐HRMS *m*/*z*: [M+H]^+^ calculated for C_15_H_10_Cl_2_N_3_O_2_ (MH^+^), 334.0145, found 334.0140. Purity (HPLC‐250 nm): 94.6%.

#### 4.1.10 N′‐[(3Z)‐5‐Chloro‐2‐Oxo‐1,2‐Dihydro‐3H‐Indol‐3‐Ylidene]benzohydrazide (3a)

The title compound was prepared from 5‐chloroisatin (1 mmol) and benzoylhydrazine (1.5 mmol). Yield: 195 mg (65%) obtained as orange solid, mp: 315 °C.

^1^H NMR (600 MHz, DMSO‐*d6*) *δ* 13.84 (s, 1H, NH‐12), 11.46 (s, 1H, NH‐9), 7.91–7.90 (m, 2H, H‐16, 17), 7.71–7.69 (m, 1H, H‐20), 7.64–7.61 (m, 2H, H‐18, 19), 7.57 (d, *J* = 2.2 Hz, 1H, H‐3), 7.43 (dd, *J* = 2.2, 8.4 Hz, 1H, H‐6), 6.98 (d, *J* = 8.4 Hz, 1H, H‐4).

^13^C NMR (151 MHz, DMSO‐*d6*) *δ* 163.38 (C‐13), 141.70 (C‐2), 133.44 (C‐20), 132.41 (C‐15), 131.67 (C‐6), 129.66 (C‐18, 19), 127.98 (C‐16, 17), 127.38 (C‐5), 122.06 (C‐1), 120.93 (C‐3), 113.28 (C‐4). *Note:* The signals for C‐7 and C‐8 (an imine and carbonyl carbon) were not observed.

APCI‐HRMS *m*/*z*: [M+H]^+^ calculated for C_15_H_11_ClN_3_O_2_ (MH^+^), 300.0534, found 300.0527. Purity (HPLC‐250 nm): 95.3%.

#### 4.1.11 N′‐[(3Z)‐5‐Chloro‐2‐Oxo‐1,2‐Dihydro‐3H‐Indol‐3‐Ylidene]‐3‐Hydroxybenzohydrazide (3b)

The title compound was prepared from 5‐chloroisatin (1 mmol) and 3‐hydroxybenzohydrazide (1.5 mmol). Yield: 149 mg (47.2%) obtained as orange solid, mp: 303 °C.

^1^H NMR (600 MHz, DMSO‐*d6*) δ 13.80 (s, 1H, NH‐12), 11.44 (s, 1H, NH‐9), 9.96 (s, 1H, OH‐22), 7.56 (d, *J* = 2.2 Hz, 1H, H‐3), 7.44–7.35 (m, 2H, H‐6, 19), 7.34–7.28 (m, 2H, H‐16, 20), 7.10–7.05 (m, 1H, H‐17), 6.97 (d, *J* = 8.4 Hz, 1H, H‐4).

^13^C NMR (151 MHz, DMSO‐*d6*) δ 163.32 (C‐13), 158.40 (C‐18), 141.56 (C‐2), 137.55 (C‐7), 133.65 (C‐15), 131.58 (C‐6), 130.78 (C‐19), 127.38 (C‐5), 122.06 (C‐1), 120.88 (C‐3), 120.54 (C‐17), 118.30 (C‐16), 114.68 (C‐20), 113.22 (C‐4). *Note:* The signal for C‐8 (a carbonyl carbon) was not observed.

APCI‐HRMS *m*/*z*: [M+H]^+^ calculated for C_15_H_11_ClN_3_O_3_ (MH^+^), 316.0483, found 316.0500. Purity (HPLC‐250 nm): 91.4%.

#### 4.1.12 2‐Chloro‐N′‐[(3Z)‐5‐Chloro‐2‐Oxo‐1,2‐Dihydro‐3H‐Indol‐3‐Ylidene]benzohydrazide (3c)

The title compound was prepared from 5‐chloroisatin (1 mmol) and 2‐chlorobenzhydrazide (1.5 mmol). Yield: 138 mg (41%) obtained as orange solid, mp: 263 °C.

^1^H NMR (600 MHz, DMSO‐*d6*) *δ* 11.94 (s, 1H, NH‐12), 10.87 (s, 1H, NH‐9), 8.23 (s, 1H, H‐3), 7.71–7.32 (m, 5H, H‐6, 17, 18, 19, 20), 6.91 (d, *J* = 8.4 Hz, 1H, H‐4).

^13^C NMR (151 MHz, DMSO‐*d6*) *δ* 164.70 (C‐13), 143.21 (C‐2), 135.19 (C‐16), 132.65 (C‐6), 132.07 (C‐20), 130.75 (C‐15), 130.04 (C‐18), 127.67 (C‐19), 126.46 (C‐5), 126.17 (C‐3), 116.78 (C‐1), 112.45 (C‐4). *Note:* The signals for C‐7 and C‐8 (an imine and carbonyl carbon) were not observed.

APCI‐HRMS *m*/*z*: [M+H]^+^ calculated for C_15_H_10_Cl_2_N_3_O_2_ (MH^+^), 334.0144, found 334.0156. Purity (HPLC‐250 nm): 98.6%.

#### 4.1.13 N′‐[(3Z)‐6‐Chloro‐2‐Oxo‐1,2‐Dihydro‐3H‐Indol‐3‐Ylidene]benzohydrazide (4a)

The title compound was prepared from 6‐chloroisatin (1 mmol) and benzoylhydrazine (1.5 mmol). Yield: 229 mg (76.4%) obtained as yellow solid, mp: 340 °C.

^1^H NMR (600 MHz, DMSO‐*d6*) *δ* 13.83 (s, 1H, NH‐12), 11.48 (s, 1H, NH‐9), 7.91 (d, *J* = 7.1 Hz, 2H, H‐16, 17), 7.70 (t, *J* = 7.4 Hz, 1H, H‐20), 7.67–7.55 (m, 3H, 3, 18, 19), 7.17 (dd, *J* = 8.1, 1.8 Hz, 1H, H‐5), 7.00 (d, *J* = 1.8 Hz, 1H, H‐4).

^13^C NMR (151 MHz, DMSO‐*d6*) *δ* 163.58 (C‐13), 144.24 (C‐2), 141.95 (C‐7), 136.33 (C‐6), 133.40 (C‐20), 132.47 (C‐15), 129.66 (C‐3), 127.92 (C‐16,17), 123.11 (C‐5), 122.82 (C‐18, 19), 119.31 (C‐1), 111.81 (C‐4). *Note:* The signal for C‐8 (a carbonyl carbon) was not observed.

APCI‐HRMS *m*/*z*: [M+H]^+^ calculated for C_15_H_10_ClN_3_O_2_ (MH^+^), 300.0534, found 300.0538. Purity (HPLC‐250 nm): 98.4%.

#### 4.1.14 N′‐[(3Z)‐6‐Chloro‐2‐Oxo‐1,2‐Dihydro‐3H‐Indol‐3‐Ylidene]‐3‐Hydroxybenzohydrazide (4b)

The title compound was prepared from 6‐chloroisatin (1 mmol) and 3‐hydroxybenzhydrazide (1.5 mmol). Yield: 173 mg (54.8%) obtained as yellow solid, mp: 337 °C.

^1^H NMR (600 MHz, DMSO‐*d6*) *δ* 13.76 (s, 1H, NH‐12), 11.30 (s, 1H, NH‐9), 10.02 (s, 1H, OH‐22), 7.59 (d, *J* = 8.1 Hz, 1H, H‐3), 7.44–7.37 (m, 1H, H‐19), 7.33–7.27 (m, 2H, H‐16, 17), 7.14 (dd, *J* = 8.1, 1.9 Hz, 1H, H‐5), 7.09–7.04 (m, 1H, H‐20), 6.97 (d, *J* = 1.8 Hz, 1H, H‐4).

^13^C NMR (151 MHz, DMSO‐*d6*) *δ* 163.51 (C‐13), 158.39 (C‐18), 144.07 (C‐2), 137.54 (C‐7), 136.26 (C‐6), 133.73 (C‐15), 130.77 (C‐19), 123.09 (C‐5), 122.74 (C‐3), 120.47 (C‐20), 119.30 (C‐1), 118.24 (C‐17), 114.63 (C‐16), 111.76 (C‐4). *Note:* The signal for C‐8 (a carbonyl carbon) was not observed.

APCI‐HRMS *m*/*z*: [M+H]^+^ calculated for C_15_H_11_ClN_3_O_3_ (MH^+^), 316.0483, found 316.0474. Purity (HPLC‐342 nm): 100%.

#### 4.1.15 N′‐[(3Z)‐7‐Chloro‐2‐Oxo‐1,2‐Dihydro‐3H‐Indol‐3‐Ylidene]benzohydrazide (5a)

The title compound was prepared from 7‐chloroisatin (1 mmol) and benzoylhydrazine (1.5 mmol). Yield: 172 mg (57.38%) obtained as yellow solid, mp: 290 °C.

^1^H NMR (600 MHz, DMSO‐*d6*) δ 13.86 (s, 1H, NH‐12), 11.79 (s, 1H, NH‐9), 7.92 (dd, *J* = 7.2, 1.8 Hz, 2H, H‐16, 17), 7.78–7.53 (m, 4H, H‐3, 18, 19, 20), 7.48 (d, *J* = 8.1 Hz, 1H, H‐6), 7.15 (t, *J* = 7.8 Hz, 1H, H‐5).

^13^C NMR (151 MHz, DMSO‐*d6*) δ 163.63 (C‐13), 140.30 (C‐2), 133.46 (C‐15), 132.41 (C‐20), 131.75 (C‐6), 129.68 (C‐18, 19), 127.97 (C‐16, 17), 124.42 (C‐5), 122.37 (C‐1), 119.96 (C‐3), 115.84 (C‐4). *Note:* The signals for C‐7 and C‐8 (an imine and carbonyl carbon) were not observed.

APCI‐HRMS *m*/*z*: [M+H]^+^ calculated for C_15_H_10_ClN_3_O_2_ (MH^+^), 300.0534, found 300.0465. Purity (HPLC‐347 nm): 100%.

#### 4.1.16 N′‐[(3Z)‐7‐Chloro‐2‐Oxo‐1,2‐Dihydro‐3H‐Indol‐3‐Ylidene]‐3‐Hydroxybenzohydrazide (5b)

The title compound was prepared from 7‐chloroisatin (1 mmol) and 3‐hydroxybenzhydrazide (1.5 mmol). Yield: 131 mg (41.49%) obtained as yellow solid, mp: 302 °C.

^1^H NMR (600 MHz, DMSO‐*d6*) *δ* 13.80 (s, 1H, NH‐12), 11.76 (s, 1H, NH‐9), 10.00 (s, 1H, OH‐22), 7.57 (dd, *J* = 7.5, 1.0 Hz, 1H, H‐3), 7.51–7.37 (m, 2H, H‐6, 19), 7.37–7.25 (m, 2H, H‐16, 17), 7.15 (t, *J* = 8.0 Hz, 1H, H‐5), 7.08 (ddd, *J* = 8.1, 2.4, 1.0 Hz, 1H, H‐20).

^13^C NMR (151 MHz, DMSO‐*d6*) *δ* 163.60 (C‐13), 158.40 (C‐18), 140.22 (C‐2), 137.92 (C‐7), 133.67 (C‐15), 131.67 (C‐19), 130.82 (C‐6), 124.37 (C‐5), 122.38 (C‐1), 120.54 (C‐20), 119.90 (C‐3), 118.29 (C‐17), 115.82 (C‐4), 114.62 (C‐16). *Note:* The signal for C‐8 (a carbonyl carbon) was not observed.

APCI‐HRMS *m*/*z*: [M+H]^+^ calculated for C_15_H_11_ClN_3_O_3_ (MH^+^), 316.0483, found 316.0480. Purity (HPLC‐250 nm): 98.0%.

#### 4.1.17 2‐Chloro‐N′‐[(3Z)‐7‐Chloro‐2‐Oxo‐1,2‐Dihydro‐3H‐Indol‐3‐Ylidene]benzohydrazide (5c)

The title compound was prepared from 7‐chloroisatin (1 mmol) and 2‐chlorobenzhydrazide (1.5 mmol). Yield: 84 mg (25.13%) obtained as yellow solid, mp: 237 °C.

^1^H NMR (600 MHz, DMSO‐*d6*) *δ* 11.92 (s, 1H, NH‐12), 11.24 (s, 1H, NH‐9), 8.05 (s, 1H, H‐3), 7.77–7.35 (m, 5H, H‐6, 17, 18, 19, 20), 7.08 (t, *J* = 8.0 Hz, 1H, H‐5).

^13^C NMR (151 MHz, DMSO‐*d6*) *δ* 164.87 (C‐13) 141.86 (C‐2), 135.07 (C‐16), 132.85 (C‐15), 132.12 (C‐20), 130.79 (C‐18), 130.05 (C‐6, 17), 127.67 (C‐19), 125.43 (C‐3), 123.28 (C‐5), 117.40 (C‐1), 115.34 (C‐4). *Note:* The signals for C‐7 and C‐8 (an imine and carbonyl carbon) were not observed.

APCI‐HRMS *m*/*z*: [M+H]^+^ calculated for C_15_H_10_Cl_2_N_3_O_2_ (MH^+^), 334.0145, found 334.0159. Purity (HPLC‐345 nm): 90.0%.

#### 4.1.18 3‐Chloro‐N′‐[(3Z)‐7‐Chloro‐2‐Oxo‐1,2‐Dihydro‐3H‐Indol‐3‐Ylidene]benzohydrazide (5d)

The title compound was prepared from 7‐chloroisatin (1 mmol) and 3‐chlorobenzhydrazide (1 mmol). Yield: 148 mg (44%) obtained as yellow solid, mp: 288 °C.

^1^H NMR (600 MHz, DMSO‐*d6*) *δ* 11.92 (s, 1H, NH‐12), 11.23 (s, 1H, NH‐9), 8.05 (s, 1H, H‐3), 7.82–7.28 (m, 5H, H‐6, 16, 17, 19, 20), 7.08 (t, *J* = 7.9 Hz, 1H, H‐5).

^13^C NMR (151 MHz, DMSO‐*d6*) *δ* 164.84 (C‐13), 141.85 (C‐2), 135.10 (C‐15), 132.83 (C‐20), 132.07 (C‐19), 130.80 (C‐18), 130.04 (C‐6), 127.66 (C‐16, 17), 125.41 (C‐3), 123.28 (C‐5), 117.42 (C‐1), 115.34 (C‐4). *Note:* The signals for C‐7 and C‐8 (an imine and carbonyl carbon) were not observed.

APCI‐HRMS *m*/*z*: [M+H]^+^ calculated for C_15_H_10_Cl_2_N_3_O_2_ (MH^+^), 334.0144, found 334.0105. Purity (HPLC‐346 nm): 91.4%.

#### 4.1.19 4‐Chloro‐N′‐[(3Z)‐7‐Chloro‐2‐Oxo‐1,2‐Dihydro‐3H‐Indol‐3‐Ylidene] Benzohydrazide (5e)

The title compound was prepared from 7‐chloroisatin (1 mmol) and 4‐chlorobenzhydrazide (1.5 mmol). Yield: 90 mg (26.93%) obtained as yellow solid, mp: 305 °C.

^1^H NMR (600 MHz, DMSO‐*d6*) *δ* 13.80 (s, 1H, NH‐12), 11.80 (s, 1H, NH‐9), 7.97–7.83 (m, 2H, H‐16, 17), 7.79–7.63 (m, 2H, H‐18, 19), 7.58 (d, *J* = 7.5 Hz, 1H, H‐3), 7.48 (dd, *J* = 8.2, 1.0 Hz, 1H, H‐6), 7.14 (t, *J* = 7.8 Hz, 1H, H‐5).

^13^C NMR (151 MHz, DMSO‐*d6*) *δ* 163.55 (C‐13), 140.33 (C‐2), 138.29 (C‐20), 131.85 (C‐6), 131.19 (C‐15), 129.96 (C‐16, 17), 129.77 (C‐18, 19), 124.45 (C‐5), 122.27 (C‐1), 120.02 (C‐3), 115.85 (C‐4). *Note:* The signals for C‐7 and C‐8 (an imine and carbonyl carbon) were not observed.

APCI‐HRMS *m*/*z*: [M+H]^+^ calculated for C_15_H_9_Cl_2_N_3_O_2_ (MH^+^), 334.0145, found 334.0093. Purity (HPLC‐250 nm): 97.0%.

### 4.2 In Vitro Monoamine Oxidase (MAO) Inhibition

The in vitro inhibitory potency for each derivative was determined against recombinant human MAO‐A and MAO‐B using a method previously described by Mostert and coworkers [59]. The enzyme reactions were prepared in 96‐well microtiter plates in potassium phosphate (K_2_HPO_4_) as reaction buffer. The reactions contained substrate (kynuramine) and the test derivative (0.003–100 µM) and were initiated with the addition of either MAO‐A or MAO‐B. Stock solutions of the test derivative were dissolved in DMSO. The reactions were allowed to proceed for 20 min, after which sodium hydroxide (NaOH) was added to stop the reactions. MAO‐A and MAO‐B catalyze the conversion of kynuramine into 4‐hydroxyquinoline, which is a fluorescent metabolite that can be measured by fluorescence spectrophotometry. The IC_50_ values were derived from sigmoidal plots of enzyme activity versus log [I] and were expressed as the mean ± standard deviation of experiments that were conducted in triplicate. Data processing and graphical representation were carried out using GraphPad Prism version 5.0 (GraphPad Software, San Diego, CA, USA).

The mode of inhibition was determined by creating Lineweaver–Burk graphs for derivatives **2a** and **2d** (MAO‐A) as well as **2d** and **5d** (MAO‐B). Enzyme activities were determined in the presence and absence of inhibitors at various IC_50_ multiples (¼ × IC_50_, ½ × IC_50_, ¾ × IC_50_, 1 × IC_50_, and 1¼ × IC_50_). Catalytic rates were evaluated using kynuramine concentrations that ranged between 15 and 250 µM [59, 60].

The protocol to determine time dependency of inhibition has been described previously [61] Derivatives **2a** and **2d** were evaluated as time‐dependent inhibitors of MAO‐A, and derivatives **2d** and **5d** as time‐dependent inhibitors of MAO‐B. Recombinant human MAO‐A or MAO‐B was preincubated with the test derivatives (at a concentration of 2 x IC_50_) for 0, 15, 30, and 60 min at 37 °C in 100 mM potassium phosphate buffer (pH 7.4). After preincubation, kynuramine (50 μM final concentration) was added (yielding an inhibitor concentration of 1 × IC_50_) and the reactions were incubated for a further 20 min. The reactions were terminated, and the residual rates of 4‐hydroxyquinoline formation were determined as described above for the determination of IC_50_ values.

### 4.3 In Vitro Antibacterial Minimum Inhibitory Concentration (MIC)

The MIC was determined using the broth microdilution method [62, 63], against *S. aureus* (ATCC 29213), *K. pneumoniae* (ATCC 13883), *P. aeruginosa* (ATCC 27853), and *K. aerogenes* (ATCC 13048).

Sterilization of equipment and culture media was performed at 121 °C for 30 min using an Equitron Medica autoclave (Equitron Medica Ltd., United Scientific, South Africa). Each inoculum was created by incubating a bacterium in Mueller–Hinton Broth (MHB) at 35 ± 2 °C while shaking the suspension (100 rpm) (Labotec, South Africa). To ensure no change in bacterial concentration, the inoculum (1 × 10^6^ CFU/mL) was used within 30 min after being prepared. The derivatives were filter sterilized (0.2 µm; Lasec, South Africa) and tested in triplicate against all bacterial strains at concentrations ranging from 4 to 128 µg/mL. Sterile MHB was used as the growth control and tetracycline (Sigma‐Aldrich, South Africa) as the positive control. The experiments were conducted in flat‐bottom 96‐well plates (Lasec, South Africa), and all serial dilutions were created using multichannel pipettes. The inoculum (100 µL) was added to each well resulting in a final volume of 200 µL and a bacterial density of 5 x 10^5^ CFU/mL. The inoculum was, however, not added to the sterility control.

To verify the concentration of the initial inoculum, a viability count was performed. A 10‐µL aliquot was withdrawn from the growth control and diluted into 10 mL of MHB. Following thorough mixing, 100 µL of this suspension was spread across three Mueller–Hinton Agar plates. Both the agar plates and the 96‐well microplates were subsequently incubated for 20–24 h at 35 ± 2 °C [64]. The MIC is defined as the lowest concentration where no bacterial growth was observed. Bacterial growth was measured with turbidity readings measured at 600 nm (OD_600_) as well as using the colorimetric indicator, resazurin (150 µg/mL) (Merck, South Africa). The OD_600_ was measured before (*t*_0_) and after (*t*_20_) incubation, and growth less than 20% compared to the growth control was considered no growth. Resazurin (60 µL) was added to each well after incubation (*t*_20_) and was reincubated at 35 ± 2 °C for 4 h [65]. A color change from blue (resazurin) to pink (resorufin) was assessed visually and indicated bacterial growth [65, 67].

### 4.4 In Vitro Urease Inhibition

The assay has been published by Tanaka and coworkers [68]. The urease enzyme reactions were carried out in 96‐well microtiter plate, where potassium phosphate buffer (15 µL, KH_2_PO_4_), Jack bean urease (25 µL, 40 units/mL), and the test derivatives (10 µL) were incubated for 30 min. Stock solutions of the test derivatives were prepared in DMSO. A volume of 200 µL solution of the substrate, urea (500 mM), and phenol red (0.002%) was added to the reactions and the absorbance was continuously measured at 570 nm for 90 min. An increase in absorbance was due to an increase in pH resulting from the formation of ammonium in the urease catalyzed reaction. The final concentration of the enzyme in these reactions was 4 units/mL, and the inhibitor concentrations ranged from 0.003 to 100 µM. The IC_50_ values were determined as described above for MAO.

### 4.5 Computer Modeling

The binding modes of the isatin‐based compounds to MAO‐A and MAO‐B were investigated with docking studies. The structures of the human MAO‐A and MAO‐B proteins were obtained from PDB entries 2Z5X and 2V5Z, respectively [69, 70], and served as the structure models for this investigation. The docking process was performed by Discovery Studio 3.1, following a validated protocol described in the literature [59]. For the final analysis and presentation of molecular interactions, PyMOL was employed [71].

## Author Contributions

**Marthinus J. Jooste**: investigation, formal analysis, writing – original draft, visualization. **Anél Petzer**: conceptualization, methodology, validation, formal analysis, resources, writing – review and editing, supervision. **Jacobus P. Petzer**: conceptualization, methodology, validation, formal analysis, resources, writing – review and editing, supervision. **Stephanus J. Cloete**: formal analysis, writing – review and editing. **Theunis T. Cloete**: conceptualization, methodology, validation, formal analysis, investigation, resources, data curation, writing – original draft, visualization, supervision, project administration, funding acquisition.

## Funding

This research did not receive any specific grant from funding agencies in the public, commercial, or not‐for‐profit sectors.

## Conflicts of Interest

The authors declare no conflicts of interest.

## Supporting information

Supplementary Material

## Data Availability

Data will be made available on request.
