# Supplementary material for: Evaluation of Novel Isatin–Schiff Base Derivatives: Monoamine Oxidase Inhibition and Antimicrobial Assessment
Source: ChemMedChem. 2026 Aug 15;21(16):e70437. doi: 10.1002/cmdc.70437 (PMC13477431; doi:10.1002/cmdc.70437)

Evaluation of novel isatin–Schiff base derivatives: Potent monoamine oxidase inhibition and antimicrobial assessment

Marthinus J. Jooste[1], Stephanus J. Cloete[1], Anél Petzer[1,2], Jacobus P. Petzer[1,2], Theunis T. Cloete[1,2]*

[1]Centre of Excellence for Pharmaceutical Sciences, North-West University, Private Bag X6001, Potchefstroom, 2520, South Africa

[2]Pharmaceutical Chemistry, School of Pharmacy, North-West University, Private Bag X6001, Potchefstroom, 2520, South Africa

First-author: Mr. Marthinus J. Jooste; Tel.: +27828151430; Email address: 34309233@mynwu.ac.za; ORCID: 0009-0005-7633-8707

Co-author: Dr. Stephanus J. Cloete; Tel.: +27721439868; Email address: 23496959@mynwu.ac.za; ORCID: 0000-0002-3770-8403

Co-author: Prof. Anél Petzer; Tel.: +2718 299 4464; Email address: anel.petzer@nwu.ac.za; ORCID: 0000-0001-8114-8223

Co-author: Prof. Jacobus P. Petzer; Tel.: +2718 299 2206; Email address: jacques.petzer@nwu.ac.za; ORCID: 0000-0002-7114-8120

*Corresponding author: Prof. Theunis T. Cloete; Tel.: +2718 299 1714; Email address: theunis.cloete@nwu.ac.za; ORCID: 0000-0003-1831-4078

Description

This Supplementary Material document contains the comprehensive analytical data supporting the structural characterisation and purity assessment of the five novel series of isatin–Schiff base derivatives (**1a–5e**) synthesised and evaluated in this study.

For each synthesised derivative, the fully resolved proton (^{1}H) NMR (600MHz), carbon (^{13}C) NMR (150MHz), and heteronuclear single quantum correlation (HSQC) spectra are provided. Spectra were recorded in deuterated dimethyl sulfoxide (DMSOd).

An additional heteronuclear multiple bond correlation (HMBC) spectrum is provided for derivative **1a**. This specific analysis was conducted to unequivocally assign the quaternary carbon signals, successfully confirming that the observed carbonyl signal corresponds to *C-13* while the *C-8* signal remained unresolved.

Structural confirmation was further validated through high-resolution mass spectrometry (HRMS) using atmospheric pressure chemical ionisation (APCI). Each experimental HRMS spectrum is presented alongside the respective chemical structure and the calculated theoretical molecular mass (*m/z*) to facilitate direct verification of the target derivatives.

The chemical purity of each derivative was established through high-performance liquid chromatography (HPLC) using a diode-array detector. The corresponding chromatograms are included to confirm that all compounds achieved a purity level between 90 and 100%.

Nuclear Magnetic Resonance (NMR) Spectra

N'-[(3Z)-2-oxo-1,2-dihydro-3H-indol-3-ylidene]benzohydrazide (**1a**)

Proton (^{1}H) NMR spectrum

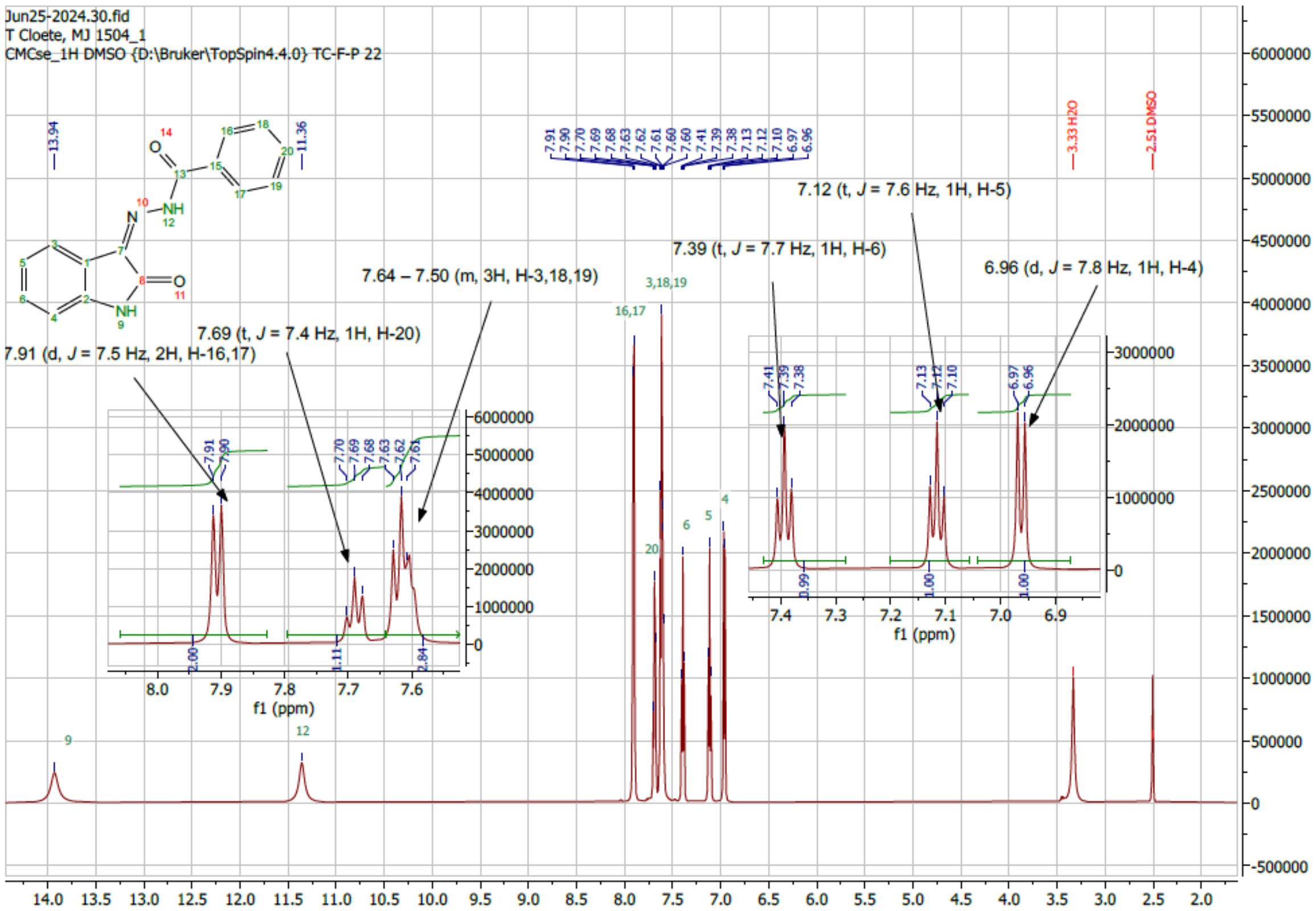

Carbon (^{13}C) NMR spectrum

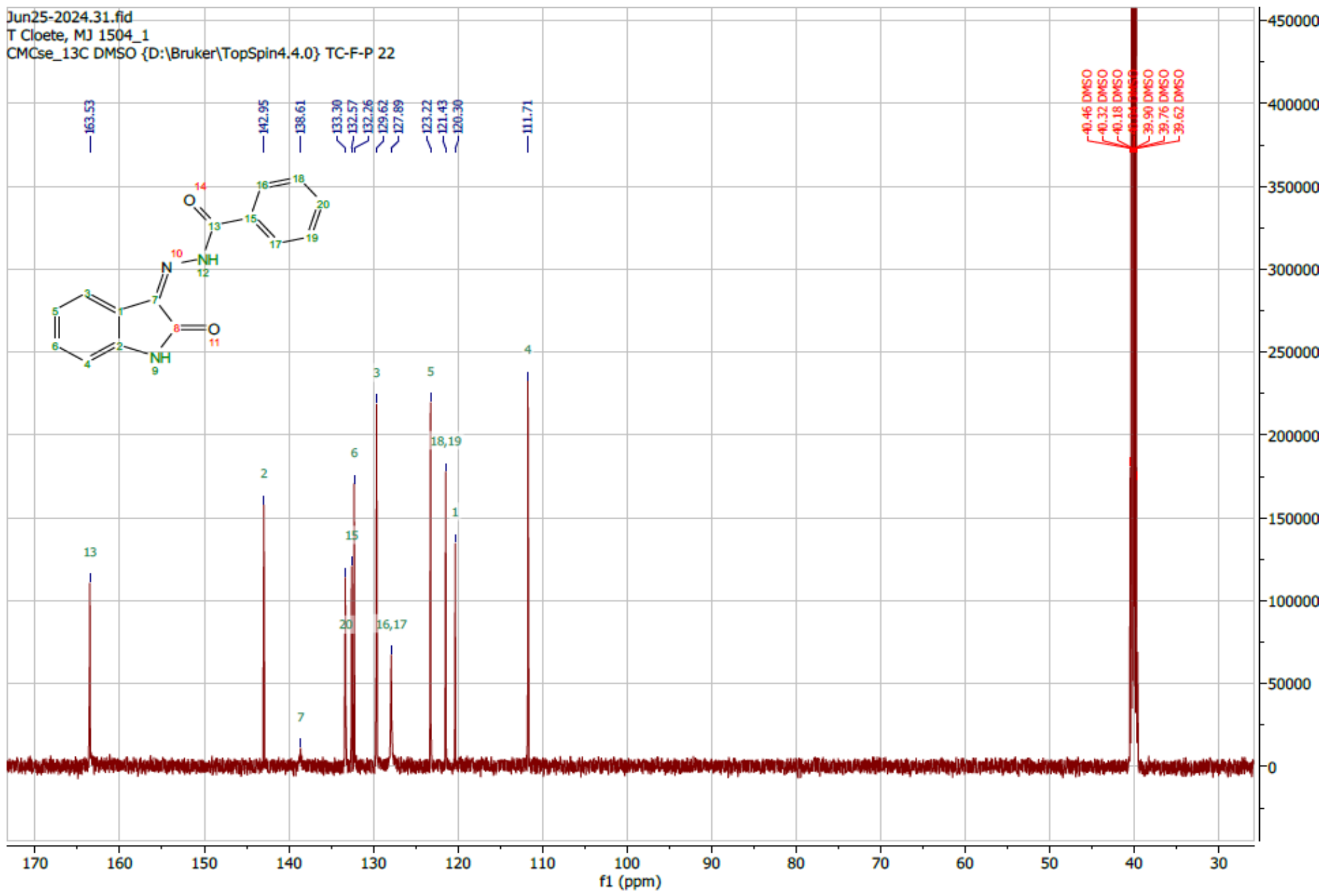

Heteronuclear single-quantum correlation (HSQC) NMR spectrum

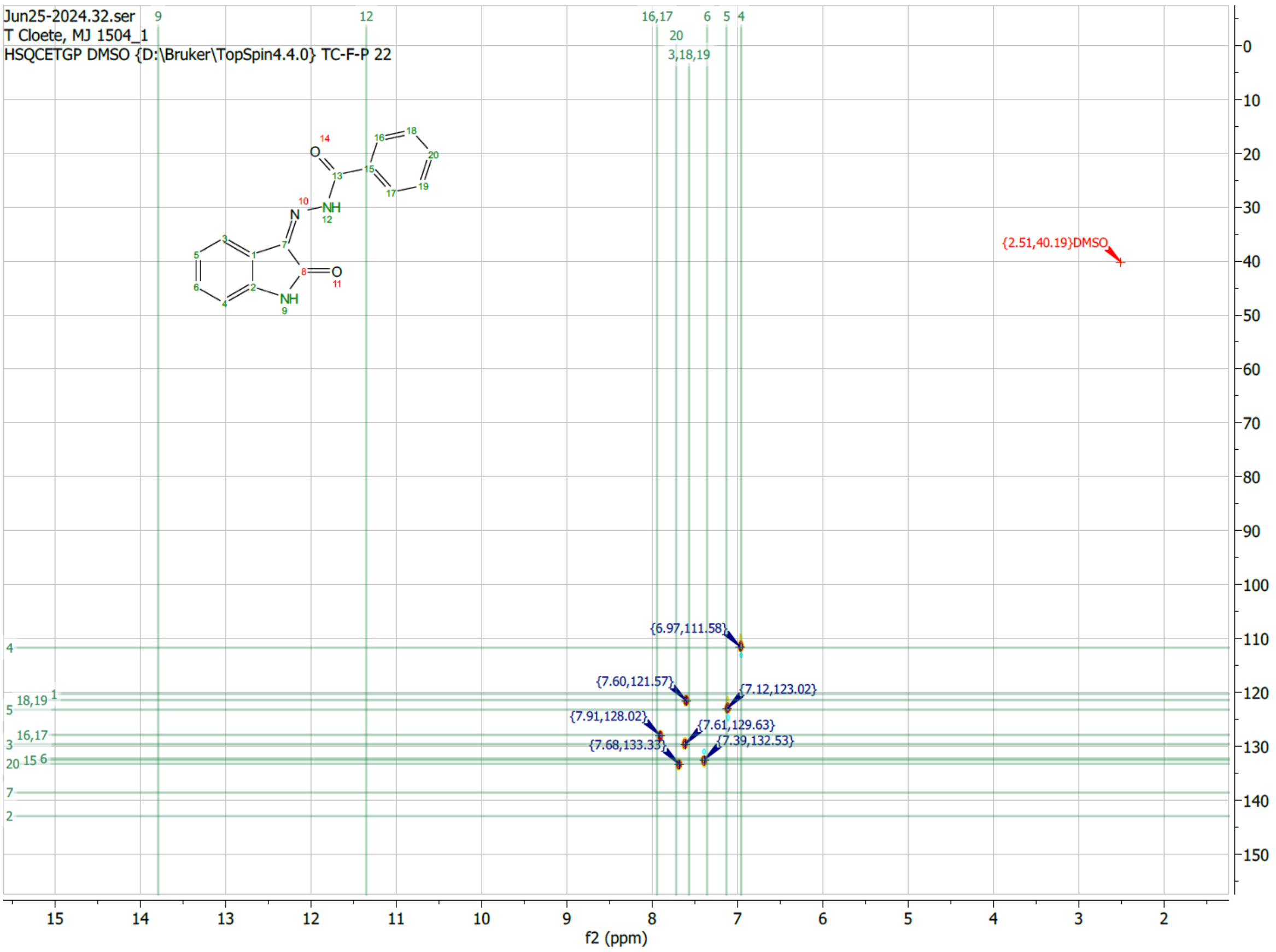

Heteronuclear multiple bond coherence (HMBC) NMR spectrum

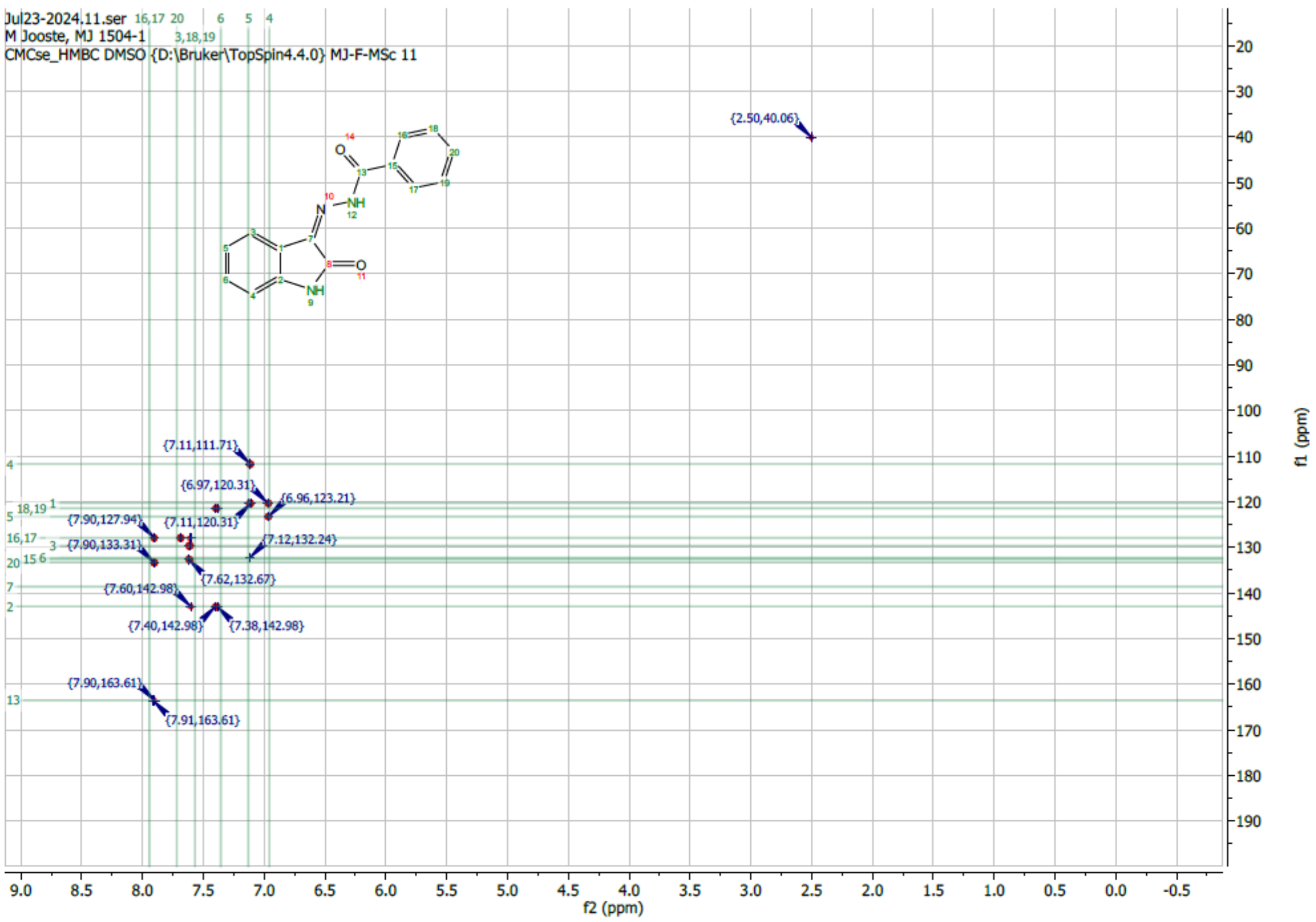

Correlation spectroscopy (COSY) NMR spectrum

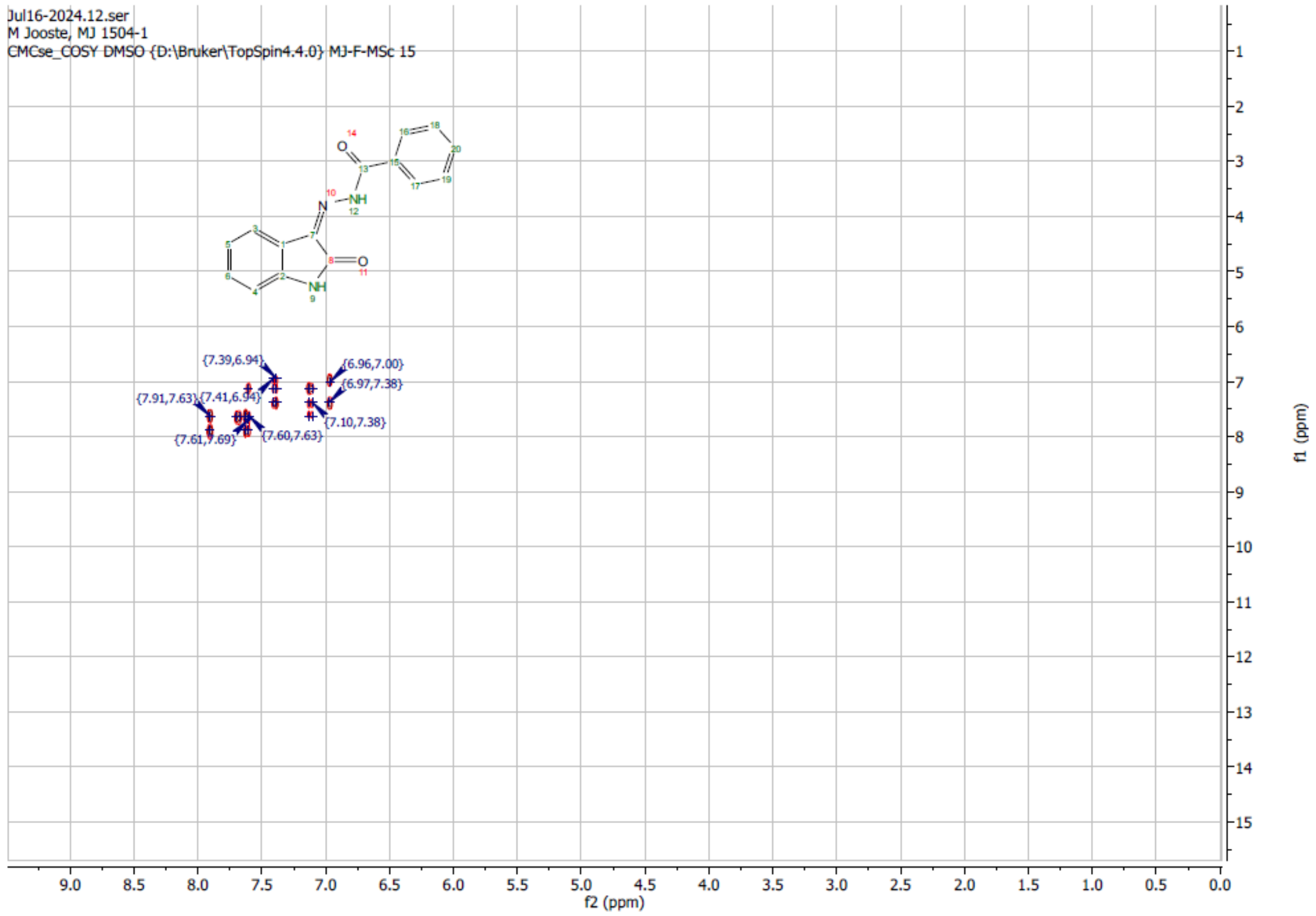

Distortion less enhancement by polarization transfer (DEPT) 135 NMR spectrum

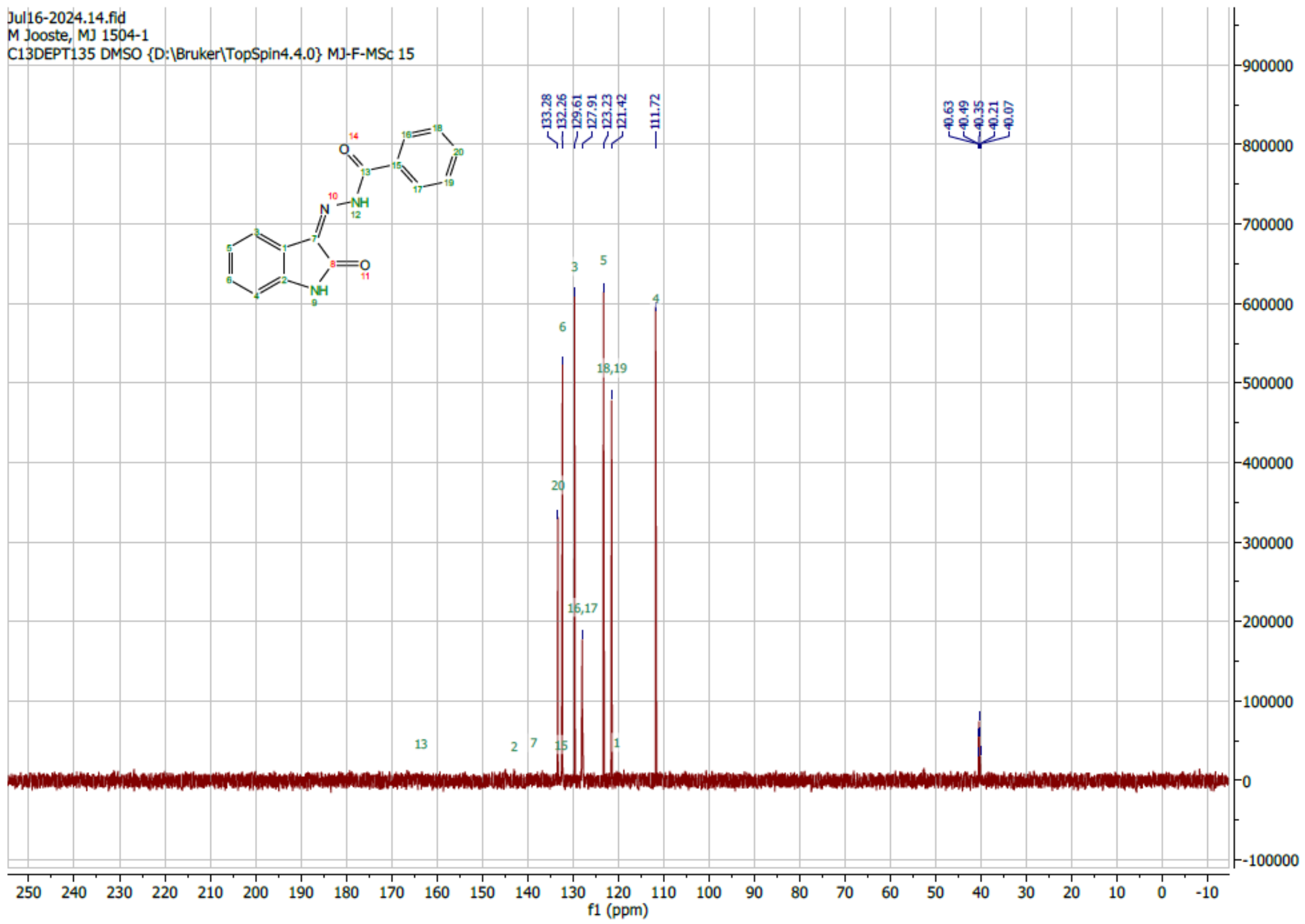

*3-hydroxy-N'-[(3Z)-2-oxo-1,2-dihydro-3H-indol-3-ylidene]benzohydrazide (**1b**)*

Proton (^{1}H) NMR spectrum

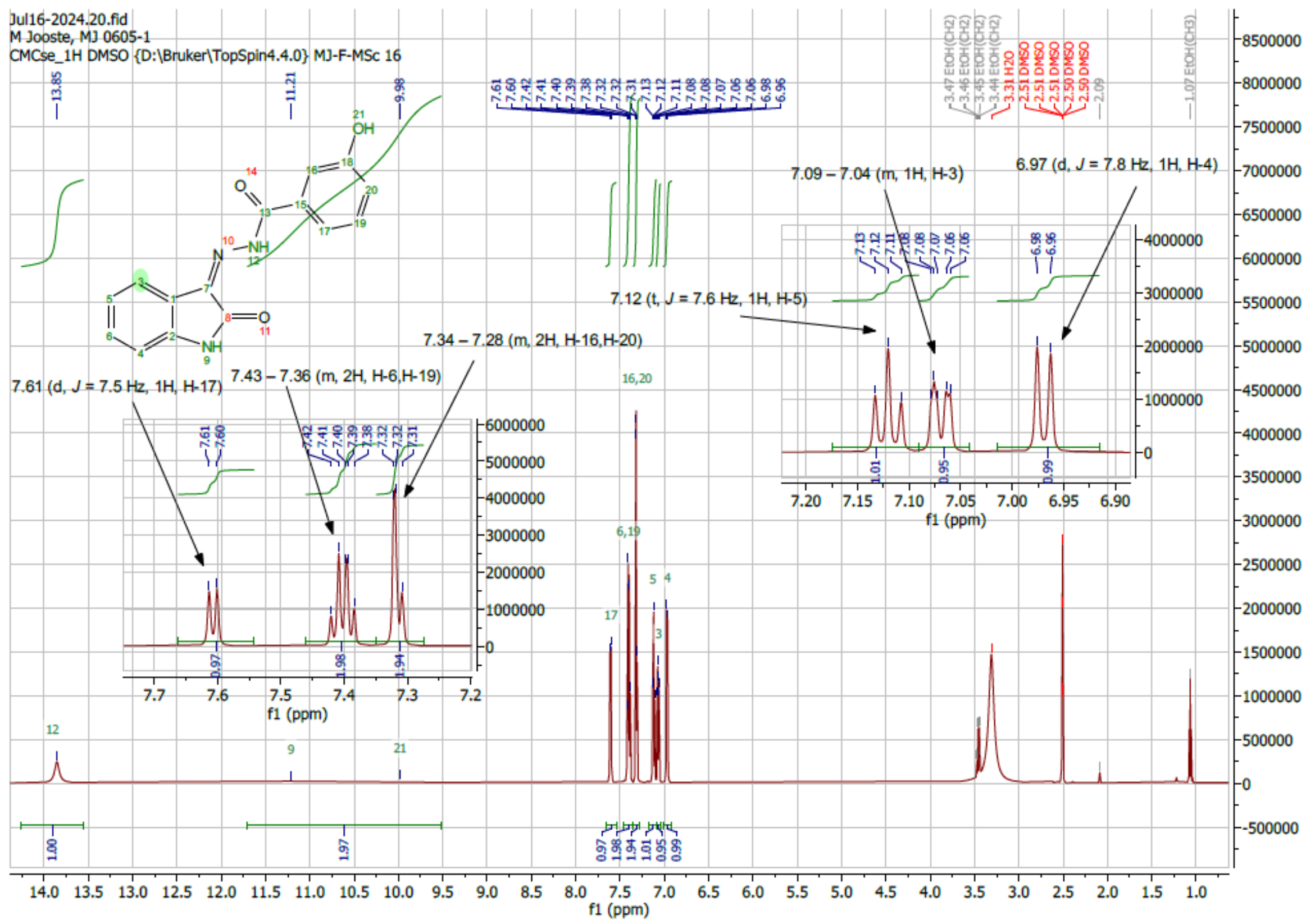

Carbon (^{13}C) NMR spectrum

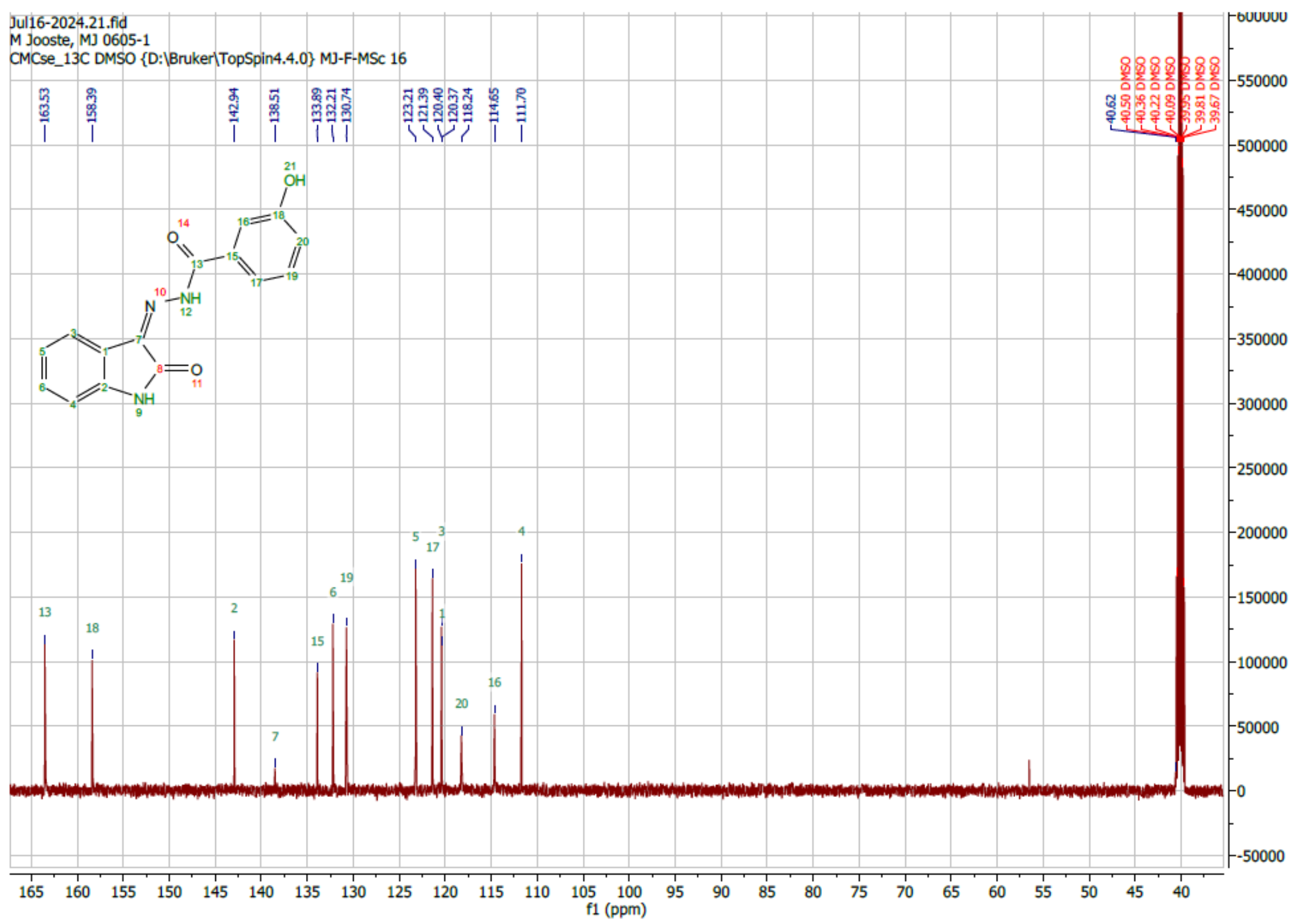

HSQC NMR spectrum

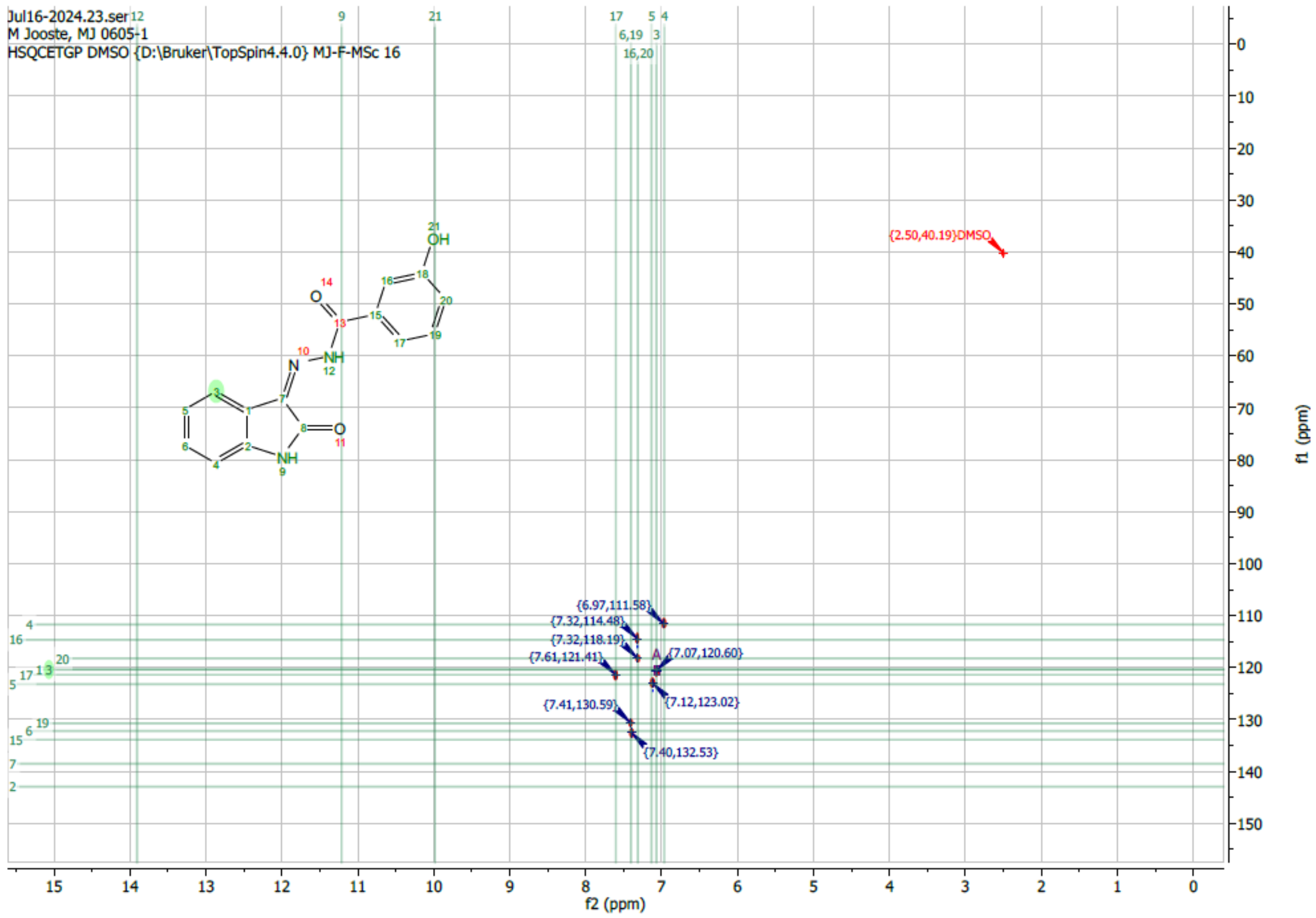

*4-hydroxy-N'-[(3Z)-2-oxo-1,2-dihydro-3H-indol-3-ylidene]benzohydrazide (**1c**)*

Proton (^{1}H) NMR spectrum

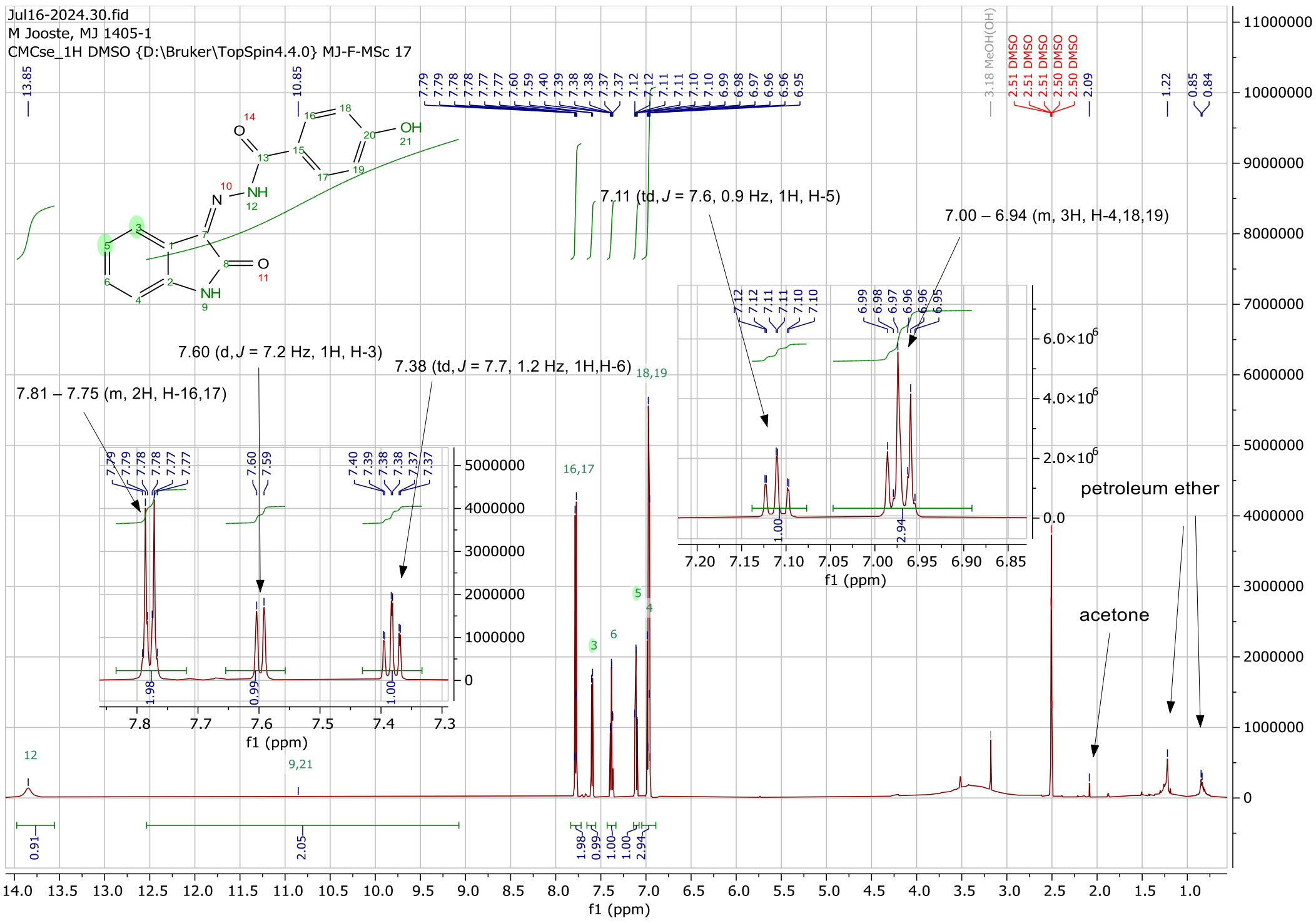

Carbon (^{13}C) NMR spectrum

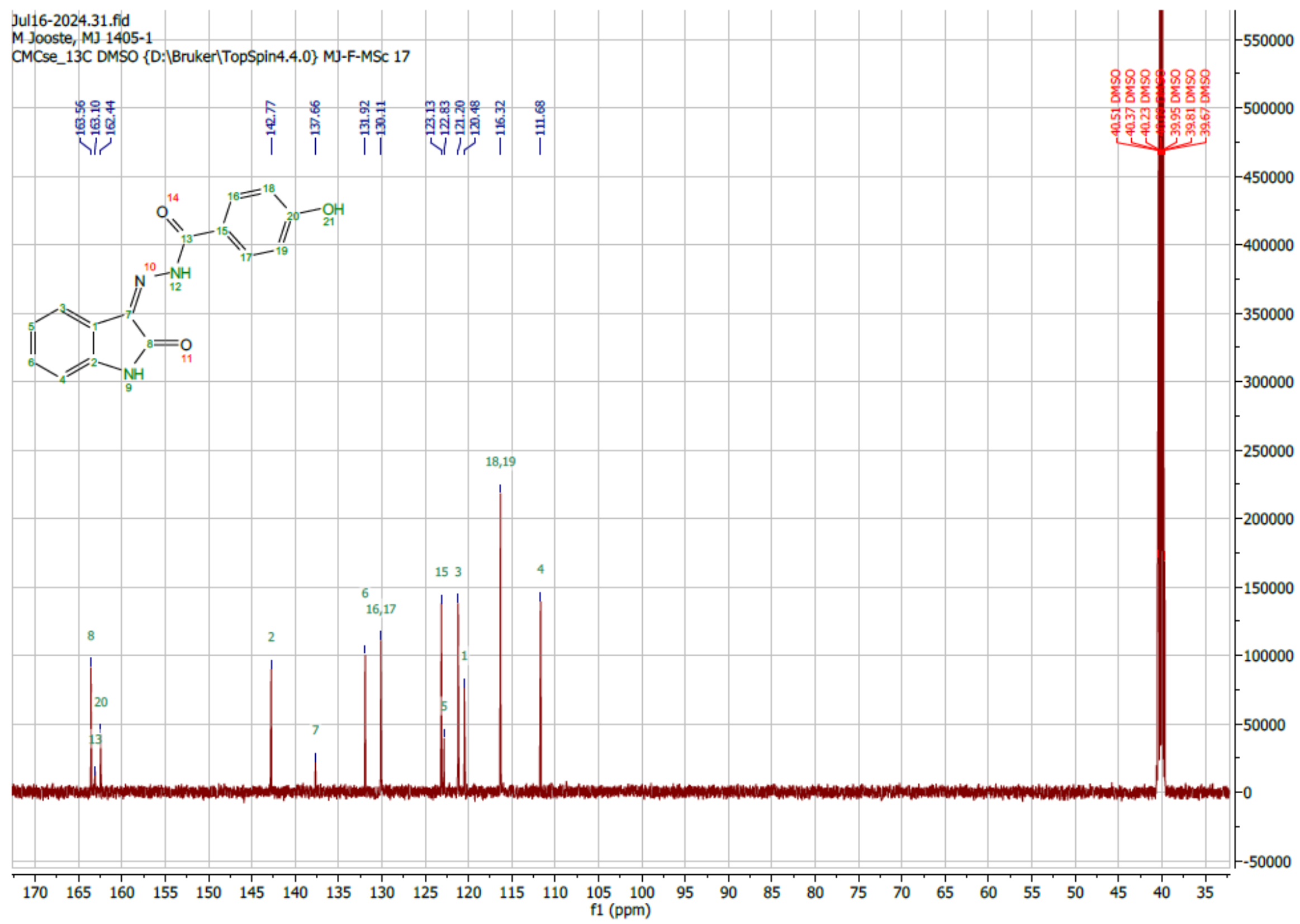

HSQC NMR spectrum

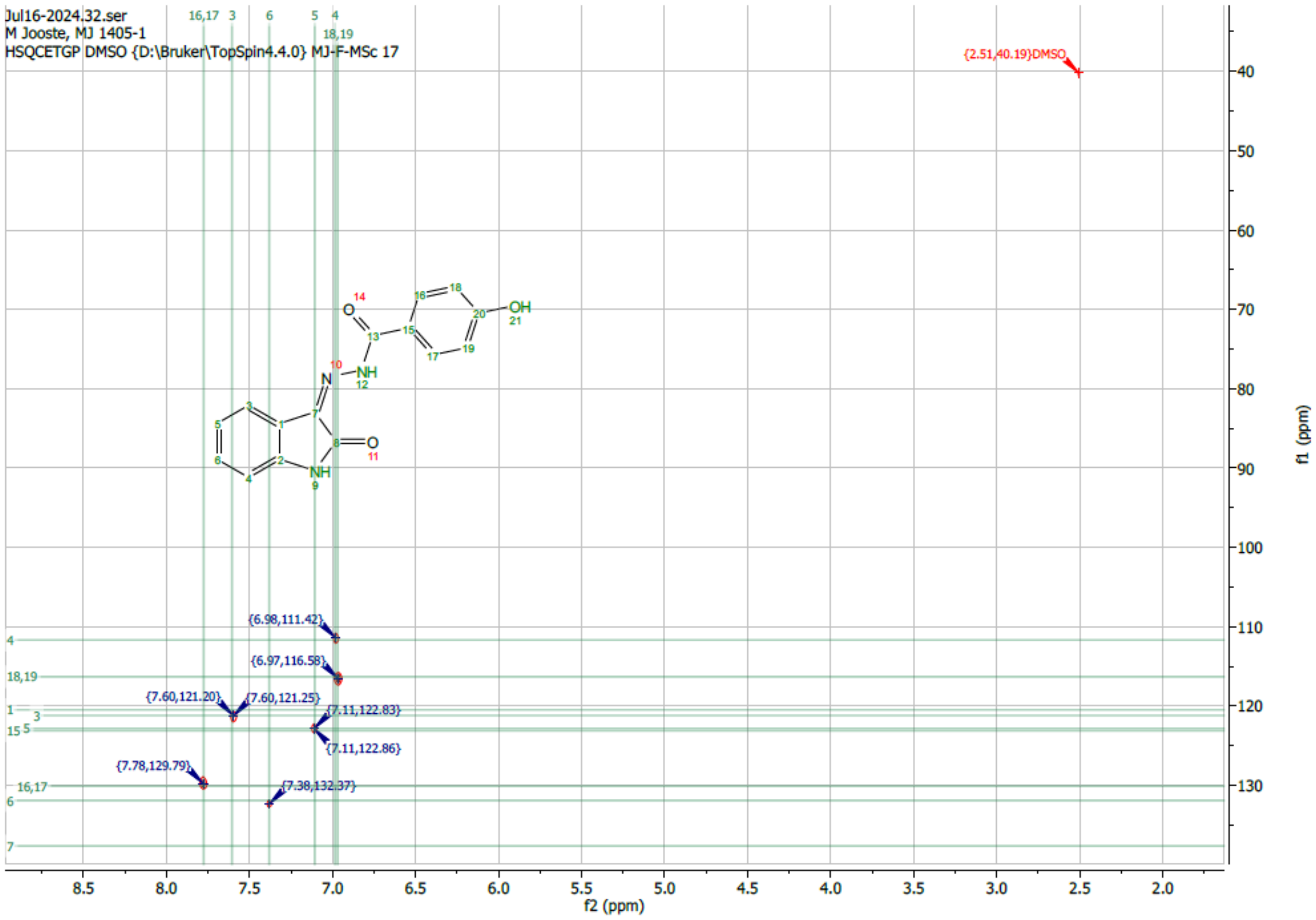

*3-chloro-N'-[(3Z)-2-oxo-1,2-dihydro-3H-indol-3-ylidene]benzohydrazide (**1d**)*

Proton (^{1}H) NMR spectrum

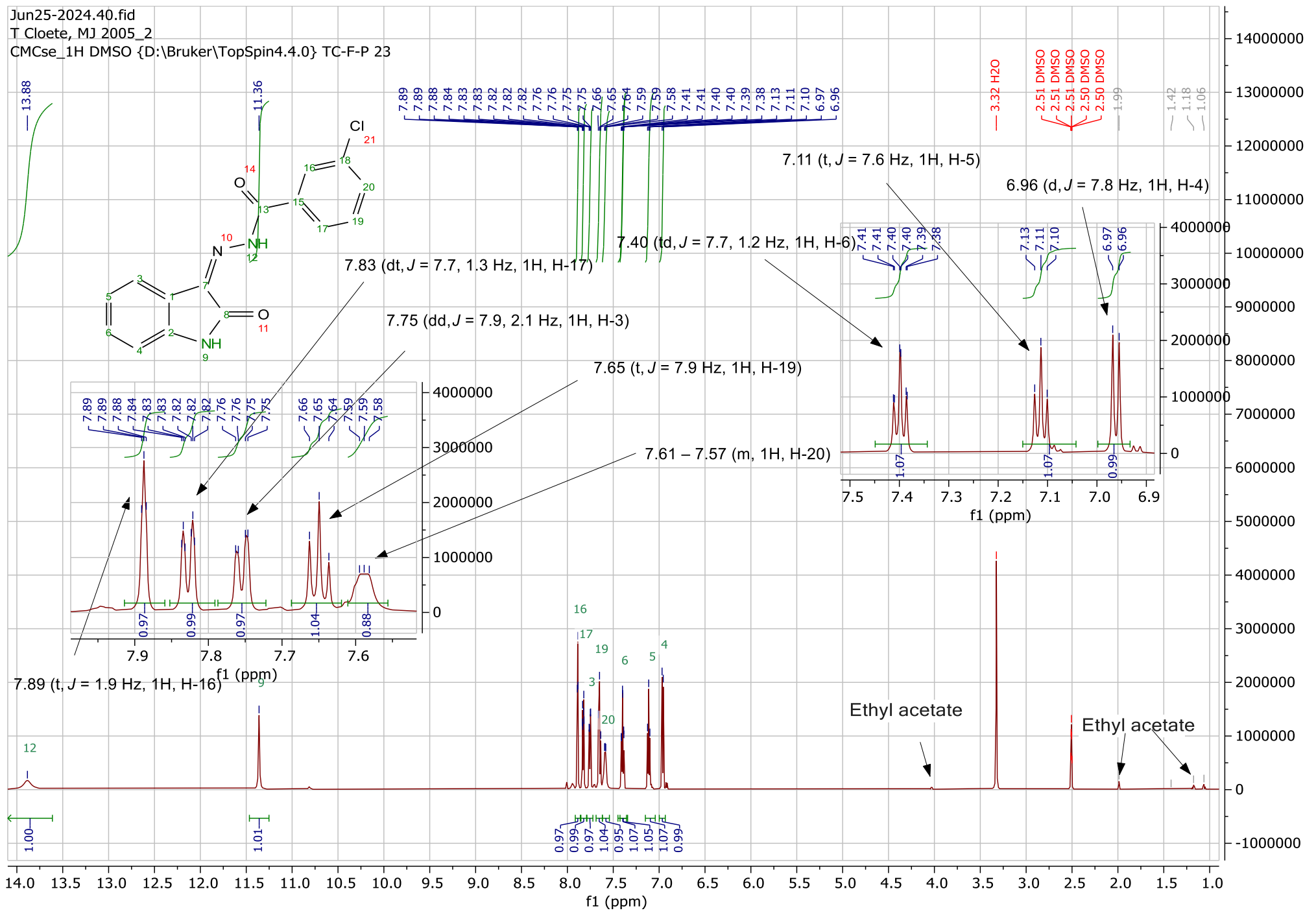

Carbon (^{13}C) NMR spectrum

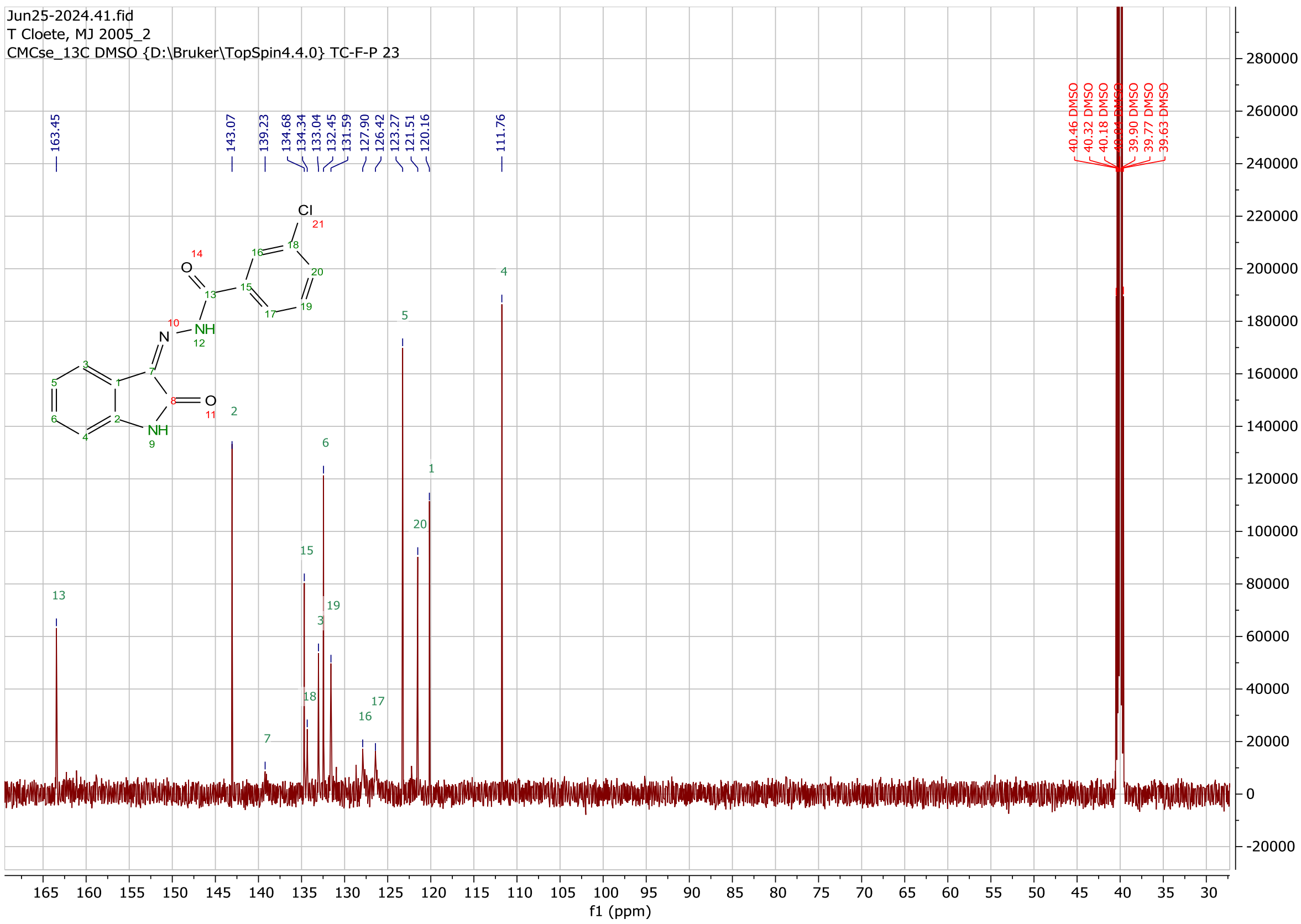

HSQC NMR spectrum

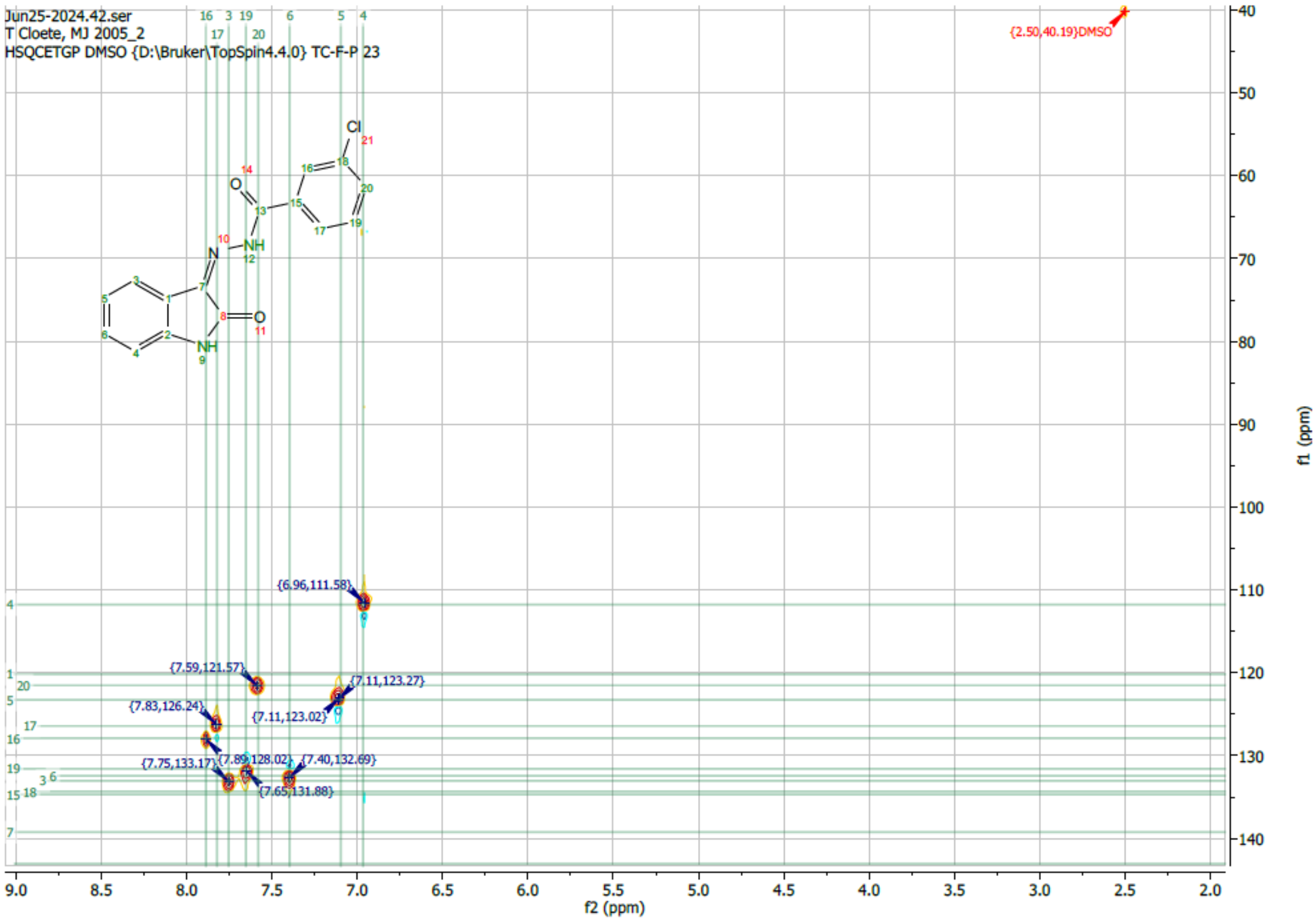

*4-chloro-N'-[(3Z)-2-oxo-1,2-dihydro-3H-indol-3-ylidene]benzohydrazide (**1e**)*

Proton (^{1}H) NMR spectrum

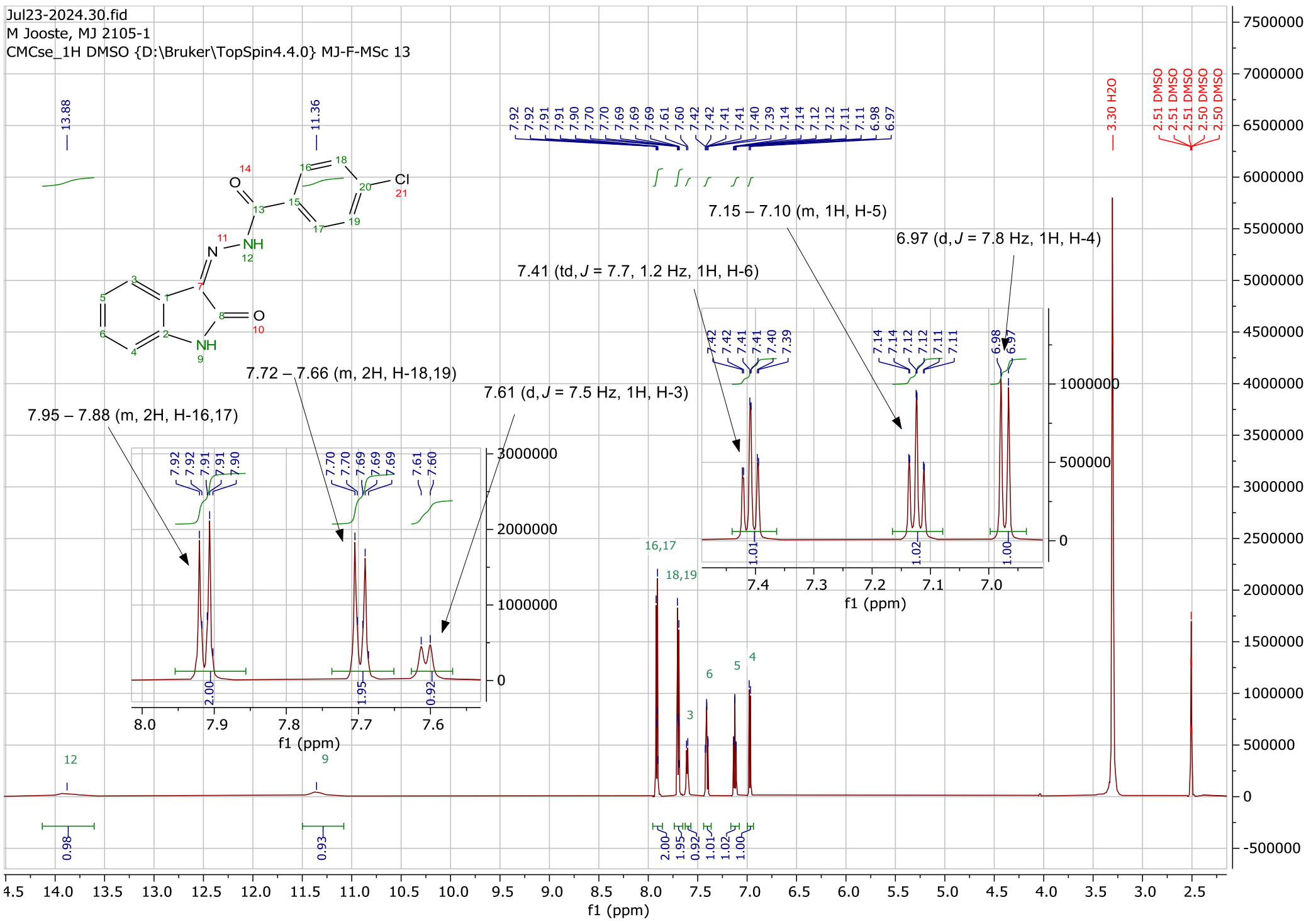

Carbon (^{13}C) NMR spectrum

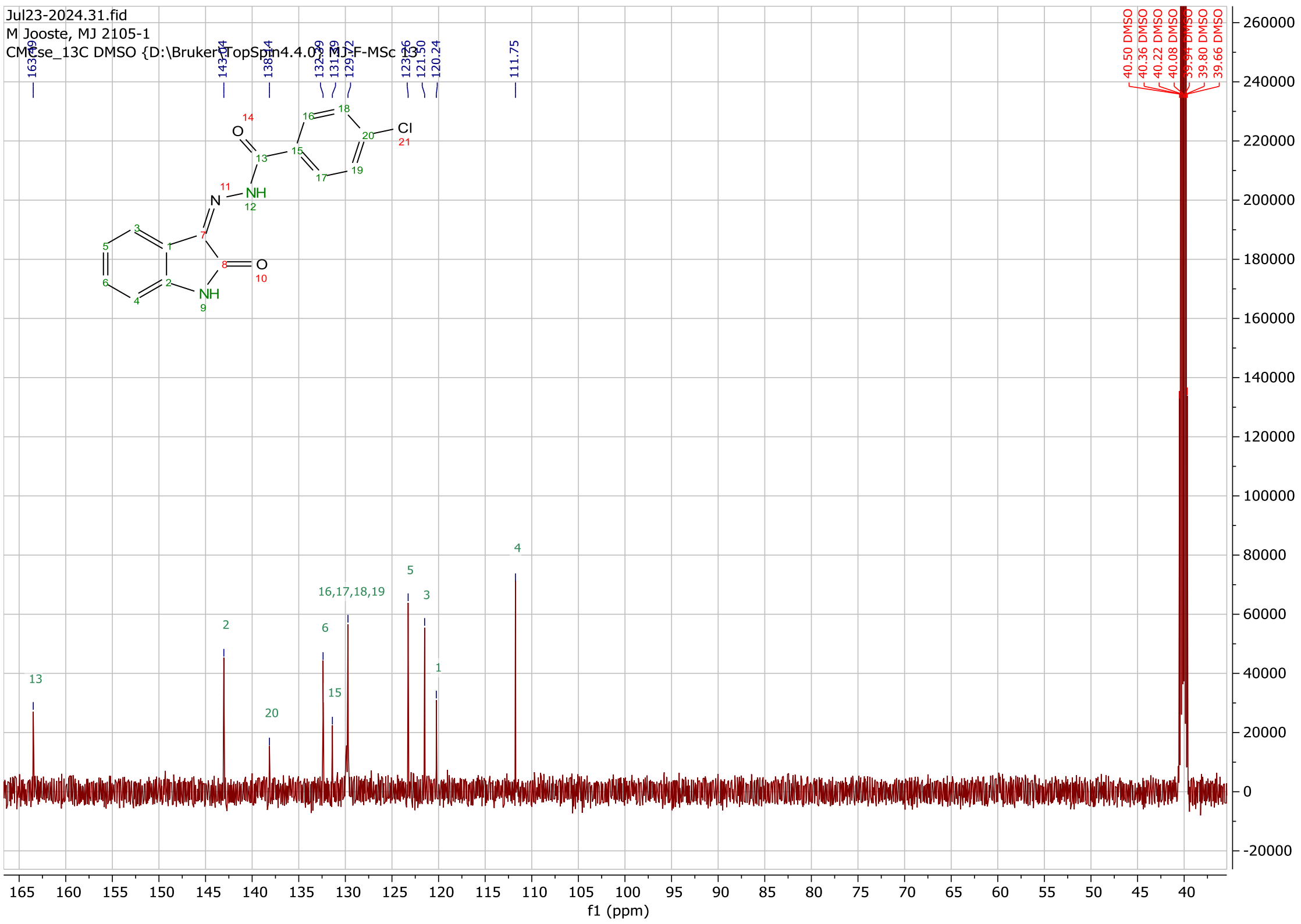

HSQC NMR spectrum

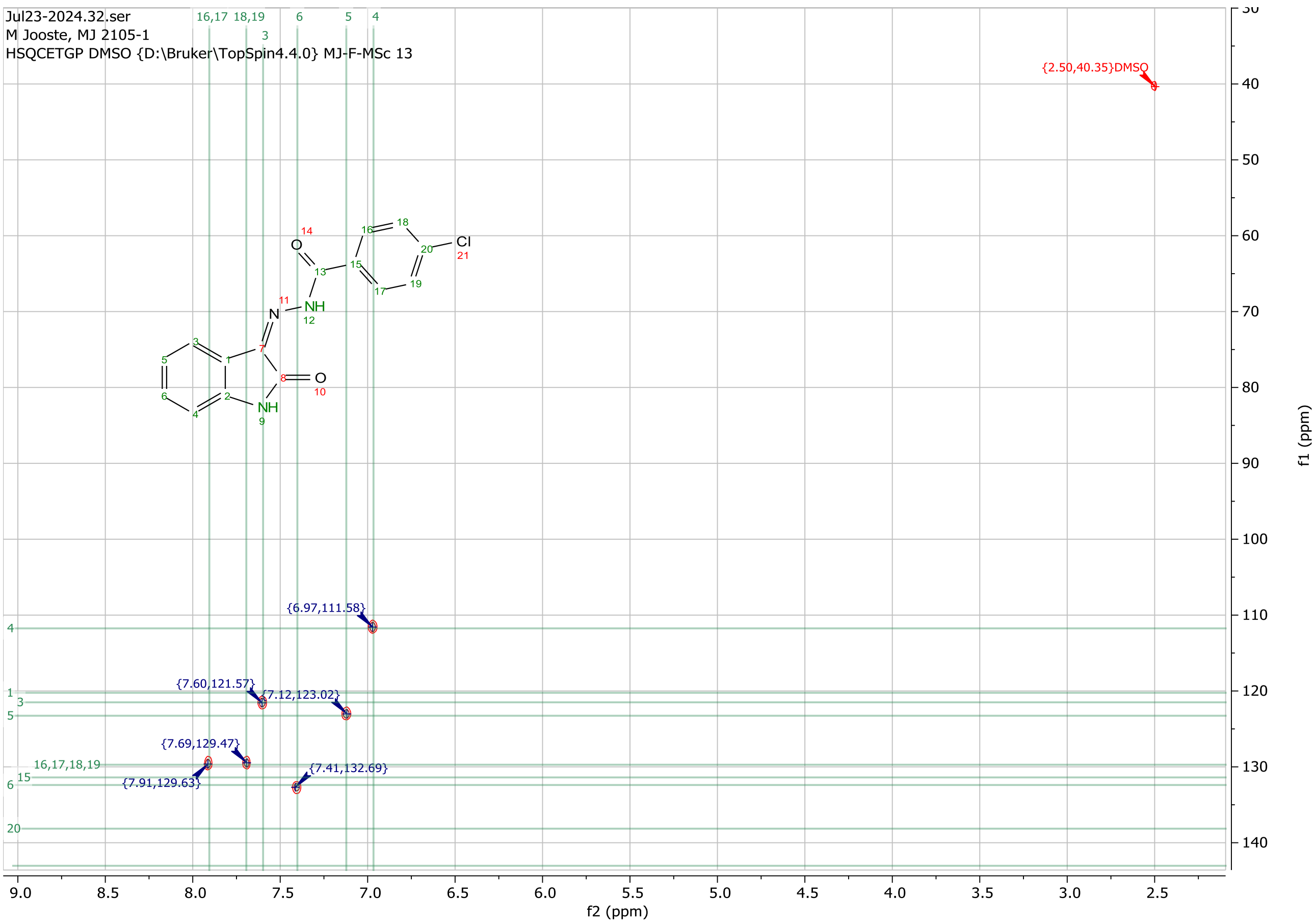

N'-[(3Z)-4-chloro-2-oxo-1,2-dihydro-3H-indol-3-ylidene]benzohydrazide (**2a**)

Proton (^{1}H) NMR spectrum

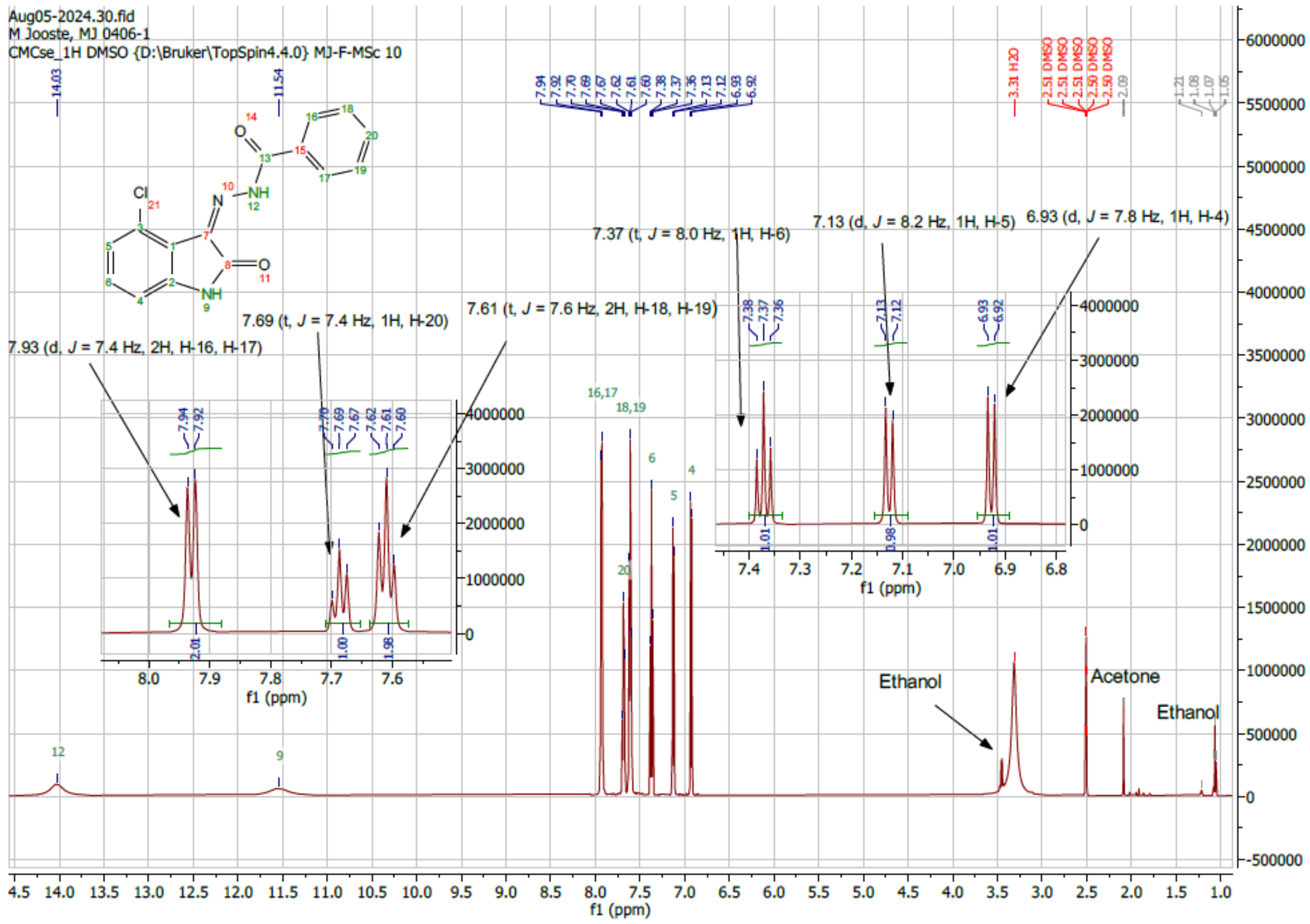

Carbon (^{13}C) NMR spectrum

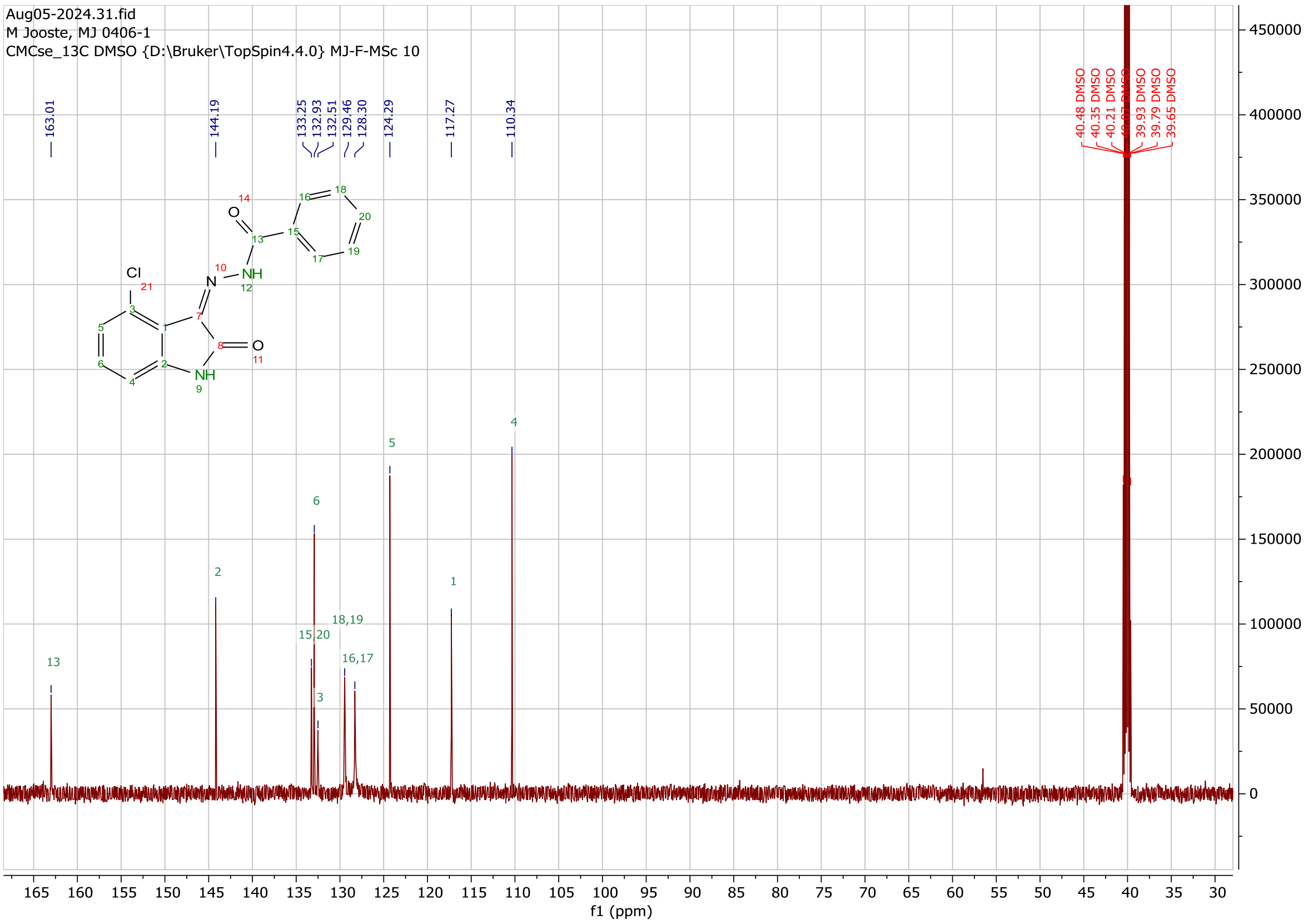

HSQC NMR spectrum

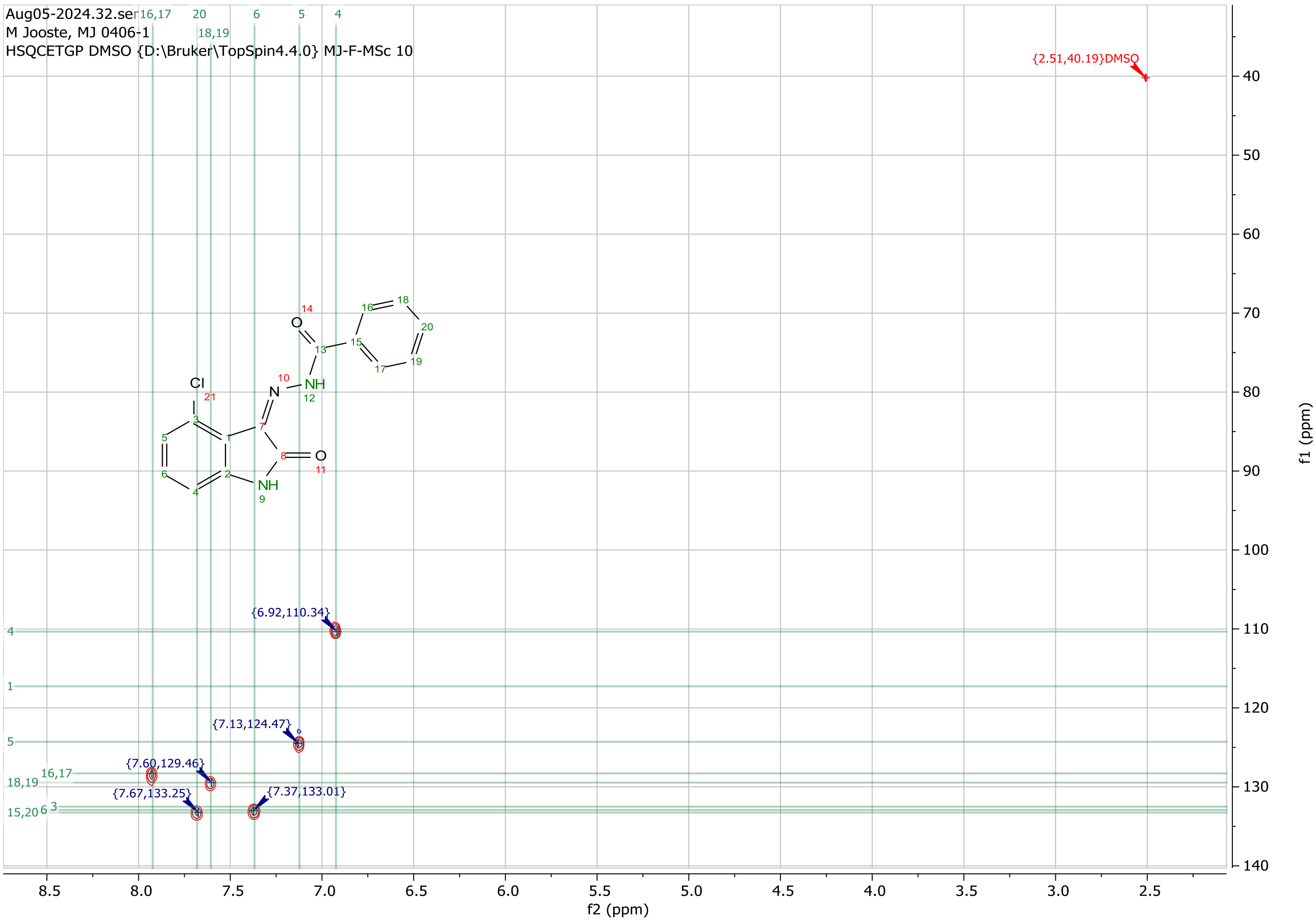

N'-[(3Z)-4-chloro-2-oxo-1,2-dihydro-3H-indol-3-ylidene]-2-hydroxybenzohydrazide (**2b**)

Proton (^{1}H) NMR spectrum

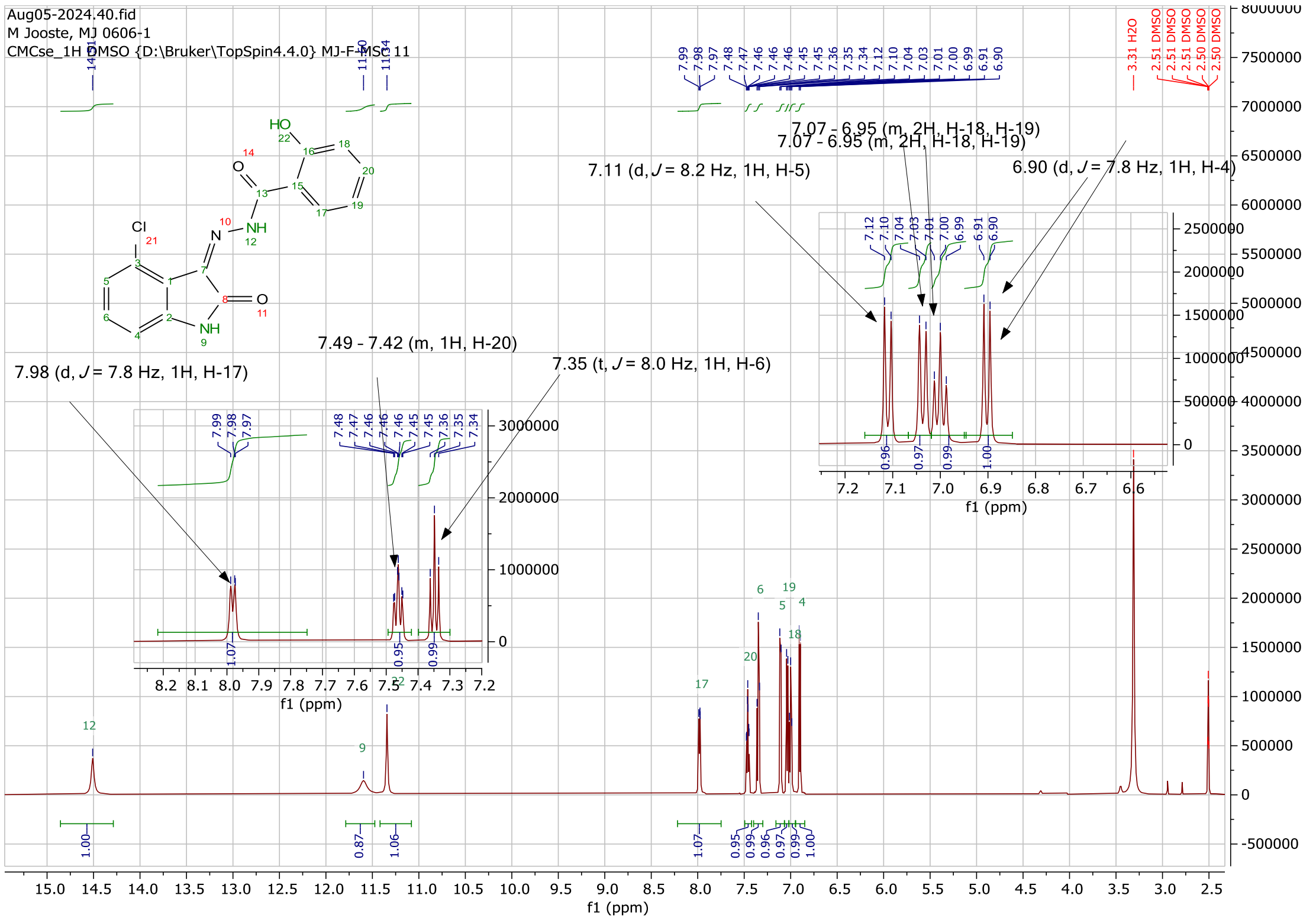

Carbon (^{13}C) NMR spectrum

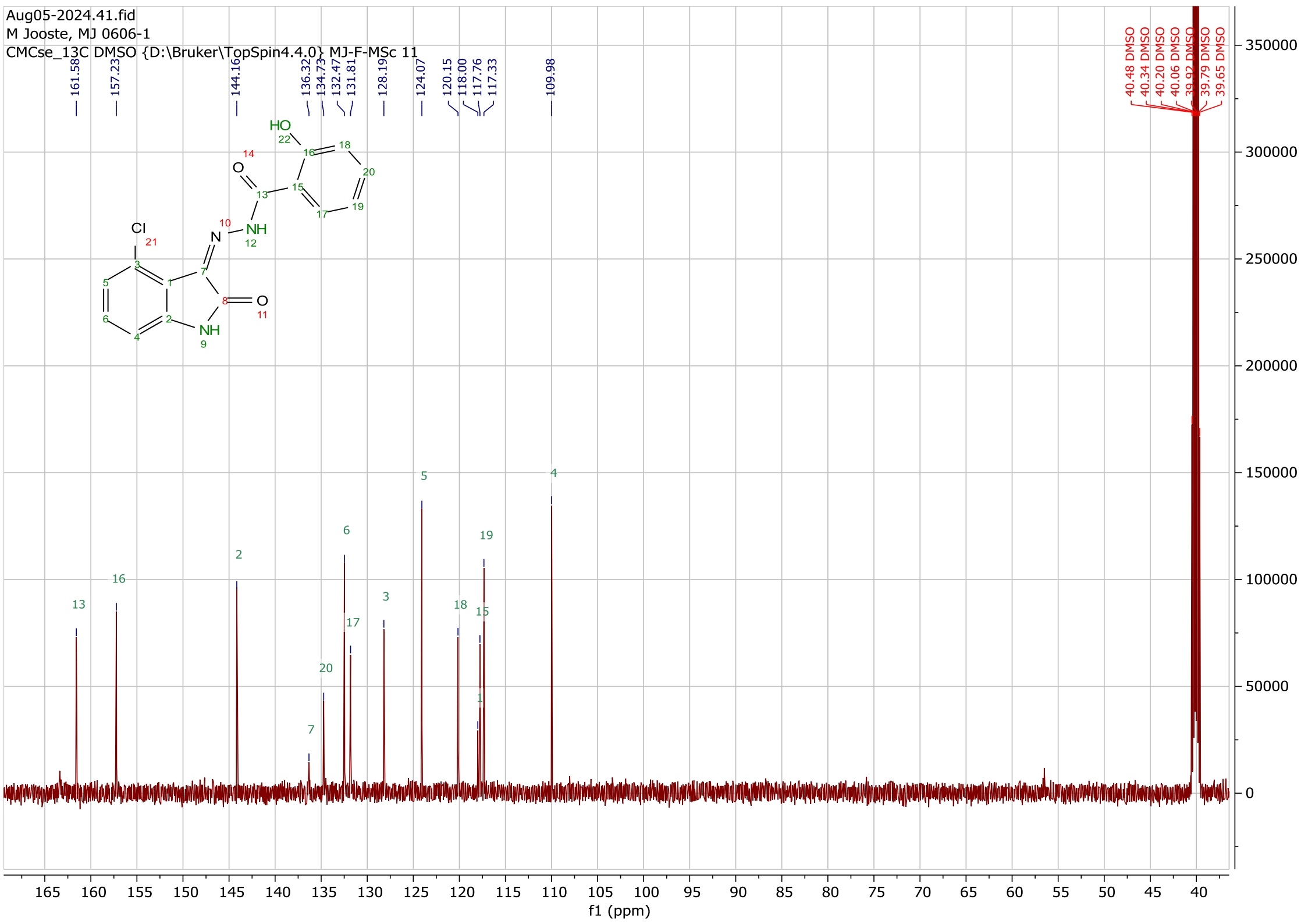

HSQC NMR spectrum

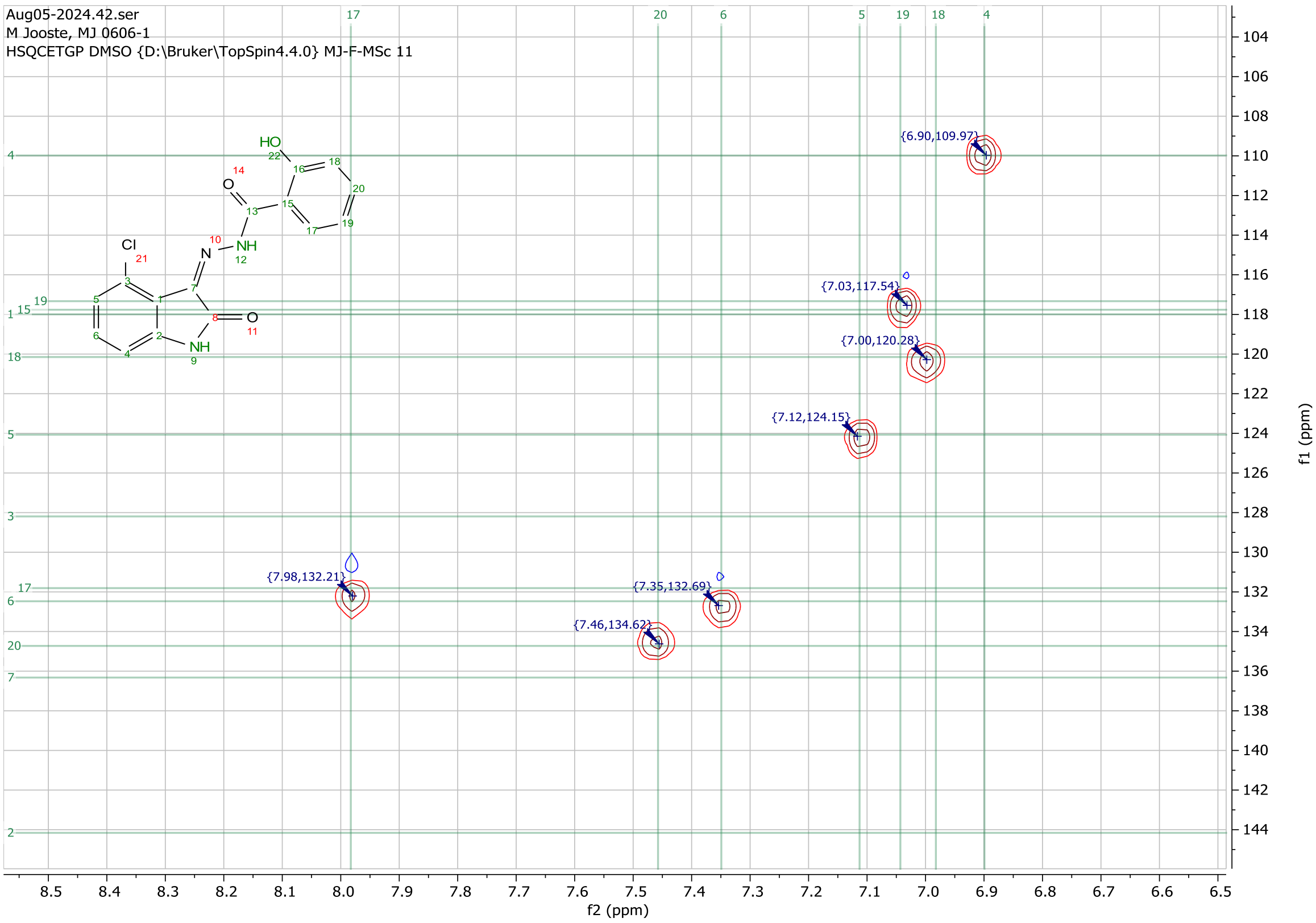

N'-[(3Z)-4-chloro-2-oxo-1,2-dihydro-3H-indol-3-ylidene]-3-hydroxybenzohydrazide (**2c**)

Proton (^{1}H) NMR spectrum

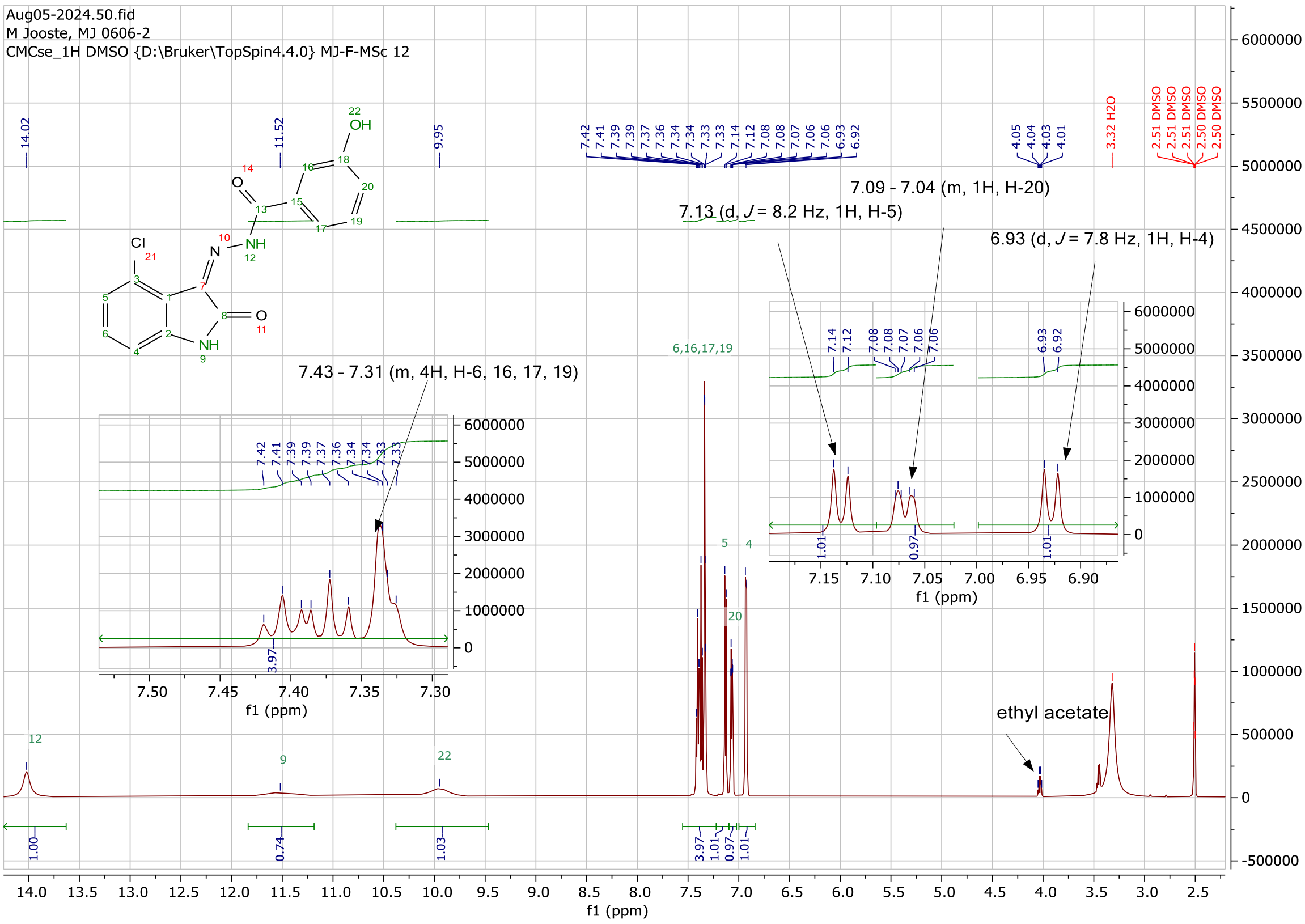

Carbon (^{13}C) NMR spectrum

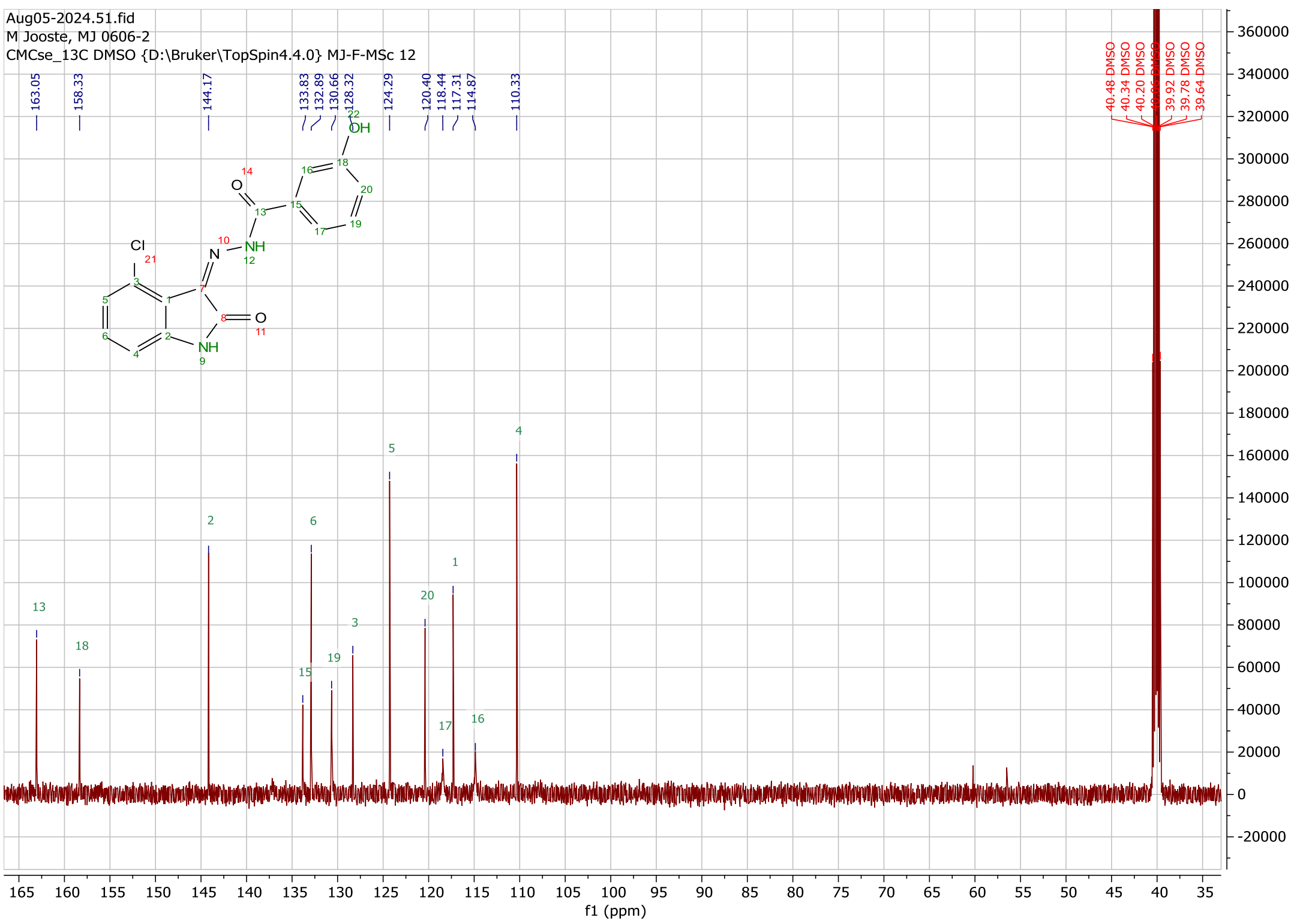

HSQC NMR spectrum

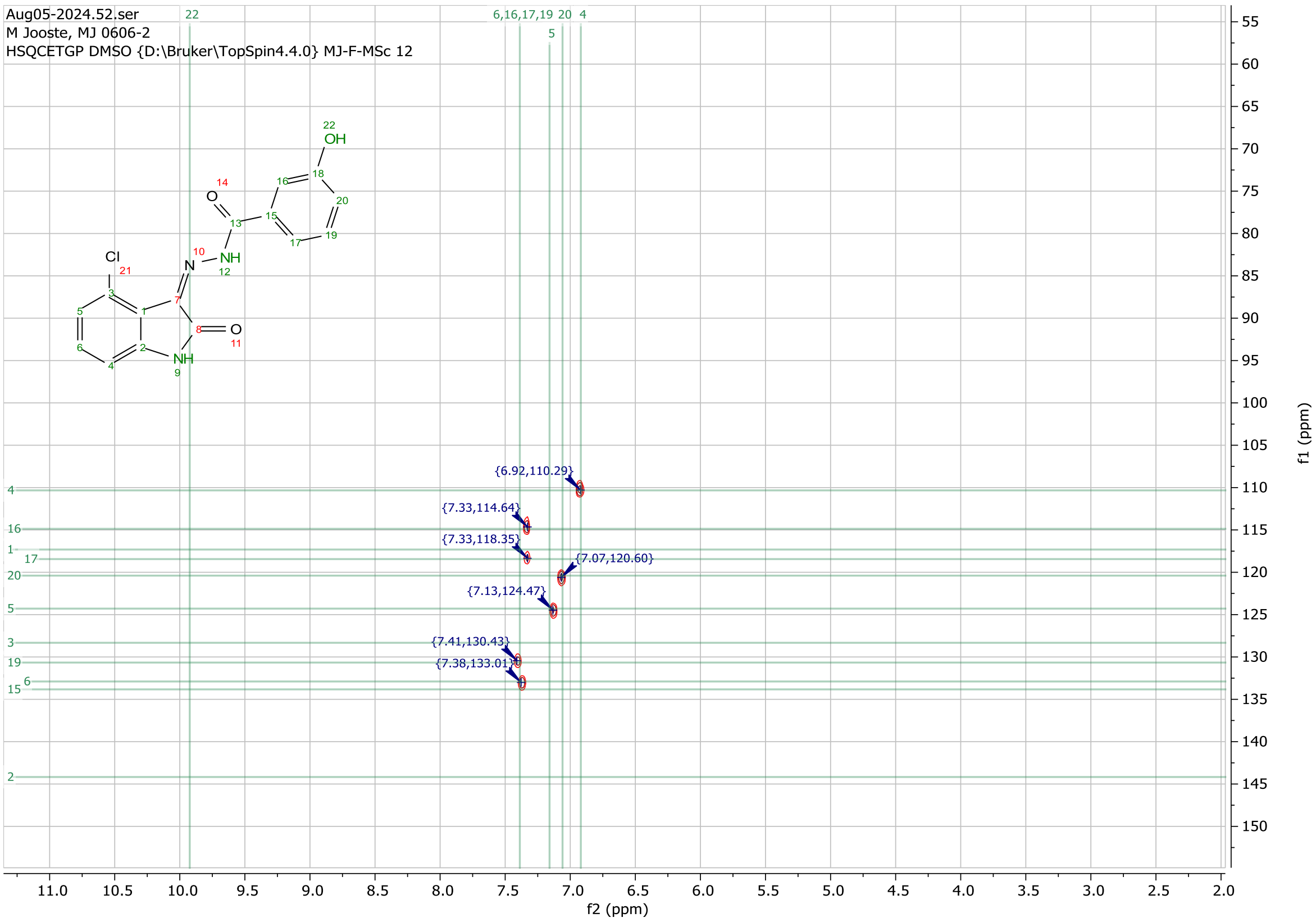

4-chloro-N'-[(3Z)-4-chloro-2-oxo-1,2-dihydro-3H-indol-3-ylidene]benzohydrazide (***2d***)

Proton (^{1}H) NMR spectrum

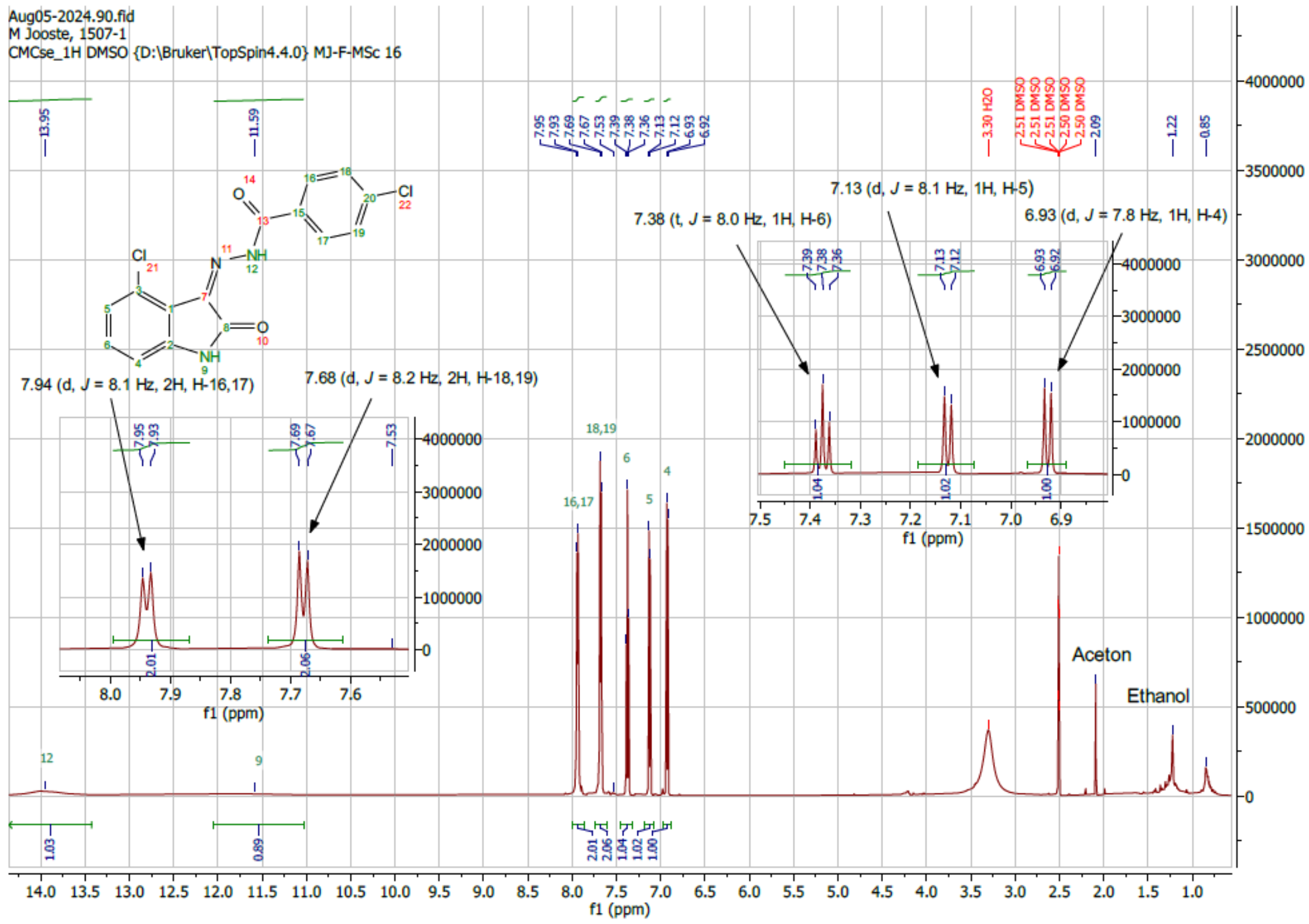

Carbon (^{13}C) NMR spectrum

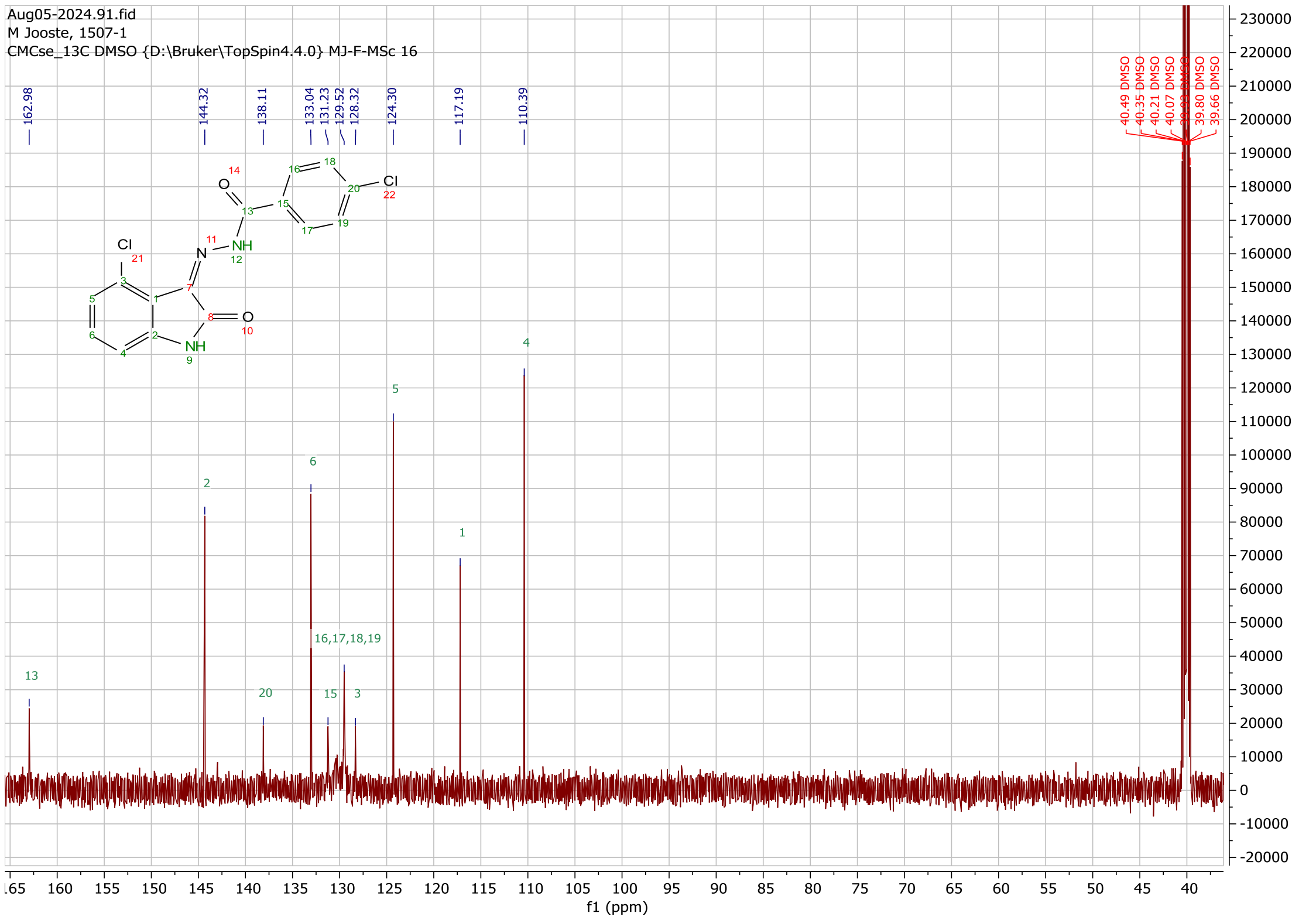

HSQC NMR spectrum

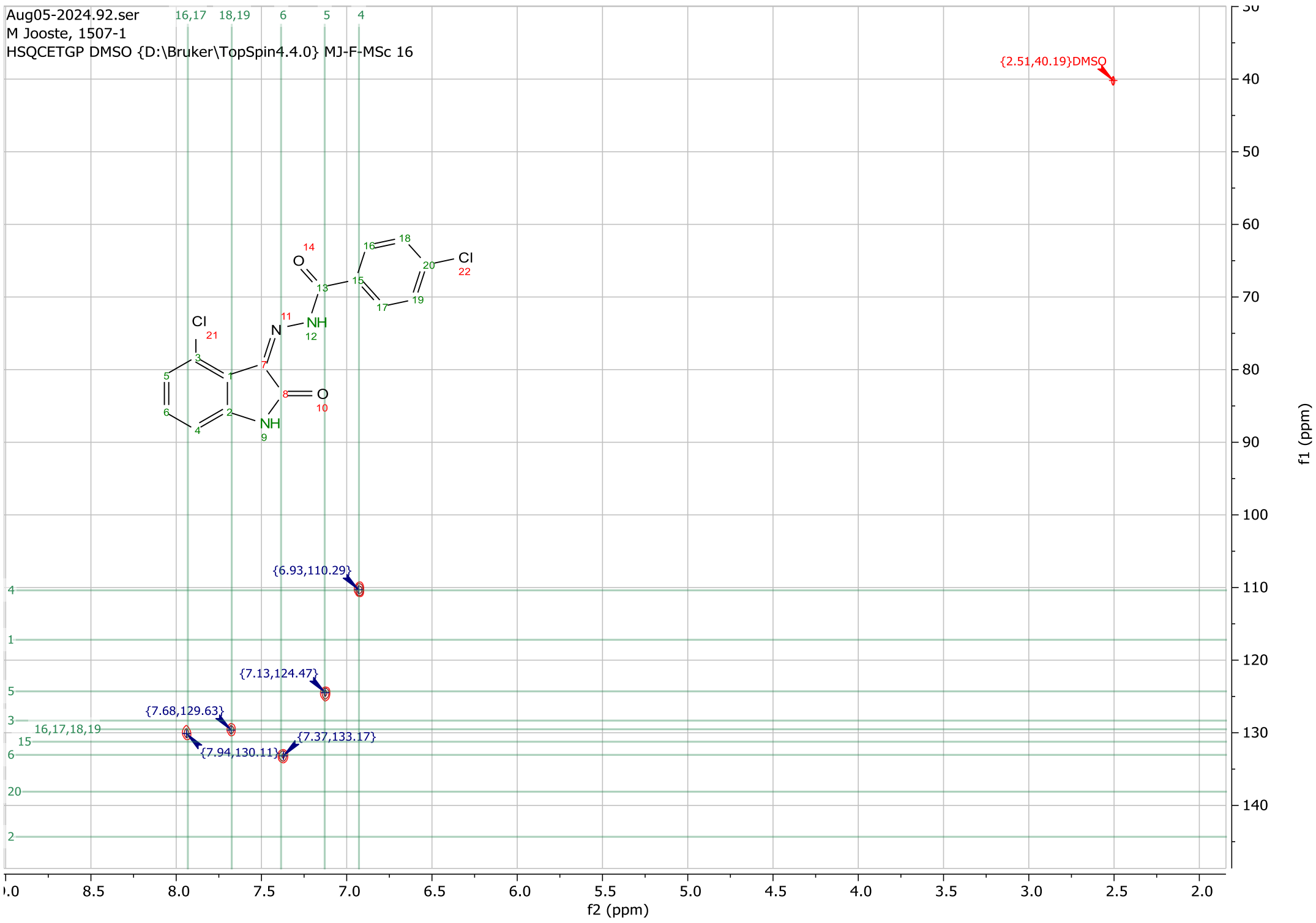

*N'-[(3Z)-5-chloro-2-oxo-1,2-dihydro-3H-indol-3-ylidene]benzohydrazide (**3a**)*

Proton (^{1}H) NMR spectrum

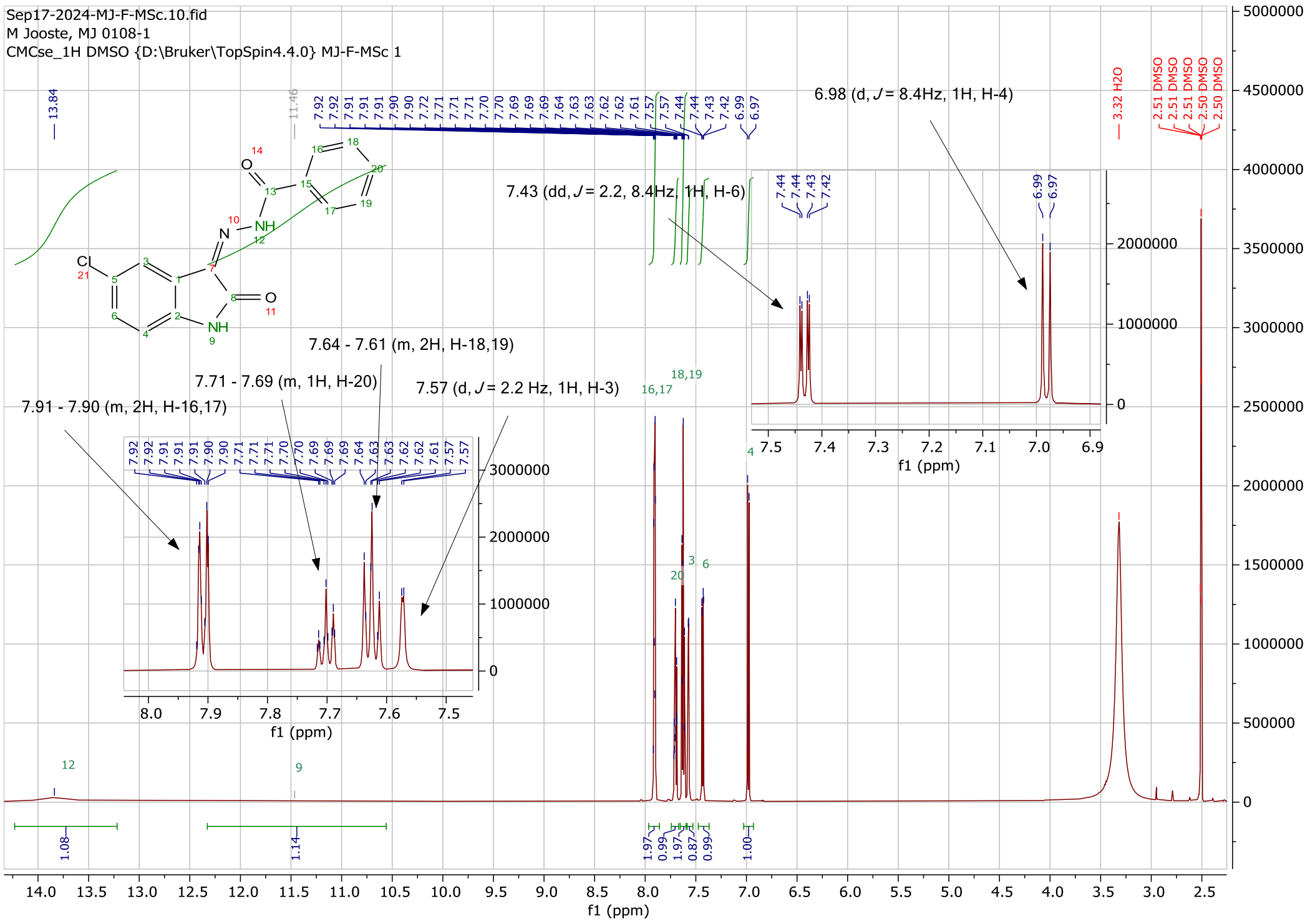

Carbon (^{13}C) NMR spectrum

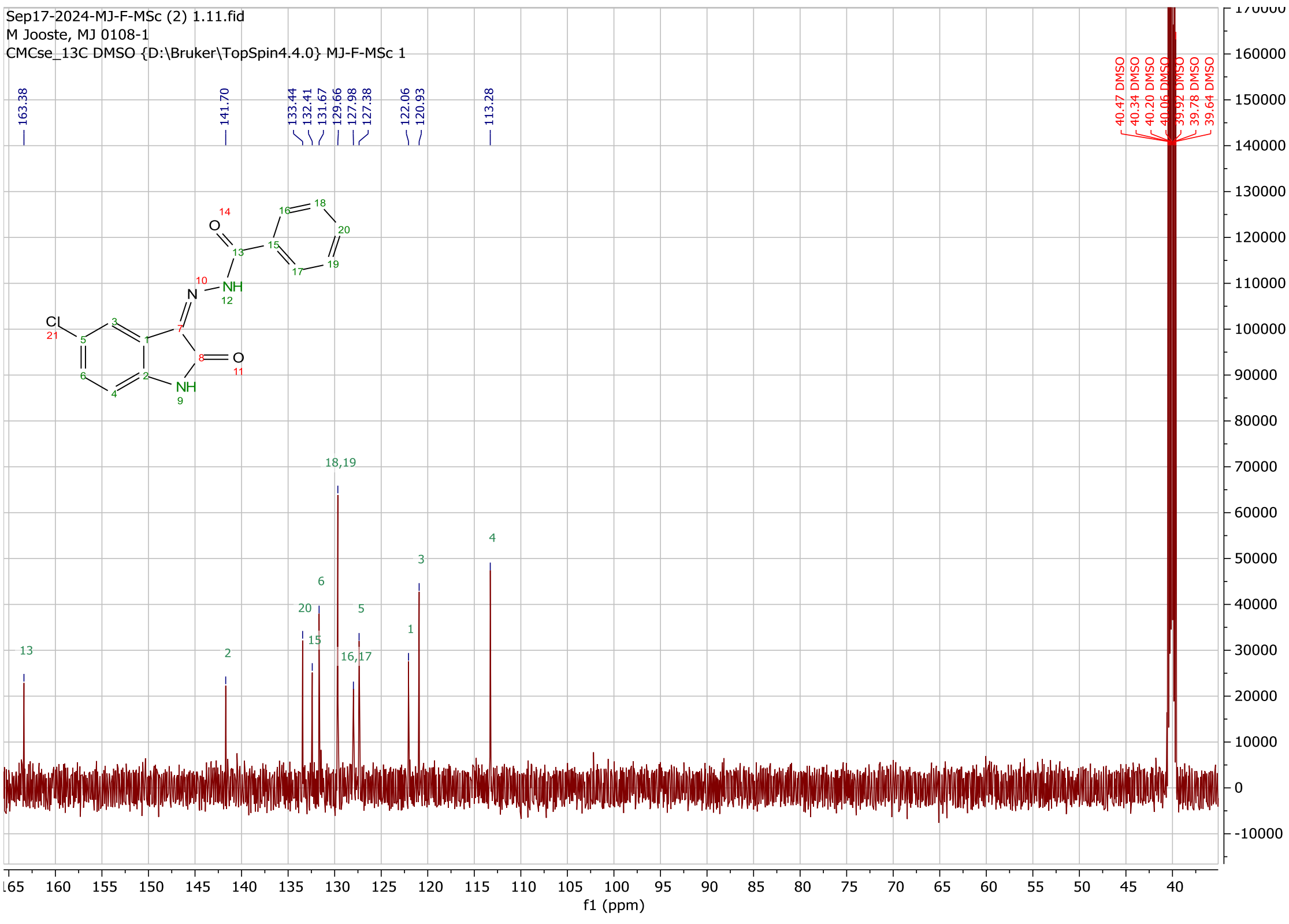

HSQC NMR spectrum

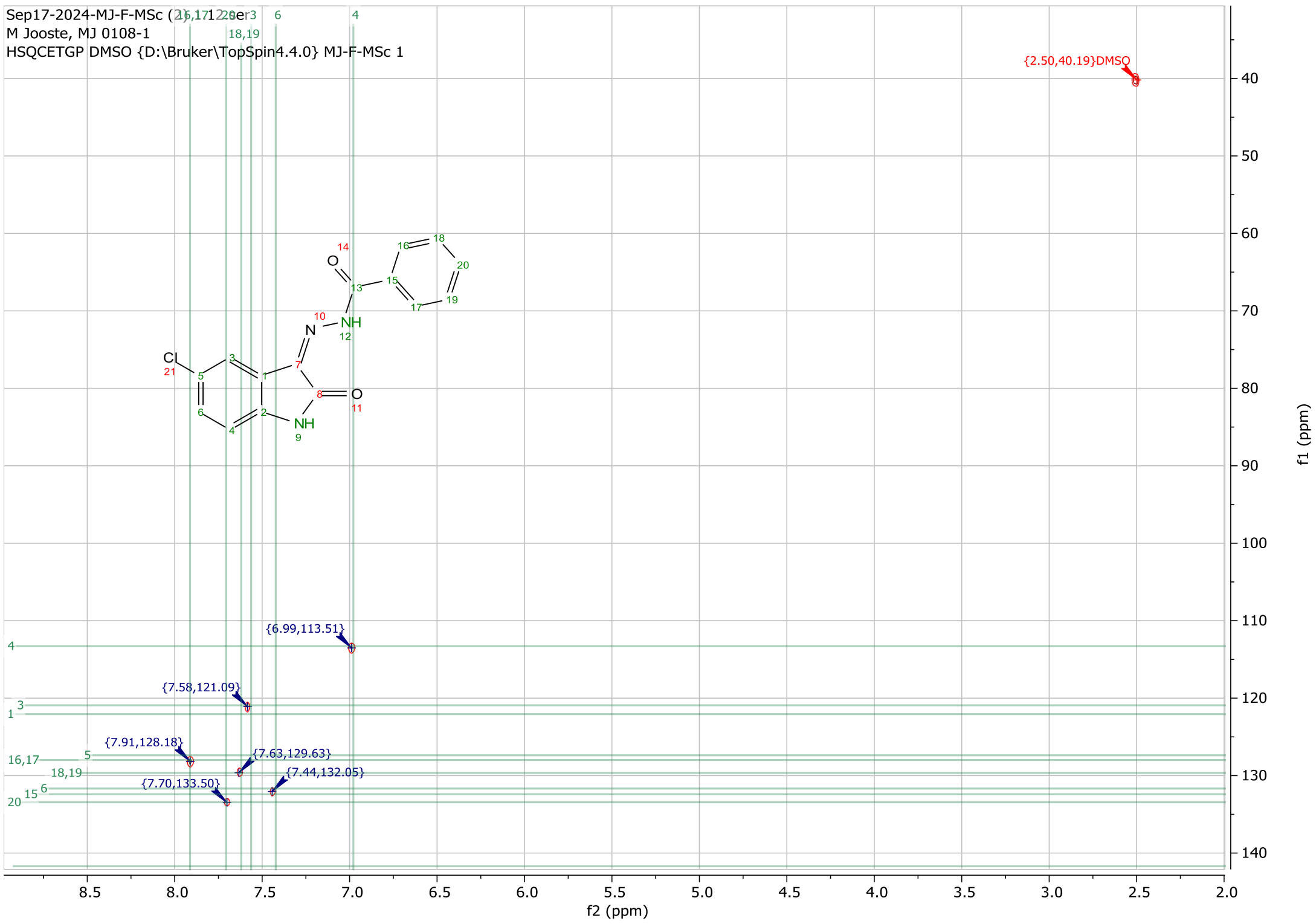

*N'-[(3Z)-5-chloro-2-oxo-1,2-dihydro-3H-indol-3-ylidene]-3-hydroxybenzohydrazide (**3b**)*

Proton (^{1}H) NMR spectrum

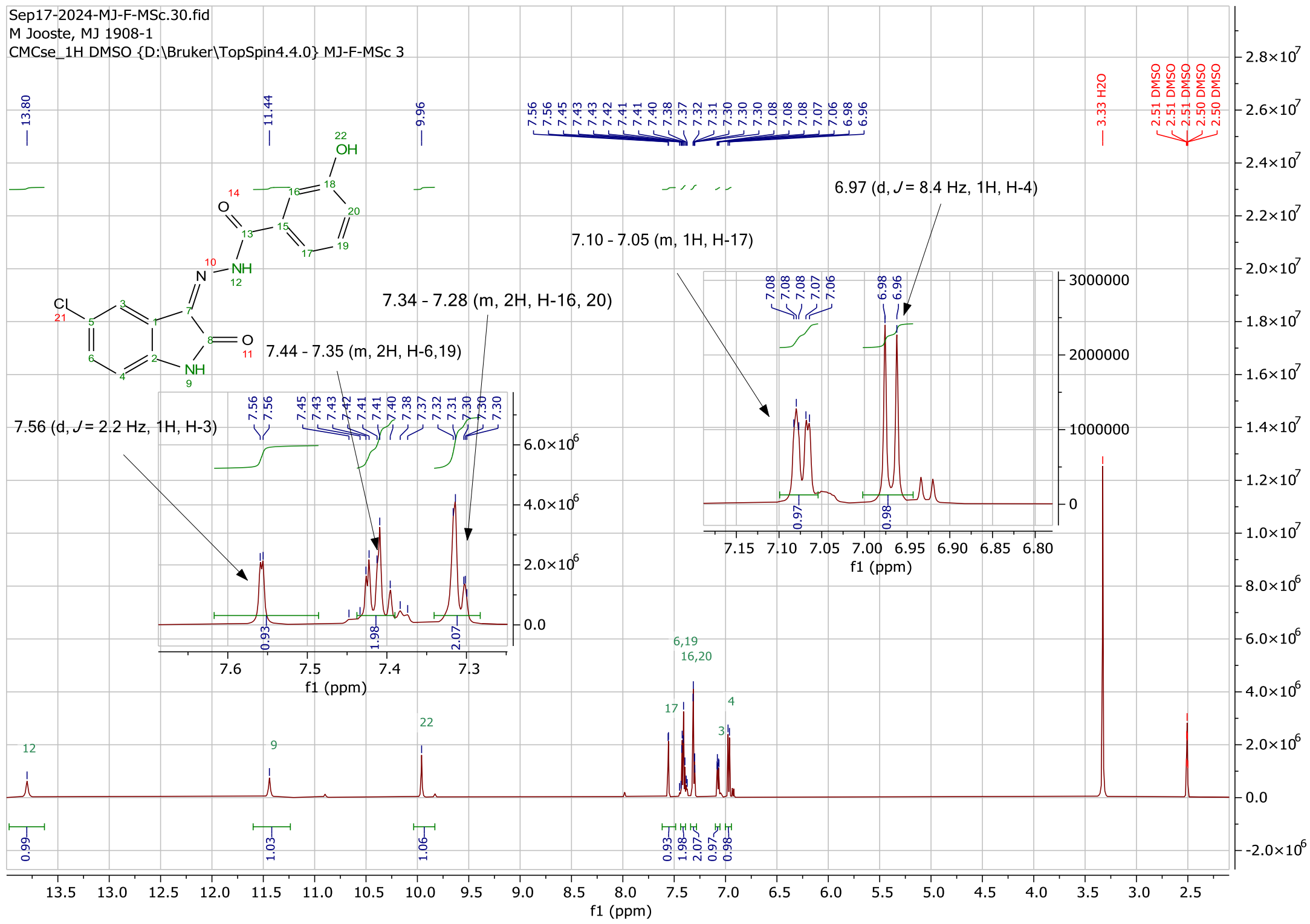

Carbon (^{13}C) NMR spectrum

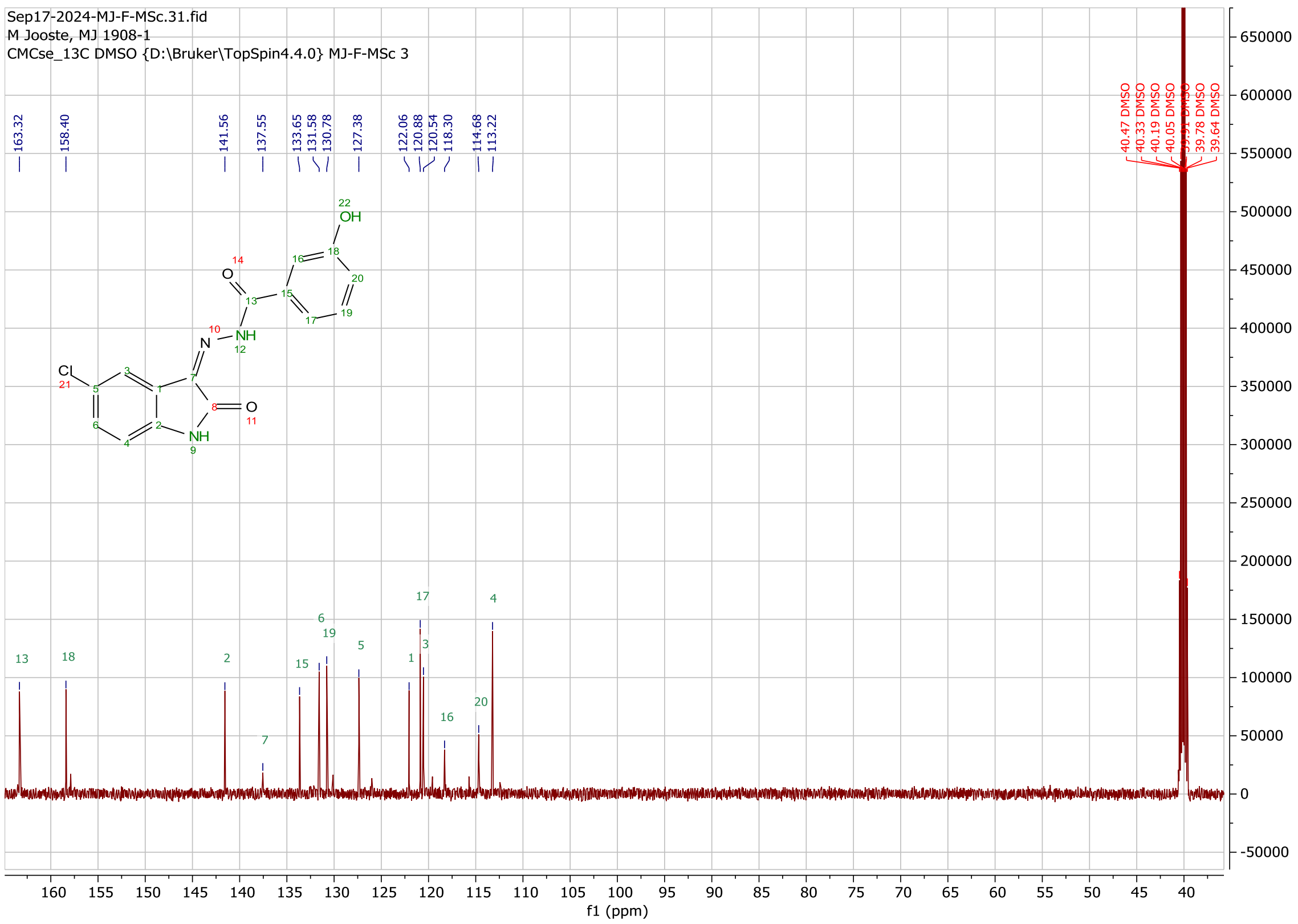

HSQC NMR spectrum

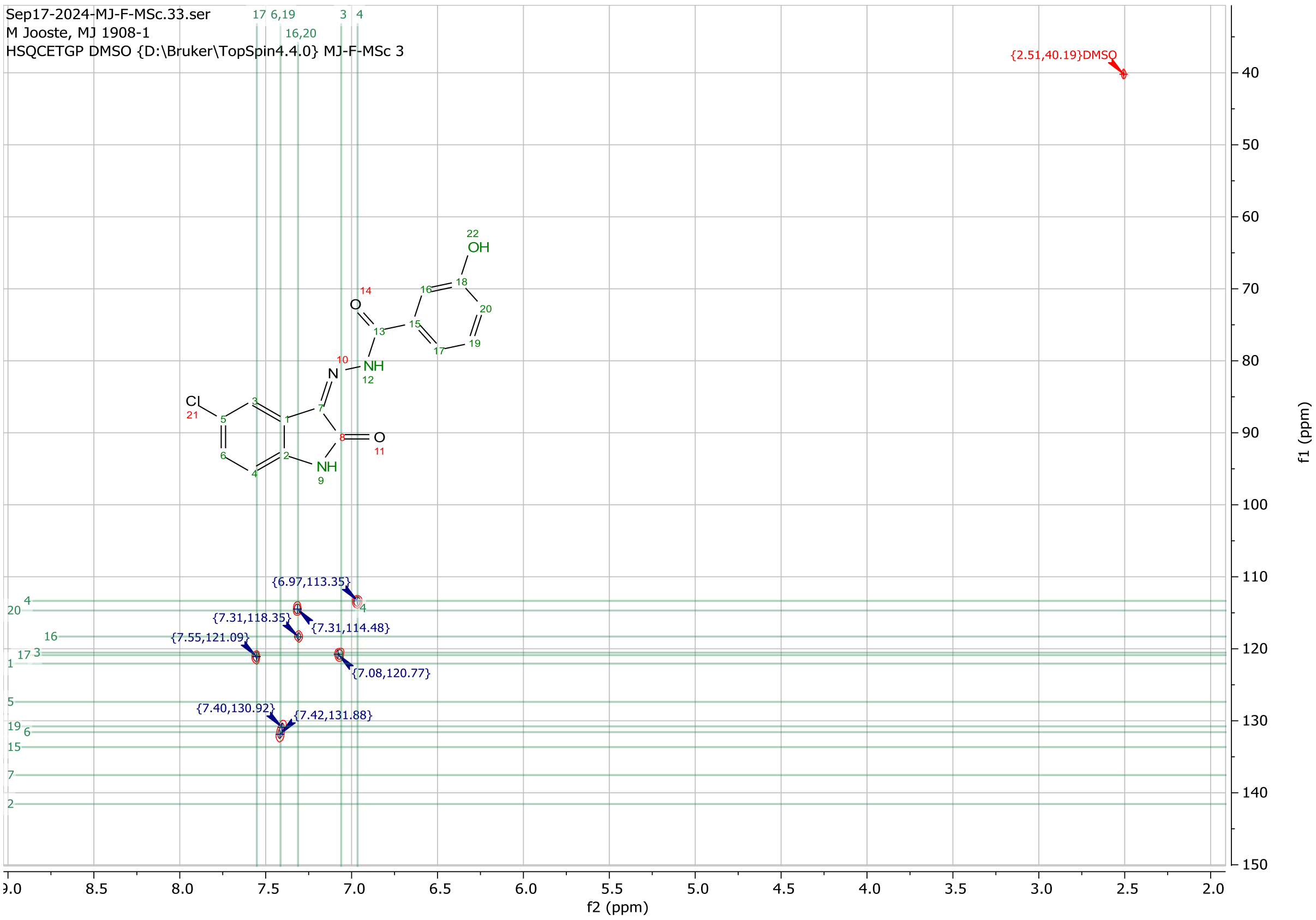

*2-chloro-N'-[(3Z)-5-chloro-2-oxo-1,2-dihydro-3H-indol-3-ylidene]benzohydrazide (**3c**)*

Proton (^{1}H) NMR spectrum

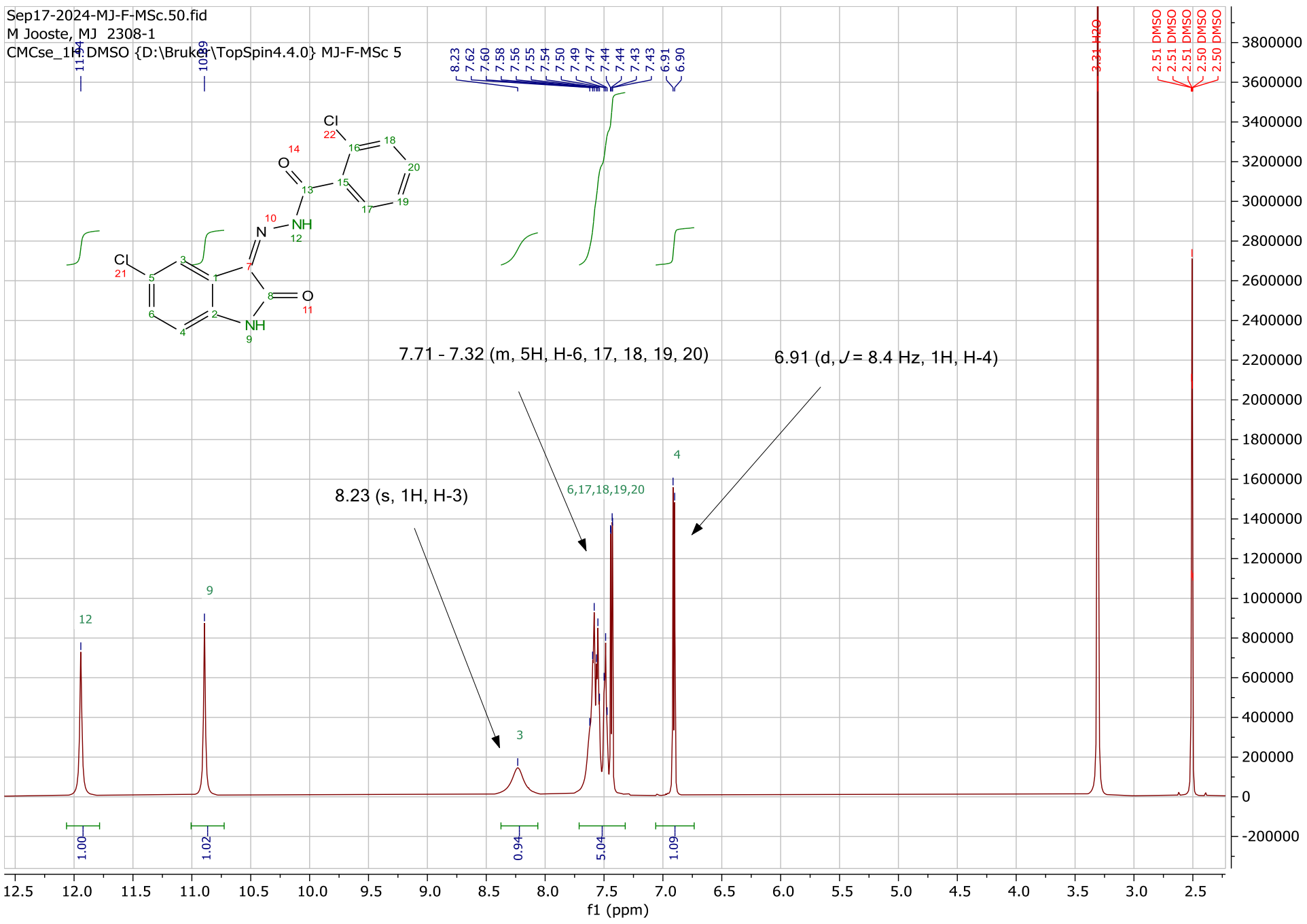

Carbon (^{13}C) NMR spectrum

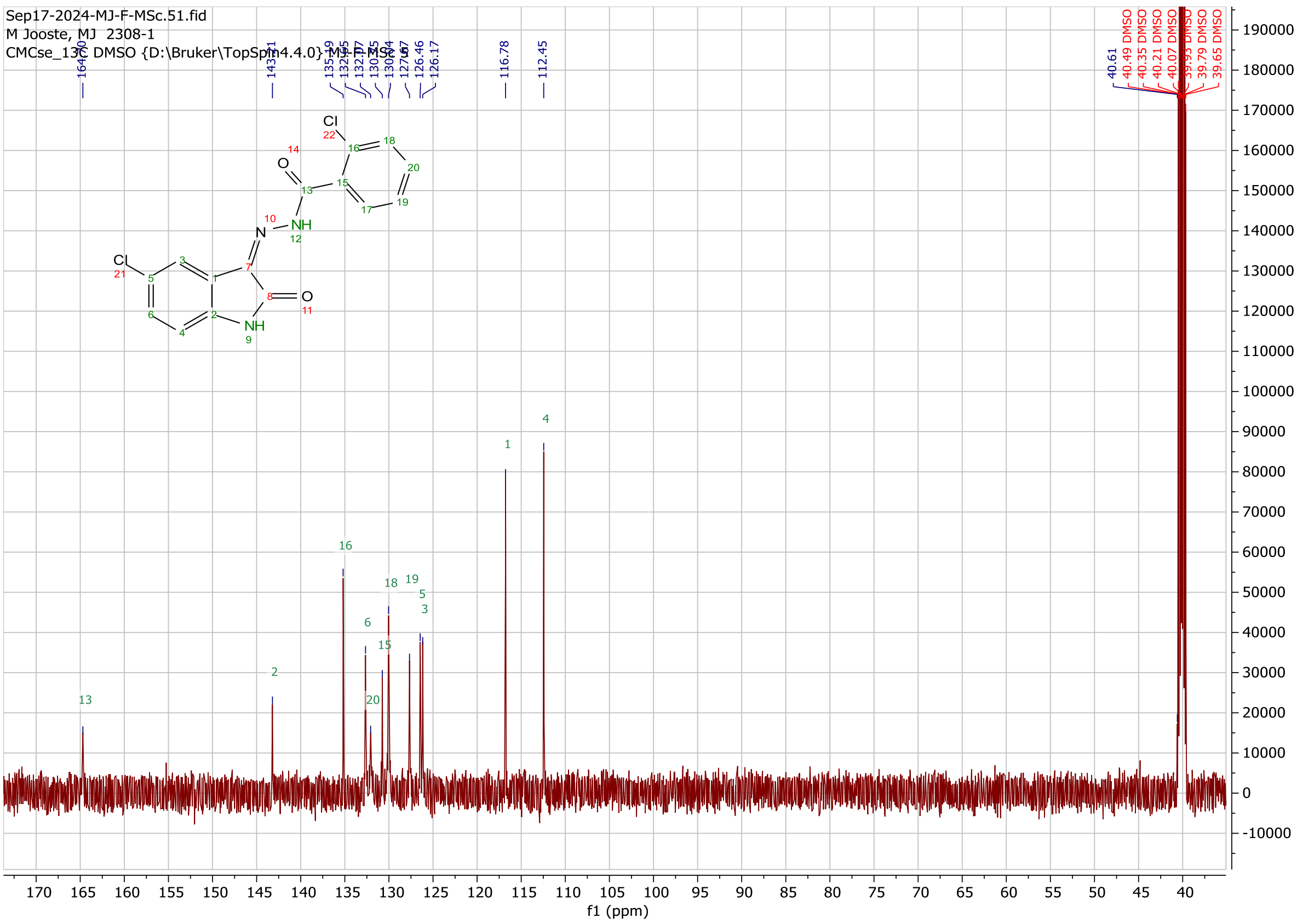

HSQC NMR spectrum

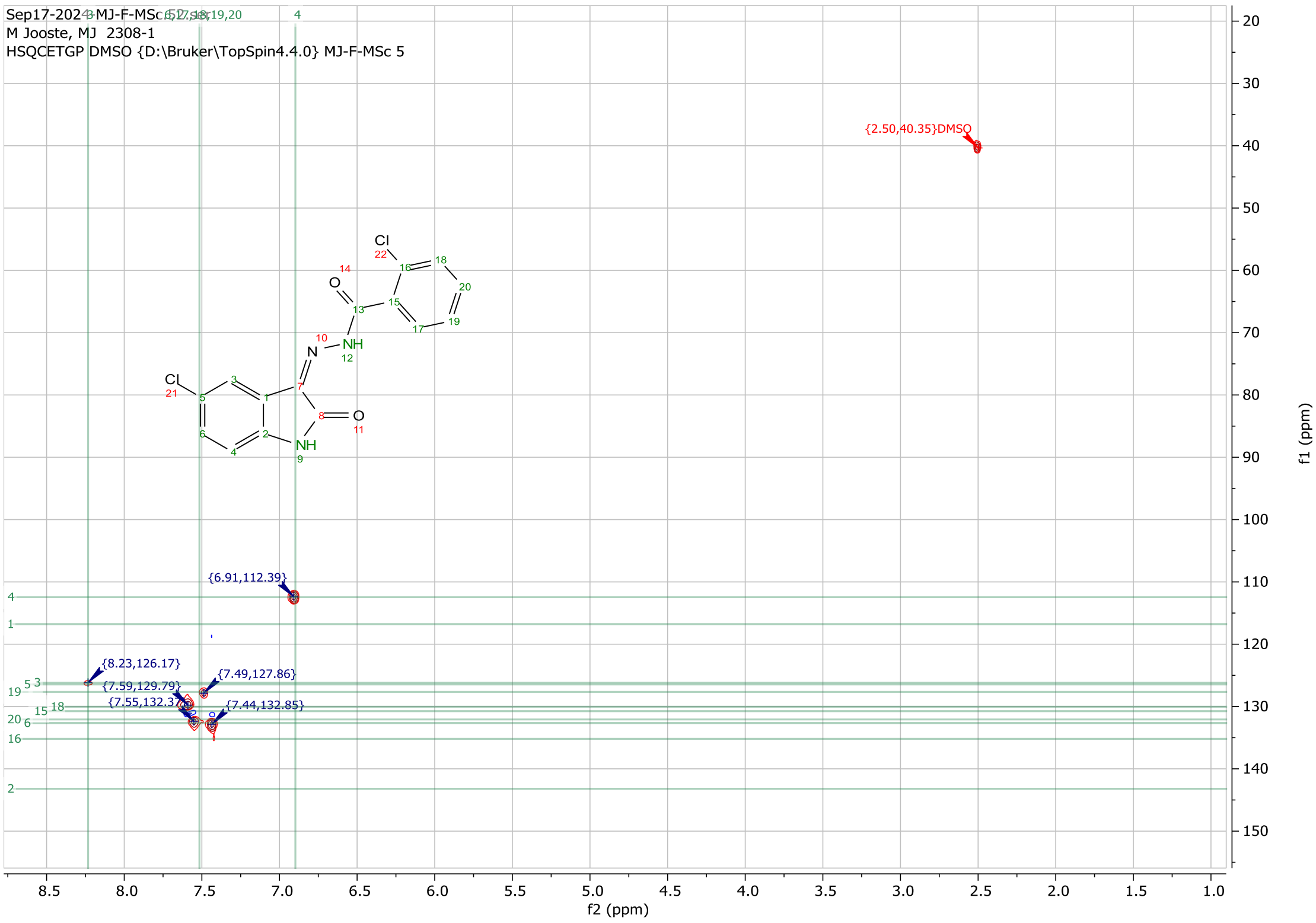

N'-[(3Z)-6-chloro-2-oxo-1,2-dihydro-3H-indol-3-ylidene]benzohydrazide (***4a***)

Proton (^{1}H) NMR spectrum

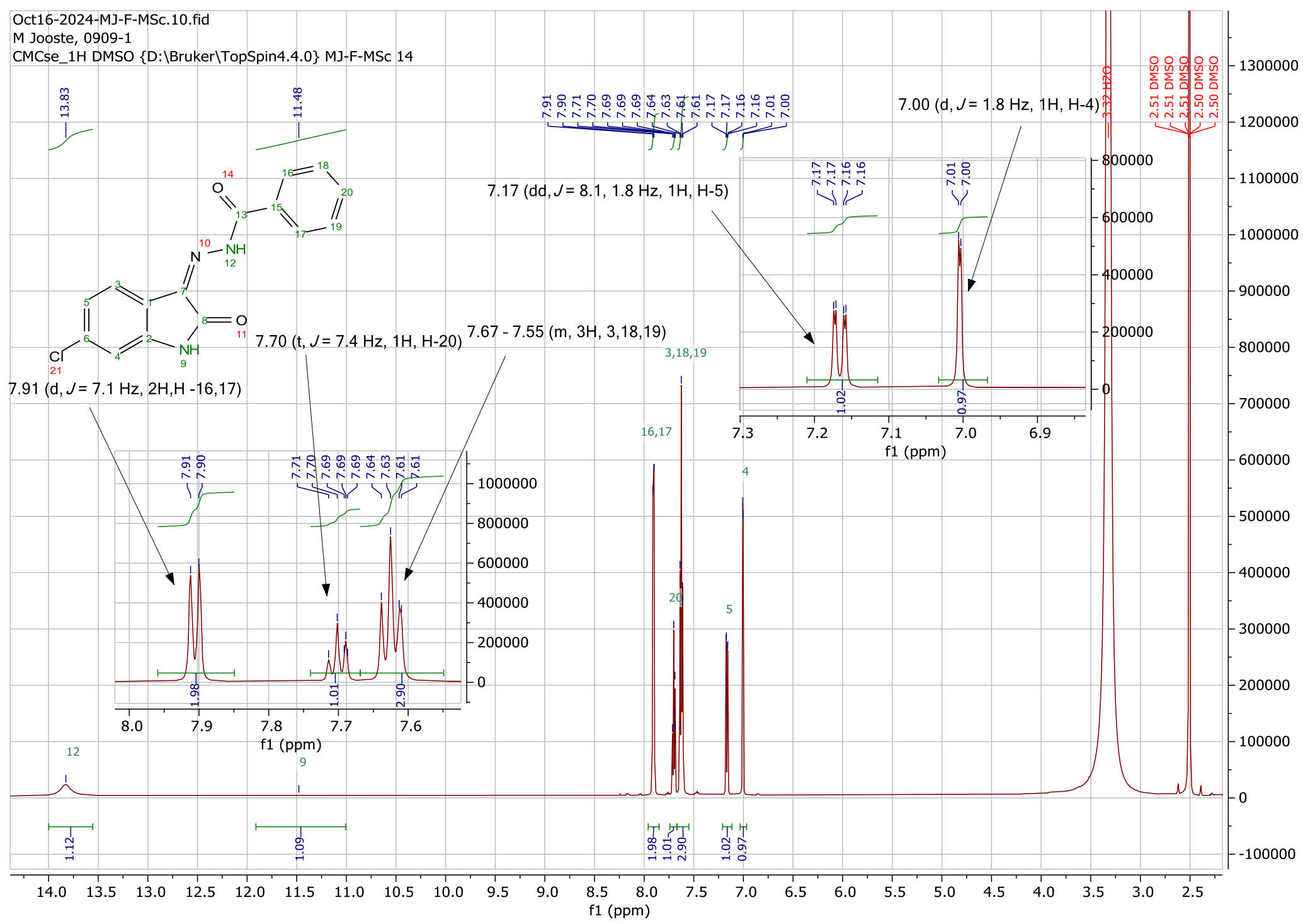

Carbon (^{13}C) NMR spectrum

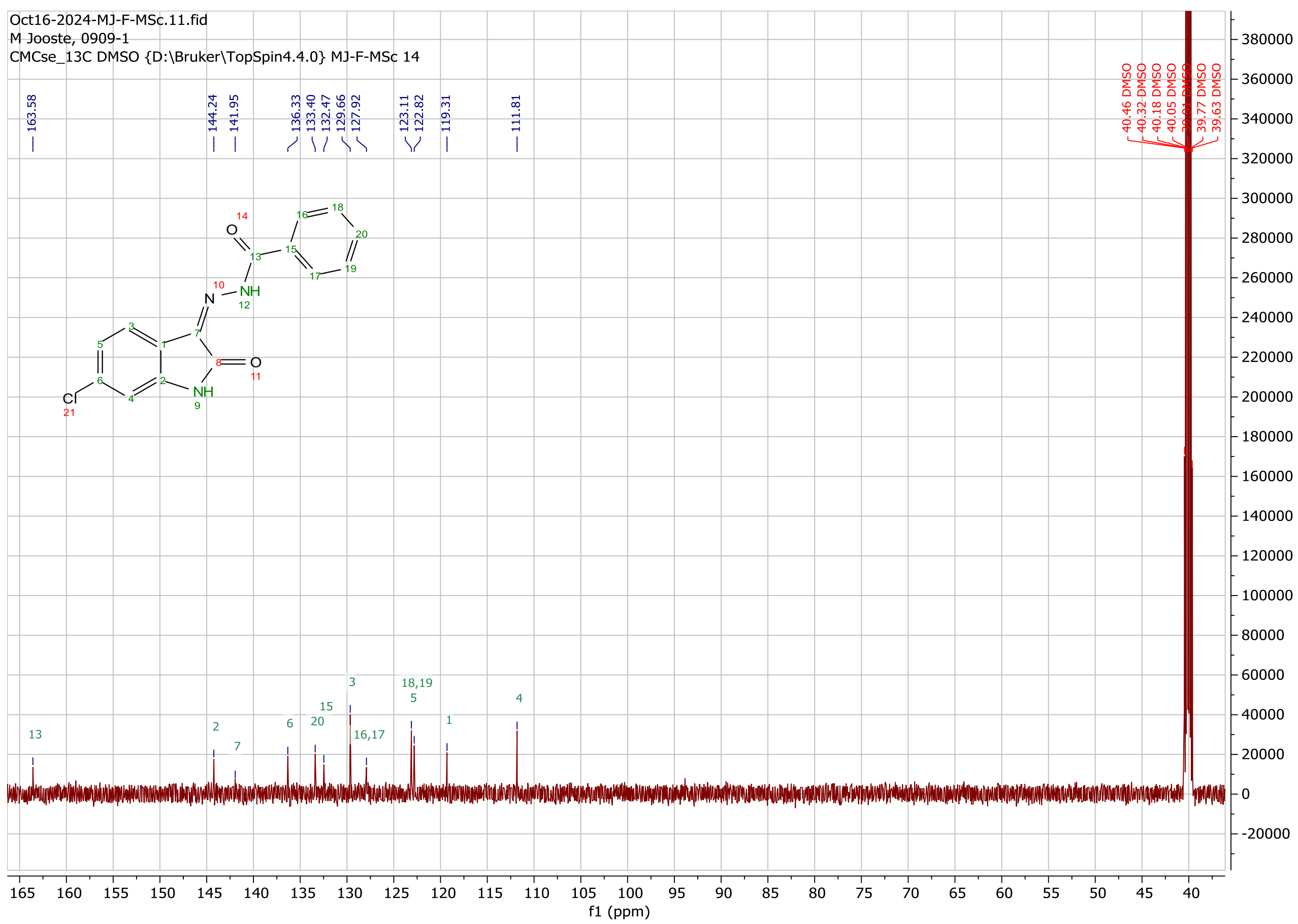

HSQC NMR spectrum

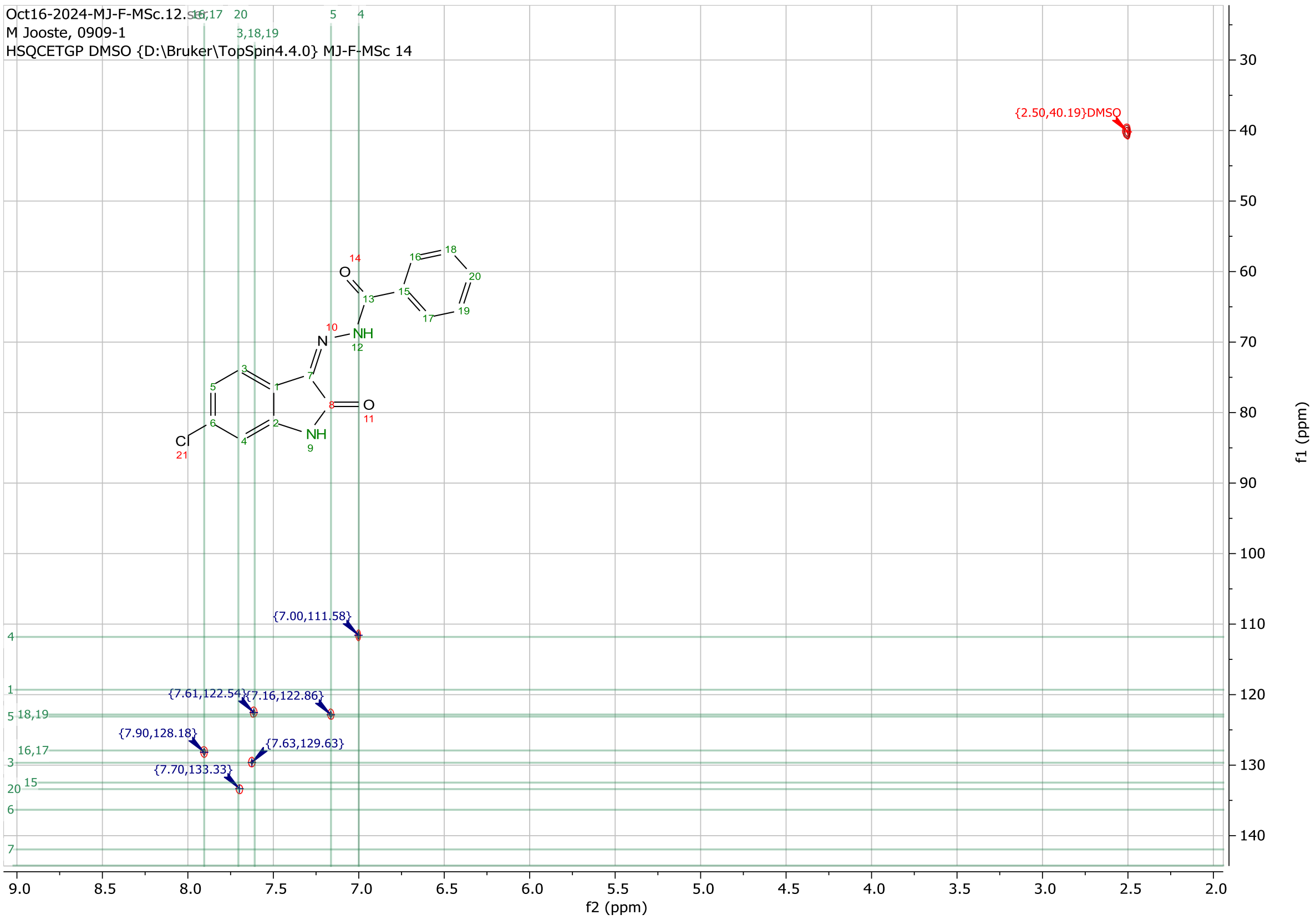

N'-[(3Z)-6-chloro-2-oxo-1,2-dihydro-3H-indol-3-ylidene]-3-hydroxybenzohydrazide (***4b***)

Proton (^{1}H) NMR spectrum

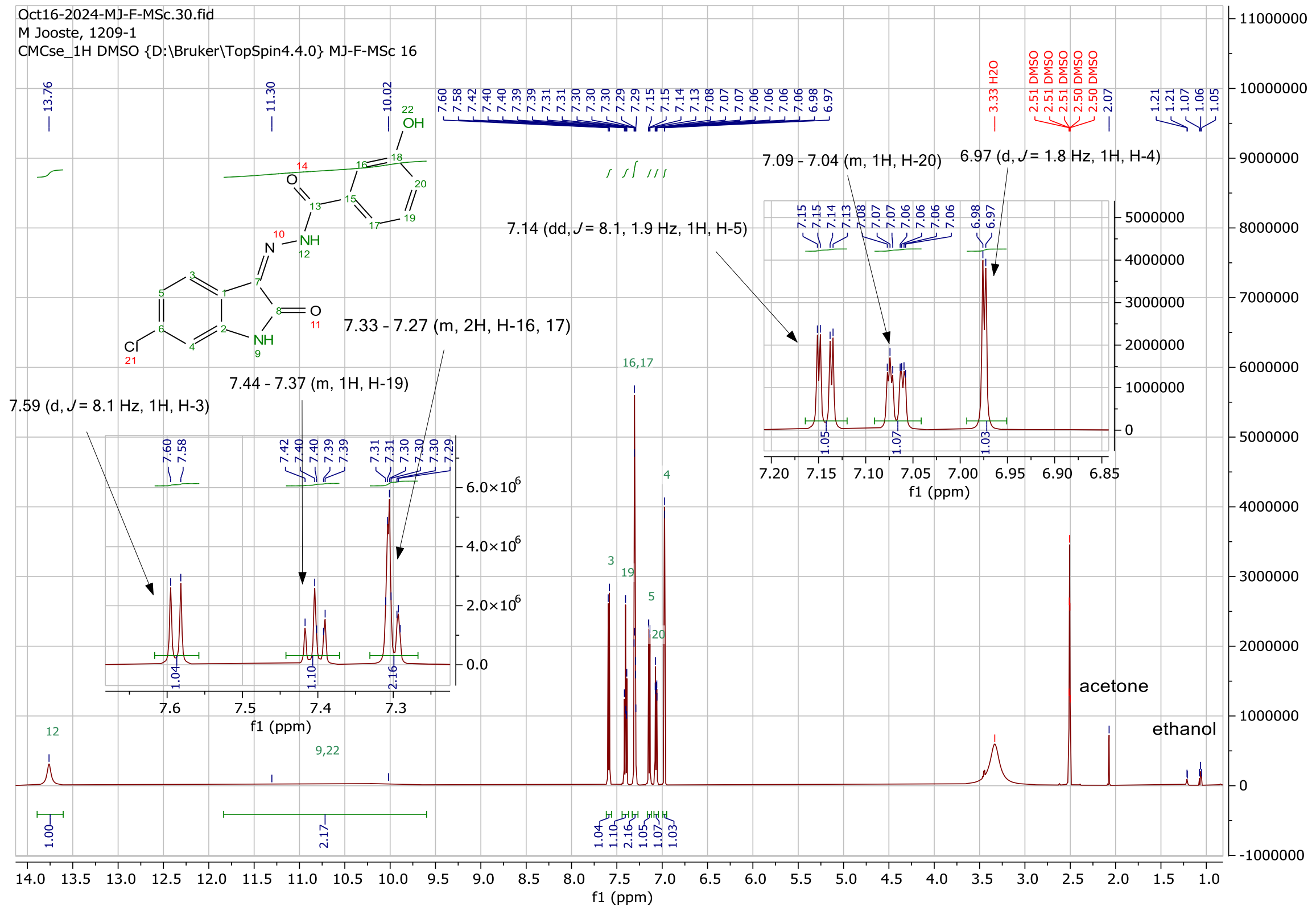

Carbon (^{13}C) NMR spectrum

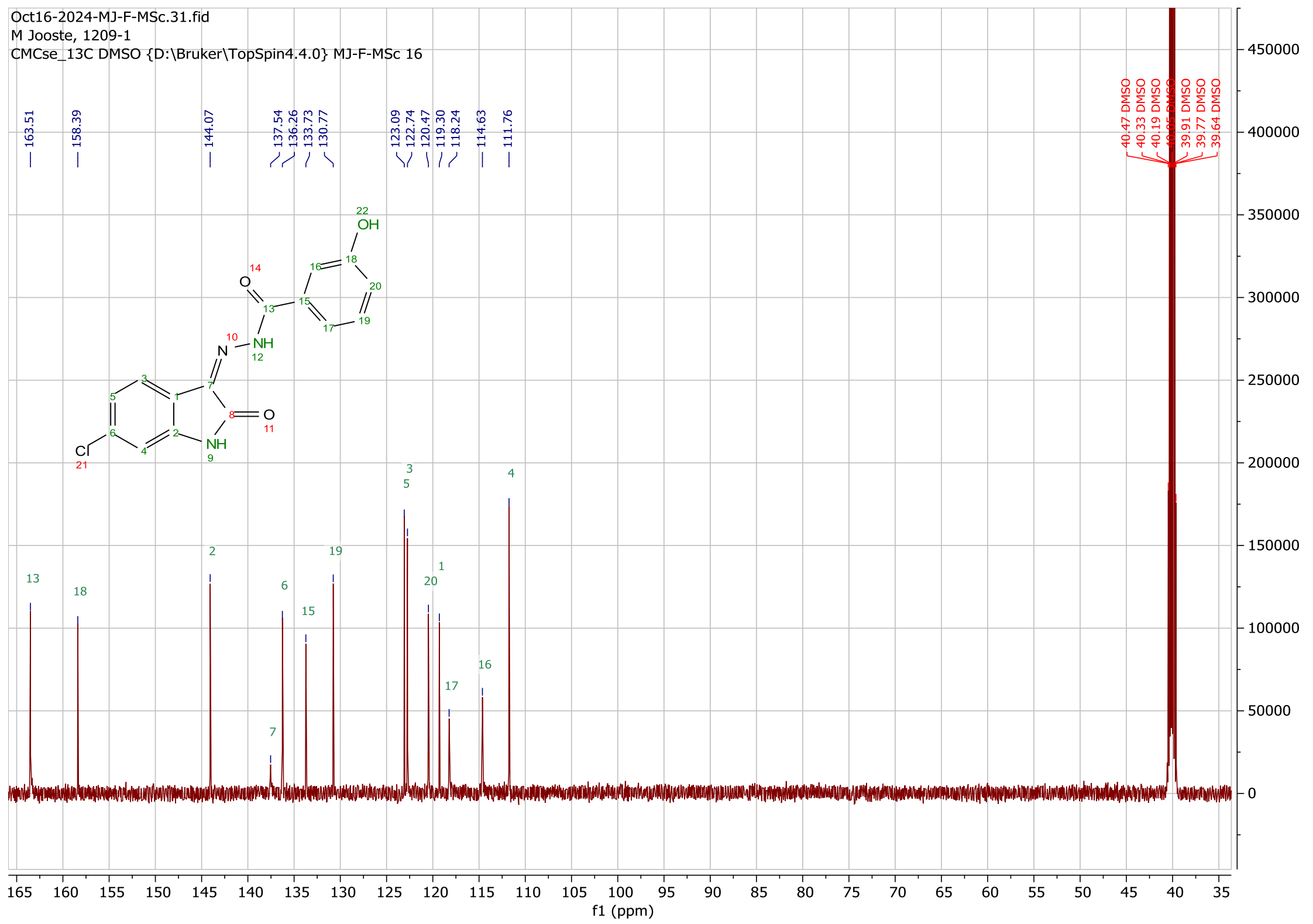

HSQC NMR spectrum

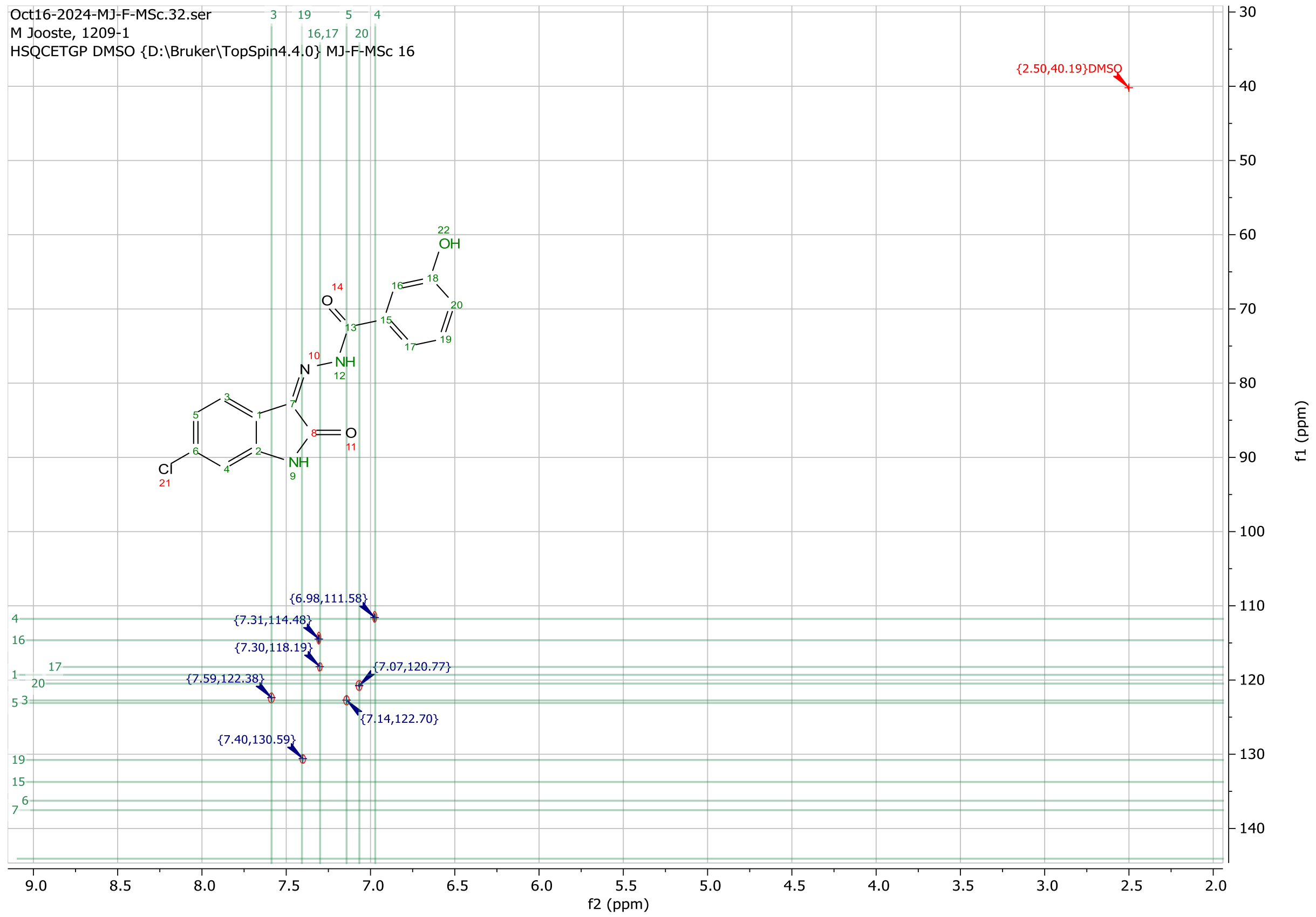

*N'-[(3Z)-7-chloro-2-oxo-1,2-dihydro-3H-indol-3-ylidene]benzohydrazide (**5a**)*

Proton (^{1}H) NMR spectrum

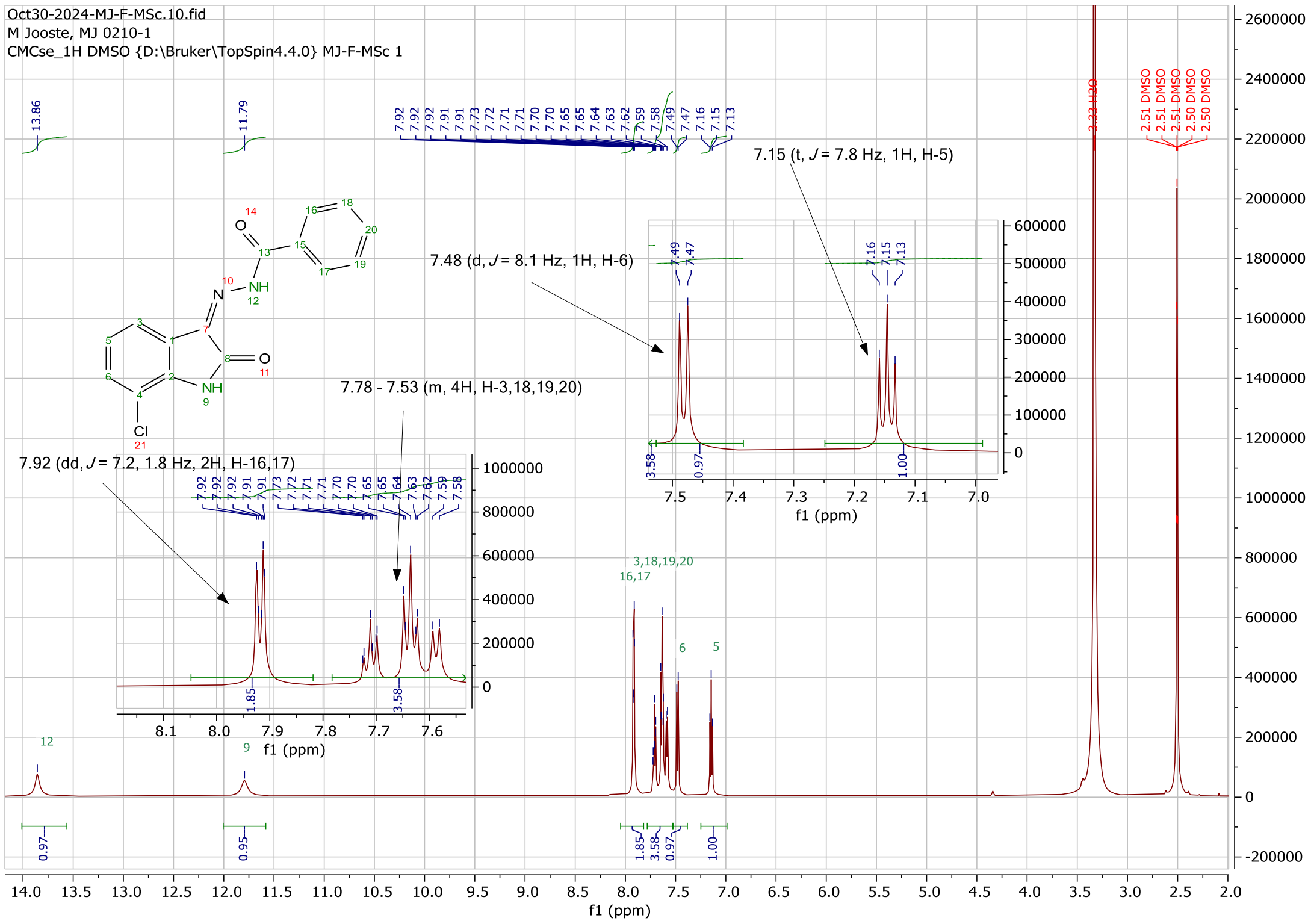

Carbon (^{13}C) NMR spectrum

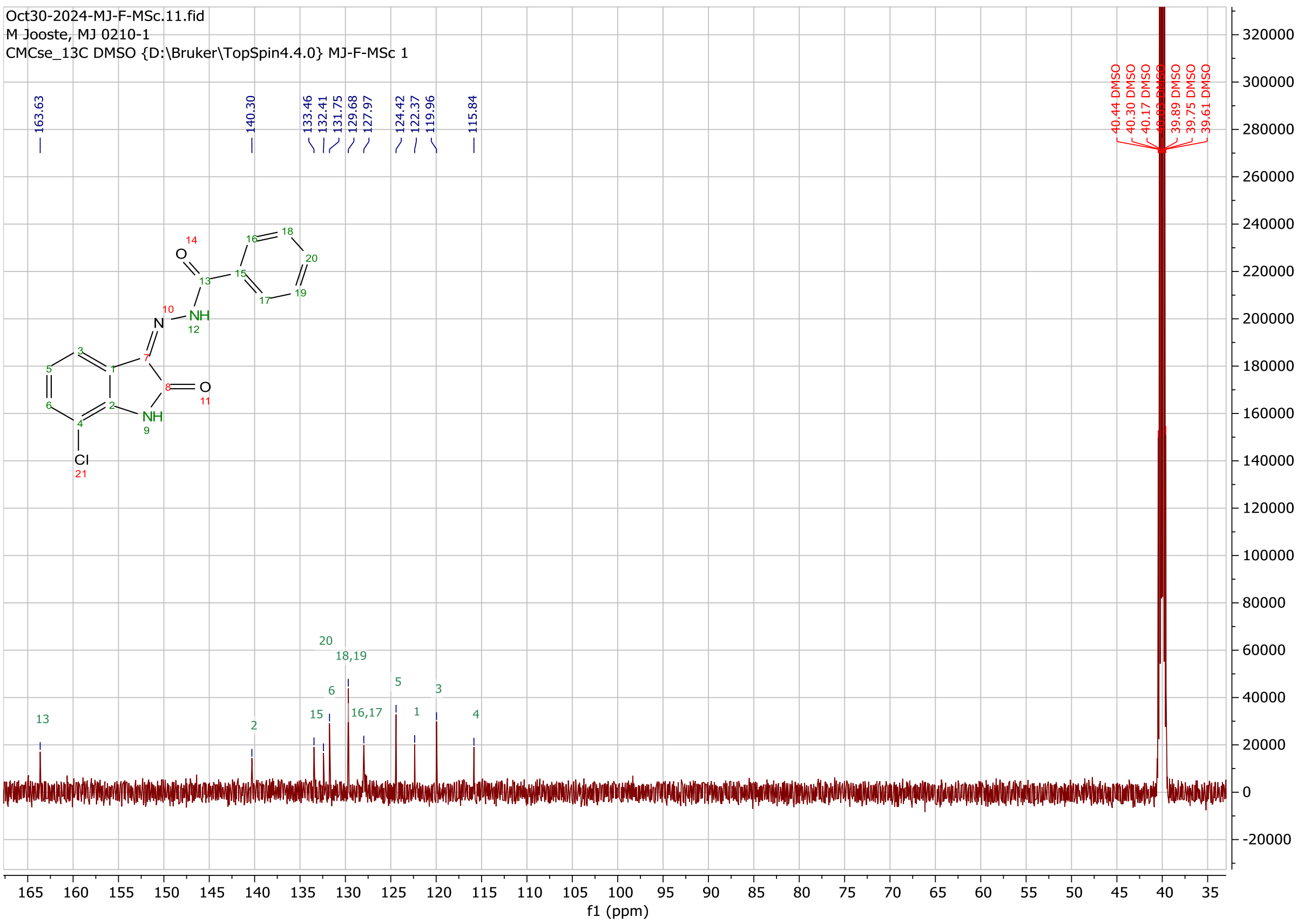

HSQC NMR spectrum

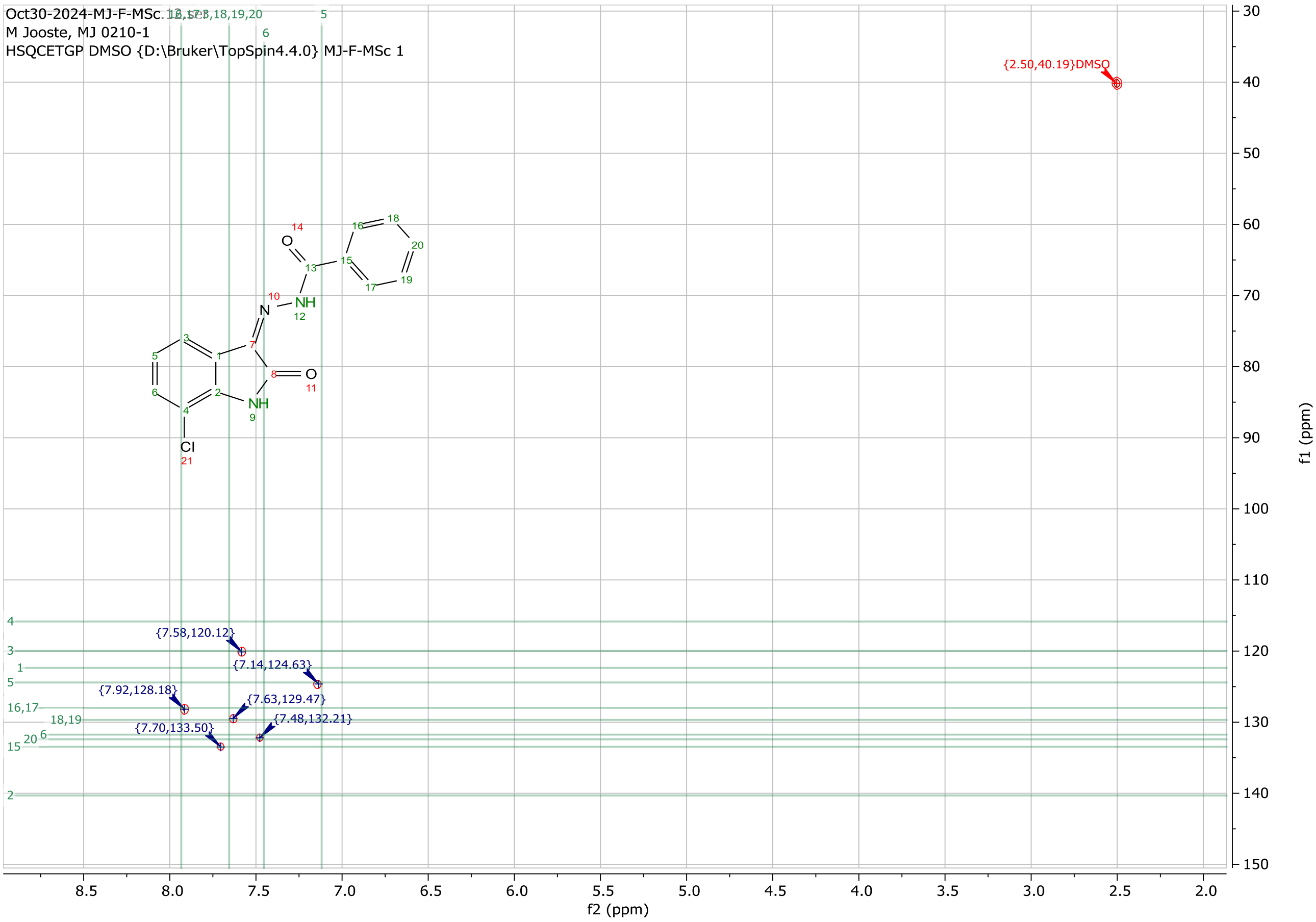

N'-[(3Z)-7-chloro-2-oxo-1,2-dihydro-3H-indol-3-ylidene]-3-hydroxybenzohydrazide

*(**5b**)*

Proton (^{1}H) NMR spectrum

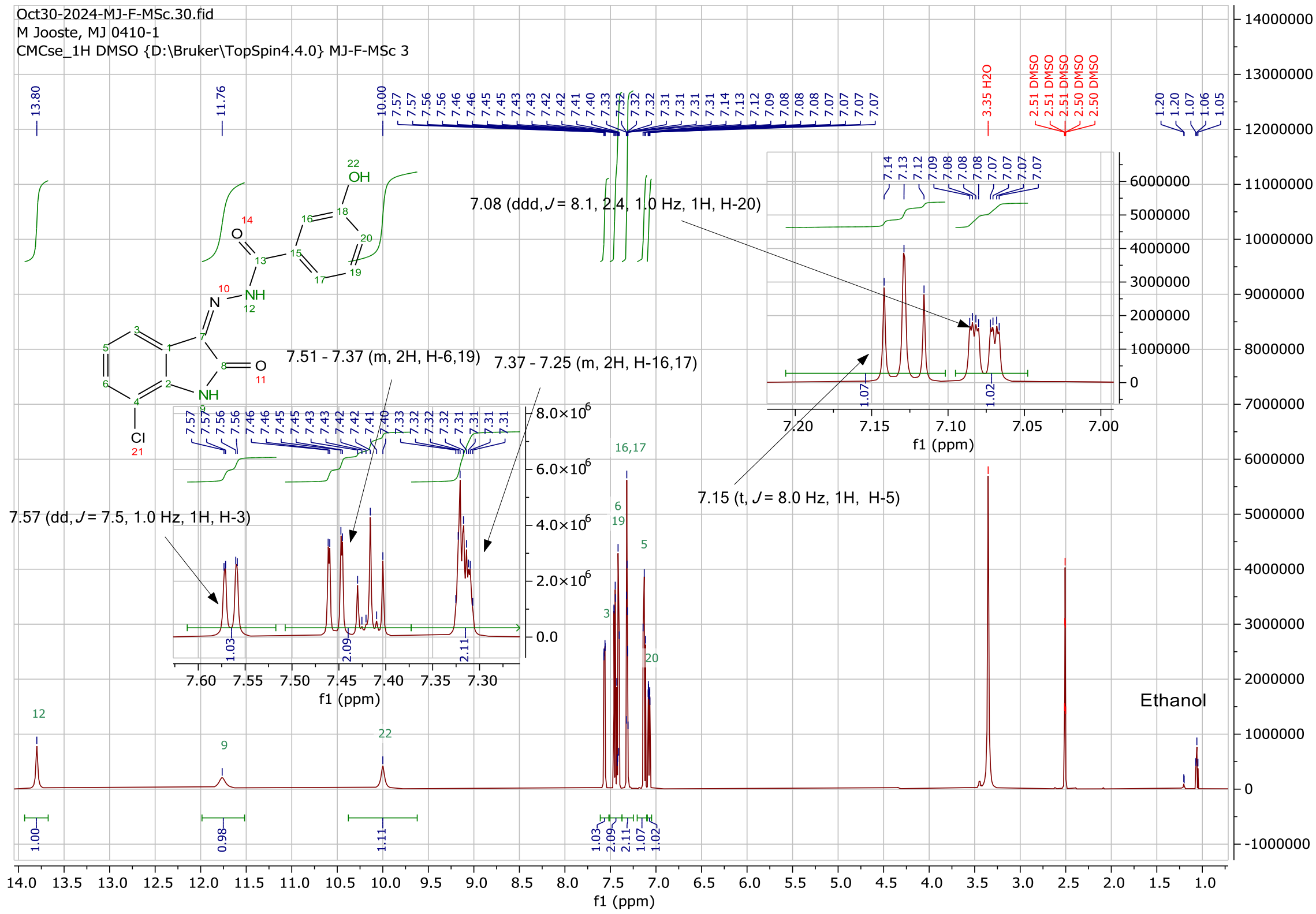

Carbon (^{13}C) NMR spectrum

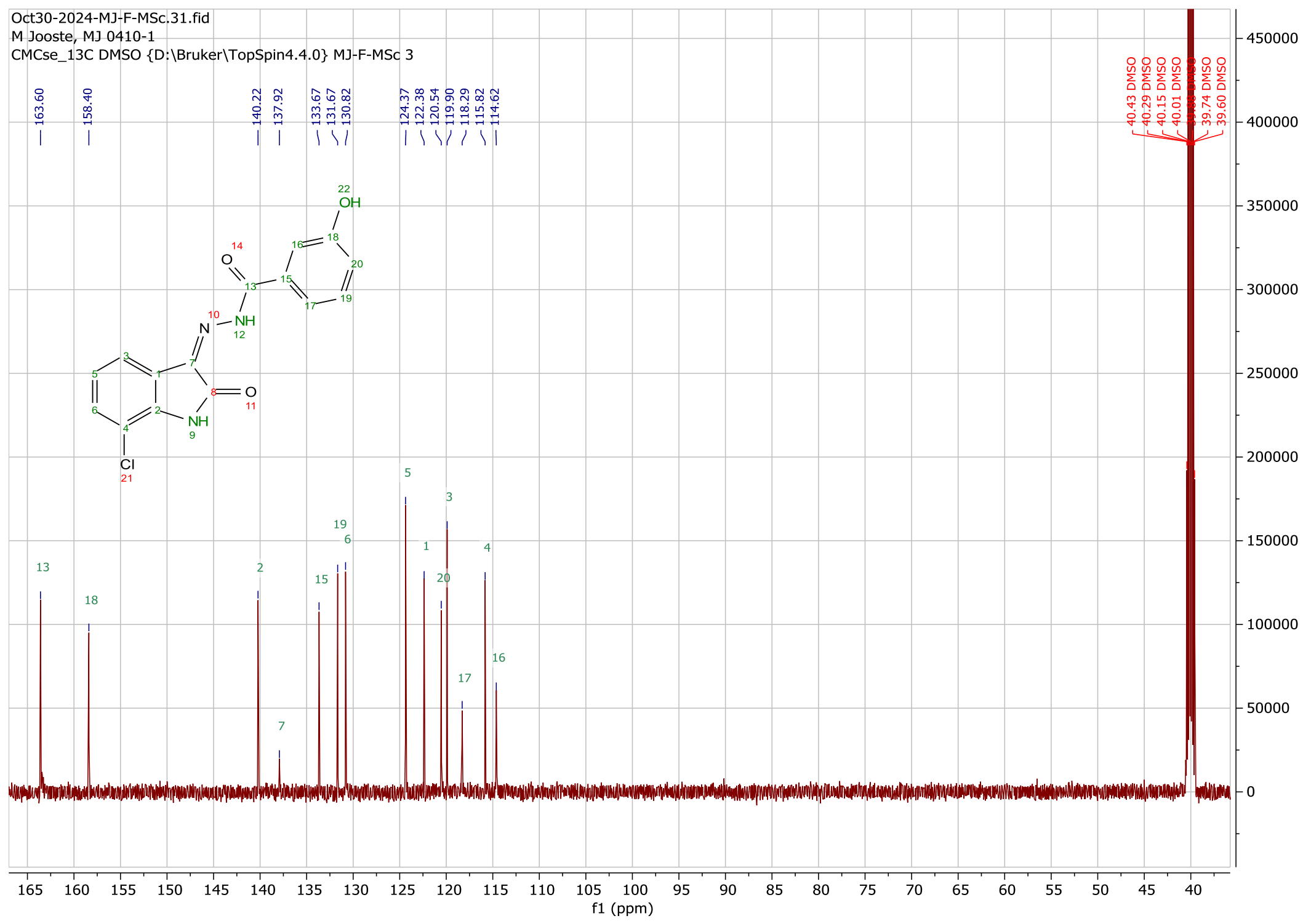

HSQC NMR spectrum

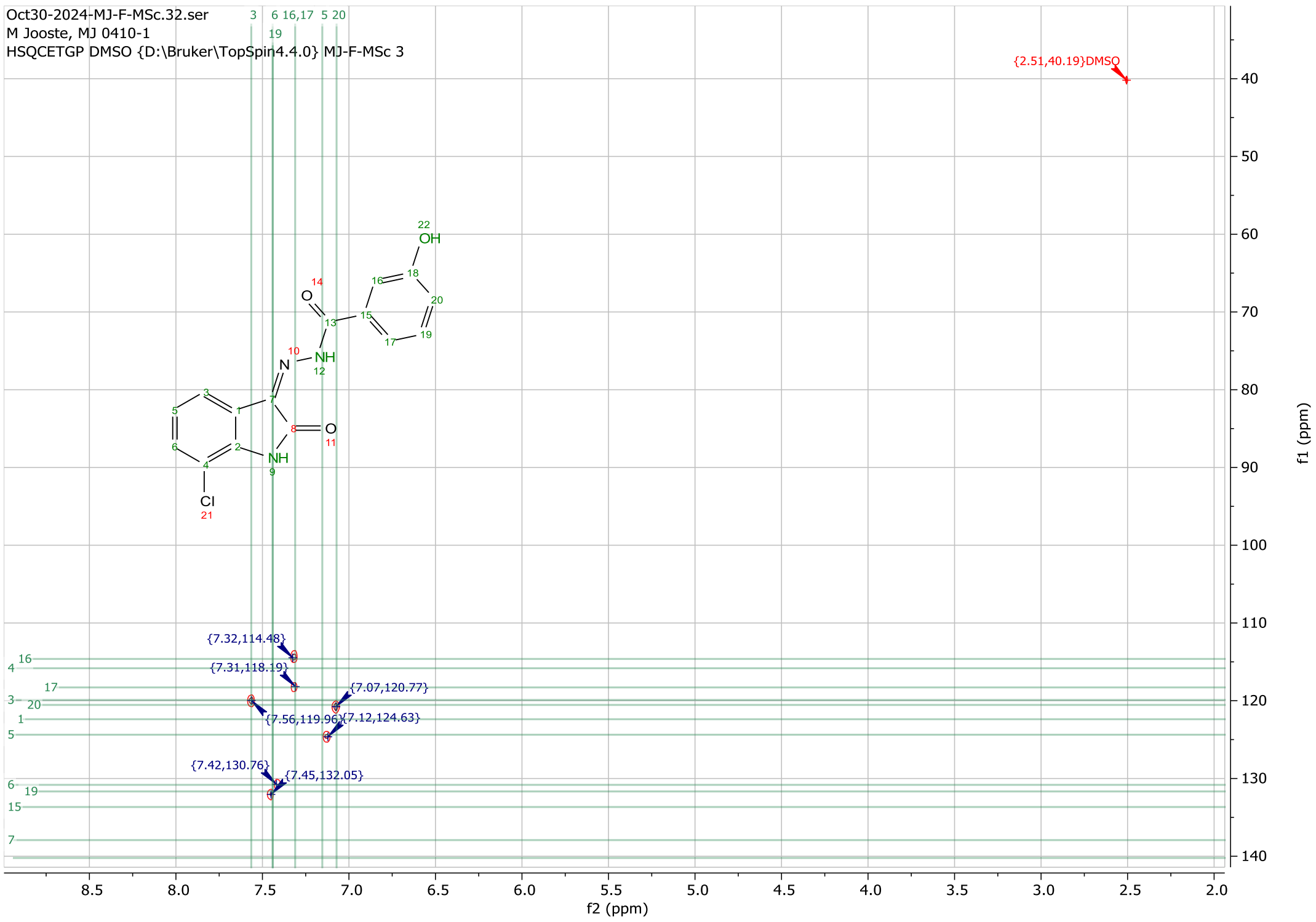

*2-chloro-N'-[(3Z)-7-chloro-2-oxo-1,2-dihydro-3H-indol-3-ylidene]benzohydrazide (**5c**)*

Proton (^{1}H) NMR spectrum

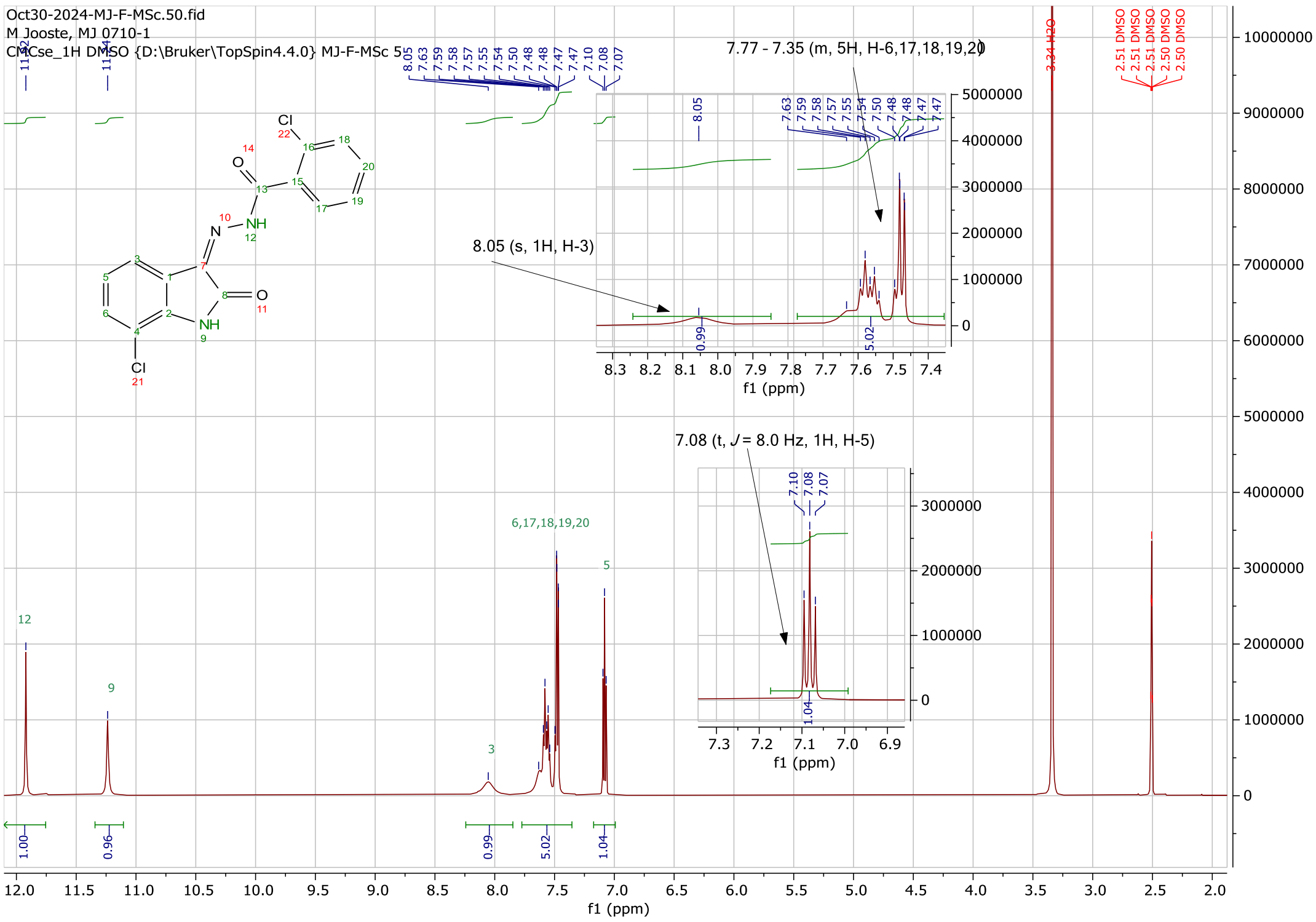

Carbon (^{13}C) NMR spectrum

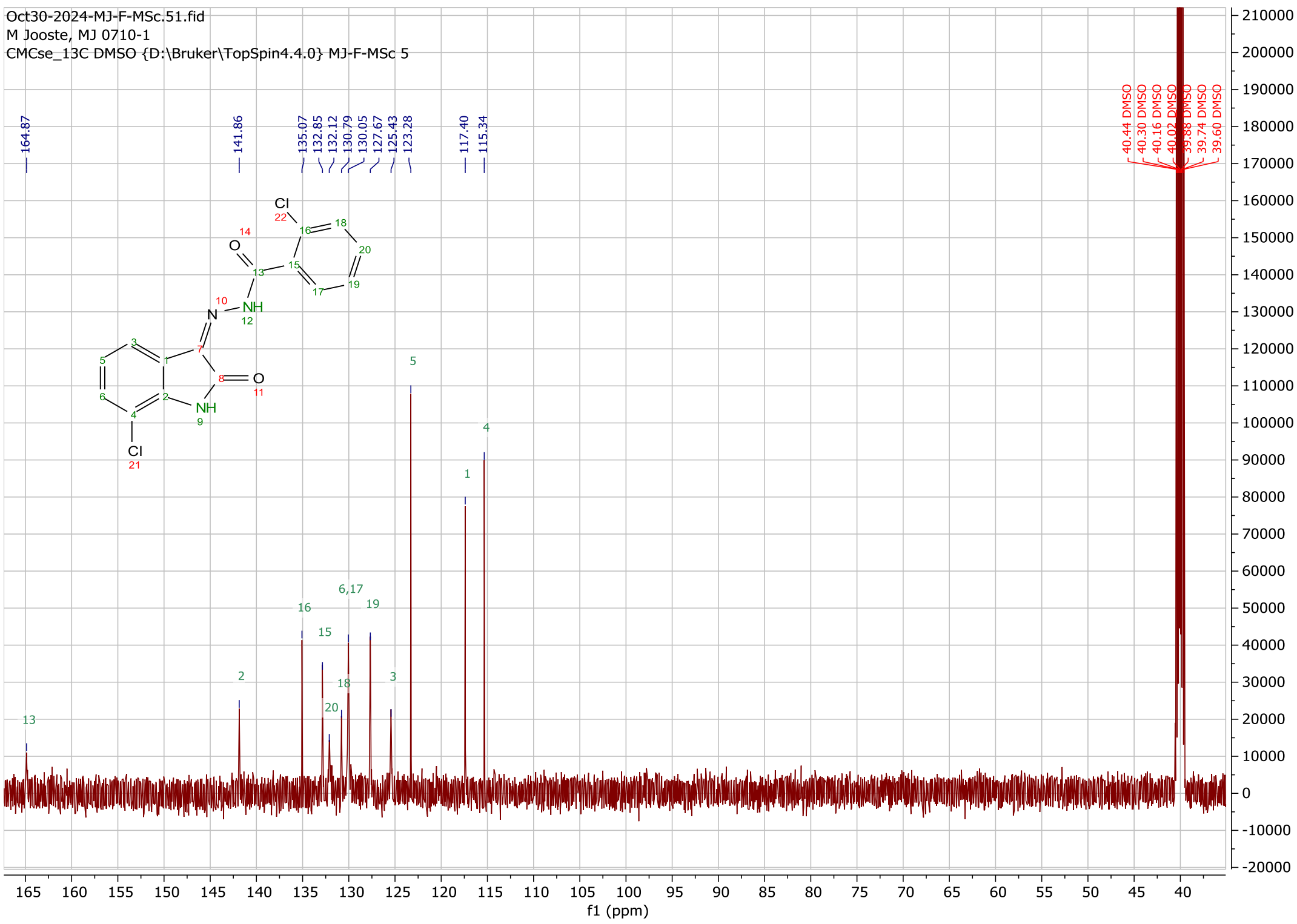

HSQC NMR spectrum

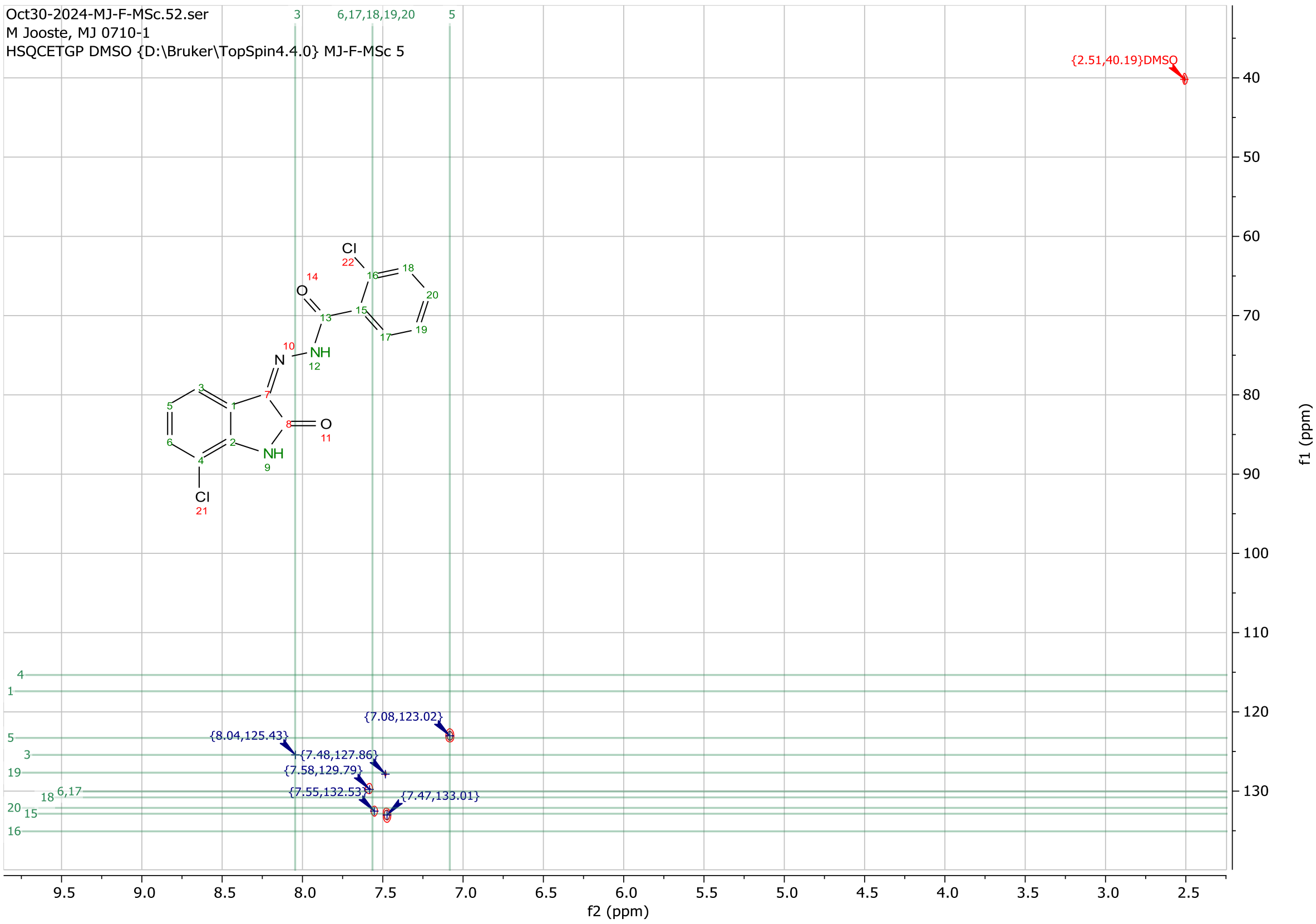

3-chloro-N'-[(3Z)-7-chloro-2-oxo-1,2-dihydro-3H-indol-3-ylidene]benzohydrazide

(5d)

Proton (^{1}H) NMR spectrum

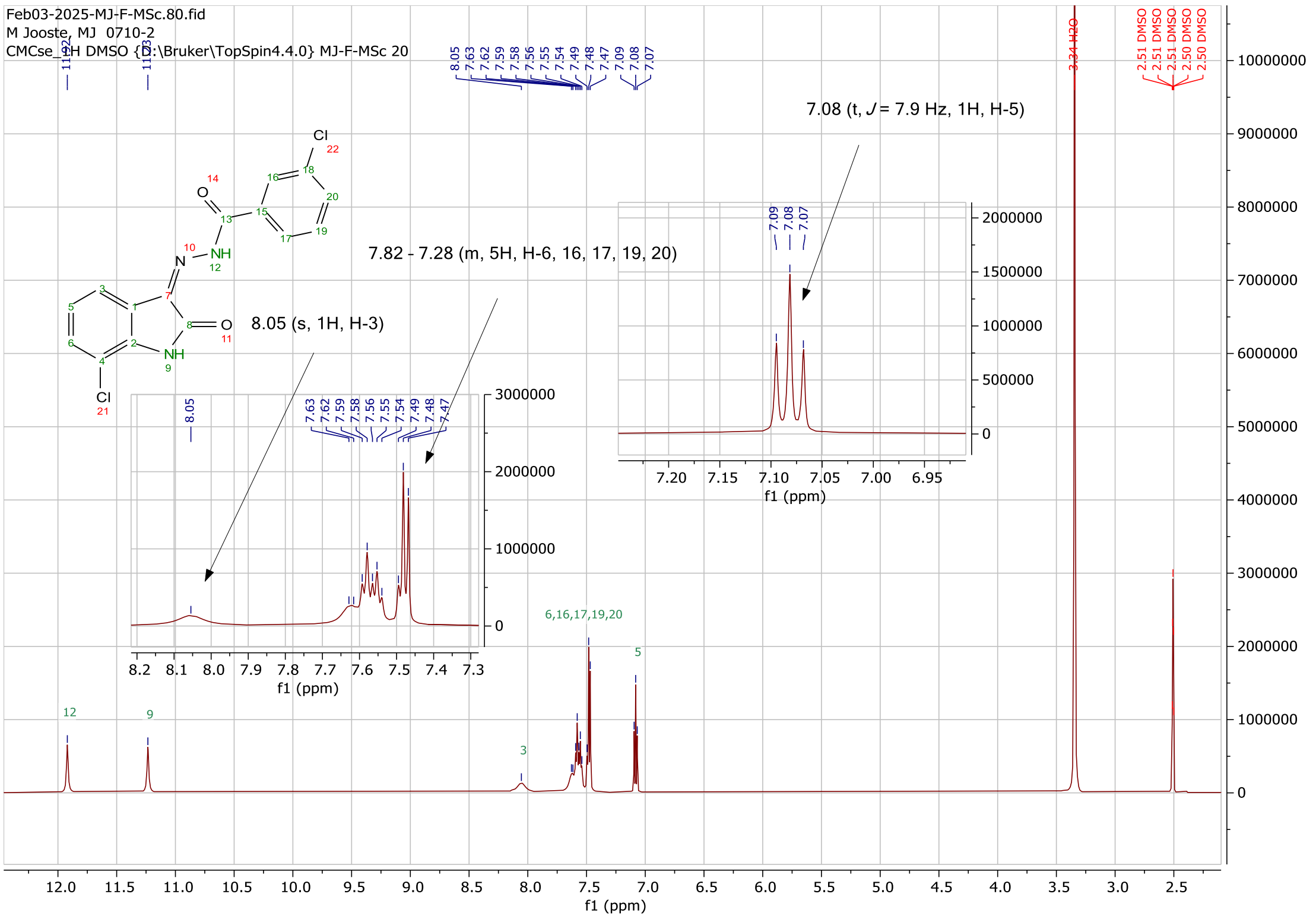

Carbon (^{13}C) NMR spectrum

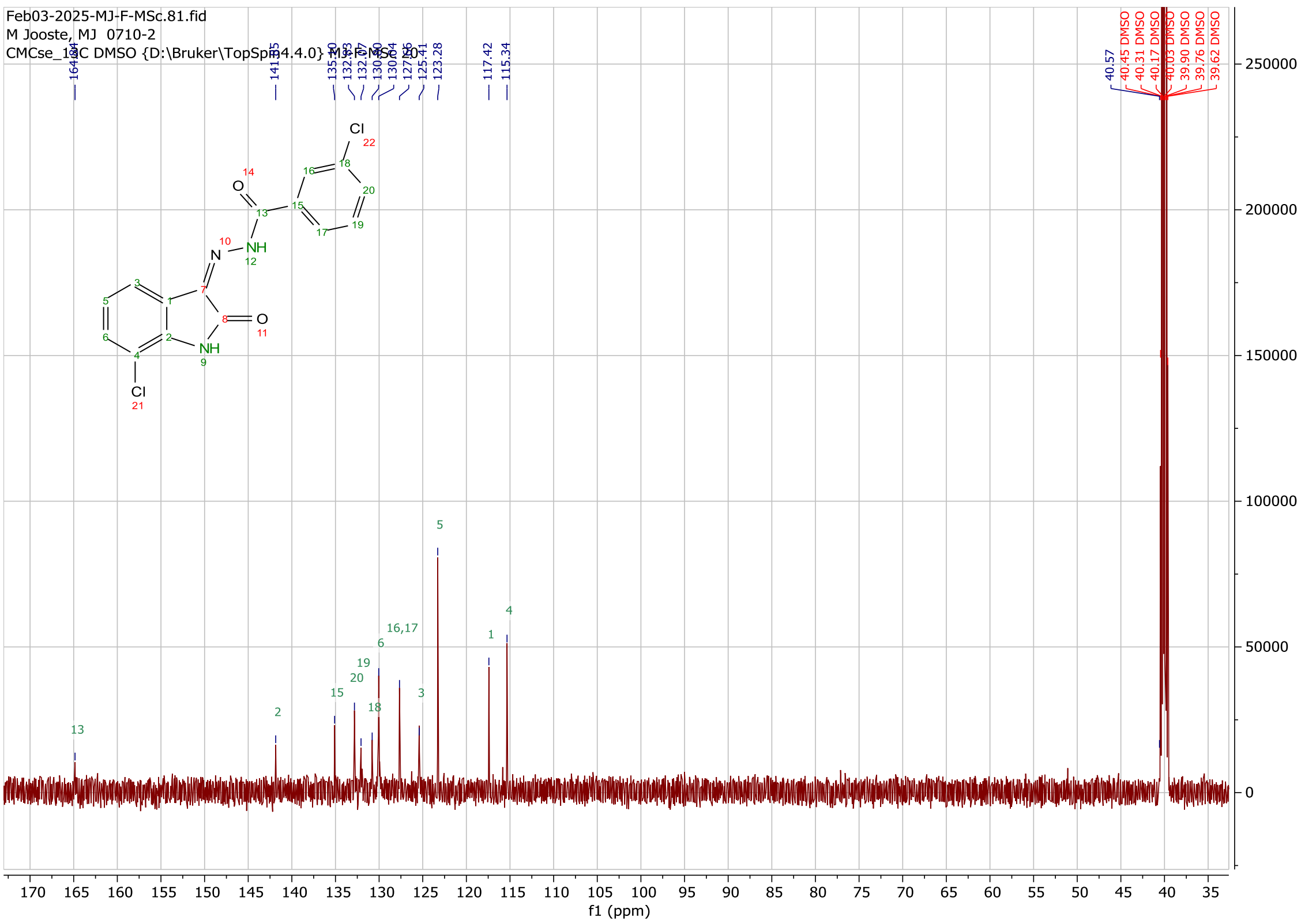

HSQC NMR spectrum

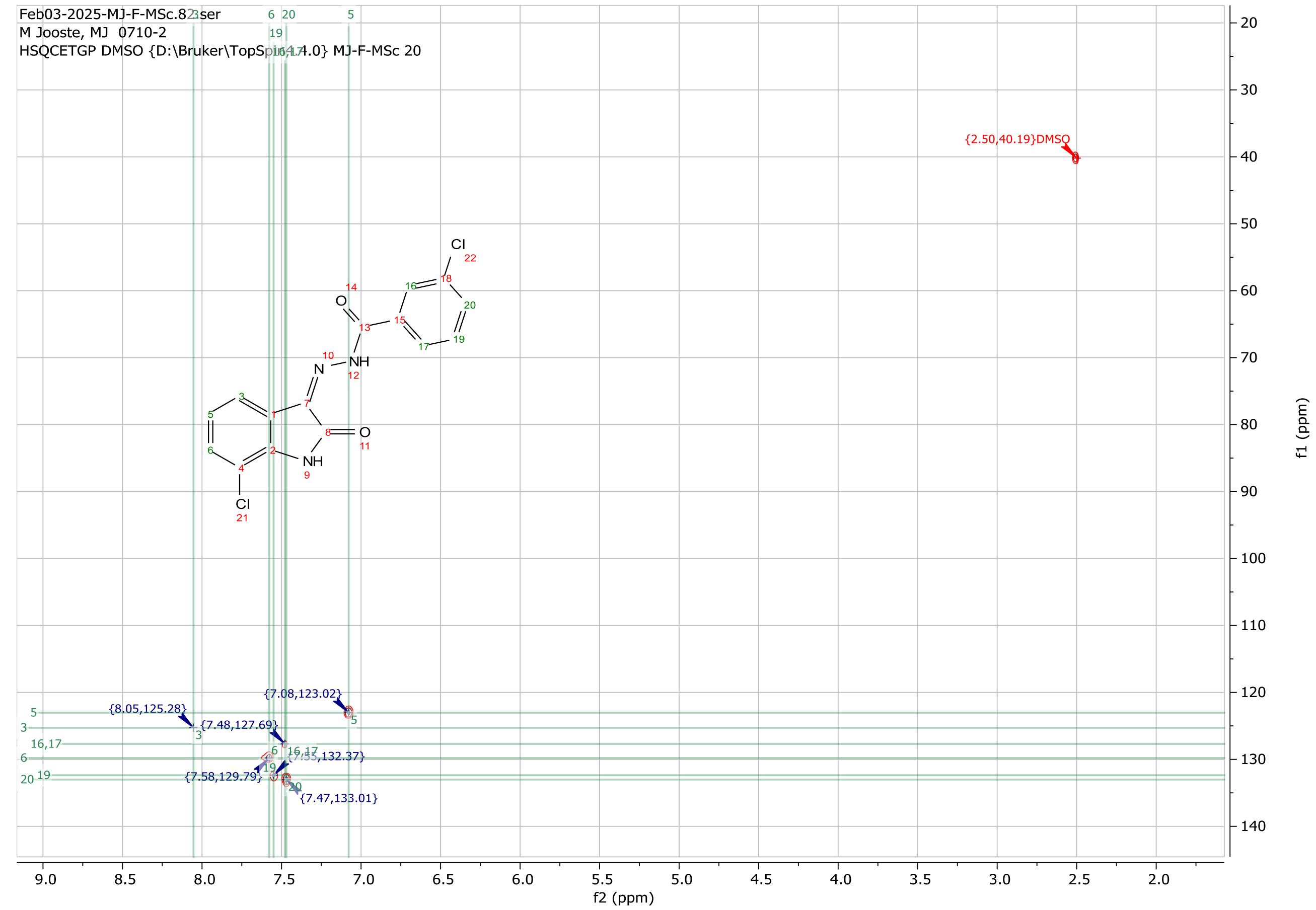

*4-chloro-N'-[(3Z)-7-chloro-2-oxo-1,2-dihydro-3H-indol-3-ylidene]benzohydrazide (**5e**)*

Proton (^{1}H) NMR spectrum

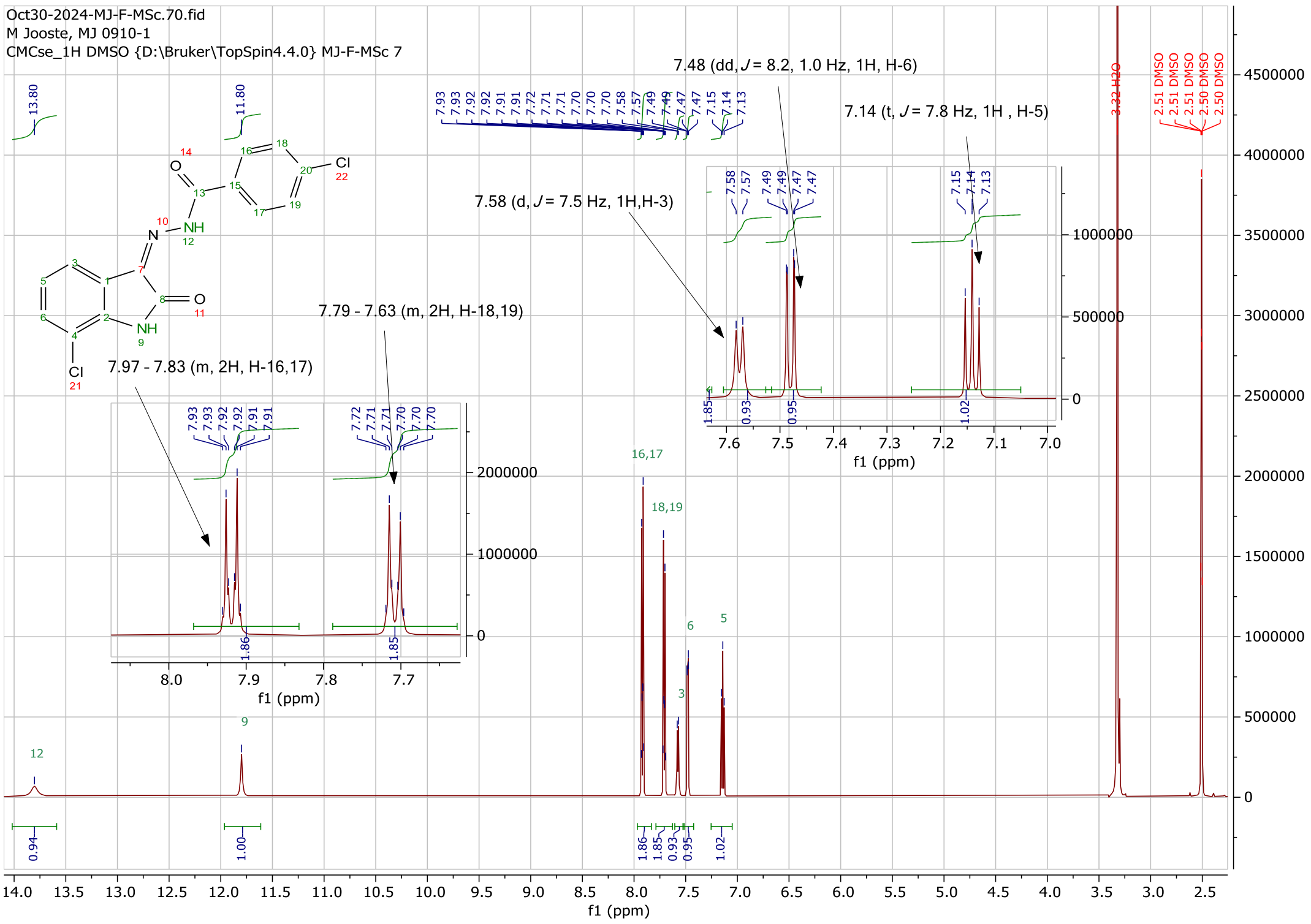

Carbon (^{13}C) NMR spectrum

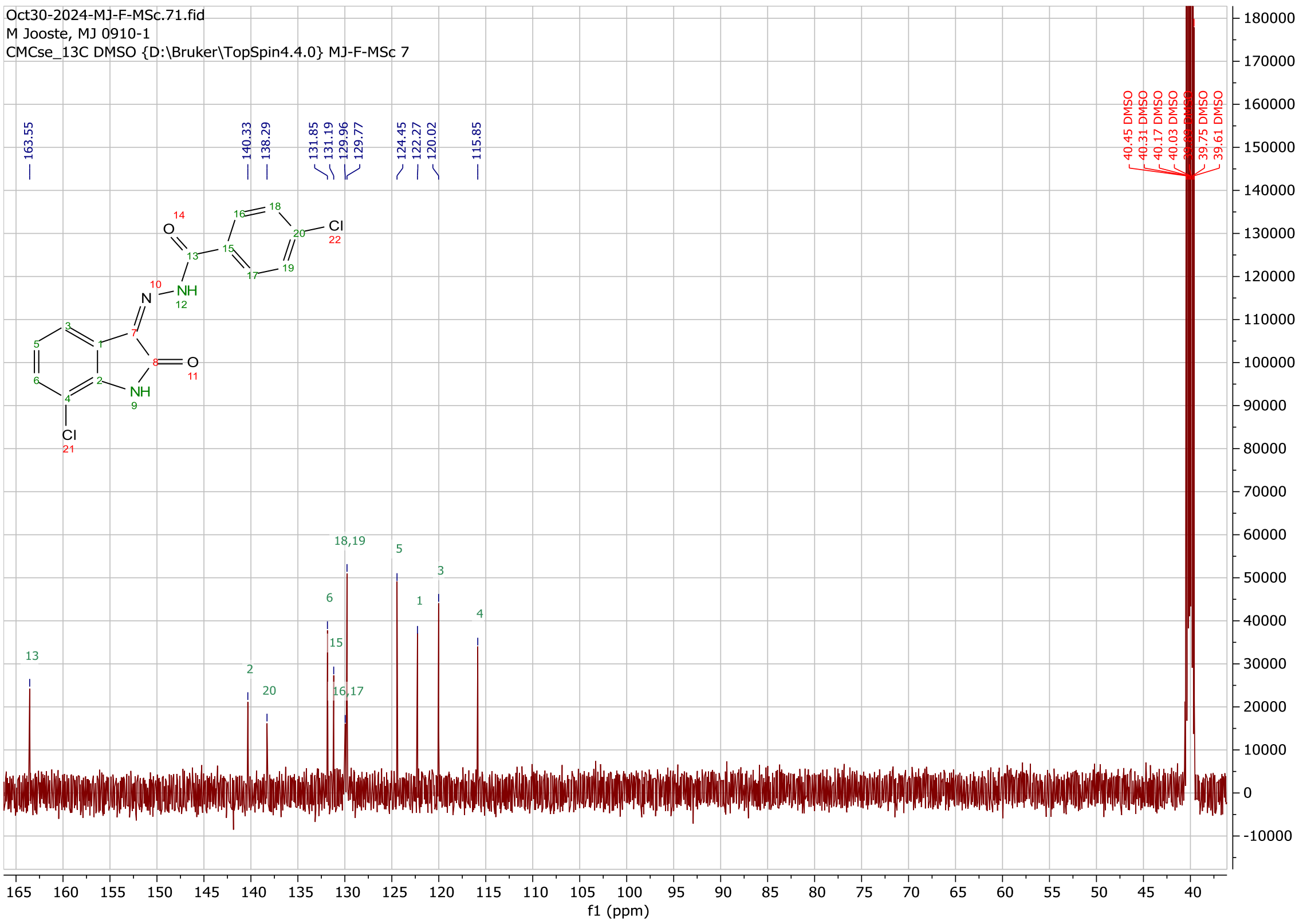

HSQC NMR spectrum

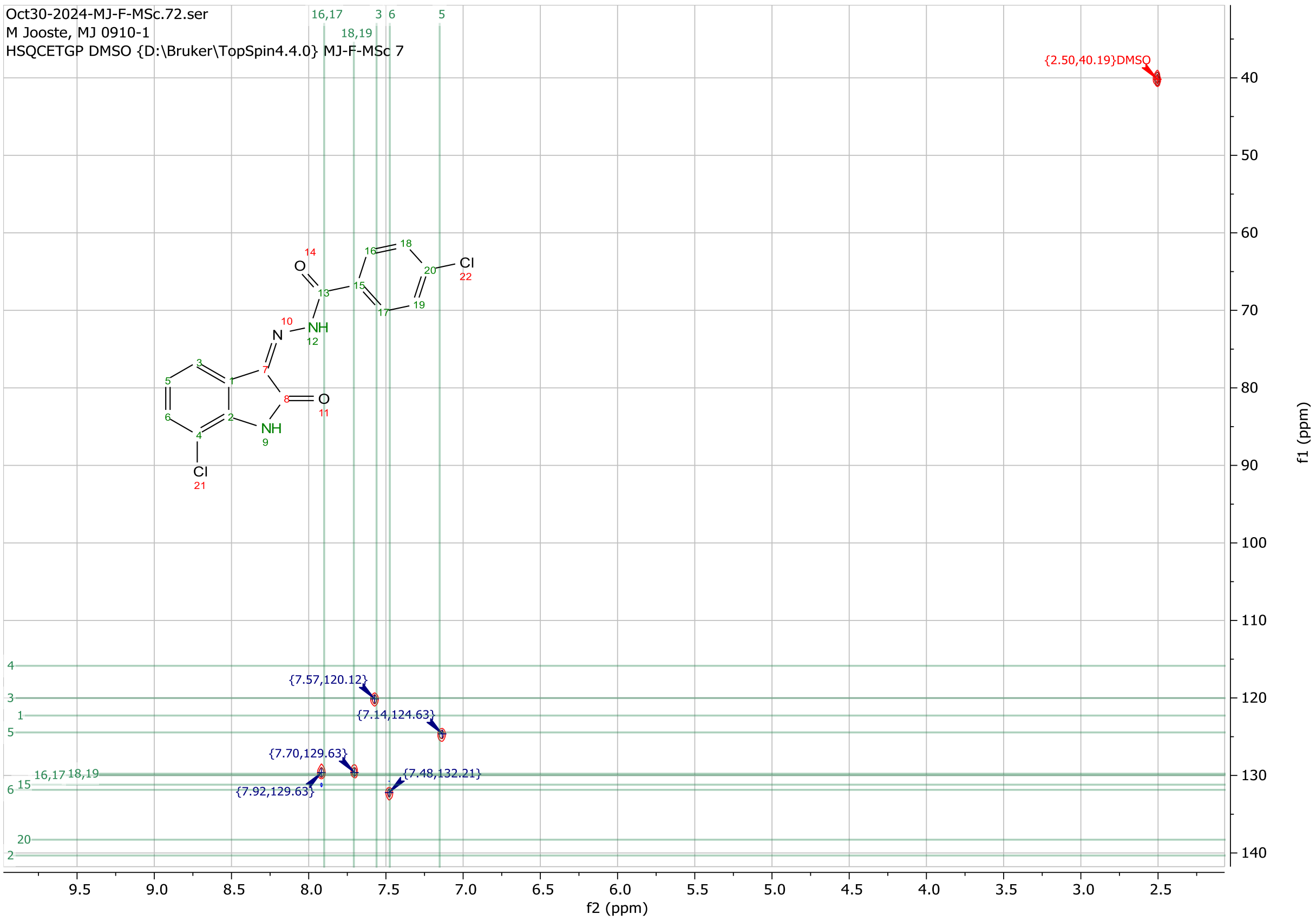

High resolution mass spectrometry (HRMS) spectra

N'-[(3Z)-2-oxo-1,2-dihydro-3H-indol-3-ylidene]benzohydrazide (**1a**)

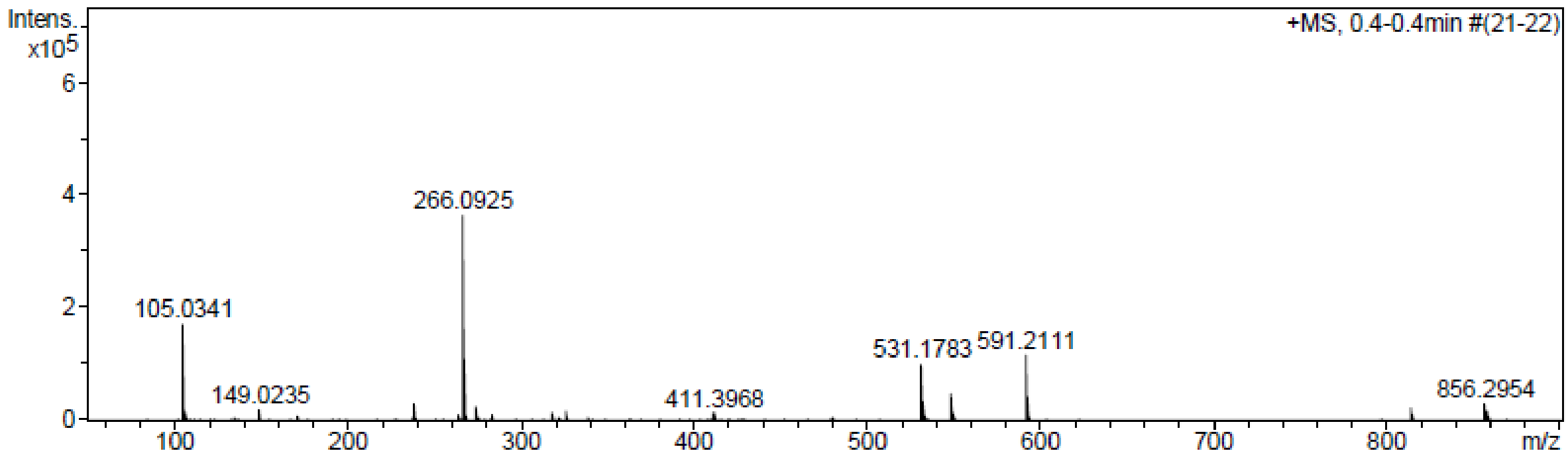

Meas. m/z	#	Formula	Score	m/z	err [mDa]	err [ppm]	mSigma	rdb	e⁻ Conf	N-Rule
105.0341	1	C 7 H 5 O	100.00	105.0335	-0.6	-5.6	0.1	5.5	even	ok
	2	C 5 H 3 N 3	39.32	105.0321	-1.9	-18.3	6.1	6.0	odd	ok
266.0925	1	C 15 H 12 N 3 O 2	100.00	266.0924	-0.1	-0.4	77.3	11.5	even	ok

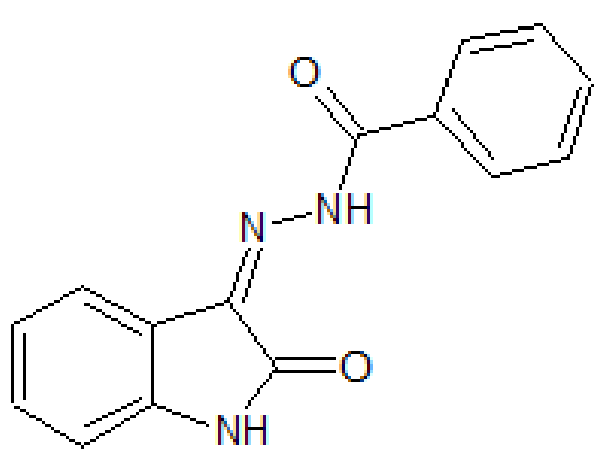

Molecular Formula: $C_{15}H_{11}N_3O_2$
Formula Weight: 265.272
Composition: C(67.92%) H(4.18%) N(15.84%) O(12.06%)
Nominal Mass: 265 Da
Average Mass: 265.272 Da
[M+H]+: 266.092403 Da
[M+H]-: 266.0935 Da

3-hydroxy-N'-[(3Z)-2-oxo-1,2-dihydro-3H-indol-3-ylidene]benzohydrazide (**1b**)

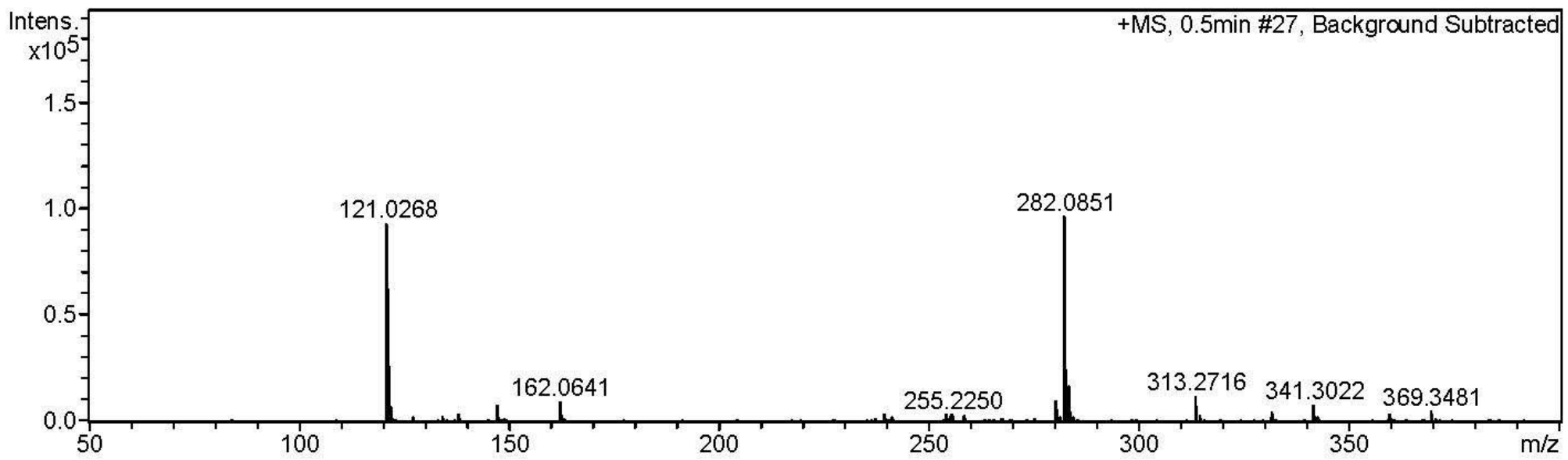

Meas. m/z	#	Formula	Score	m/z	err [mDa]	err [ppm]	mSigma	rdb	e⁻ Conf	N-Rule
121.0268	1	C 7 H 5 O 2	49.19	121.0284	1.6	13.4	2.5	5.5	even	ok
	2	C 5 H 3 N 3 O	100.00	121.0271	0.3	2.3	5.3	6.0	odd	ok
282.0851	1	C 15 H 12 N 3 O 3	100.00	282.0873	2.2	7.9	0.9	11.5	even	ok

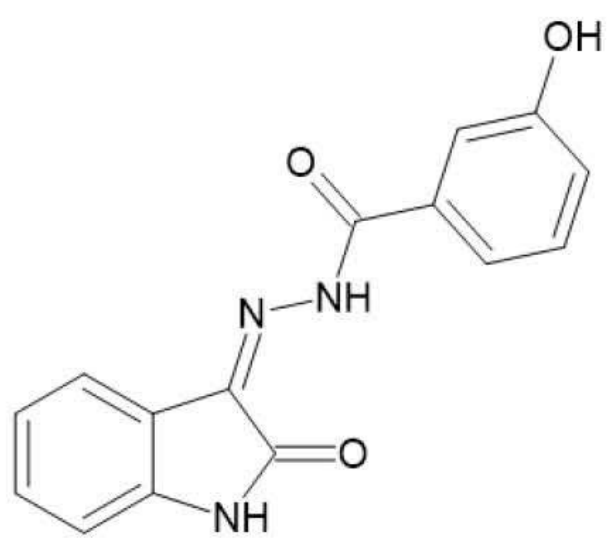

Molecular Formula: $C_{15}H_{11}N_3O_3$
Formula Weight: 281.271
Composition: C(64.05%) H(3.94%) N(14.94%) O(17.06%)
Nominal Mass: 281 Da
Average Mass: 281.271 Da
[M+H]+: 282.087318 Da
[M+H]-: 282.088415 Da

4-hydroxy-N'-[(3Z)-2-oxo-1,2-dihydro-3H-indol-3-ylidene]benzohydrazide (**1c**)

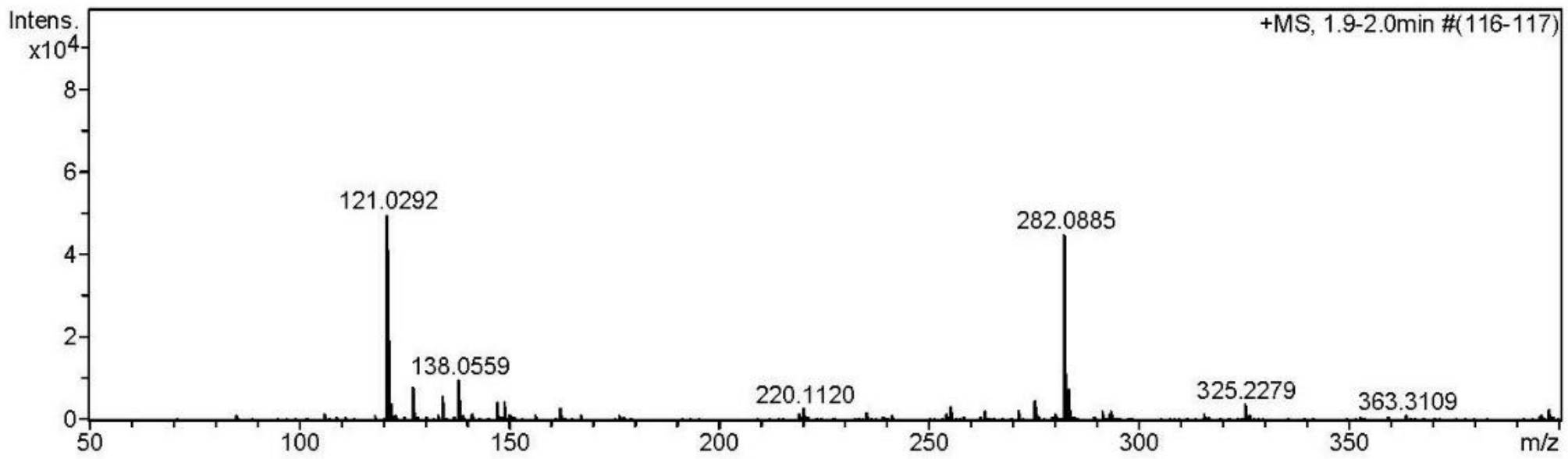

Meas. m/z	#	Formula	Score	m/z	err [mDa]	err [ppm]	mSigma	rdb	e⁻ Conf	N-Rule
121.0292	1	C 7 H 5 O 2	100.00	121.0284	-0.8	-6.5	2.6	5.5	even	ok
	2	C 5 H 3 N 3 O	38.30	121.0271	-2.1	-17.6	7.0	6.0	odd	ok
282.0885	1	C 15 H 12 N 3 O 3	100.00	282.0873	-1.1	-4.0	2.2	11.5	even	ok

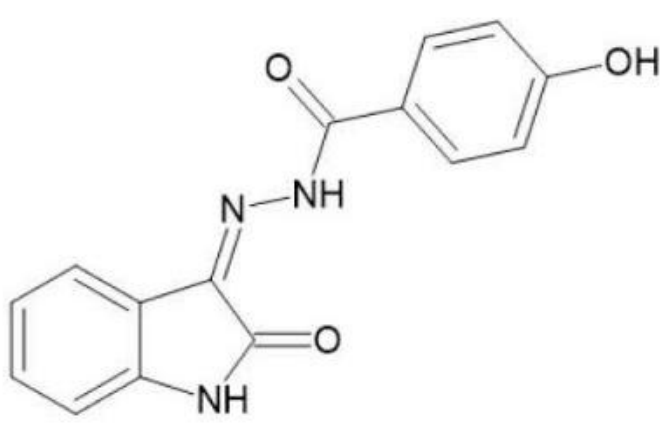

Molecular Formula: $C_{15}H_{11}N_3O_3$
Formula Weight: 281.271
Composition: C(64.05%) H(3.94%) N(14.94%) O(17.06%)
Nominal Mass: 281 Da
Average Mass: 281.271 Da
[M+H]+: 282.087318 Da
[M+H]-: 282.088415 Da

3-chloro-N'-[(3Z)-2-oxo-1,2-dihydro-3H-indol-3-ylidene]benzohydrazide (**1d**)

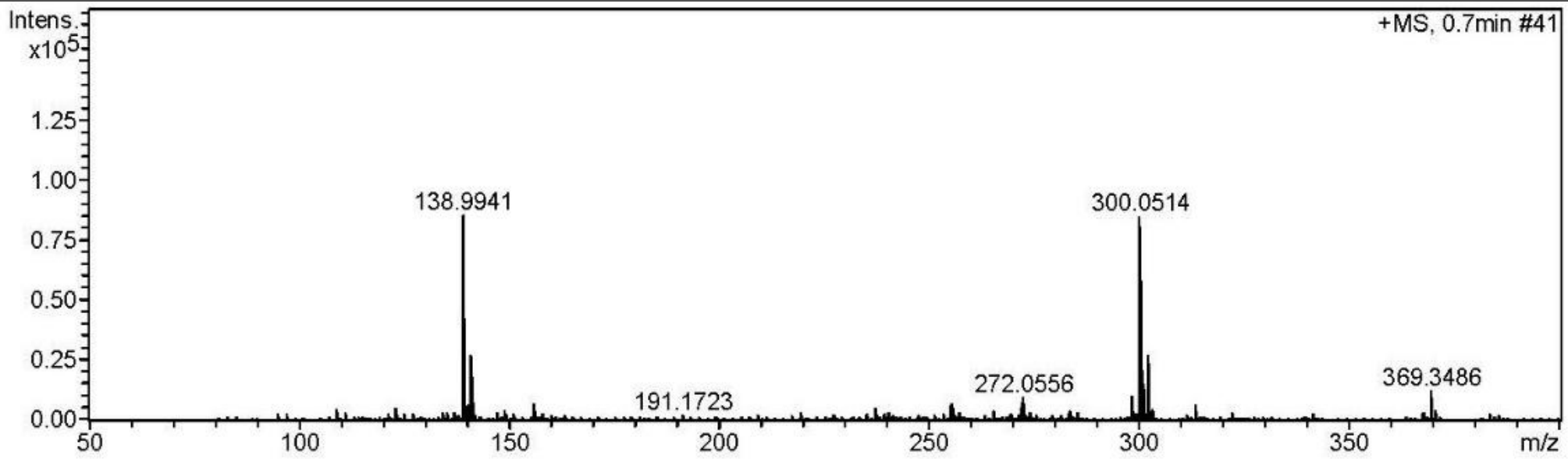

Meas. m/z	#	Formula	Score	m/z	err [mDa]	err [ppm]	mSigma	rdb	e⁻ Conf	N-Rule
138.9941	1	C 5 H 2 Cl N 3	76.98	138.9932	-0.9	-6.8	11.3	6.0	odd	ok
	2	C 7 H 4 Cl O	100.00	138.9945	0.4	2.9	12.3	5.5	even	ok
	3	C 2 H 4 Cl N 3 O 2	0.00	136.9987	1.7	12.1	604.6	2.0	odd	ok
	4	C 7 H 3 Cl N	0.00	135.9949	0.9	6.3	717.4	6.5	even	ok
300.0514	1	C 15 H 11 Cl N 3 O 2	100.00	300.0534	2.1	6.9	9.4	11.5	even	ok
	2	C 15 H 10 Cl N 3 O 2	0.00	299.0456	-2.8	-9.3	588.2	12.0	odd	ok

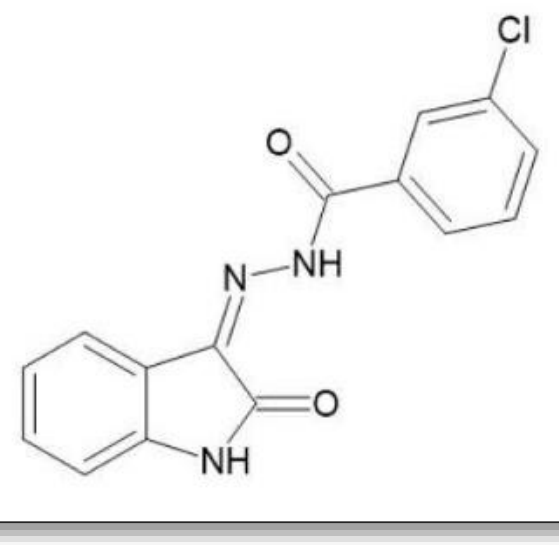

Molecular Formula: $C_{15}H_{10}ClN_3O_2$
Formula Weight: 299.714
Composition: C(60.11%) H(3.36%) Cl(11.83%) N(14.02%) O(10.68%)
Nominal Mass: 299 Da
Average Mass: 299.714 Da
[M+H]+: 300.053431 Da
[M+H]-: 300.054528 Da

4-chloro-N'-[(3Z)-2-oxo-1,2-dihydro-3H-indol-3-ylidene]benzohydrazide (**1e**)

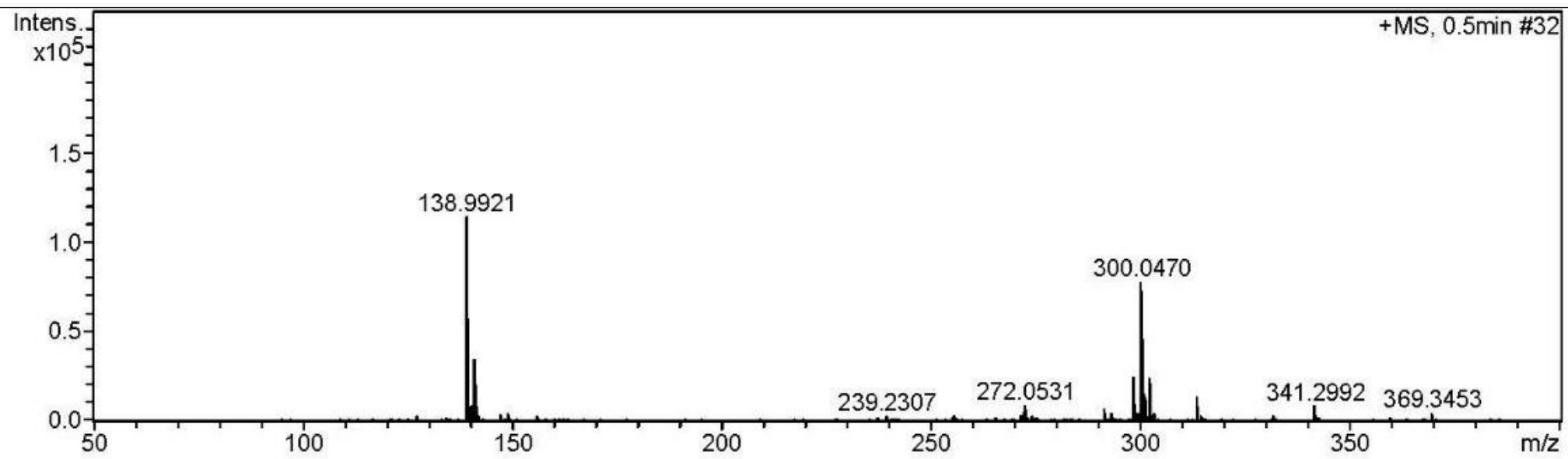

Meas. m/z	#	Formula	Score	m/z	err [mDa]	err [ppm]	mSigma	rdb	e⁻ Conf	N-Rule
138.9921	1	C 7 H 4 Cl O	39.57	138.9945	2.4	17.3	8.1	5.5	even	ok
	2	C 5 H 2 Cl N 3	100.00	138.9932	1.1	7.6	9.6	6.0	odd	ok
	3	C 2 H 3 Cl N 3 O 2	0.00	135.9908	-2.8	-20.2	162.9	2.5	even	ok
	4	C 2 H 4 Cl N 3 O 2	0.00	136.9987	3.7	26.5	162.9	2.0	odd	ok
	5	C 7 H 3 Cl O	0.00	137.9867	-2.0	-14.7	164.7	6.0	odd	ok
	6	C 7 H 3 Cl N	0.00	135.9949	2.9	20.7	165.3	6.5	even	ok
300.0470	1	C 15 H 10 Cl N 3 O 2	100.00	299.0456	1.6	5.4	578.2	12.0	odd	ok

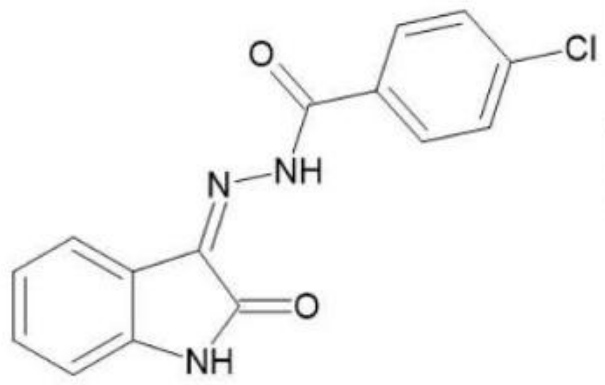

Molecular Formula: $C_{15}H_{10}ClN_3O_2$
Formula Weight: 299.714
Composition: C(60.11%) H(3.36%) Cl(11.83%) N(14.02%) O(10.68%)
Nominal Mass: 299 Da
Average Mass: 299.714 Da
[M+H]+: 300.053431 Da
[M+H]-: 300.054528 Da

N'-[(3Z)-4-chloro-2-oxo-1,2-dihydro-3H-indol-3-ylidene]benzohydrazide (**2a**)

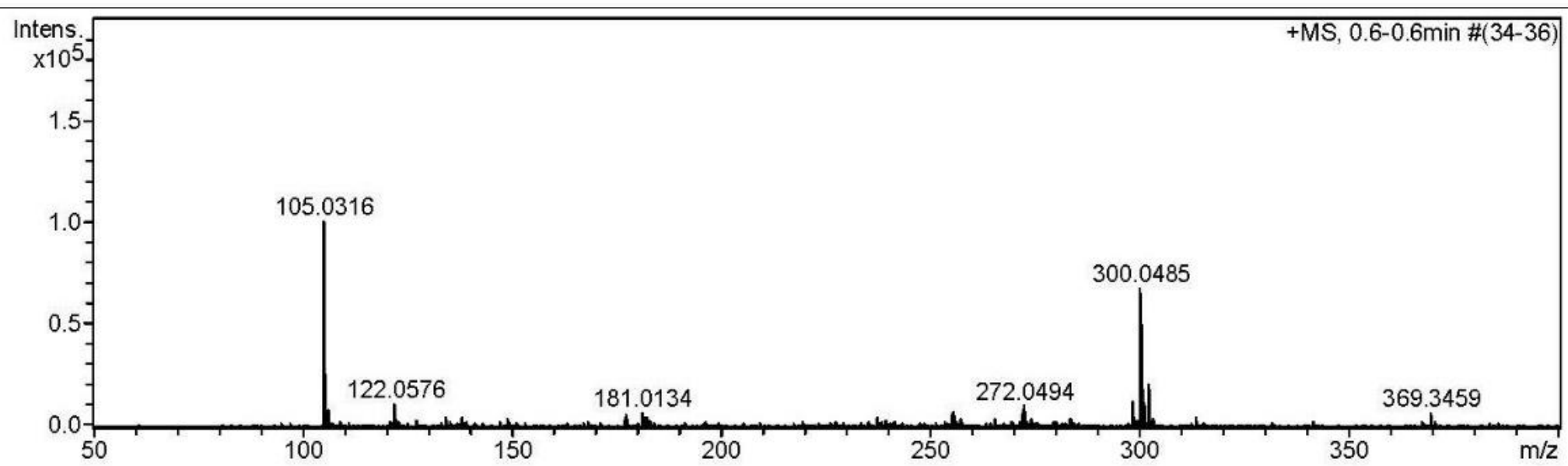

Meas. m/z	#	Formula	Score	m/z	err [mDa]	err [ppm]	mSigma	rdb	e⁻ Conf	N-Rule
105.0316	1	C 7 H 5 O	48.42	105.0335	1.8	17.6	0.5	5.5	even	ok
	2	C 5 H 3 N 3	100.00	105.0321	0.5	4.8	5.6	6.0	odd	ok
	3	C 4 H 8 Cl N	0.04	105.0340	2.3	22.2	160.6	1.0	odd	ok
	4	C 5 H 8 Cl	0.00	103.0309	-3.7	-34.8	606.0	1.5	even	ok
	5	C 4 H 7 Cl N	0.00	104.0262	-2.6	-24.5	701.5	1.5	even	ok
	6	C 7 H 4 O	0.00	104.0257	-2.6	-24.8	787.4	6.0	odd	ok
300.0485	1	C 15 H 10 Cl N 3 O 2	100.00	299.0456	0.1	0.4	583.6	12.0	odd	ok

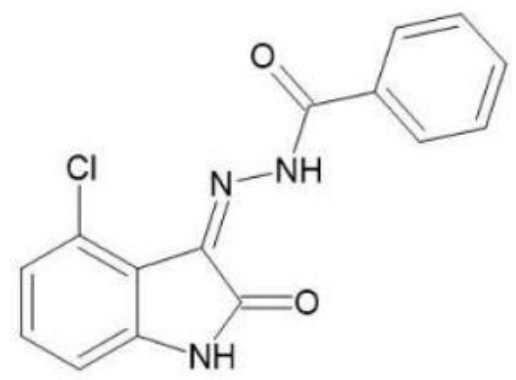

Molecular Formula: $C_{15}H_{10}ClN_3O_2$
Formula Weight: 299.714
Composition: C(60.11%) H(3.36%) Cl(11.83%) N(14.02%) O(10.68%)
Nominal Mass: 299 Da
Average Mass: 299.714 Da
[M+H]+: 300.053431 Da
[M+H]-: 300.054528 Da

N'-[(3Z)-4-chloro-2-oxo-1,2-dihydro-3H-indol-3-ylidene]-2-hydroxybenzohydrazide (**2b**)

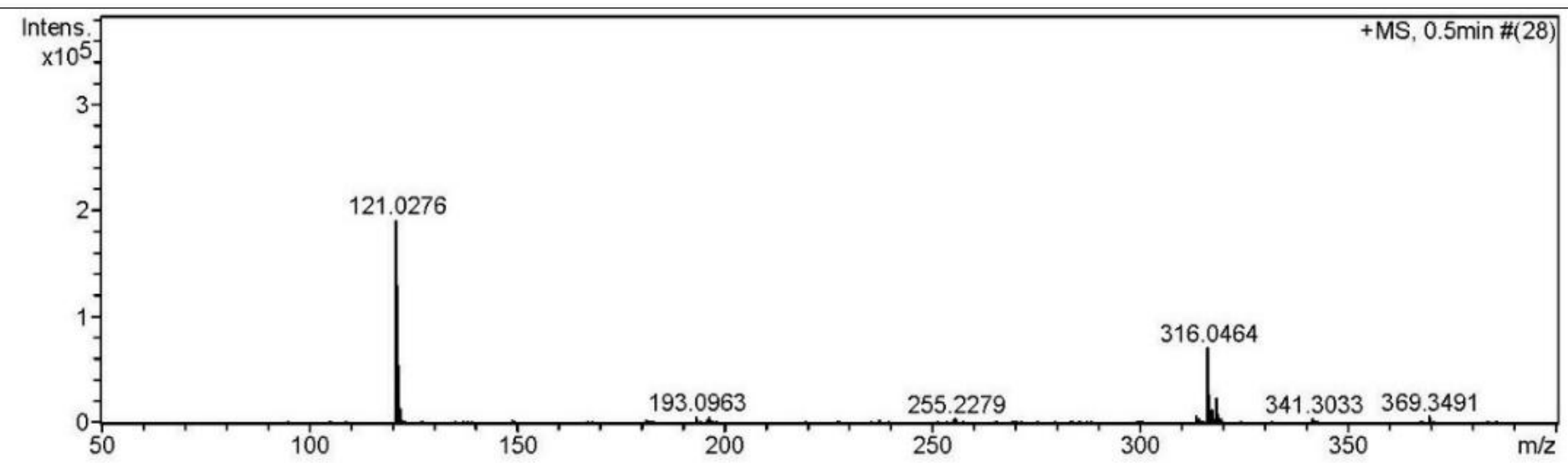

Meas. m/z	#	Formula	Score	m/z	err [mDa]	err [ppm]	mSigma	rdb	e⁻ Conf	N-Rule
121.0276	1	C 5 H 3 N 3 O	100.00	121.0271	-0.5	-4.5	3.4	6.0	odd	ok
	2	C 7 H 5 O 2	87.28	121.0284	0.8	6.6	3.6	5.5	even	ok
	3	C 4 H 8 Cl N O	0.09	121.0289	1.3	10.6	160.5	1.0	odd	ok
	4	C 4 H 7 Cl N O	0.00	120.0211	-3.6	-29.8	502.7	1.5	even	ok
	5	C 7 H 4 O 2	0.00	120.0206	-3.6	-30.1	533.6	6.0	odd	ok
	6	C 4 H 7 Cl N 2	0.00	118.0292	1.2	9.7	763.5	2.0	odd	ok
316.0464	1	C 15 H 11 Cl N 3 O 3	100.00	316.0483	1.9	6.1	3.8	11.5	even	ok
	2	C 15 H 10 Cl N 3 O 3	0.00	315.0405	-2.9	-9.2	591.2	12.0	odd	ok

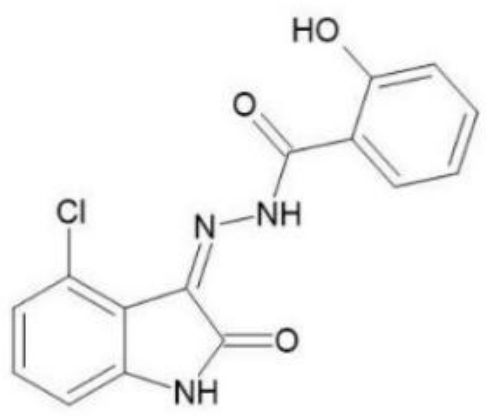

Molecular Formula: $C_{15}H_{10}ClN_3O_3$
Formula Weight: 315.713
Composition: C(57.07%) H(3.19%) Cl(11.23%) N(13.31%) O(15.20%)
Nominal Mass: 315 Da
Average Mass: 315.713 Da
[M+H]+: 316.048345 Da
[M+H]-: 316.049443 Da

N'-[(3Z)-4-chloro-2-oxo-1,2-dihydro-3H-indol-3-ylidene]-3-hydroxybenzohydrazide (**2c**)

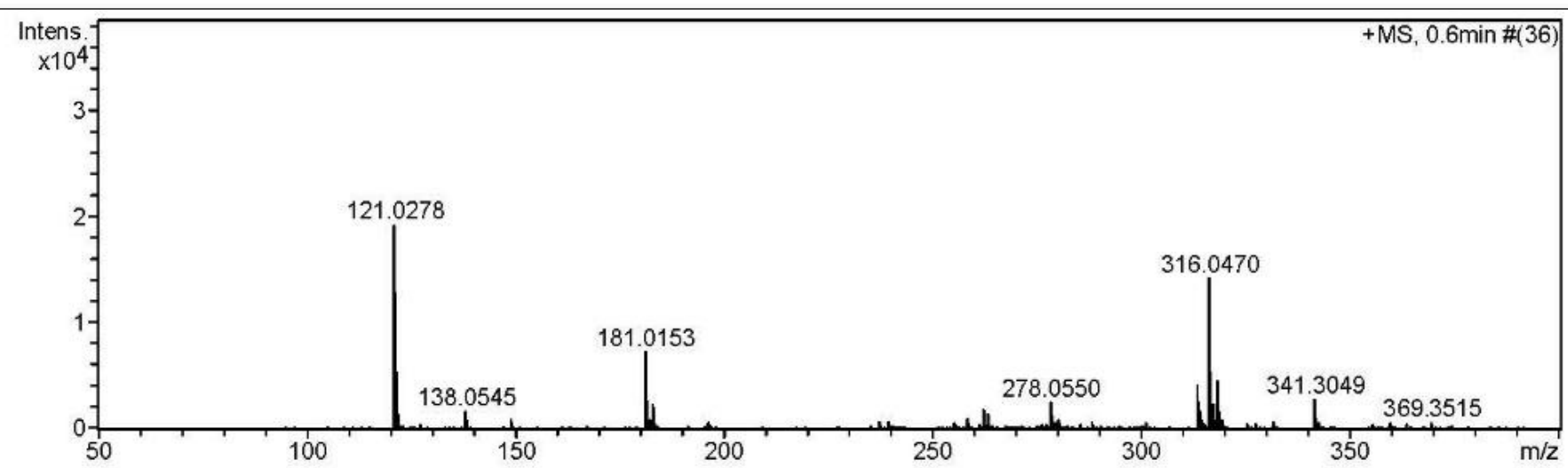

Meas. m/z	#	Formula	Score	m/z	err [mDa]	err [ppm]	mSigma	rdb	e⁻ Conf	N-Rule
121.0278	1	C 5 H 3 N 3 O	93.44	121.0271	-0.7	-6.2	3.1	6.0	odd	ok
	2	C 7 H 5 O 2	100.00	121.0284	0.6	4.9	3.8	5.5	even	ok
	3	C 4 H 8 Cl N O	0.11	121.0289	1.1	8.9	160.5	1.0	odd	ok
	4	C 4 H 7 Cl N O	0.00	120.0211	-3.8	-31.5	502.7	1.5	even	ok
	5	C 7 H 4 O 2	0.00	120.0206	-3.8	-31.8	533.6	6.0	odd	ok
	6	C 4 H 7 Cl N 2	0.00	118.0292	1.0	8.0	763.5	2.0	odd	ok
181.0153	1	C 8 H 6 Cl N 2 O	100.00	181.0163	1.0	5.8	23.1	6.5	even	ok
	2	C 12 H 3 O 2	0.00	179.0128	3.4	18.6	75.2	11.5	even	ok
	3	C 8 H 5 Cl N 2 O	0.00	180.0085	-3.9	-21.5	167.5	7.0	odd	ok
	4	C 8 H 5 Cl N 3	0.00	178.0167	1.1	6.2	168.3	7.5	even	ok
	5	C 10 H 7 Cl O	0.00	178.0180	3.2	17.9	172.1	7.0	odd	ok
	6	C 11 H 3 N O 2	0.04	181.0158	0.6	3.1	186.7	11.0	odd	ok
316.0470	1	C 15 H 11 Cl N 3 O 3	100.00	316.0483	1.4	4.3	8.1	11.5	even	ok
	2	C 15 H 10 Cl N 3 O 3	0.00	315.0405	-3.5	-11.0	588.8	12.0	odd	ok

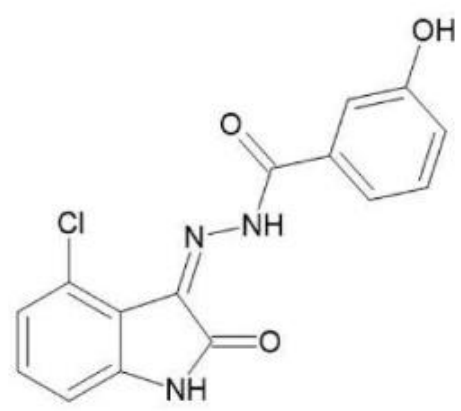

Molecular Formula: $C_{15}H_{10}ClN_3O_3$
Formula Weight: 315.713
Composition: C(57.07%) H(3.19%) Cl(11.23%) N(13.31%) O(15.20%)
Nominal Mass: 315 Da
Average Mass: 315.713 Da
[M+H]+: 316.048345 Da
[M+H]-: 316.049443 Da

4-chloro-N'-[(3Z)-4-chloro-2-oxo-1,2-dihydro-3H-indol-3-ylidene]benzohydrazide (**2d**)

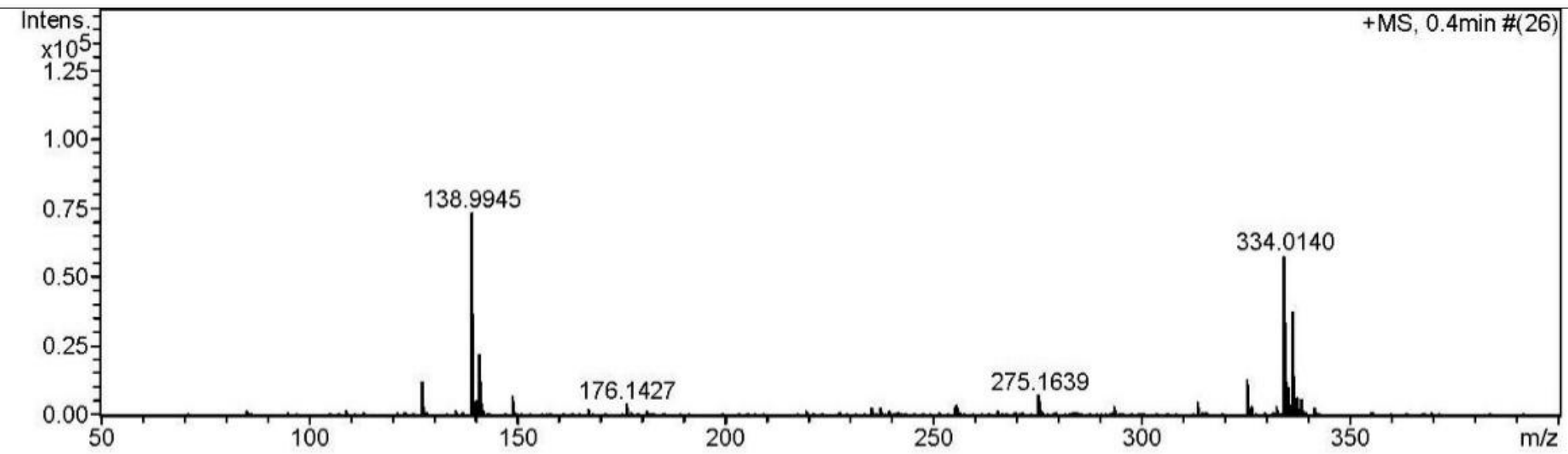

Meas. m/z	#	Formula	Score	m/z	err [mDa]	err [ppm]	mSigma	rdb	e⁻ Conf	N-Rule
138.9945	1	C 7 H 4 Cl O	100.00	138.9945	0.0	0.3	8.2	5.5	even	ok
	2	C 5 H 2 Cl N 3	51.05	138.9932	-1.3	-9.4	9.4	6.0	odd	ok
	3	C 2 H 4 Cl N 2 O 3	3.28	138.9905	-4.0	-28.7	25.8	1.5	even	ok
	4	C 4 N 3 O 3	0.00	137.9934	1.0	7.5	31.6	6.5	even	ok
	5	C 4 H 7 Cl 2 N	0.37	138.9950	0.5	3.8	143.8	1.0	odd	ok
	6	C 2 H 4 Cl N 3 O 2	0.00	136.9987	1.3	9.5	162.9	2.0	odd	ok
	7	C 3 H 5 Cl N O 3	0.00	137.9952	3.6	25.7	164.1	1.5	even	ok
	8	C 4 H 5 Cl O 3	0.00	135.9922	-1.7	-12.3	164.5	2.0	odd	ok
	9	C 4 H 6 Cl O 3	0.00	137.0000	2.7	19.6	164.5	1.5	even	ok
	10	C 7 H 3 Cl N	0.00	135.9949	0.5	3.7	165.3	6.5	even	ok
334.0140	1	C 15 H 10 Cl 2 N 3 O 2	100.00	334.0145	0.5	1.4	3.6	11.5	even	ok

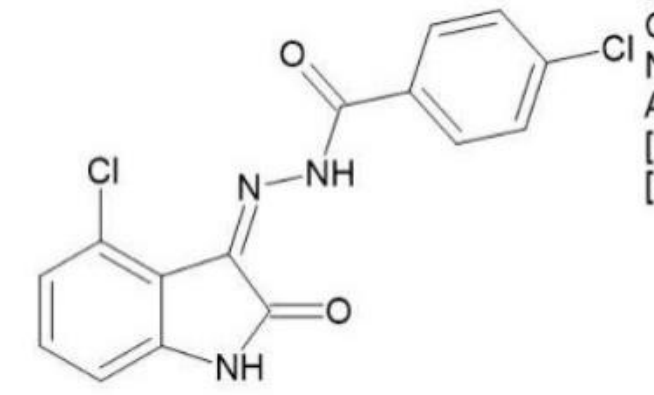

Molecular Formula: $C_{15}H_9Cl_2N_3O_2$
Formula Weight: 334.156
Composition: C(53.92%) H(2.71%) Cl(21.22%) N(12.58%) O(9.58%)
Nominal Mass: 333 Da
Average Mass: 334.156 Da
[M+H]+: 334.014458 Da
[M+H]-: 334.015556 Da

N'-[(3Z)-5-chloro-2-oxo-1,2-dihydro-3H-indol-3-ylidene]benzohydrazide (**3a**)

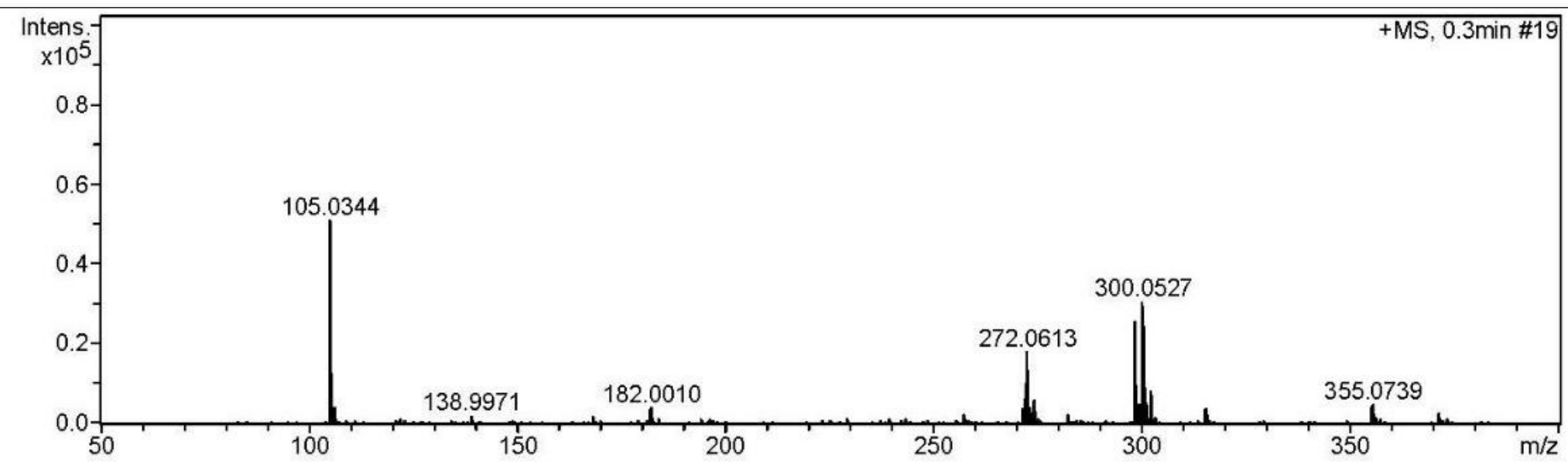

Meas. m/z	#	Formula	Score	m/z	err [mDa]	err [ppm]	mSigma	rdb	e⁻ Conf	N-Rule
105.0344	1	C 7 H 5 O	100.00	105.0335	-0.9	-8.6	2.8	5.5	even	ok
	2	C 5 H 3 N 3	36.04	105.0321	-2.2	-21.4	8.8	6.0	odd	ok
	3	C 4 H 8 Cl N	0.17	105.0340	-0.4	-4.0	160.9	1.0	odd	ok
	4	C 3 H 6 N O 3	0.00	104.0342	2.6	25.1	556.5	1.5	even	ok
300.0527	1	C 15 H 11 Cl N 3 O 2	100.00	300.0534	0.7	2.4	32.6	11.5	even	ok

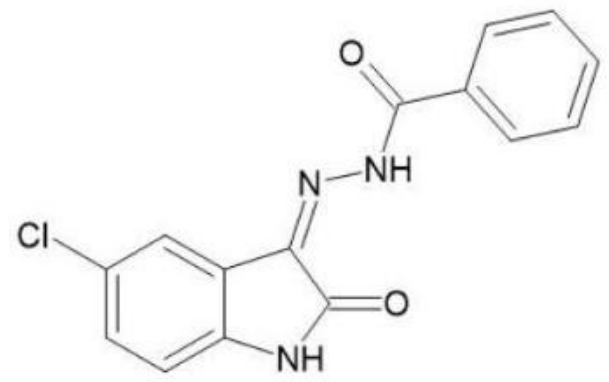

Molecular Formula: $C_{15}H_{10}ClN_3O_2$
Formula Weight: 299.714
Composition: C(60.11%) H(3.36%) Cl(11.83%) N(14.02%) O(10.68%)
Nominal Mass: 299 Da
Average Mass: 299.714 Da
[M+H]+: 300.053431 Da
[M+H]-: 300.054528 Da

N'-[(3Z)-5-chloro-2-oxo-1,2-dihydro-3H-indol-3-ylidene]-3-hydroxybenzohydrazide (**3b**)

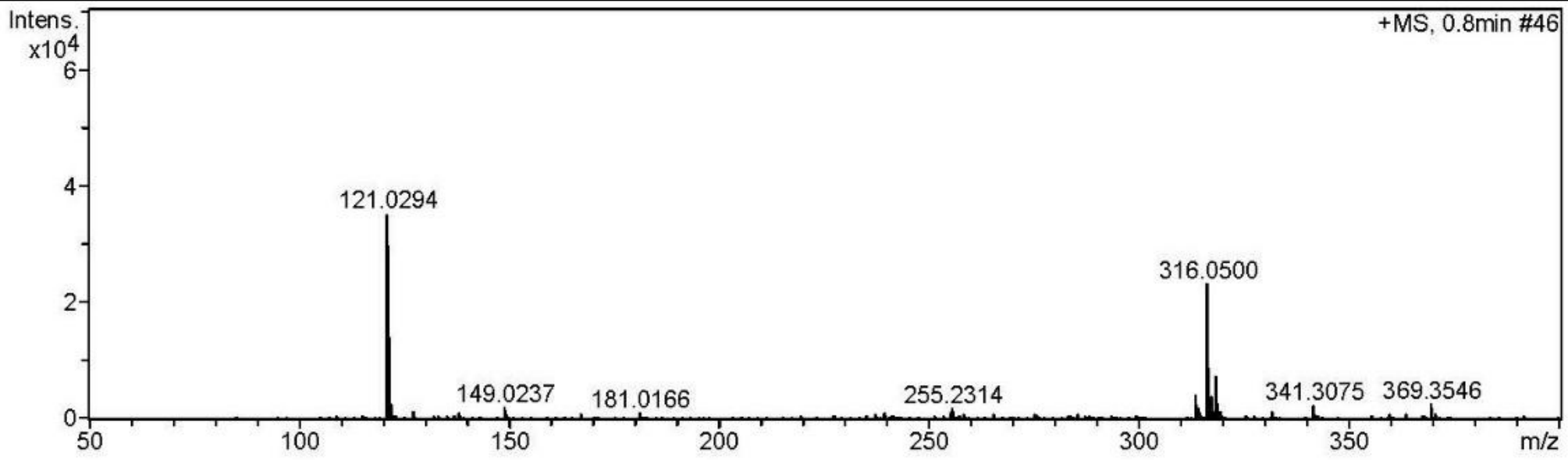

Meas. m/z	#	Formula	Score	m/z	err [mDa]	err [ppm]	mSigma	rdb	e⁻ Conf	N-Rule
121.0294	1	C 7 H 5 O 2	100.00	121.0284	-1.0	-8.6	2.4	5.5	even	ok
	2	C 5 H 3 N 3 O	36.15	121.0271	-2.4	-19.7	6.4	6.0	odd	ok
	3	C 4 H 8 Cl N O	0.18	121.0289	-0.6	-4.6	160.7	1.0	odd	ok
	4	C 4 H 7 Cl N 2	0.00	118.0292	-0.7	-5.5	763.5	2.0	odd	ok
316.0500	1	C 15 H 11 Cl N 3 O 3	100.00	316.0483	-1.6	-5.2	12.3	11.5	even	ok

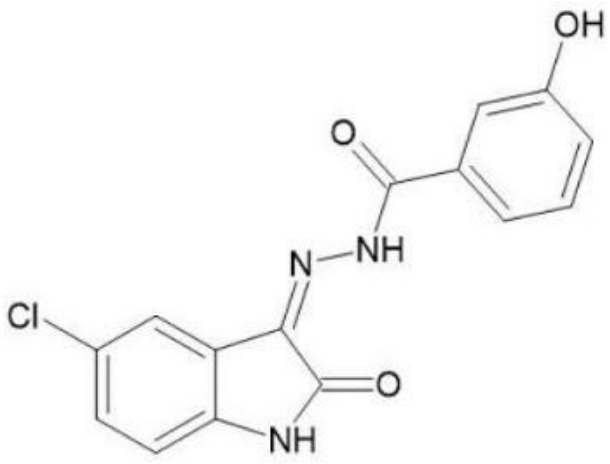

Molecular Formula: $C_{15}H_{10}ClN_3O_3$
Formula Weight: 315.713
Composition: C(57.07%) H(3.19%) Cl(11.23%) N(13.31%) O(15.20%)
Nominal Mass: 315 Da
Average Mass: 315.713 Da
[M+H]+: 316.048345 Da
[M+H]-: 316.049443 Da

2-chloro-N'-[(3Z)-5-chloro-2-oxo-1,2-dihydro-3H-indol-3-ylidene]benzohydrazide (**3c**)

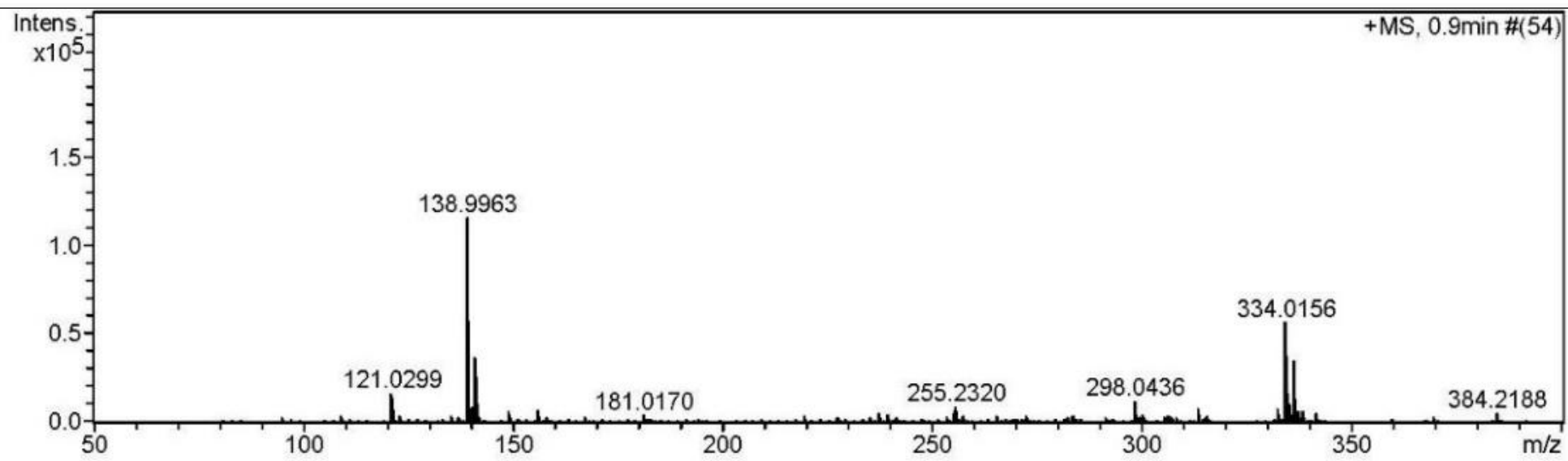

Meas. m/z	#	Formula	Score	m/z	err [mDa]	err [ppm]	mSigma	rdb	e⁻ Conf	N-Rule
138.9963	1	C 7 H 4 Cl O	100.00	138.9945	-1.7	-12.6	2.2	5.5	even	ok
	2	C 5 H 2 Cl N 3	29.75	138.9932	-3.1	-22.2	6.5	6.0	odd	ok
	3	C 4 H 7 Cl 2 N	0.76	138.9950	-1.3	-9.1	139.3	1.0	odd	ok
	4	C 4 H 6 Cl O 3	0.00	137.0000	0.9	6.8	604.2	1.5	even	ok
	5	C 2 H 4 Cl N 3 O 2	0.00	136.9987	-0.5	-3.3	604.7	2.0	odd	ok
	6	C 7 H 4 Cl N	0.00	137.0027	3.5	25.5	606.5	6.0	odd	ok
	7	C 7 H 3 Cl N	0.00	135.9949	-1.3	-9.1	717.4	6.5	even	ok
	8	C 4 H 5 Cl O 3	0.00	135.9922	-3.5	-25.1	721.1	2.0	odd	ok
	9	C 3 H 5 Cl N O 3	0.00	137.9952	1.8	12.9	721.7	1.5	even	ok
	10	C 4 N 3 O 3	0.00	137.9934	-0.7	-5.3	795.6	6.5	even	ok
334.0156	1	C 15 H 10 Cl 2 N 3 O 2	100.00	334.0145	-1.1	-3.3	13.8	11.5	even	ok

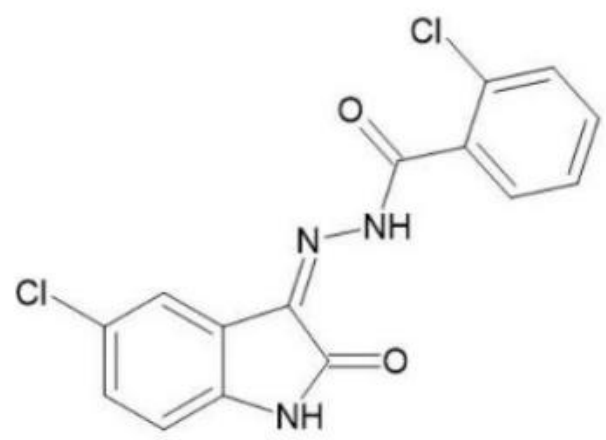

Molecular Formula: $C_{15}H_9Cl_2N_3O_2$
Formula Weight: 334.156
Composition: C(53.92%) H(2.71%) Cl(21.22%) N(12.58%) O(9.58%)
Nominal Mass: 333 Da
Average Mass: 334.156 Da
[M+H]+: 334.014458 Da
[M+H]-: 334.015556 Da

N'-[(3Z)-6-chloro-2-oxo-1,2-dihydro-3H-indol-3-ylidene]benzohydrazide (**4a**)

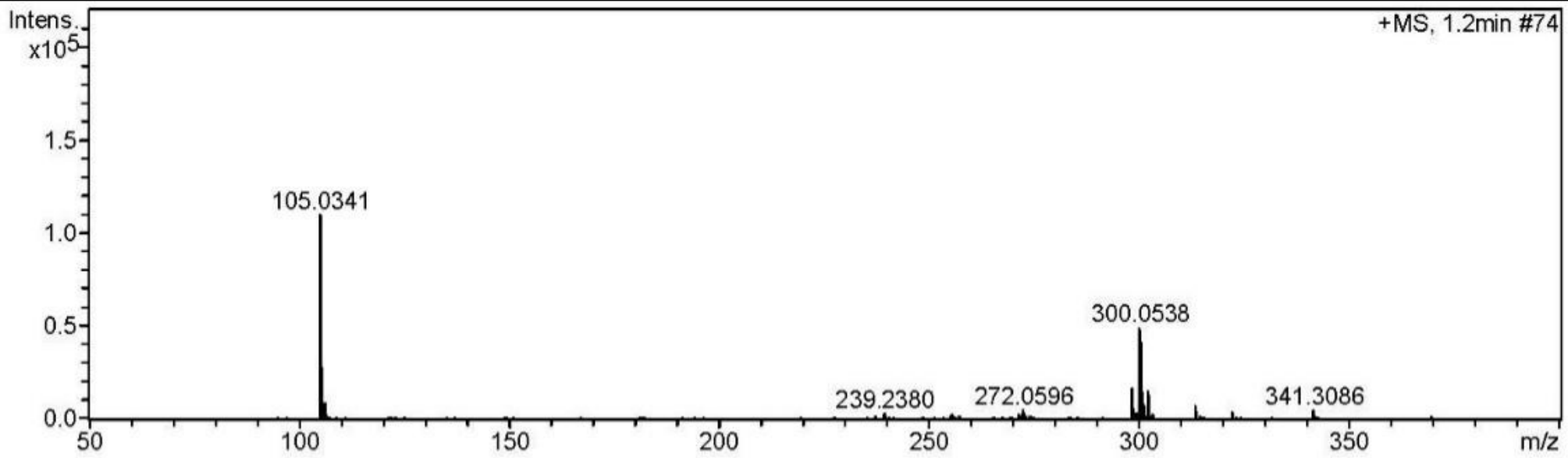

Meas. m/z	#	Formula	Score	m/z	err [mDa]	err [ppm]	mSigma	rdb	e⁻ Conf	N-Rule
105.0341	1	C 7 H 5 O	100.00	105.0335	-0.6	-6.1	1.3	5.5	even	ok
	2	C 5 H 3 N 3	38.67	105.0321	-2.0	-18.9	7.4	6.0	odd	ok
	3	C 4 H 8 Cl N	0.17	105.0340	-0.2	-1.5	160.8	1.0	odd	ok
	4	C 3 H 6 N O 3	0.00	104.0342	2.9	27.7	556.5	1.5	even	ok
300.0538	1	C 15 H 11 Cl N 3 O 2	100.00	300.0534	-0.4	-1.3	14.7	11.5	even	ok

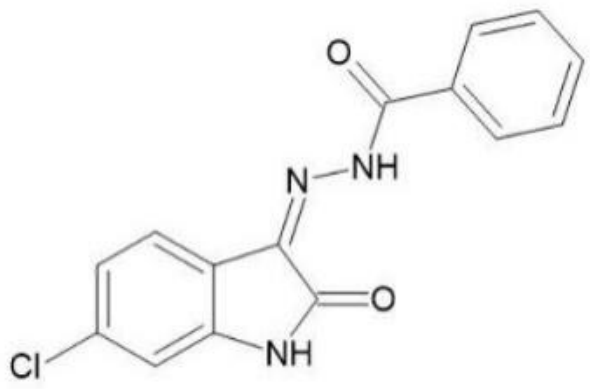

Molecular Formula: $C_{15}H_{10}ClN_3O_2$
Formula Weight: 299.714
Composition: C(60.11%) H(3.36%) Cl(11.83%) N(14.02%) O(10.68%)
Nominal Mass: 299 Da
Average Mass: 299.714 Da
[M+H]+: 300.053431 Da
[M+H]-: 300.054528 Da

N'-[(3Z)-6-chloro-2-oxo-1,2-dihydro-3H-indol-3-ylidene]-3-hydroxybenzohydrazide (**4b**)

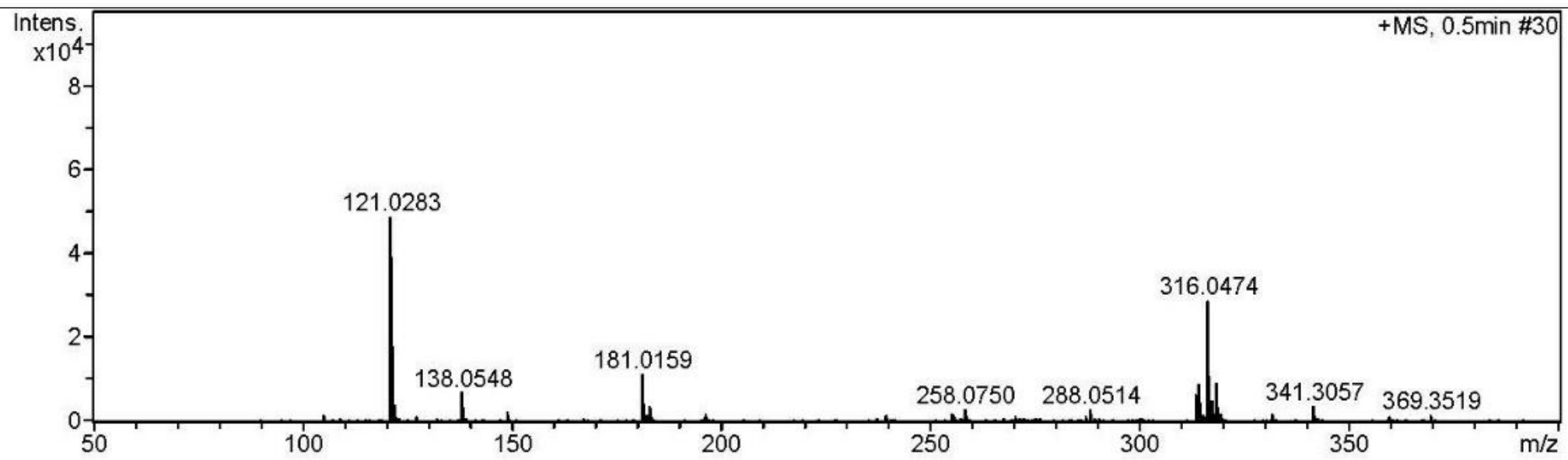

Meas. m/z	#	Formula	Score	m/z	err [mDa]	err [ppm]	mSigma	rdb	e⁻ Conf	N-Rule
121.0283	1	C 7 H 5 O 2	100.00	121.0284	0.1	1.2	2.8	5.5	even	ok
	2	C 5 H 3 N 3 O	53.90	121.0271	-1.2	-9.9	7.6	6.0	odd	ok
	3	C 4 H 8 Cl N O	0.11	121.0289	0.6	5.3	160.8	1.0	odd	ok
	4	C 4 H 7 Cl N 2	0.00	118.0292	0.5	4.3	162.2	2.0	odd	ok
316.0474	1	C 15 H 11 Cl N 3 O 3	100.00	316.0483	0.9	3.0	12.3	11.5	even	ok
	2	C 15 H 10 Cl N 3 O 3	0.00	315.0405	-3.9	-12.3	578.2	12.0	odd	ok

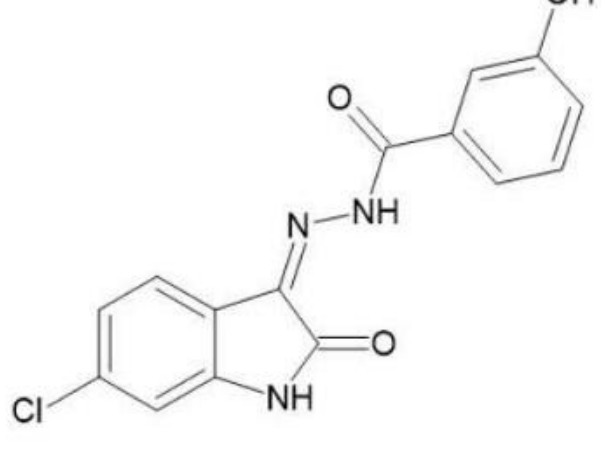

Molecular Formula: $C_{15}H_{10}ClN_3O_3$
Formula Weight: 315.713
Composition: C(57.07%) H(3.19%) Cl(11.23%) N(13.31%) O(15.20%)
Nominal Mass: 315 Da
Average Mass: 315.713 Da
[M+H]+: 316.048345 Da
[M+H]-: 316.049443 Da

N'-[(3Z)-7-chloro-2-oxo-1,2-dihydro-3H-indol-3-ylidene]benzohydrazide (**5a**)

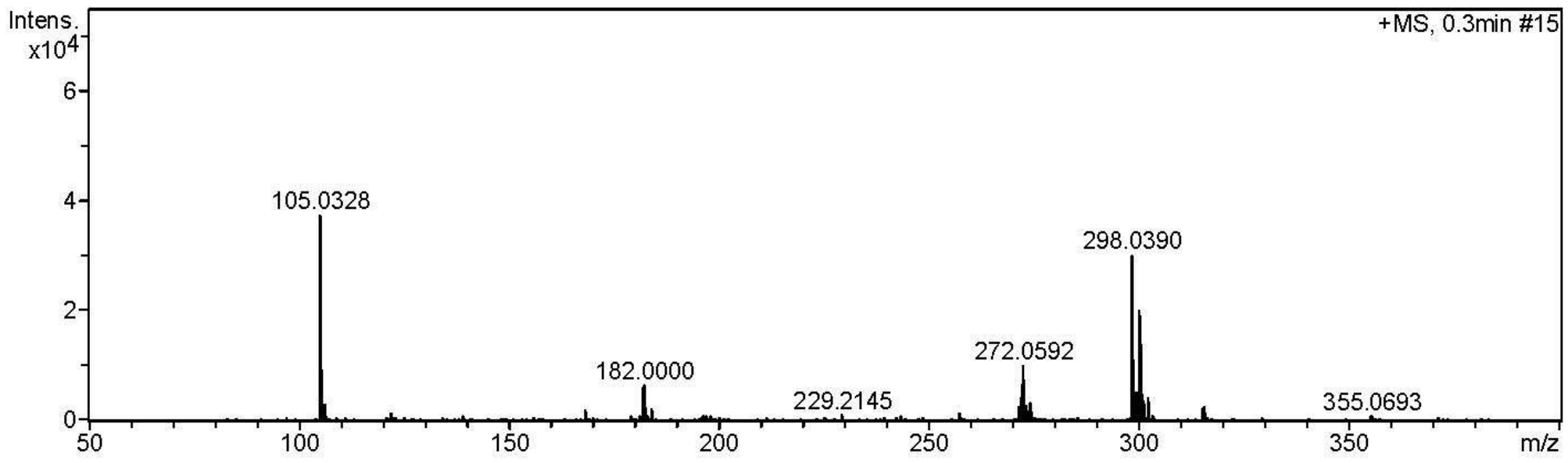

Meas. m/z	#	Formula	Score	m/z	err [mDa]	err [ppm]	mSigma	rdb	e⁻ Conf	N-Rule
105.0328	1	C 7 H 5 O	100.00	105.0335	0.7	7.0	1.5	5.5	even	ok
	2	C 5 H 3 N 3	96.14	105.0321	-0.6	-5.8	7.6	6.0	odd	ok
	3	C 2 H 5 N 2 O 3	8.15	105.0295	-3.3	-31.3	28.7	1.5	even	ok
	4	C 4 H 8 Cl N	0.10	105.0340	1.2	11.6	160.8	1.0	odd	ok
	5	C 4 H 7 Cl N	0.00	104.0262	-3.7	-35.1	502.7	1.5	even	ok
	6	C 7 H 4 O	0.00	104.0257	-3.7	-35.4	533.6	6.0	odd	ok
298.0390	1	C 15 H 9 Cl N 3 O 2	100.00	298.0378	-1.3	-4.2	160.0	12.5	even	ok
300.0465	1	C 15 H 10 Cl N 3 O 2	100.00	299.0456	2.0	6.8	508.0	12.0	odd	ok

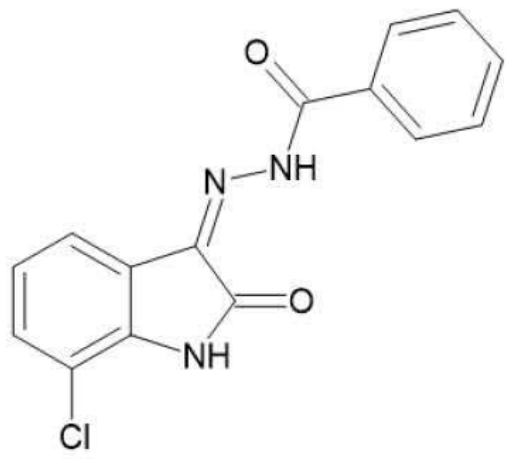

Molecular Formula: $C_{15}H_{10}ClN_3O_2$
Formula Weight: 299.714
Composition: C(60.11%) H(3.36%) Cl(11.83%) N(14.02%) O(10.68%)
Nominal Mass: 299 Da
Average Mass: 299.714 Da
[M+H]+: 300.053431 Da
[M+H]-: 300.054528 Da

N'-[(3Z)-7-chloro-2-oxo-1,2-dihydro-3H-indol-3-ylidene]-3-hydroxybenzohydrazide (**5b**)

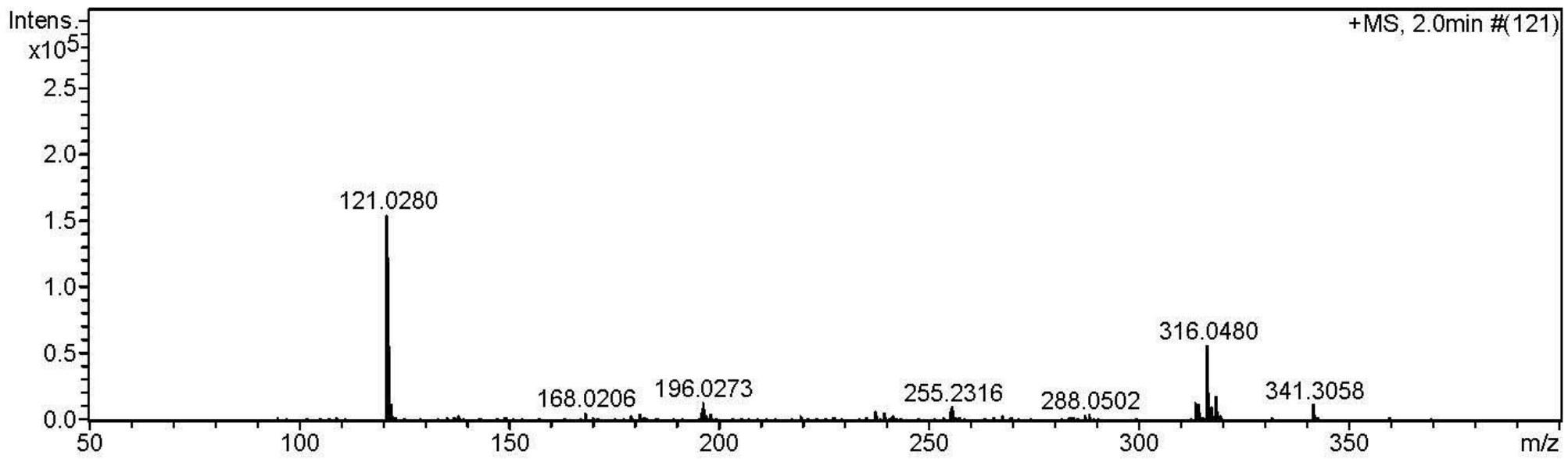

Meas. m/z	#	Formula	Score	m/z	err [mDa]	err [ppm]	mSigma	rdb	e⁻ Conf	N-Rule
121.0280	1	C 7 H 5 O 2	100.00	121.0284	0.4	3.3	2.6	5.5	even	ok
	2	C 5 H 3 N 3 O	69.69	121.0271	-0.9	-7.8	7.1	6.0	odd	ok
	3	C 4 H 8 Cl N O	0.11	121.0289	0.9	7.3	160.8	1.0	odd	ok
	4	C 4 H 7 Cl N 2	0.00	118.0292	0.8	6.3	763.5	2.0	odd	ok
316.0480	1	C 15 H 11 Cl N 3 O 3	100.00	316.0483	0.4	1.2	9.0	11.5	even	ok

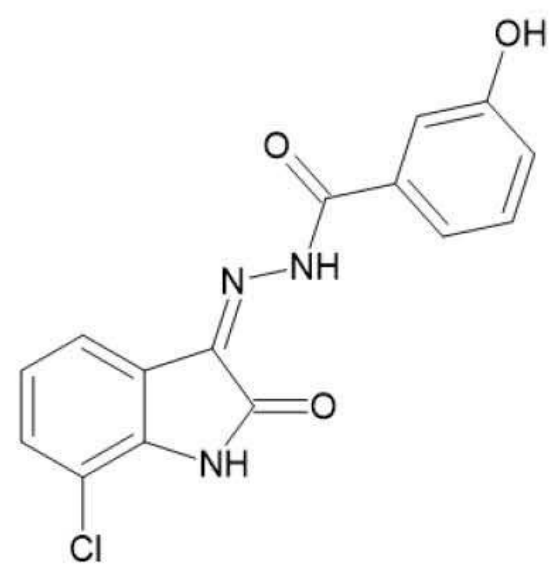

Molecular Formula: $C_{15}H_{10}ClN_3O_3$
Formula Weight: 315.713
Composition: C(57.07%) H(3.19%) Cl(11.23%) N(13.31%) O(15.20%)
Nominal Mass: 315 Da
Average Mass: 315.713 Da
[M+H]+: 316.048345 Da
[M+H]-: 316.049443 Da

2-chloro-N'-[(3Z)-7-chloro-2-oxo-1,2-dihydro-3H-indol-3-ylidene]benzohydrazide (**5c**)

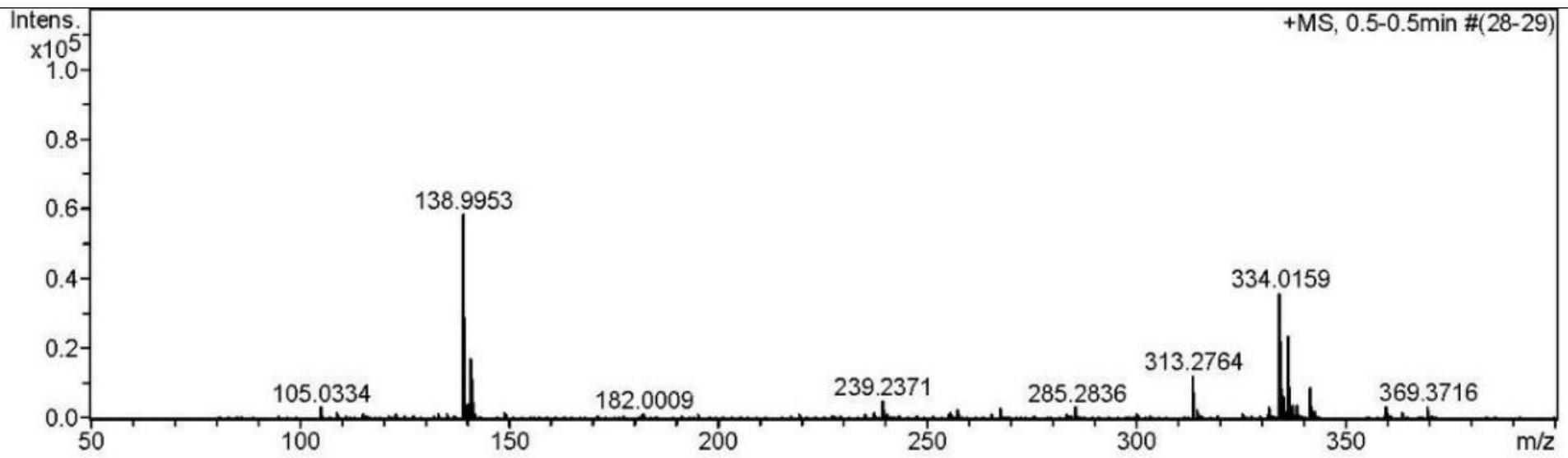

Meas. m/z	#	Formula	Score	m/z	err [mDa]	err [ppm]	mSigma	rdb	e⁻ Conf	N-Rule
138.9953	1	C 7 H 4 Cl O	100.00	138.9945	-0.8	-5.7	10.1	5.5	even	ok
	2	C 5 H 2 Cl N 3	40.55	138.9932	-2.1	-15.3	11.1	6.0	odd	ok
	3	C 4 N 3 O 3	0.00	137.9934	0.2	1.6	31.6	6.5	even	ok
	4	C 4 H 7 Cl 2 N	0.55	138.9950	-0.3	-2.2	145.2	1.0	odd	ok
	5	C 2 H 4 Cl N 3 O 2	0.00	136.9987	0.5	3.6	162.9	2.0	odd	ok
	6	C 3 H 5 Cl N O 3	0.00	137.9952	2.8	19.8	164.1	1.5	even	ok
	7	C 4 H 6 Cl O 3	0.00	137.0000	1.9	13.7	164.5	1.5	even	ok
	8	C 4 H 5 Cl O 3	0.00	135.9922	-2.5	-18.2	164.5	2.0	odd	ok
	9	C 7 H 3 Cl N	0.00	135.9949	-0.3	-2.2	165.3	6.5	even	ok
334.0159	1	C 15 H 10 Cl 2 N 3 O 2	100.00	334.0145	-1.5	-4.4	2.8	11.5	even	ok

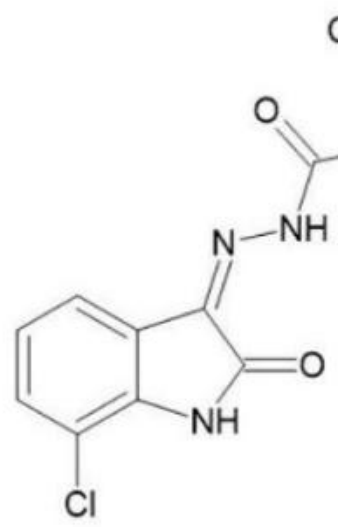

Molecular Formula: $C_{15}H_9Cl_2N_3O_2$
Formula Weight: 334.156
Composition: C(53.92%) H(2.71%) Cl(21.22%) N(12.58%) O(9.58%)
Nominal Mass: 333 Da
Average Mass: 334.156 Da
[M+H]+: 334.014458 Da
[M+H]-: 334.015556 Da

3-chloro-N'-[(3Z)-7-chloro-2-oxo-1,2-dihydro-3H-indol-3-ylidene]benzohydrazide (**5d**)

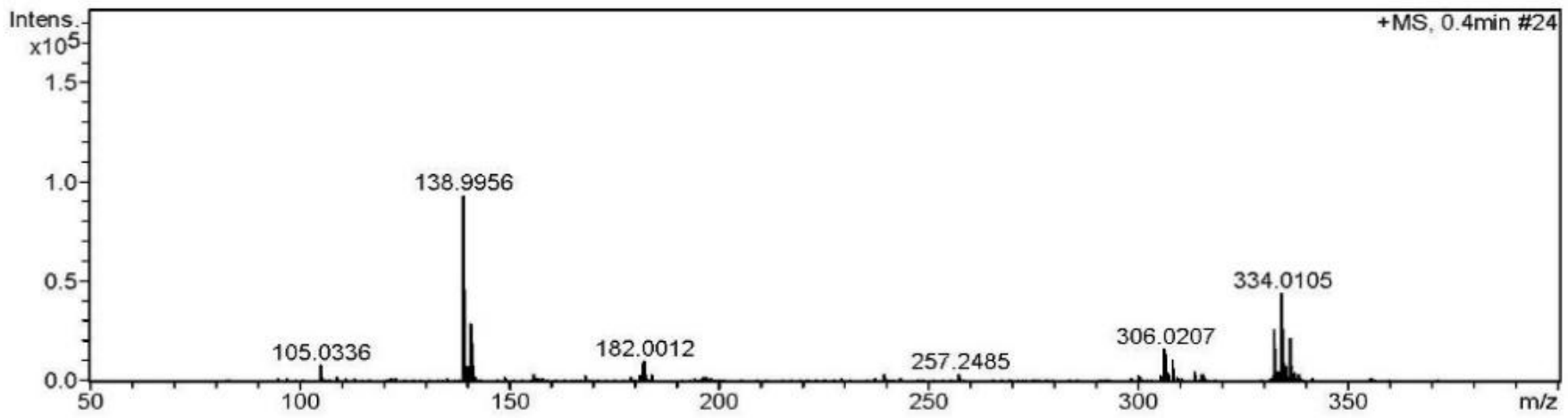

Meas. m/z	#	Formula	Score	m/z	err [mDa]	err [ppm]	mSigma	rdb	e⁻ Conf	N-Rule
138.9956	1	C 7 H 4 Cl O	100.00	138.9945	-1.1	-7.8	4.7	5.5	even	ok
	2	C 5 H 2 Cl N 3	36.04	138.9932	-2.4	-17.4	8.2	6.0	odd	ok
	3	C 4 N 3 O 3	0.00	137.9934	-0.1	-0.5	31.6	6.5	even	ok
	4	C 4 H 7 Cl 2 N	0.67	138.9950	-0.6	-4.3	141.2	1.0	odd	ok
	5	C 2 H 4 Cl N 3 O 2	0.00	136.9987	0.2	1.5	162.9	2.0	odd	ok
	6	C 3 H 5 Cl N O 3	0.00	137.9952	2.5	17.7	164.1	1.5	even	ok
	7	C 4 H 6 Cl O 3	0.00	137.0000	1.6	11.6	164.5	1.5	even	ok
	8	C 4 H 5 Cl O 3	0.00	135.9922	-2.8	-20.3	164.5	2.0	odd	ok
	9	C 7 H 3 Cl N	0.00	135.9949	-0.6	-4.3	165.3	6.5	even	ok
332.0011	1	C 15 H 8 Cl 2 N 3 O 2	100.00	331.9988	-2.3	-6.9	272.5	12.5	even	ok
334.0105	1	C 15 H 9 Cl 2 N 3 O 2	100.00	333.0066	-0.8	-2.5	558.2	12.0	odd	ok

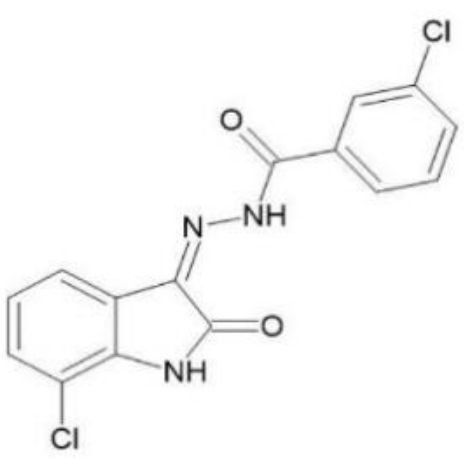

Molecular Formula: $C_{15}H_9Cl_2N_3O_2$
Formula Weight: 334.156
Composition: C(53.92%) H(2.71%) Cl(21.22%) N(12.58%) O(9.58%)
Nominal Mass: 333 Da
Average Mass: 334.156 Da
[M+H]+: 334.014458 Da
[M+H]-: 334.015556 Da

4-chloro-N'-[(3Z)-7-chloro-2-oxo-1,2-dihydro-3H-indol-3-ylidene] benzohydrazide (**5e**)

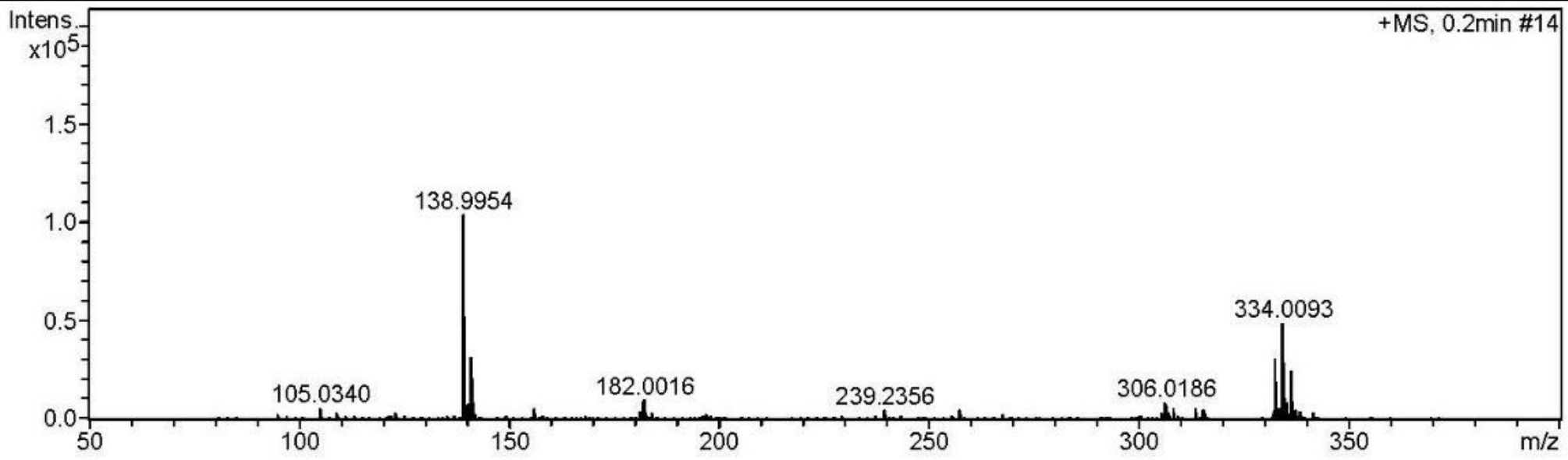

Meas. m/z	#	Formula	Score	m/z	err [mDa]	err [ppm]	mSigma	rdb	e⁻ Conf	N-Rule
138.9954	1	C 7 H 4 Cl O	100.00	138.9945	-0.9	-6.2	7.0	5.5	even	ok
	2	C 5 H 2 Cl N 3	39.13	138.9932	-2.2	-15.8	9.0	6.0	odd	ok
	3	C 4 N 3 O 3	0.00	137.9934	0.1	1.1	31.6	6.5	even	ok
	4	C 4 H 7 Cl 2 N	0.61	138.9950	-0.4	-2.7	143.0	1.0	odd	ok
	5	C 2 H 4 Cl N 3 O 2	0.00	136.9987	0.4	3.1	162.9	2.0	odd	ok
	6	C 3 H 5 Cl N O 3	0.00	137.9952	2.7	19.3	164.1	1.5	even	ok
	7	C 4 H 6 Cl O 3	0.00	137.0000	1.8	13.2	164.5	1.5	even	ok
	8	C 4 H 5 Cl O 3	0.00	135.9922	-2.6	-18.7	164.5	2.0	odd	ok
	9	C 7 H 3 Cl N	0.00	135.9949	-0.4	-2.7	165.3	6.5	even	ok
332.0000	1	C 15 H 8 Cl 2 N 3 O 2	100.00	331.9988	-1.2	-3.5	265.2	12.5	even	ok
334.0093	1	C 15 H 9 Cl 2 N 3 O 2	100.00	333.0066	0.3	0.9	558.7	12.0	odd	ok

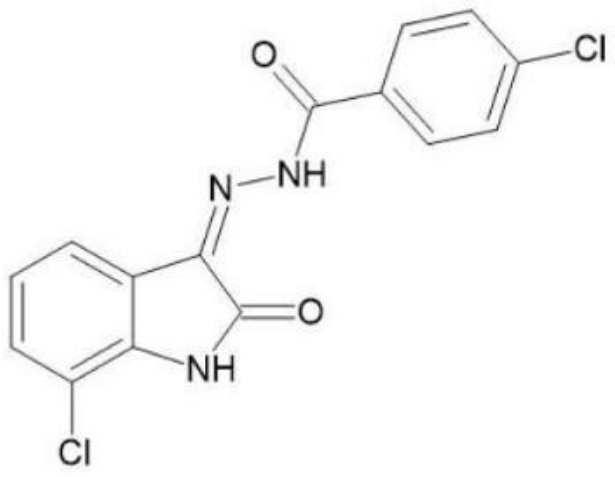

Molecular Formula: $C_{15}H_9Cl_2N_3O_2$
Formula Weight: 334.156
Composition: C(53.92%) H(2.71%) Cl(21.22%) N(12.58%) O(9.58%)
Nominal Mass: 333 Da
Average Mass: 334.156 Da
[M+H]+: 334.014458 Da
[M+H]-: 334.015556 Da

High-performance liquid chromatography (HPLC) spectra

N'-[(3Z)-2-oxo-1,2-dihydro-3H-indol-3-ylidene]benzohydrazide (**1a**)

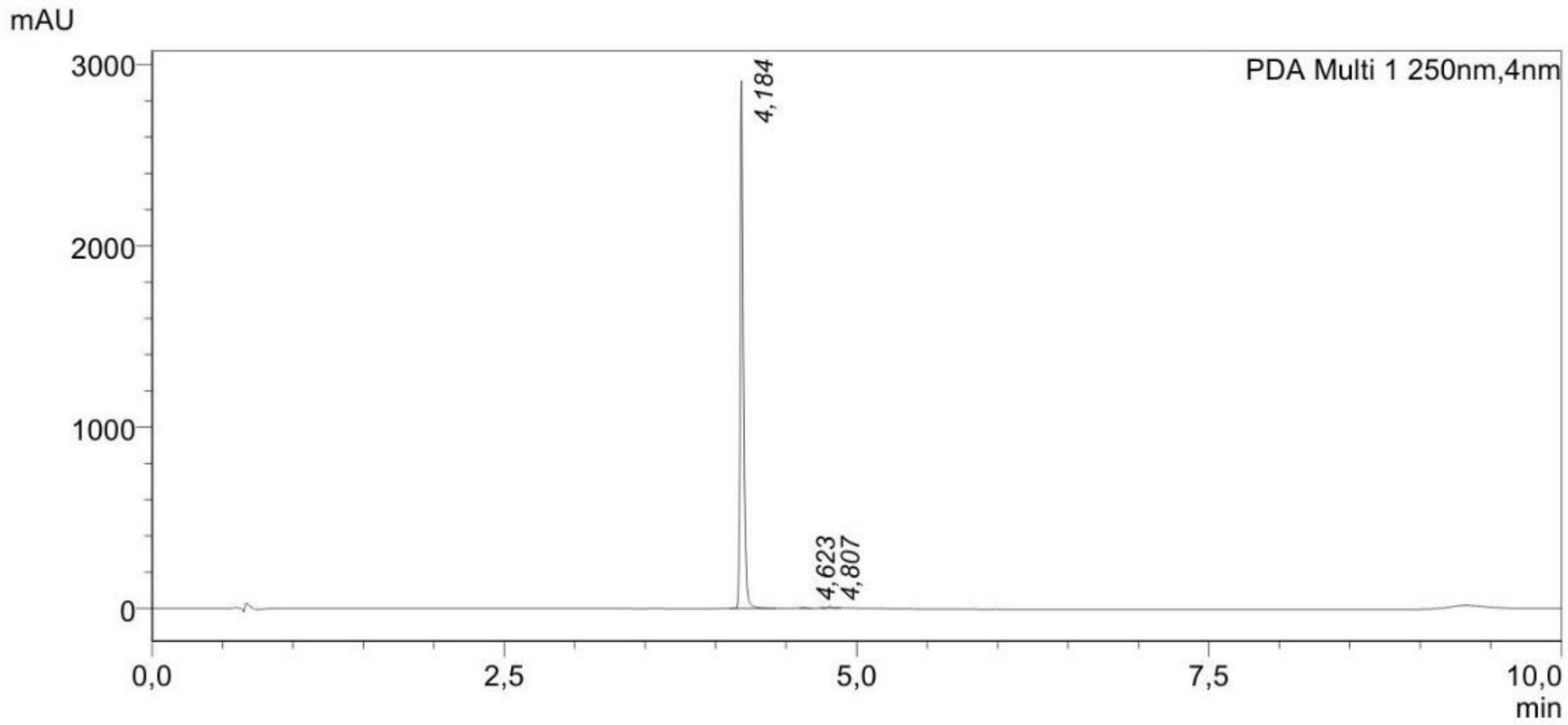

<PeakTable>

PDA Ch1 250nm

Peak#	Ret. Time	Area	Area%
1	4,184	4497996	99,550
2	4,623	5391	0,119
3	4,807	14951	0,331
Total		4518338	100,000

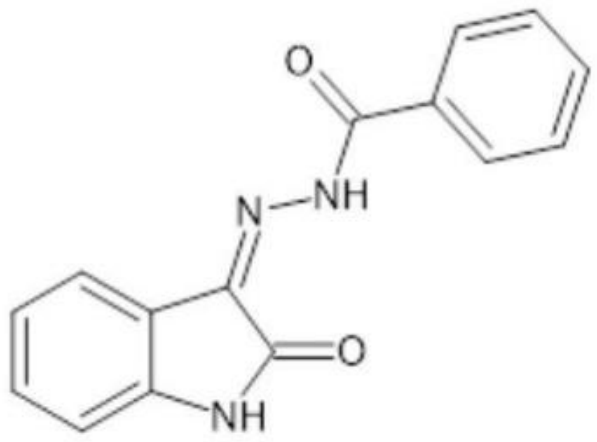

3-hydroxy-N'-[(3Z)-2-oxo-1,2-dihydro-3H-indol-3-ylidene]benzohydrazide (**1b**)

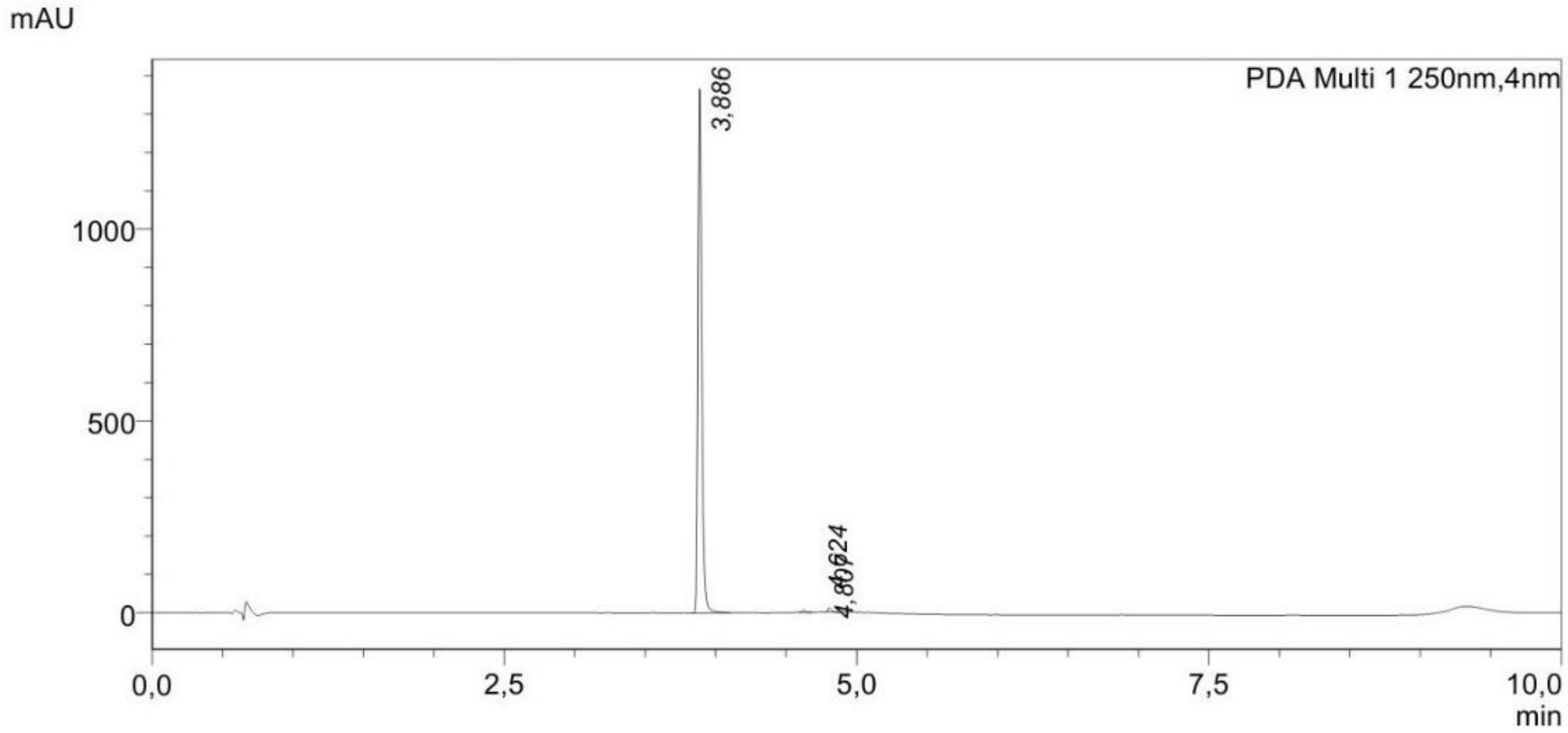

<PeakTable>

PDA Ch1 250nm

Peak#	Ret. Time	Area	Area%
1	3,886	2553006	98,978
2	4,624	7039	0,273
3	4,807	19318	0,749
Total		2579364	100,000

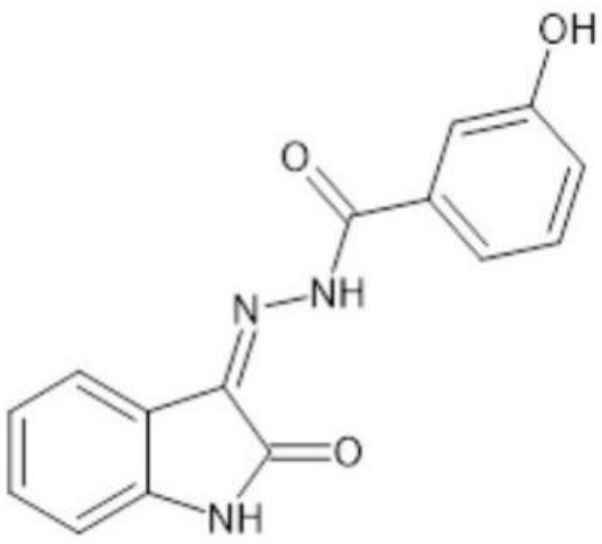

4-hydroxy-N'-[(3Z)-2-oxo-1,2-dihydro-3H-indol-3-ylidene]benzohydrazide (**1c**)

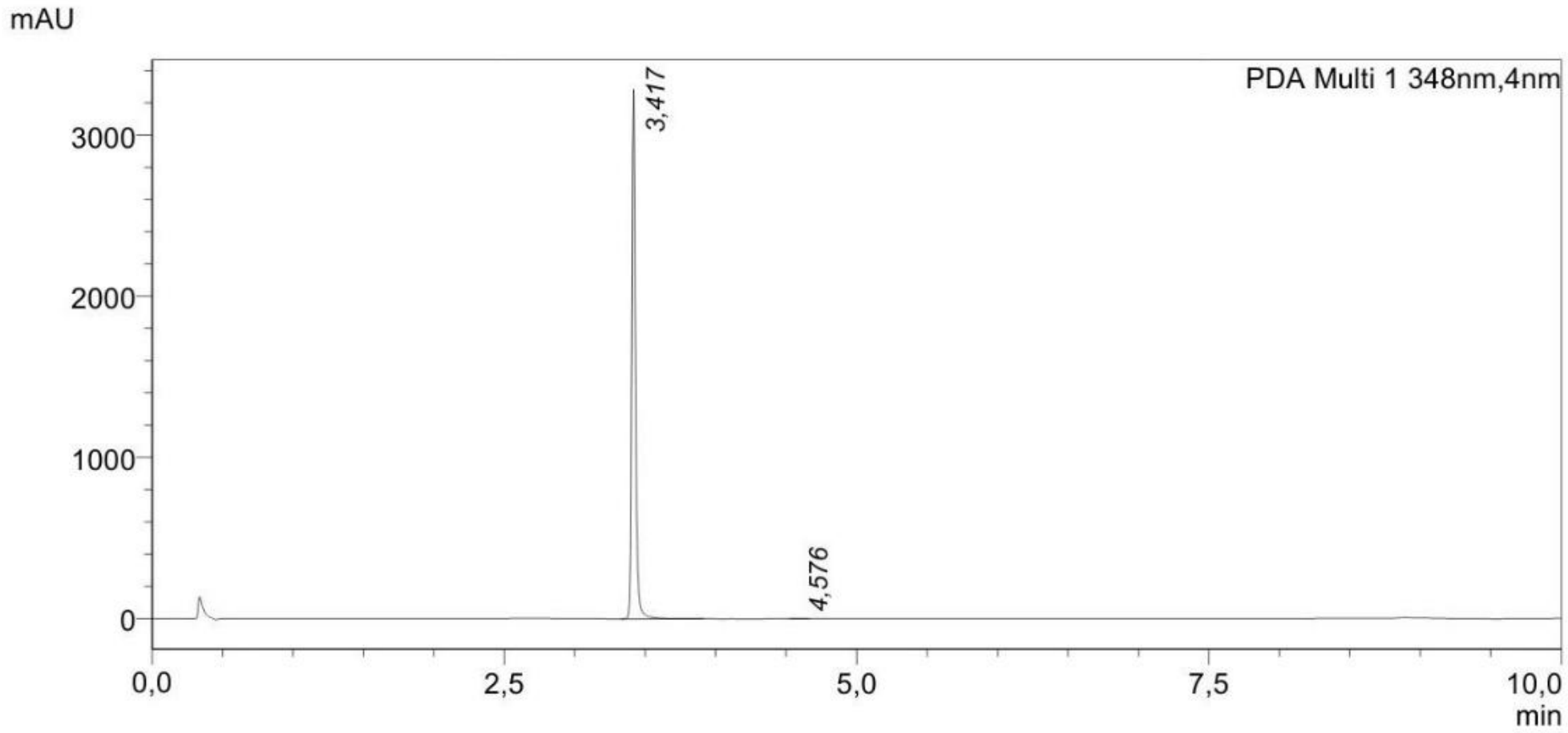

<PeakTable>

PDA Ch1 348nm

Peak#	Ret. Time	Area	Area%
1	3,417	5868501	99,956
2	4,576	2572	0,044
Total		5871073	100,000

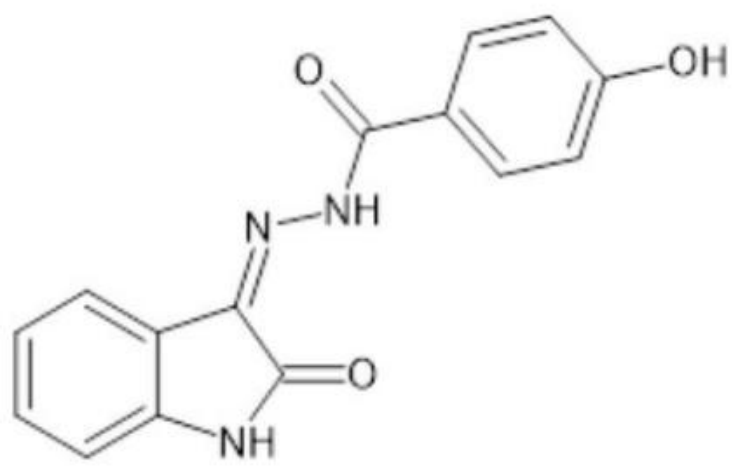

3-chloro-N'-[(3Z)-2-oxo-1,2-dihydro-3H-indol-3-ylidene]benzohydrazide (**1d**)

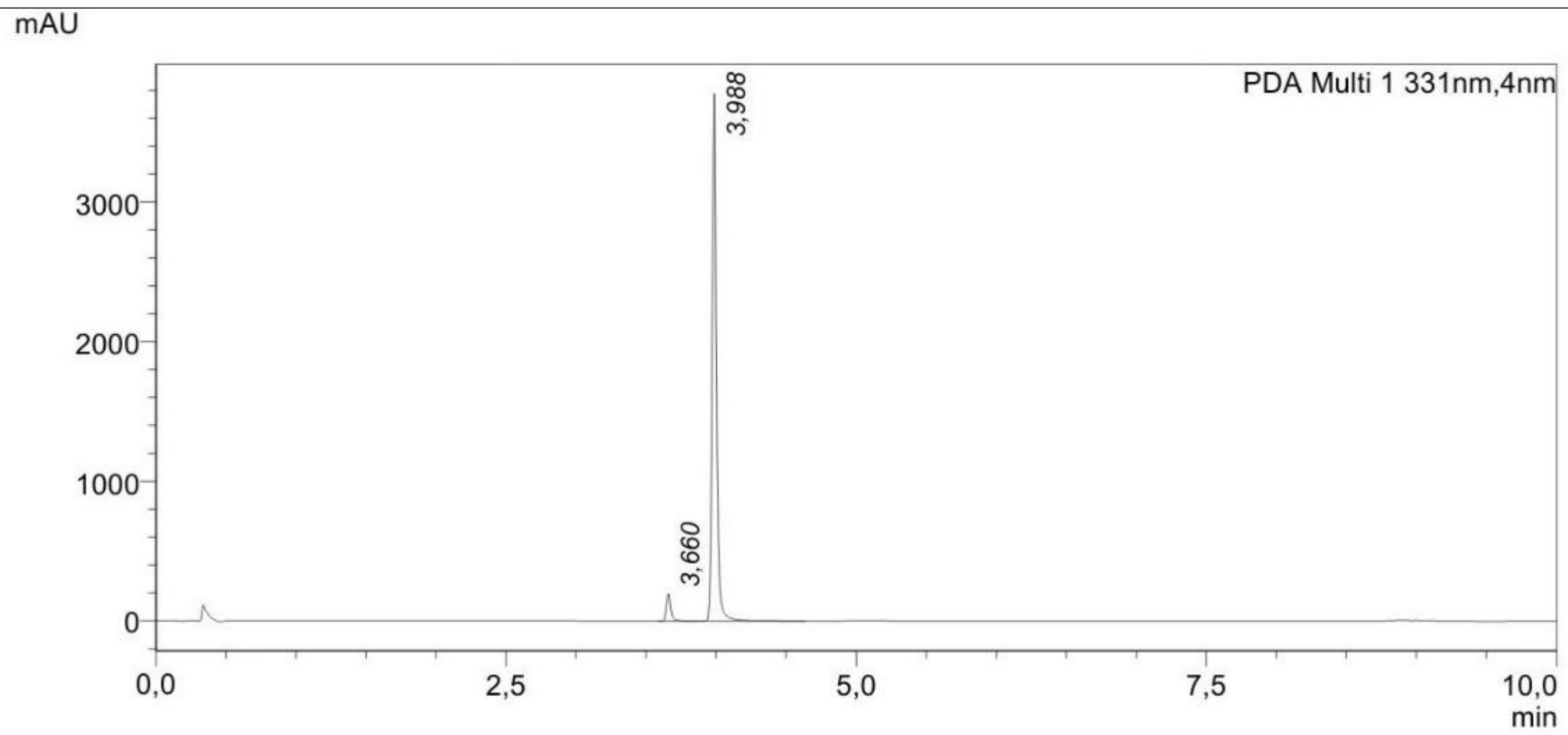

<PeakTable>

PDA Ch1 331nm

Peak#	Ret. Time	Area	Area%
1	3,660	443873	5,152
2	3,988	8172303	94,848
Total		8616176	100,000

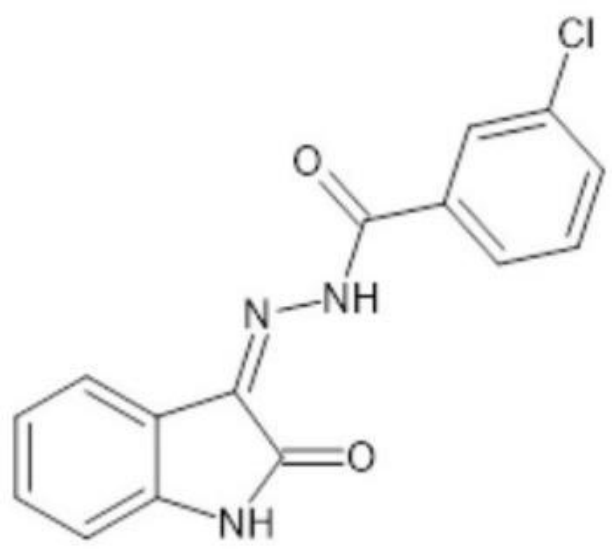

4-chloro-N'-[(3Z)-2-oxo-1,2-dihydro-3H-indol-3-ylidene]benzohydrazide (**1e**)

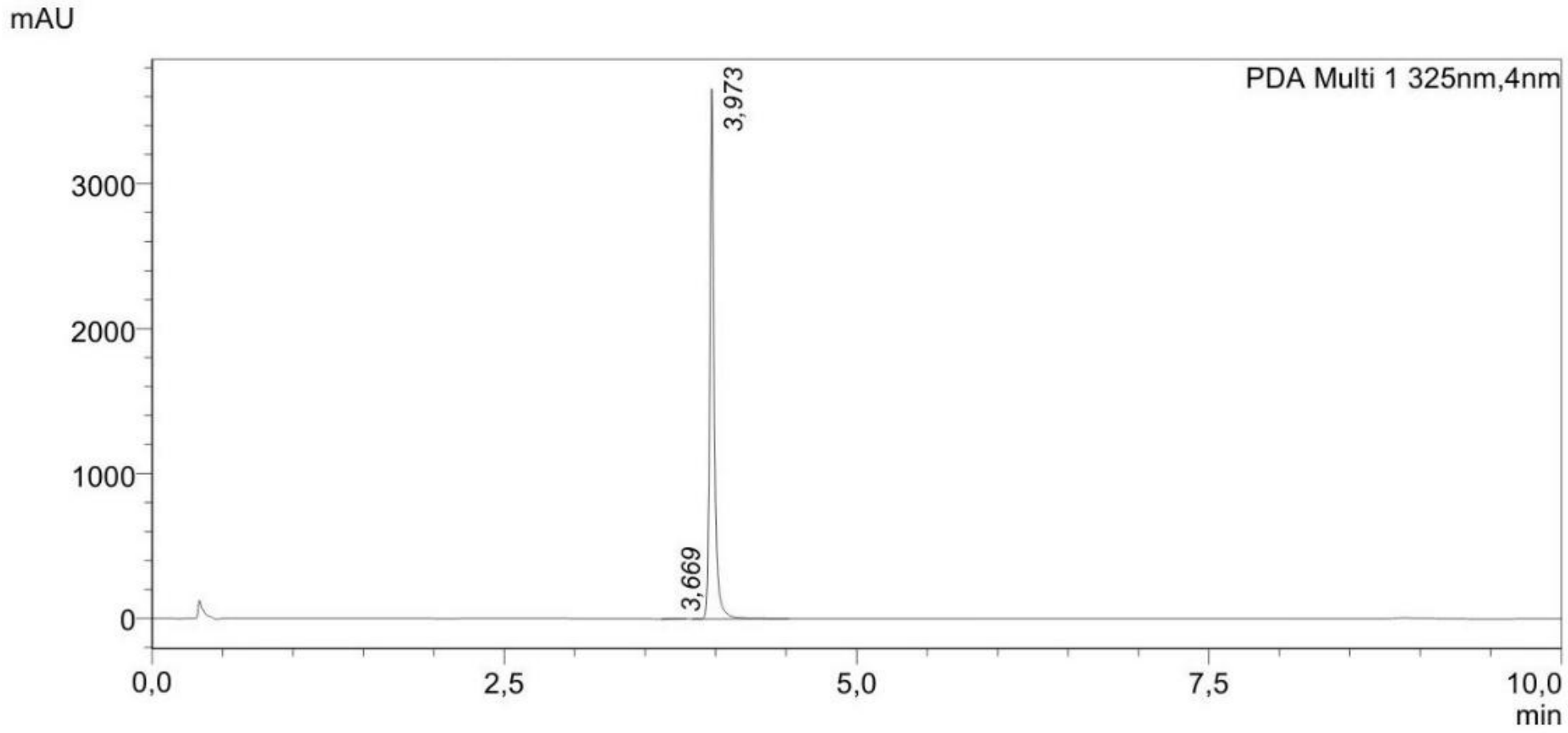

<PeakTable>

PDA Ch1 325nm

Peak#	Ret. Time	Area	Area%
1	3,669	5524	0,069
2	3,973	8035282	99,931
Total		8040806	100,000

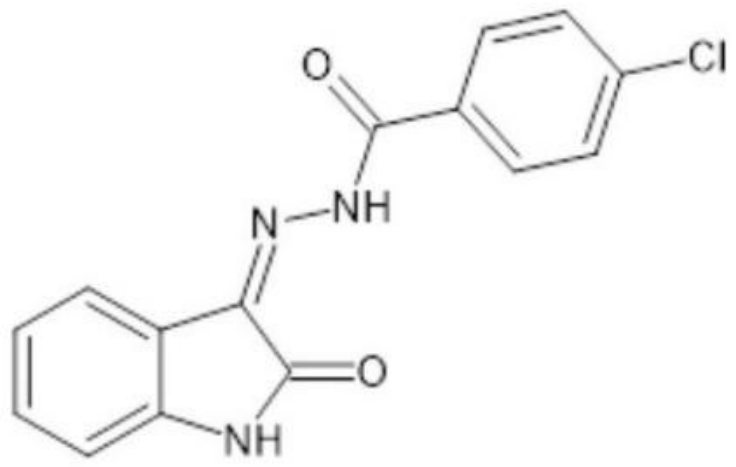

N'-[(3Z)-4-chloro-2-oxo-1,2-dihydro-3H-indol-3-ylidene]benzohydrazide (**2a**)

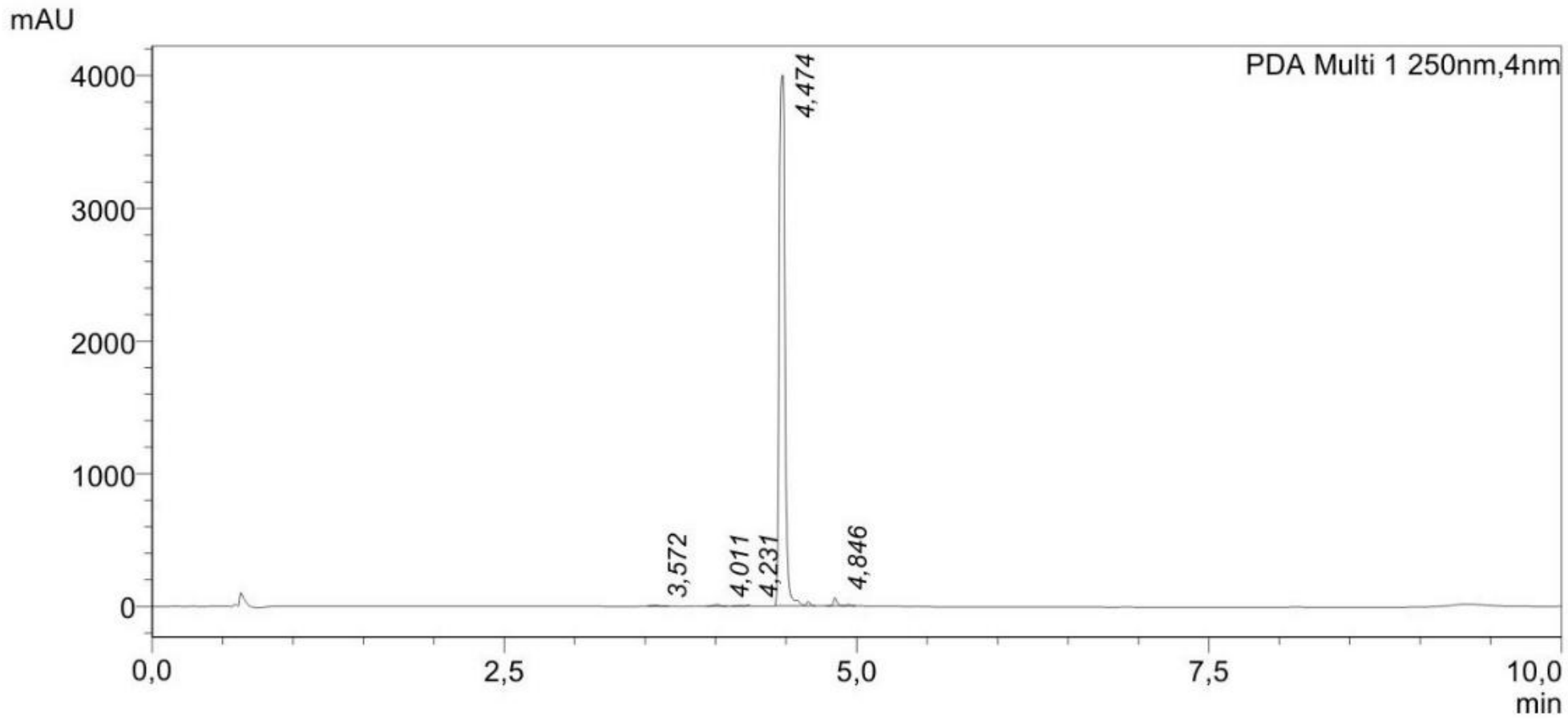

<PeakTable>

PDA Ch1 250nm

Peak#	Ret. Time	Area	Area%
1	3,572	19624	0,172
2	4,011	42939	0,376
3	4,231	10607	0,093
4	4,474	11197415	98,181
5	4,846	134285	1,177
Total		11404870	100,000

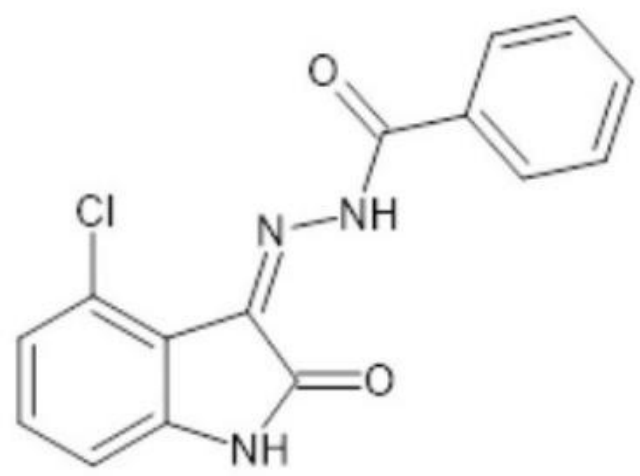

N'-[(3Z)-4-chloro-2-oxo-1,2-dihydro-3H-indol-3-ylidene]-2-hydroxybenzohydrazide (**2b**)

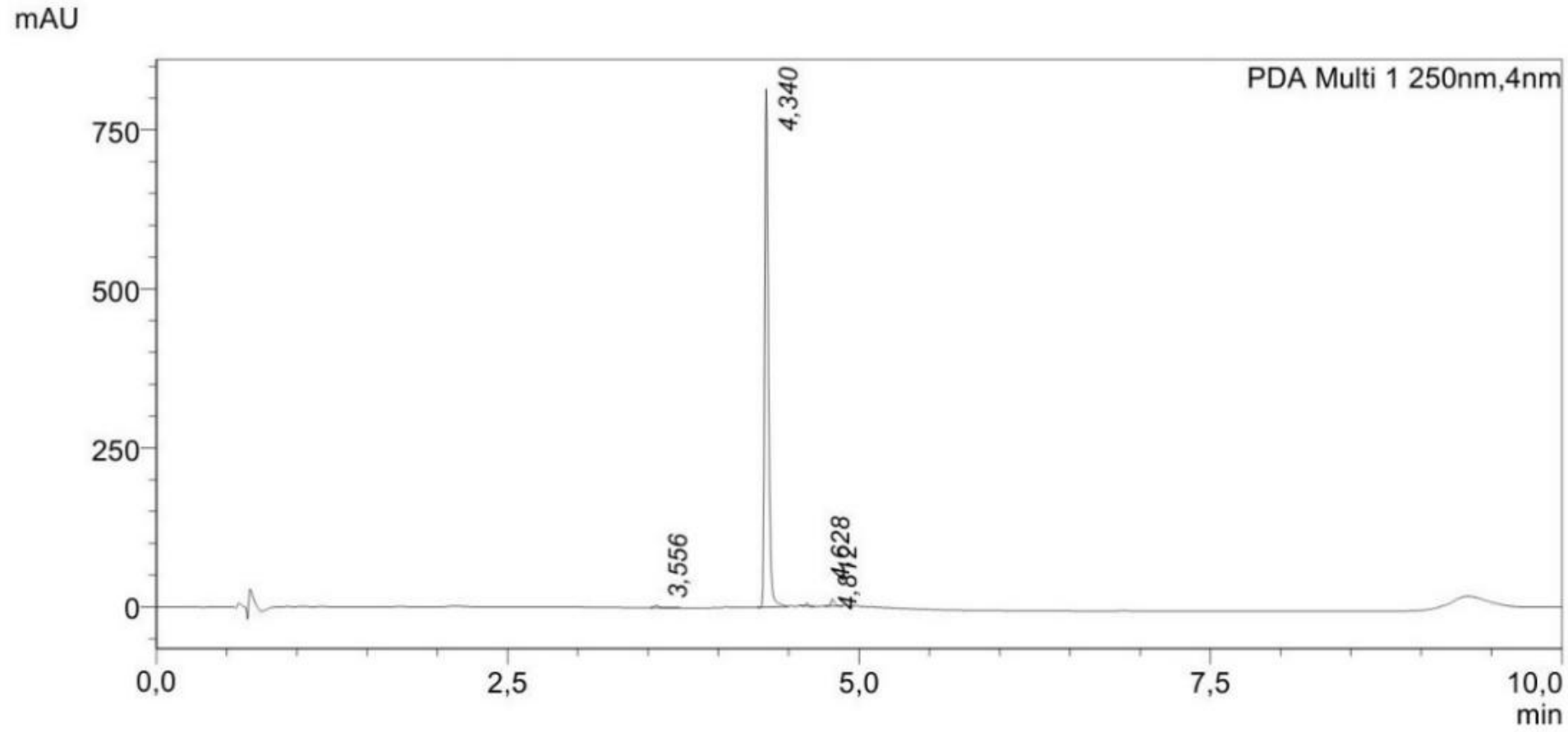

<PeakTable>

PDA Ch1 250nm

Peak#	Ret. Time	Area	Area%
1	3,556	7853	0,523
2	4,340	1469790	97,809
3	4,628	5728	0,381
4	4,812	19350	1,288
Total		1502720	100,000

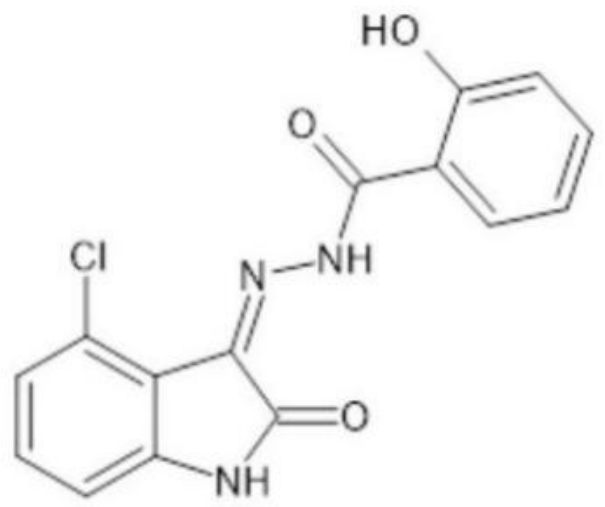

N'-[(3Z)-4-chloro-2-oxo-1,2-dihydro-3H-indol-3-ylidene]-3-hydroxybenzohydrazide (**2c**)

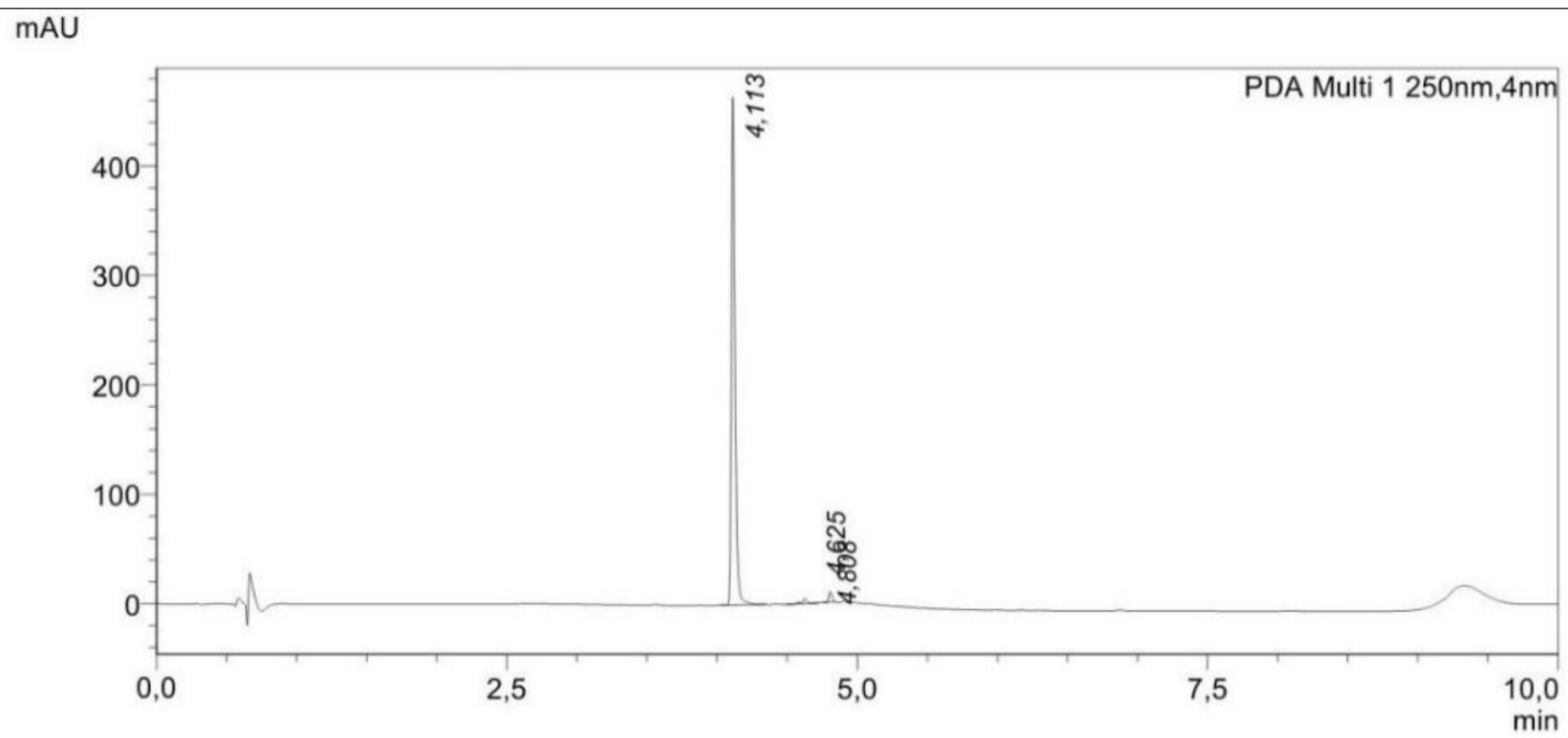

<PeakTable>

PDA Ch1 250nm

Peak#	Ret. Time	Area	Area%
1	4,113	854019	97,392
2	4,625	7814	0,891
3	4,808	15052	1,717
Total		876884	100,000

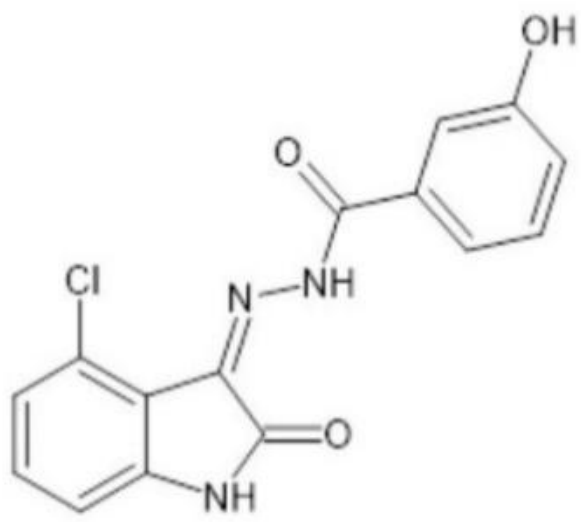

4-chloro-N'-[(3Z)-4-chloro-2-oxo-1,2-dihydro-3H-indol-3-ylidene]benzohydrazide (**2d**)

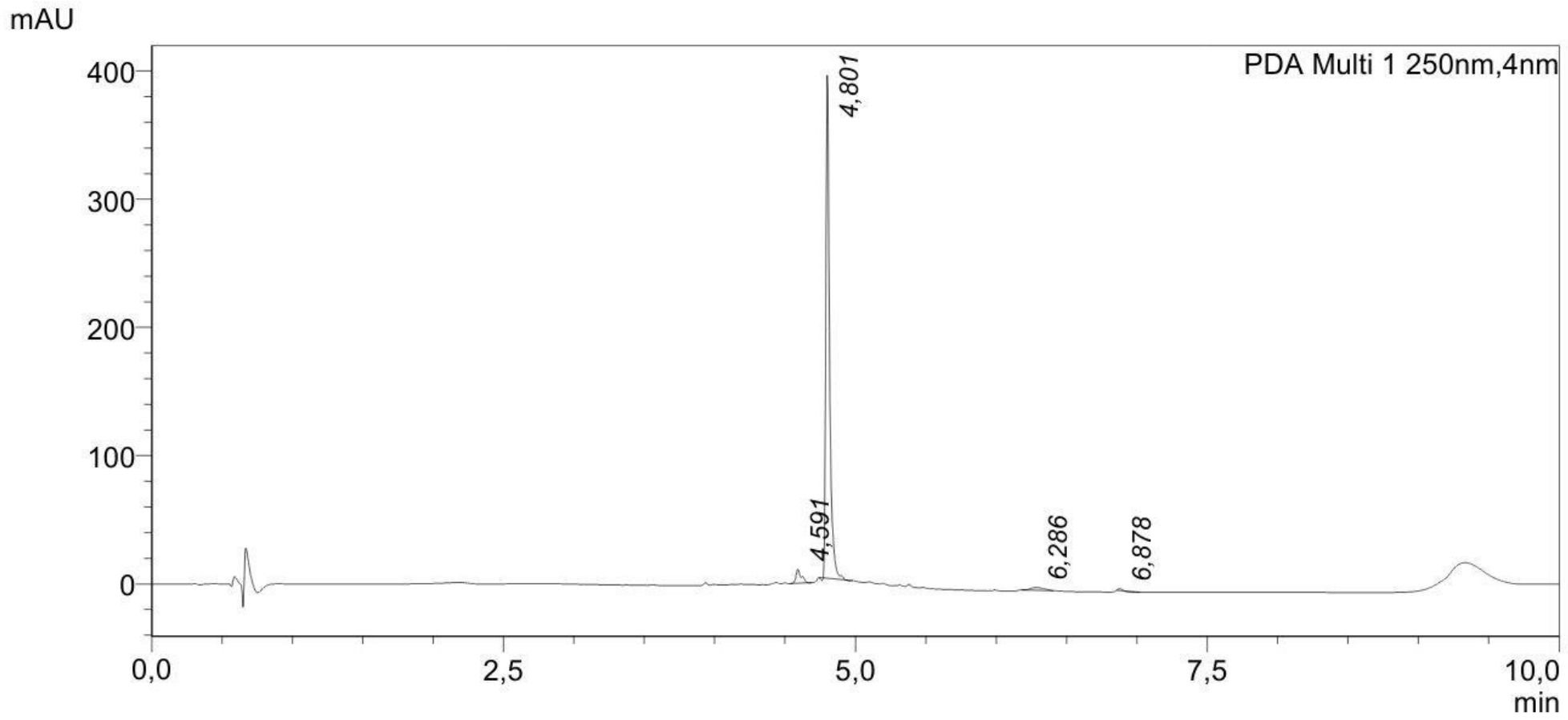

<PeakTable>

PDA Ch1 250nm

Peak#	Ret. Time	Area	Area%
1	4,591	27668	3,604
2	4,801	726318	94,604
3	6,286	13156	1,714
4	6,878	603	0,079
Total		767744	100,000

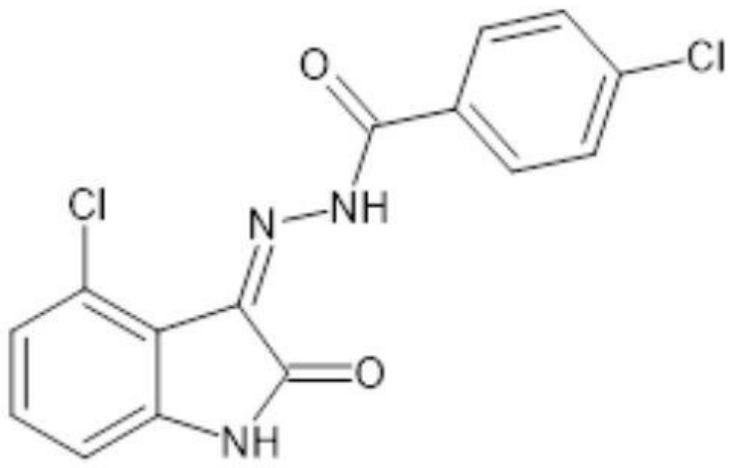

N'-[(3Z)-5-chloro-2-oxo-1,2-dihydro-3H-indol-3-ylidene]benzohydrazide (**3a**)

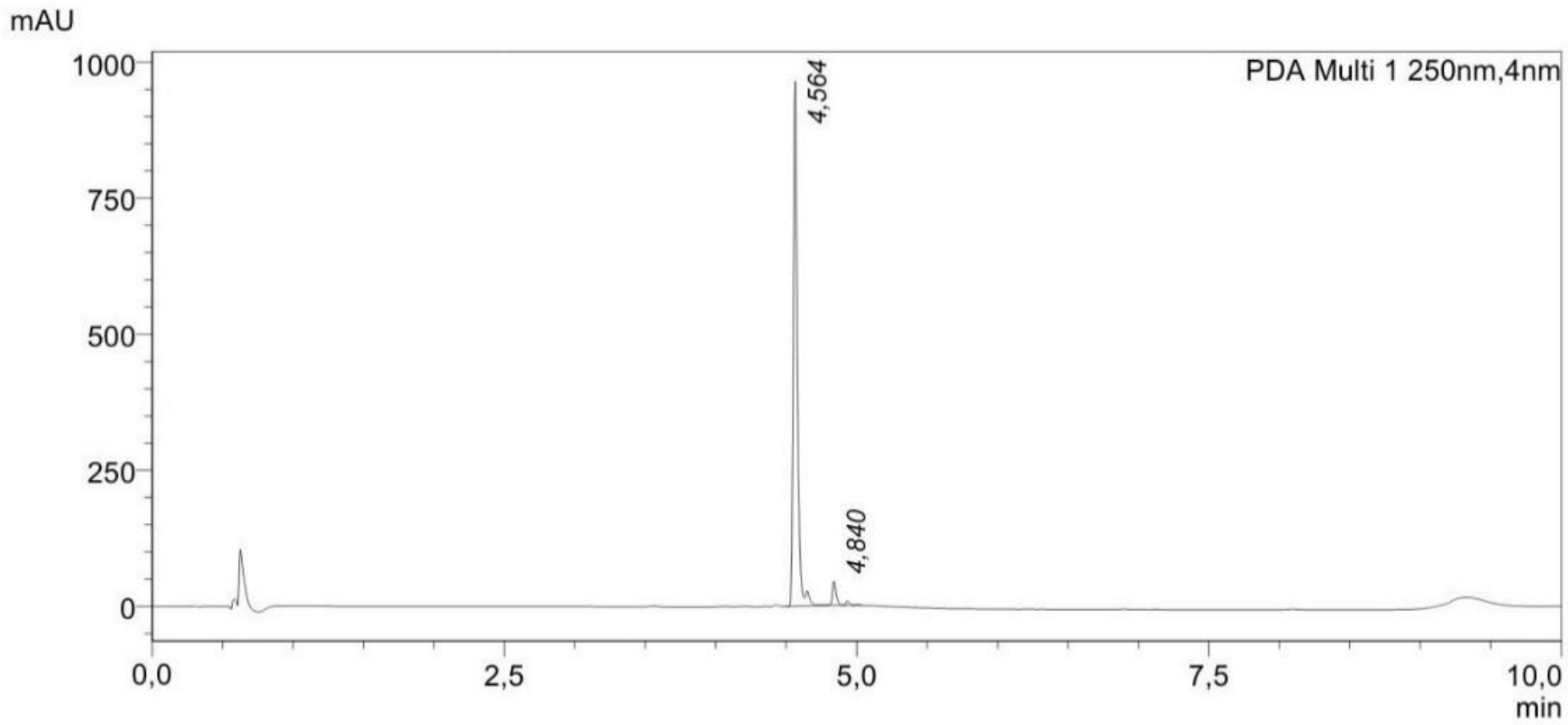

<PeakTable>

PDA Ch1 250nm

Peak#	Ret. Time	Area	Area%
1	4,564	1964213	95,292
2	4,840	97046	4,708
Total		2061259	100,000

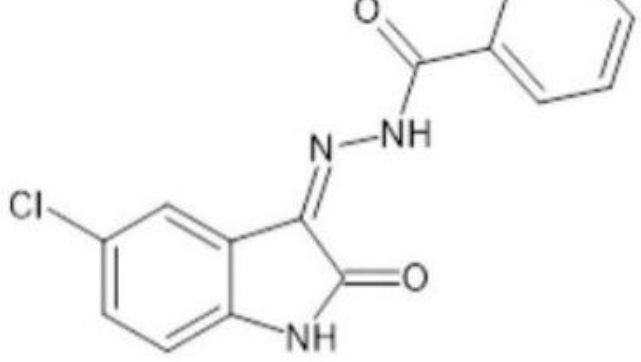

N'-[(3Z)-5-chloro-2-oxo-1,2-dihydro-3H-indol-3-ylidene]-3-hydroxybenzohydrazide (**3b**)

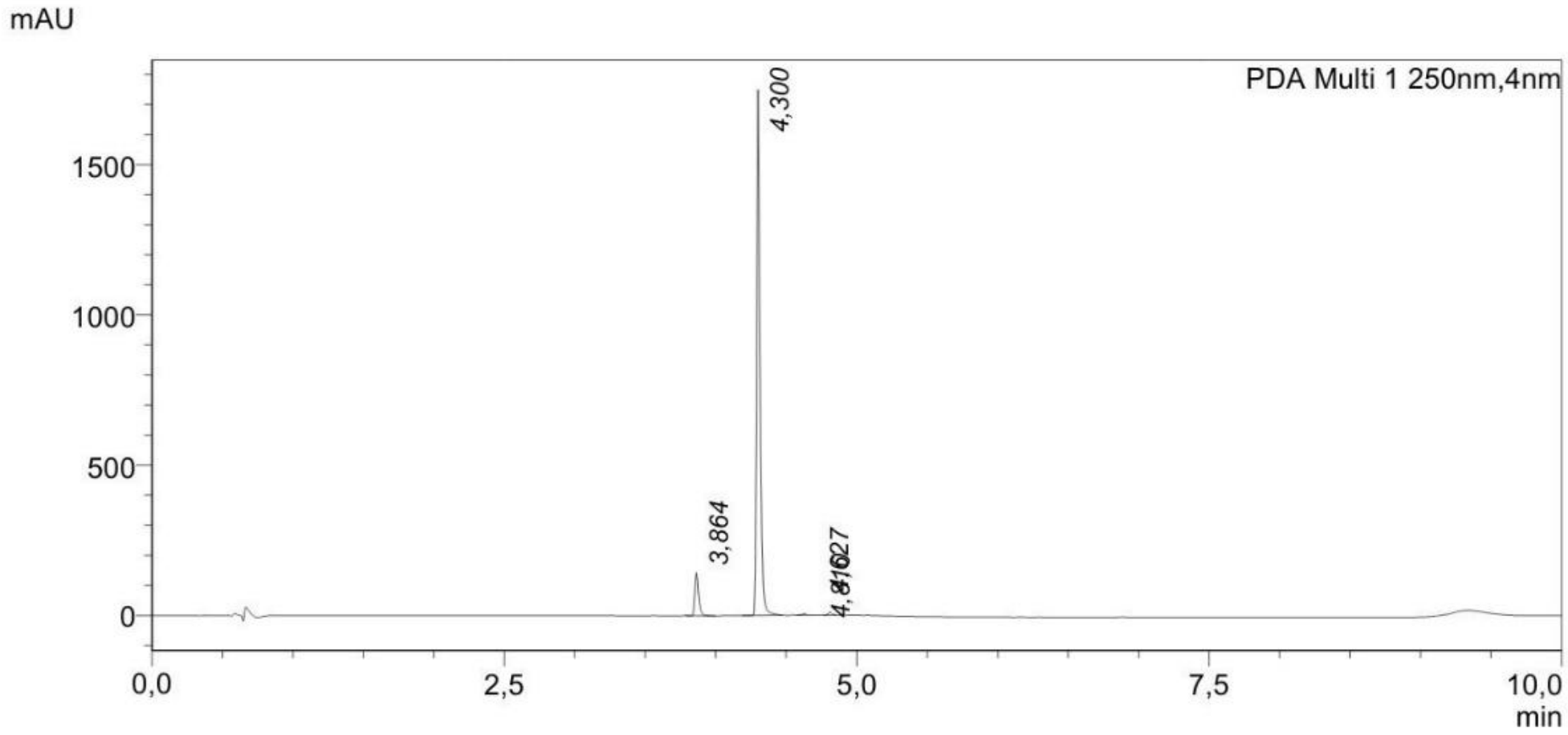

<PeakTable>

PDA Ch1 250nm

Peak#	Ret. Time	Area	Area%
1	3,864	258936	7,897
2	4,300	2997038	91,398
3	4,627	6785	0,207
4	4,810	16331	0,498
Total		3279090	100,000

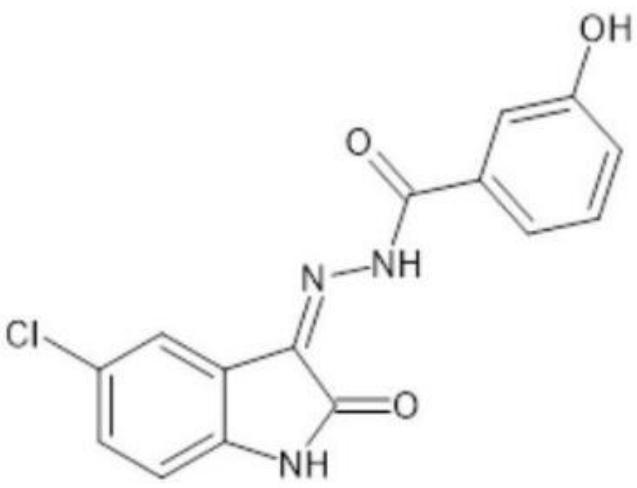

2-chloro-N'-[(3Z)-5-chloro-2-oxo-1,2-dihydro-3H-indol-3-ylidene]benzohydrazide (**3c**)

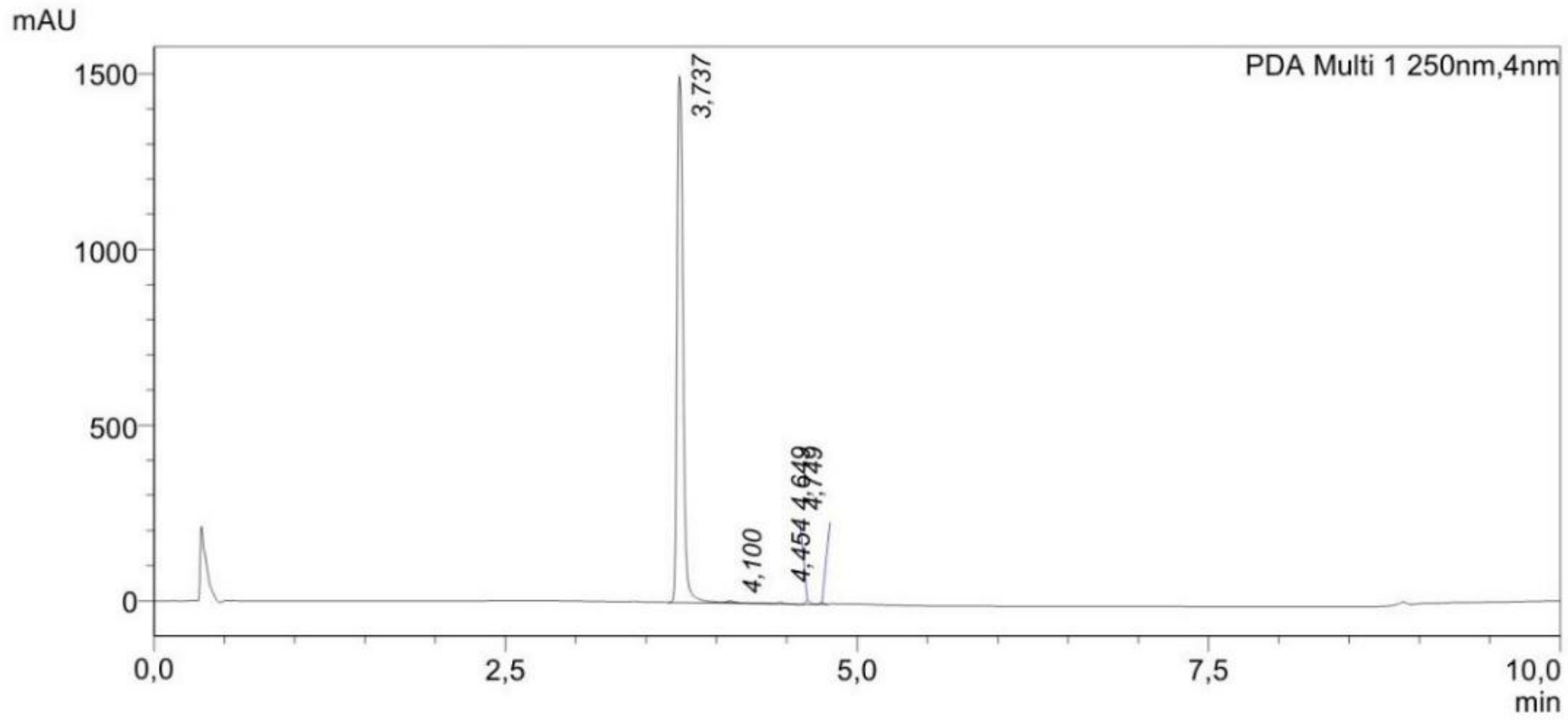

<PeakTable>

PDA Ch1 250nm

Peak#	Ret. Time	Area	Area%
1	3,737	4469922	98,615
2	4,100	12834	0,283
3	4,454	12421	0,274
4	4,649	26238	0,579
5	4,749	11284	0,249
Total		4532698	100,000

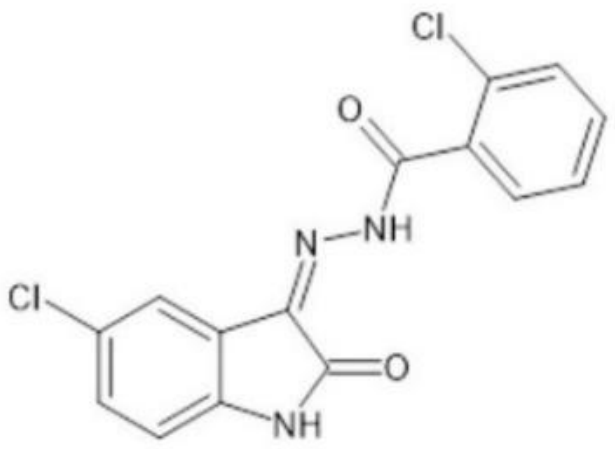

N'-[(3Z)-6-chloro-2-oxo-1,2-dihydro-3H-indol-3-ylidene]benzohydrazide (**4a**)

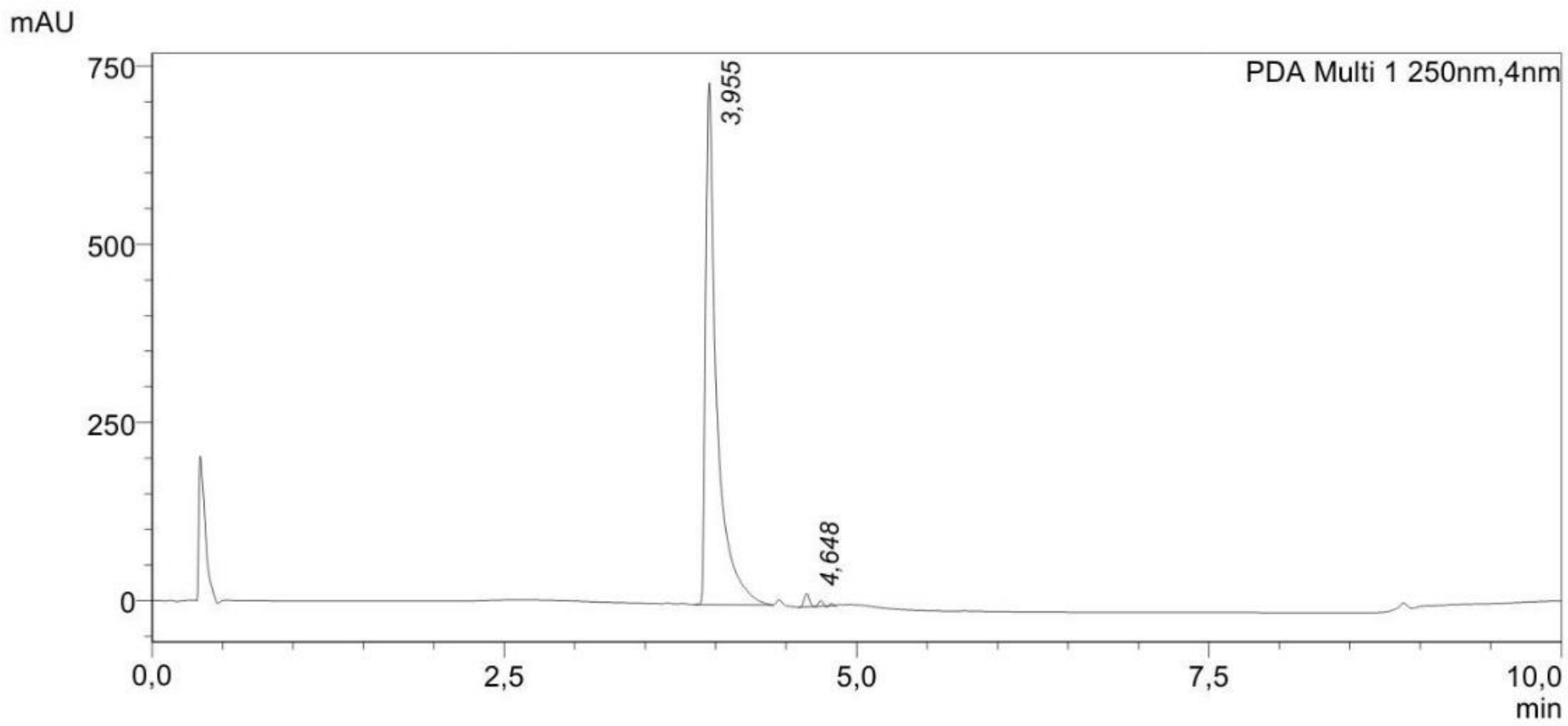

<PeakTable>

PDA Ch1 250nm

Peak#	Ret. Time	Area	Area%
1	3,955	4018966	98,416
2	4,648	64692	1,584
Total		4083659	100,000

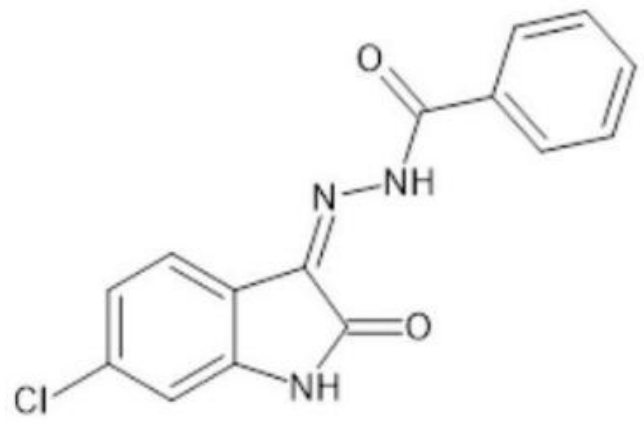

N'-[(3Z)-6-chloro-2-oxo-1,2-dihydro-3H-indol-3-ylidene]-3-hydroxybenzohydrazide (**4b**)

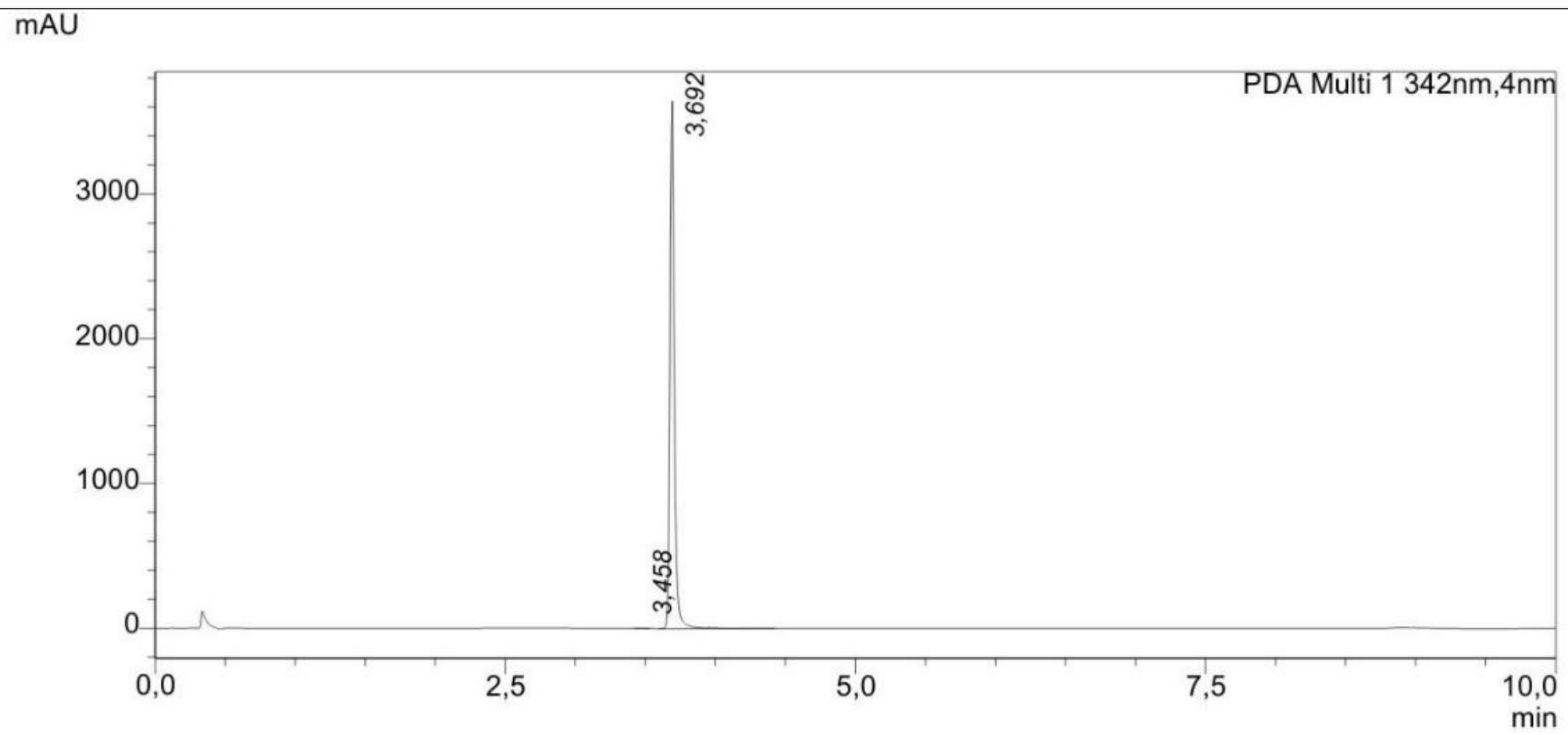

<PeakTable>

PDA Ch1 342nm

Peak#	Ret. Time	Area	Area%
1	3,458	2160	0,028
2	3,692	7809757	99,972
Total		7811917	100,000

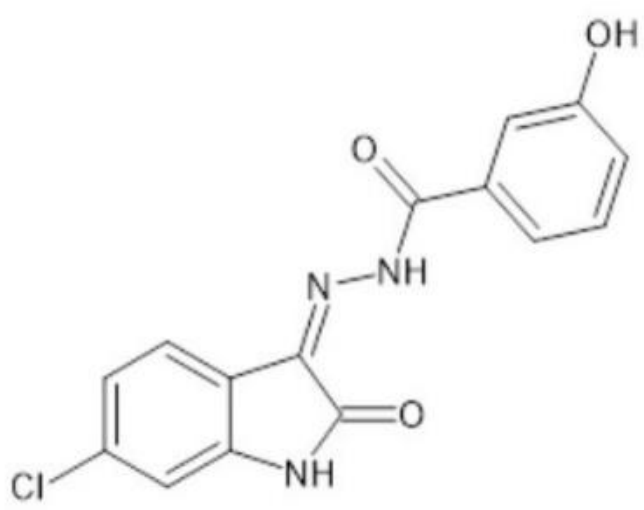

N'-[(3Z)-7-chloro-2-oxo-1,2-dihydro-3H-indol-3-ylidene]benzohydrazide (**5a**)

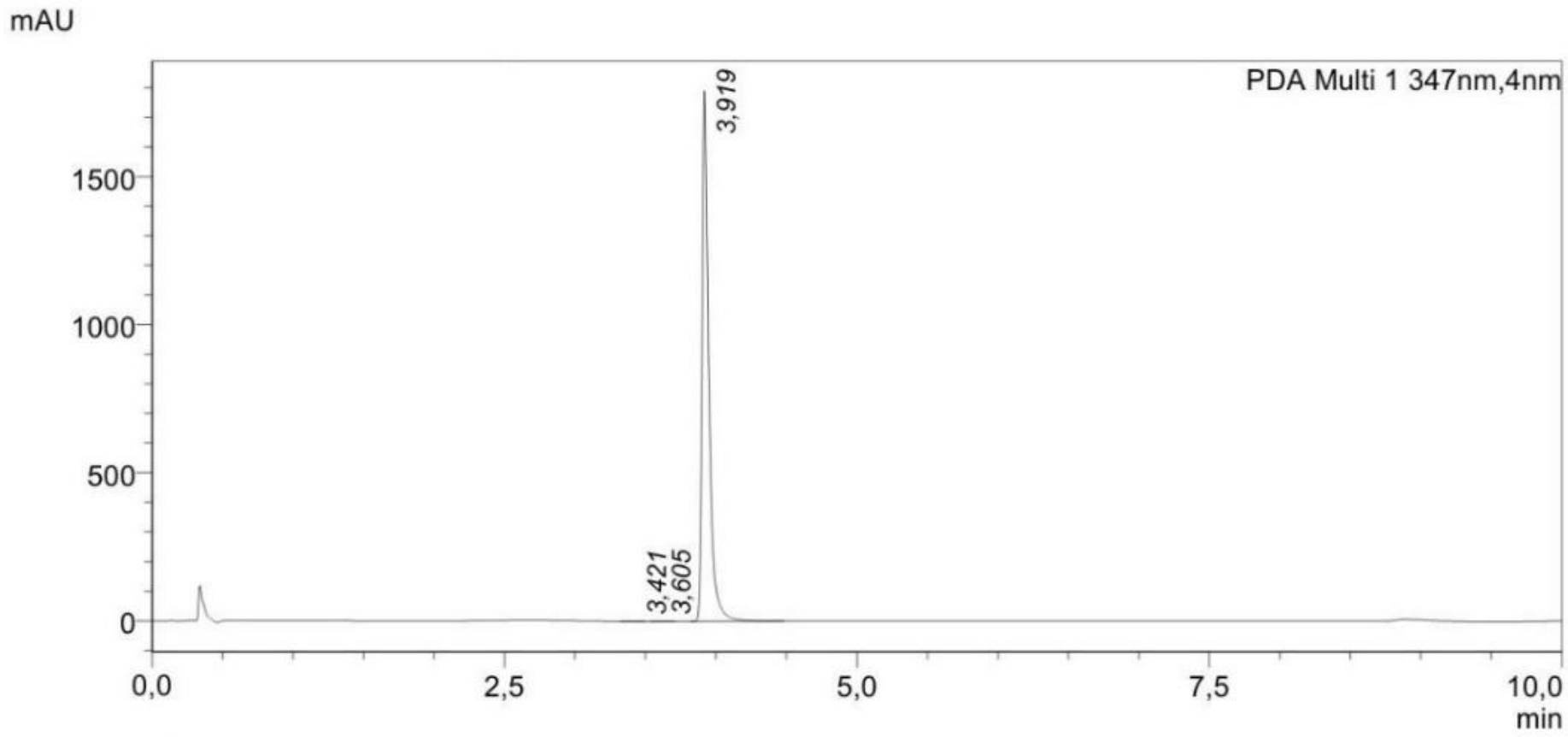

<PeakTable>

PDA Ch1 347nm

Peak#	Ret. Time	Area	Area%
1	3,421	1488	0,025
2	3,605	1314	0,022
3	3,919	5912970	99,953
Total		5915773	100,000

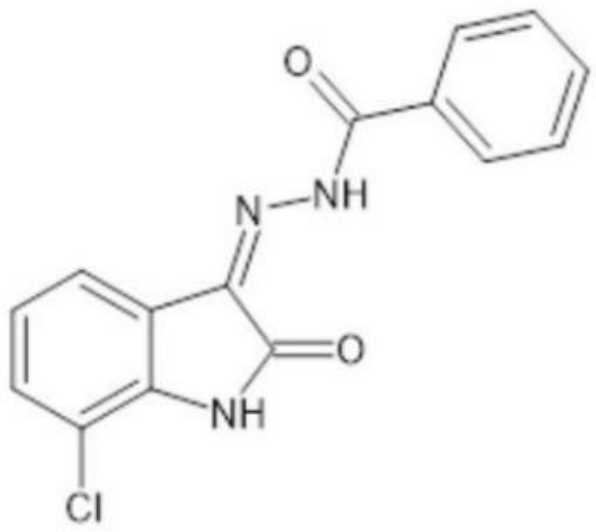

N'-[(3Z)-7-chloro-2-oxo-1,2-dihydro-3H-indol-3-ylidene]-3-hydroxybenzohydrazide (**5b**)

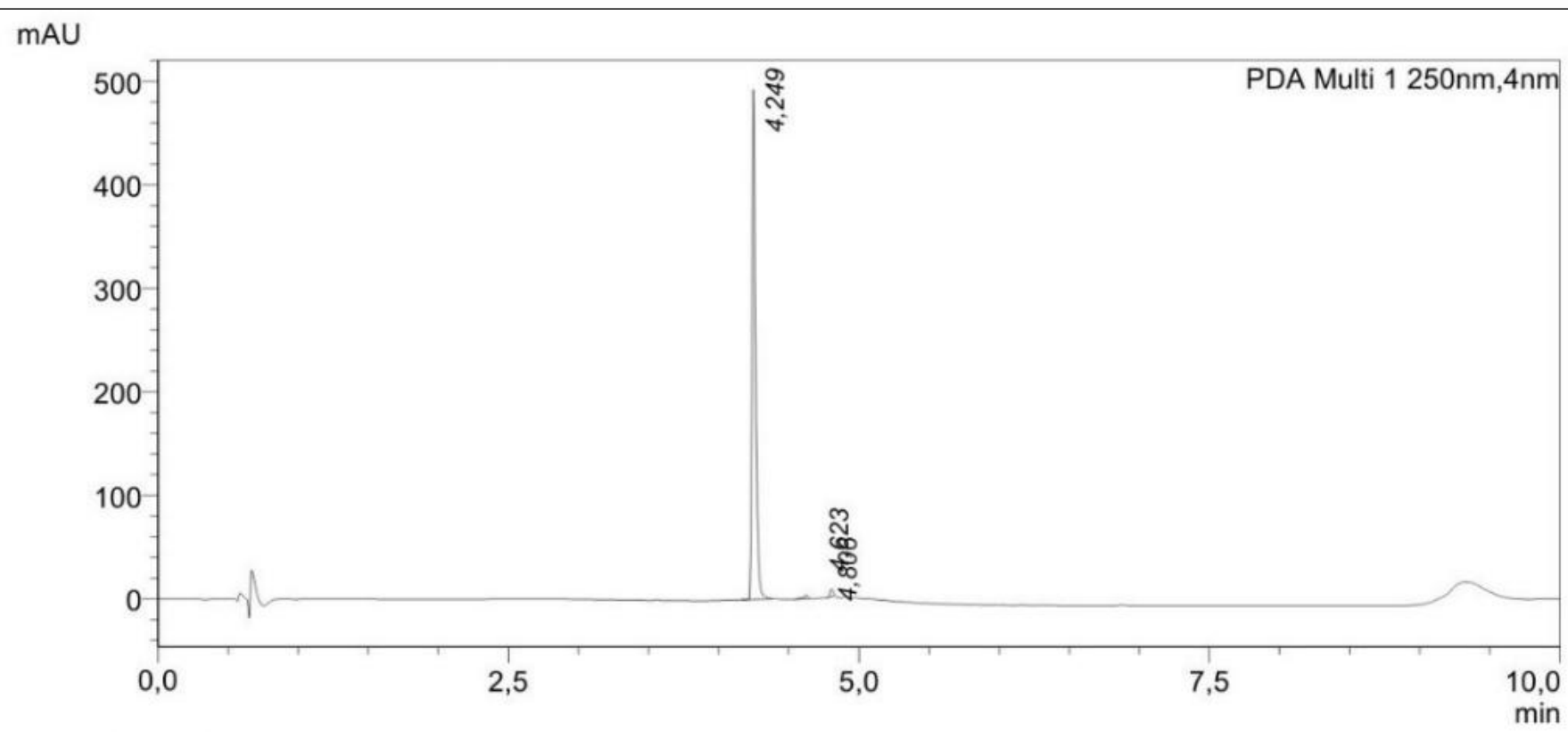

<PeakTable>

PDA Ch1 250nm

Peak#	Ret. Time	Area	Area%
1	4,249	902265	98,039
2	4,623	8450	0,918
3	4,806	9599	1,043
Total		920314	100,000

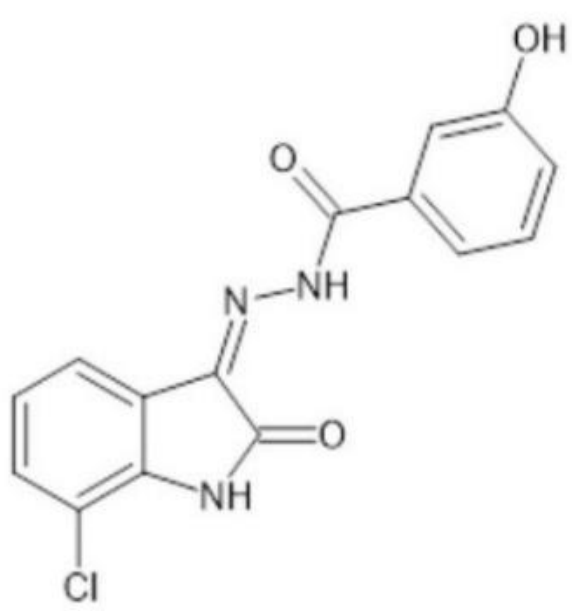

2-chloro-N'-[(3Z)-7-chloro-2-oxo-1,2-dihydro-3H-indol-3-ylidene]benzohydrazide (**5c**)

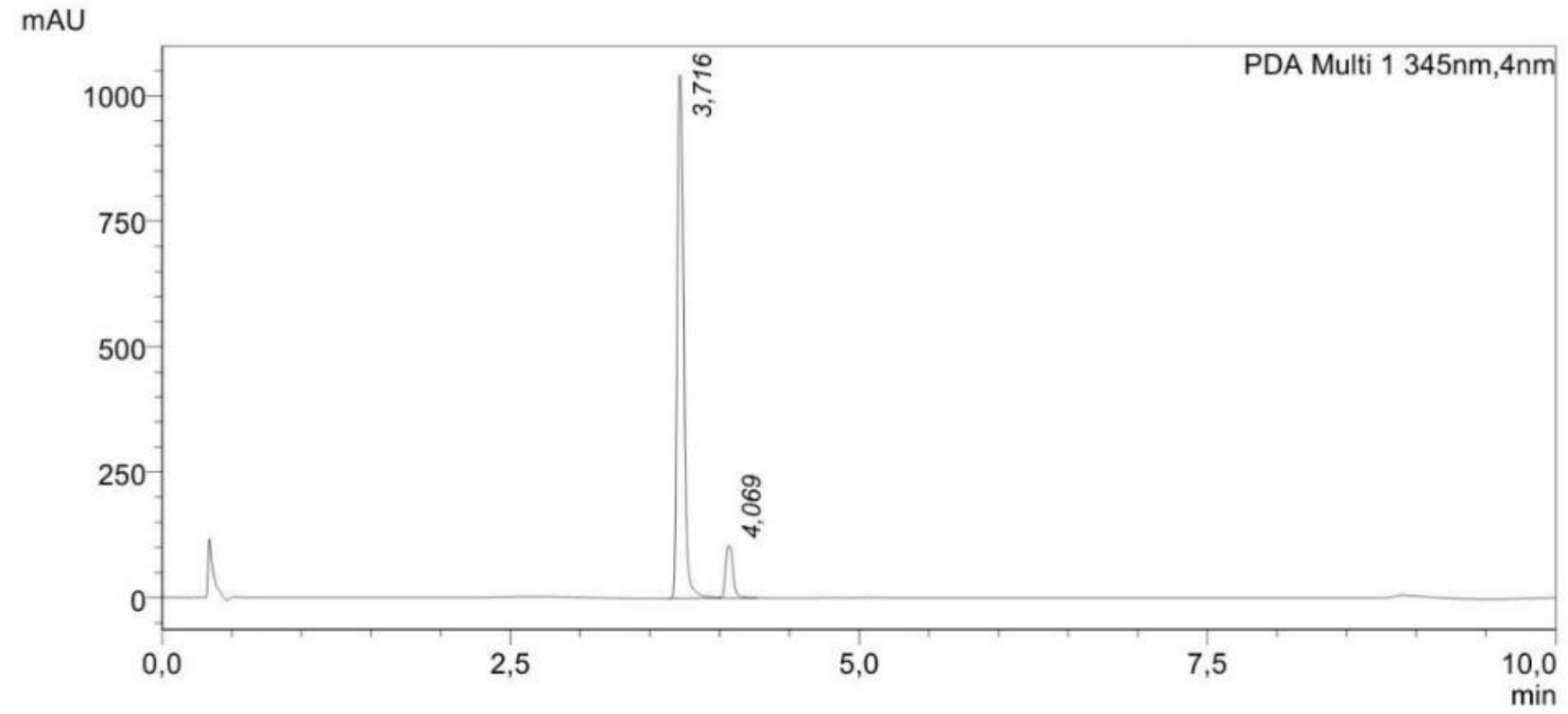

<PeakTable>

PDA Ch1 345nm

Peak#	Ret. Time	Area	Area%
1	3,716	3486072	90,048
2	4,069	385292	9,952
Total		3871364	100,000

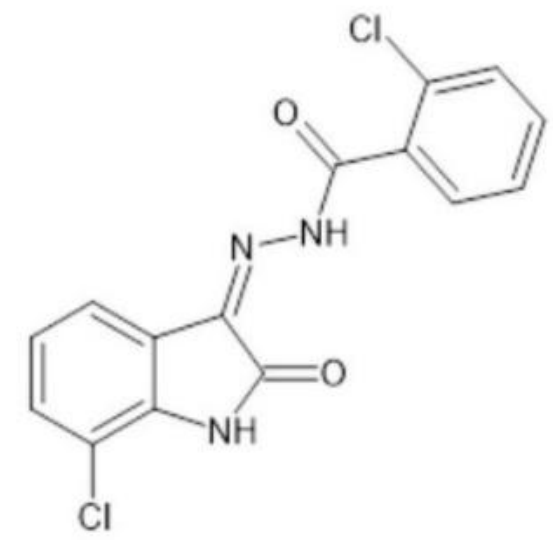

3-chloro-N'-[(3Z)-7-chloro-2-oxo-1,2-dihydro-3H-indol-3-ylidene]benzohydrazide (**5d**)

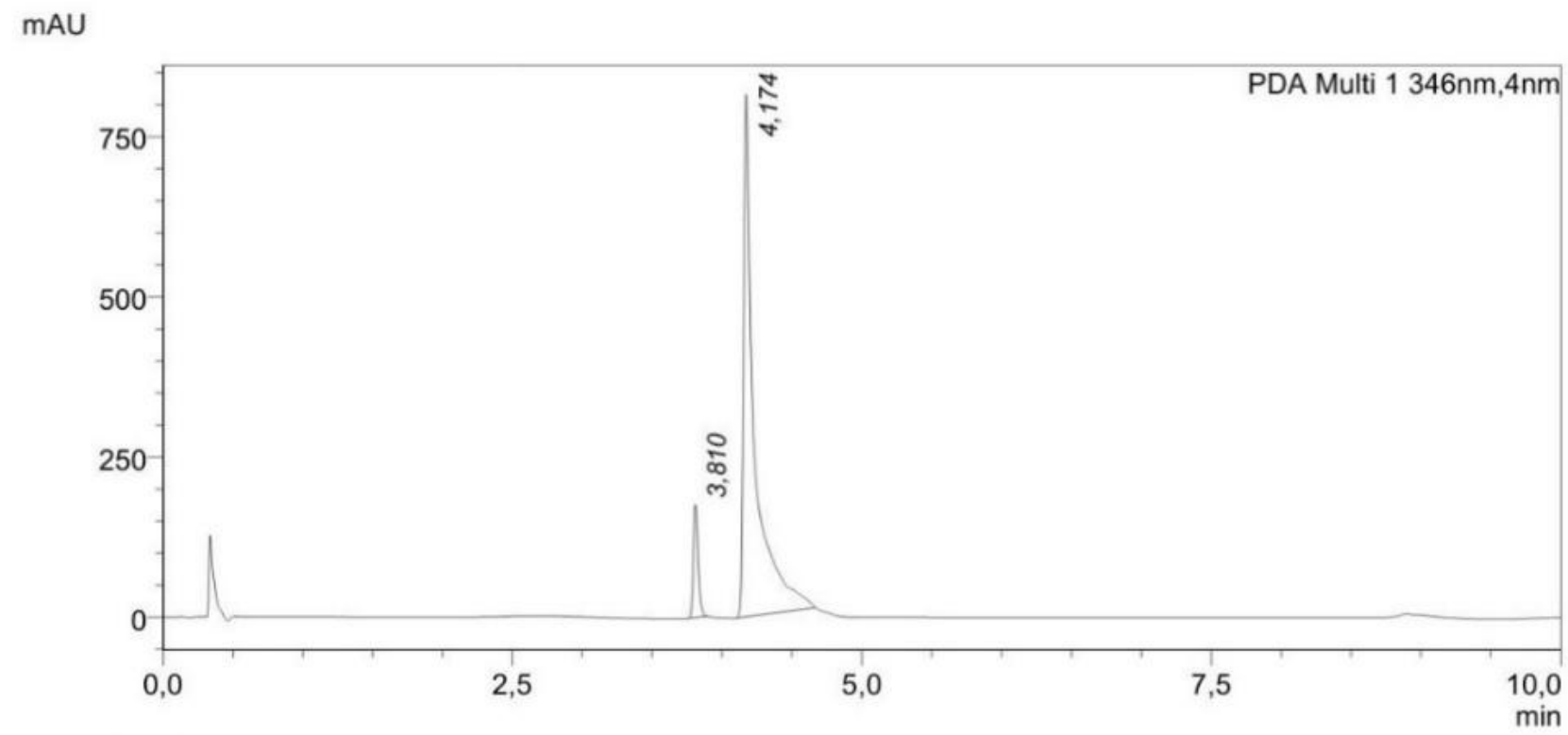

<PeakTable>

PDA Ch1 346nm

Peak#	Ret. Time	Area	Area%
1	3,810	424767	8,603
2	4,174	4512390	91,397
Total		4937157	100,000

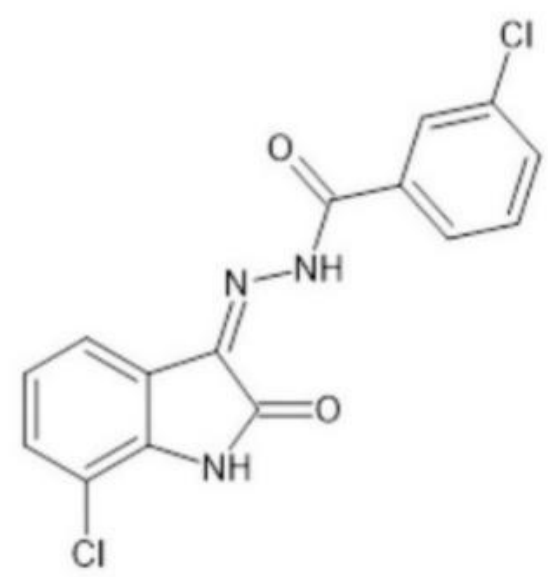

4-chloro-N'-[(3Z)-7-chloro-2-oxo-1,2-dihydro-3H-indol-3-ylidene] benzohydrazide (**5e**)

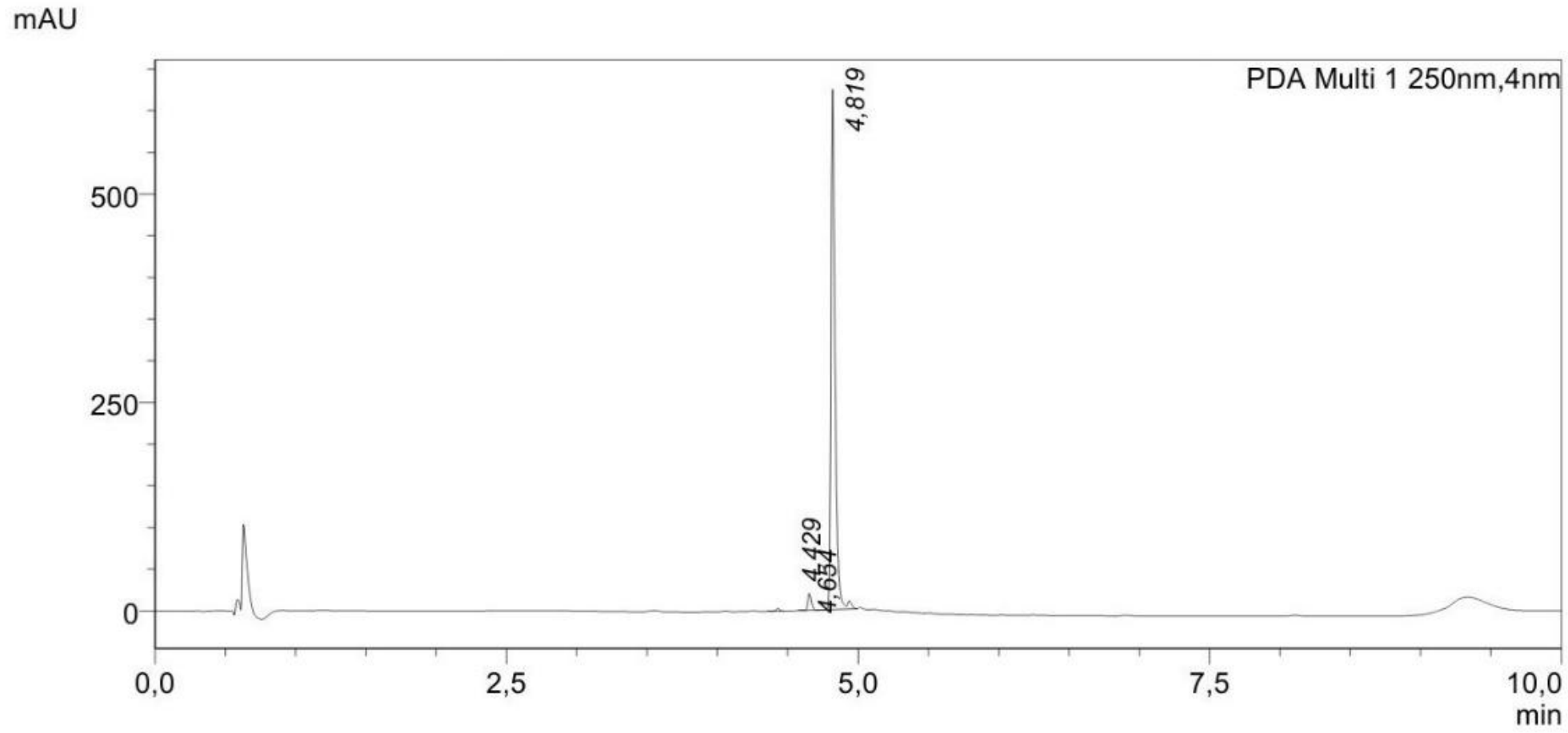

<PeakTable>

PDA Ch1 250nm

Peak#	Ret. Time	Area	Area%
1	4,429	5533	0,418
2	4,654	34102	2,576
3	4,819	1283956	97,006
Total		1323590	100,000

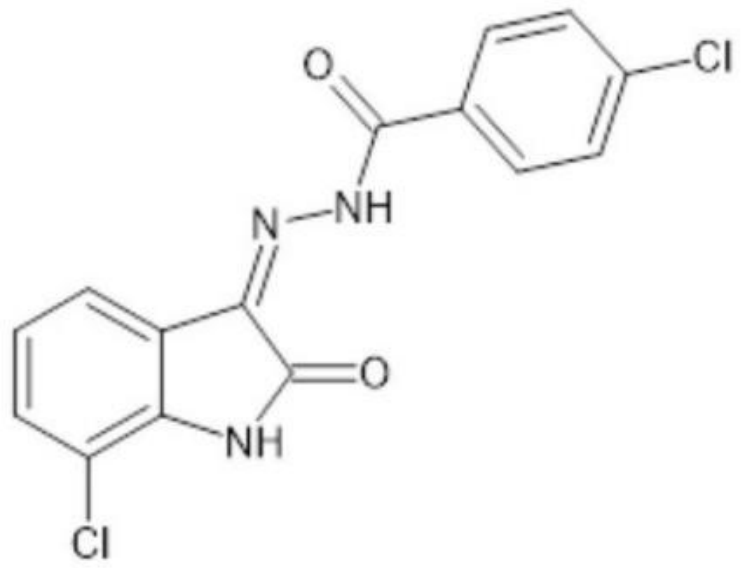

Supplement: Supplementary file 1 [file CMDC-21-e70437-s001.pdf]
